# High-Throughput Screening of 3-Dimensional Co-culture Hair Follicle Mimetic Tissue with an Enhanced Extracellular Matrix for the Screening of Hair Growth-Promoting Compounds

**DOI:** 10.34133/bmr.0125

**Published:** 2024-12-27

**Authors:** Huyen T. M. Pham, Hyo-Sop Kim, Duc Long Nguyen, Hyun Woo Joo, Min Kyu Kim, Young Kwan Sung, Minh Hung Vu, Heung Sik Hahm, Woo Jung Kim, Jae-Ho Kim, Hyun-Ji Park

**Affiliations:** ^1^ Department of Molecular Science and Technology, Ajou University, Suwon 16499, South Korea.; ^2^ Department of Immunology, School of Medicine, Kyungpook National University, Daegu 41944, South Korea.; ^3^ Uppthera, Incheon 21988, South Korea.; ^4^ Ellead Co. Ltd. Skin Bio Research, Seongnam 13590, South Korea.; ^5^ Advanced College of Bio-Convergence Engineering, Ajou University, Suwon 16499, South Korea.

## Abstract

Hair follicle cells reside within a complex extracellular matrix (ECM) environment in vivo, where physical and chemical cues regulate their behavior. The ECM is crucial for hair follicle development and regeneration, particularly through epithelial–mesenchymal interactions. Current in vitro models often fail to replicate this complexity, leading to inconsistencies in evaluating hair loss treatments. Advanced 3-dimensional (3D) culture systems that better mimic in vivo ECM dynamics are needed for more effective therapeutic assessments. Here, we introduce a 3D co-culture system designed to replicate in vivo ECM dynamics. The system incorporates primary dermal papilla cells from human patients, co-cultured with neonatal keratinocytes. This platform facilitates uniform spheroid formation through cell sliding and aggregation, enabling the evaluation of approximately 60 spheroids per well. The model is optimized for high-throughput screening, allowing precise assessments of hair-loss-inducing compounds under consistent conditions. We successfully generated dermal papilla cell and keratinocyte spheroids that closely resemble the native ECM structure, providing an optimal microenvironment for studying hair follicle biology. The 3D co-culture model supported efficient spheroid formation with consistent cellular organization and polarization, along with enhanced ECM-related gene expression crucial for hair follicle regeneration. Uniform spheroid formation and reproducibility were demonstrated across experiments. Overall, the novel 3D co-culture system provides a robust platform for replicating in vivo-like ECM conditions, enabling effective assessment of potential hair loss treatments through epithelial–mesenchymal interactions. Its high-throughput capacity, combined with reproducibility and ease of use, makes it a valuable tool for screening therapeutic candidates and advancing hair loss treatment development.

## Introduction

Hair loss, or alopecia, is a prevalent condition with complex, multifactorial causes, including genetic predisposition, aging, hormonal imbalances, immune system reactions, psychological stress, and medication use [1,2]. The profound sociopsychological impact of hair loss highlights the urgent need for effective prevention and treatment strategies. Current pharmaceutical and cosmetic treatments, while widely used, often demonstrate limited efficacy and are associated with undesirable side effects, underscoring the need for safer and more effective alternatives [3,4]. The limitations of existing treatments have driven notable interest in developing advanced therapeutic approaches, particularly through the use of innovative in vitro models that more accurately simulate the in vivo environment of hair follicles (HFs) [4].

Traditional 2-dimensional (2D) cell culture models, although useful for basic cellular studies, fail to replicate the complex 3-dimensional (3D) microenvironment of HFs, often leading to discrepancies between in vitro and in vivo results [5–7]. HF biology relies heavily on dynamic cell–cell and cell–matrix interactions that are difficult to model in 2D systems. In contrast, 3D culture systems have proven more successful in mimicking these interactions, particularly by replicating the extracellular matrix (ECM), which plays a vital role in regulating cellular behavior such as proliferation, differentiation, and migration [8,9]. These 3D models are crucial for studying dermal papilla cells (DPCs), which are the key regulators of HF growth, development, and regeneration [10,11].

Moreover, conventional 2D monolayer cultures have a substantial limitation in that the hair-inductive potency of human hair follicle dermal papilla cells (HFDPCs) is rapidly lost. Recent reports have highlighted that HFDPCs cultured in 2D conditions exhibit a decline in the expression of key inductive markers such as alkaline phosphatase (ALP), versican, and α smooth muscle actin (α-SMA), leading to reduced ability to induce HF formation. In contrast, 3D culture systems provide a more favorable environment for maintaining the cells’ hair-inductive ability by better mimicking the in vivo microenvironment [12]. Recent studies have demonstrated that 3D spheroid cultures enhance the stemness and long-term expansion capacity of human HFDPCs [13,14]. These findings underscore the importance of 3D culture systems in preserving the functional properties of DPCs, making them crucial for advancing hair regeneration therapies.

Recent advancements in 3D culture models have enabled detailed investigations into the role of DPCs in hair growth, highlighting the importance of ECM simulation in promoting the hair-inducing potential of these cells [15,16]; 3D systems not only promote more physiologically relevant gene expression profiles in DPCs but also enhance their functional properties, such as the secretion of growth factors essential for hair regeneration [4,11]. Additionally, these models provide insights into epithelial–mesenchymal interactions (EMIs), which are fundamental to the formation and cycling of HFs [11,17–25]. The ability of 3D culture systems to simulate ECM conditions and cell–matrix interactions makes them superior to 2D models in evaluating therapeutic compounds and predicting their in vivo effects [26,27].

Given these advantages, several biomimetic 3D culture systems have been developed to replicate the in vivo conditions required for HF formation and regeneration. These systems support the long-term maintenance of cellular characteristics, enabling the study of HF dynamics over extended periods [26]. For example, 3D models have been used to investigate the effects of growth factors, cytokines, and other bioactive compounds on DPC function, leading to breakthroughs in understanding the molecular mechanisms of hair growth and HF regeneration [28,29]. Furthermore, the development of high-throughput 3D culture systems has enabled more efficient screening of therapeutic candidates, reducing the gap between in vitro testing and clinical application [26,27].

In this study, we introduce a 3D co-culture system named PAMCELL, designed to induce rapid spheroid formation through cell sliding, enabling a more effective assessment of potential hair loss treatments. PAMCELL incorporates primary human DPCs co-cultured with neonatal keratinocytes, creating uniform spheroids that replicate the natural structure and function of HFs. The radially patterned design of the PAMCELL platform enhances ECM-related functions, promoting cell–cell and cell–matrix interactions that are crucial for HF regeneration. By fostering rapid spheroid formation, PAMCELL supports the formation of viable, polarized dermal papilla (DP) spheroids that exhibit enhanced ECM production and up**-**regulated gene expression linked to hair growth and follicular development.

Our findings demonstrate that PAMCELL provides a notable advantage over conventional methods, such as ultralow attachment (ULA) plates, by offering a more in vivo-like environment that accelerates spheroid formation and improves the functional properties of DP cells. PAMCELL-derived DP spheroids successfully induced hair growth in vivo, underscoring the platform’s potential for regenerative medicine applications. Additionally, PAMCELL’s high-throughput capabilities make it an ideal system for screening hair-loss-inducing compounds and testing the efficacy of various therapeutic candidates. This study highlights PAMCELL as a robust tool for advancing the development of new hair loss treatments by providing a comprehensive, efficient, and physiologically relevant platform for studying HF biology and regeneration.

## Materials and Methods

### Fabrication of 3D culture plate substrates

A circular radial-type pattern was designed using AutoCAD 2018. Bare silica particles and peptides, similar to the ones used in the study by Lee et al. [30], were employed, but the substrate material was modified to cyclo olefin polymer and polyethylene glycol. Figure S1 illustrates the main process of plate fabrication summarized in 3 key steps: (a) polydimethylsiloxane patterning molding, (b) particle patterning using 700-μm silica particles functionalized with RGD peptide, and (c) demolding to remove the cured polydimethylsiloxane layer after ultraviolet exposure. The final products underwent sterilization using ethylene oxide gas, were packed in aluminum. The plates were opened and exposed in a cell culture incubator for a minimum of 24 h before use for cell seeding.

### DP isolation

Residual human HF tissues were provided by the Kyungpook National University Hair Transplantation Center after autologous transplantation surgery with patient consent. Human DPCs were isolated from the dissected HF bulbs and transferred onto plastic dishes of low-glucose Dulbecco’s modified Eagle medium (DMEM) supplemented with penicillin–streptomycin (PS) and 20% heat-inactivated fetal bovine serum, and incubated at 37 °C with 5% CO_2_. Throughout an 18-d incubation period, the medium was replaced every 3 d as DPC flourished into the attached DP. DPCs were harvested using 0.25% trypsin/10 mM EDTA and subcultured at a split ratio of 1:3. Subsequently, DPCs were maintained in a DMEM maintenance medium including low-glucose DMEM with PS, 4 ng/ml fibroblast growth factor-basic, and 10% fetal bovine serum and used in the experiment up to passage 4. Biopsy specimens were obtained with written informed consent from the patients. The study was conducted in accordance with the Declaration of Helsinki and approved by the Medical Ethical Committee of Kyungpook National University (IRB Number KNU-2021-0113). Non-balding scalp specimens were obtained from patients undergoing hair transplantation surgery. Informed consent was obtained from all patients.

### Chamber assay

The animal experiments were approved by the Institutional Animal Care and Use Committee at Kyungpook National University (Daegu, Korea). The assessment of the hair-inductive capacity of human DPCs was conducted through a well-established “chamber assay” [29]. In summary, DPCs were cultured in the wells of a 96-well PAMCELL plate with a density of 80,000 cells per well to form uniform-sized spheroids (Video S1). DP spheroids were subsequently mixed with freshly isolated neonatal mouse epidermal cells, which were isolated following a similar protocol as in a previous study [28].

The mixtures were cotransplanted into a silicon chamber, which was subsequently installed on the backs of nude mice to confine the cells within the assay space. The chamber was removed 2 weeks after the initial cell implantation. Approximately 1 week following chamber removal, noticeable HF induction was observed.

### Formation and elongation of HF-like structures

The primary DP cells, isolated from patients as mentioned above, were co-cultivated with neonatal keratinocytes. Primary epidermal neonatal foreskin keratinocytes (HEKn) were purchased from American Type Culture Collection and proliferated on a substrate coated with iMatrix-511 silk at a concentration of 3 μg/ml diluted in phosphate-buffered saline (PBS), incubated at 37 °C for at least 30 min. After removing the coating solution, cells were seeded and maintained under the manufacturer’s instructions, using Dermal Cell Basal Medium plus one Keratinocyte Growth Kit. After reaching optimal density (80% confluence), the cultured DPCs and keratinocyte cells were labeled with fluorochromes (CellTracker CM-DiI Dye and CellTracker Green CMFDA), following the manufacturer’s instructions, respectively. After 30 min of staining, the cells were harvested using TrypLE Express and neutralized with completed media for DPCs and defined trypsin inhibitor for keratinocytes. The harvested DPCs were then filtered through a 15-μm strainer (SPL, Seoul, Korea). DPCs and keratinocytes were mixed together in a ratio of 3:1. Subsequently, the mixture containing 80,000 total cells of both types in 300 μl of DMEM maintenance medium was seeded into 1 well of a 96-well PAMCELL plate. The culture medium was replaced by removing 150 μl of the old medium and adding 150 μl of fresh Williams maintenance medium, consisting of Williams’ Medium E supplemented with 2 mM l-glutamine (100×), 10 ng/ml hydrocortisone, 10 μg/ml insulin, and 1% PS (5 ml), 24 h after seeding the cells. The cell movement during the first 30 h postseeding was recorded using Agilent BioTek Cytation C10 (YBioTech Inc., Gyeonggi, South Korea), capturing images every hour under conditions of 37 °C and 5% CO_2_ (Video S2, seconds 0 to 11). Detailed information on the composition of the culture medium is provided in Table S1. Fluorescent images of the same well in a 96-well plate on PAMCELL dishes from day 1 to day 6 were taken using Cell3iMager duos 2 CC-8300 (SCREEN Holdings Co., Ltd., Kyoto, Japan) (Video S2, seconds 11 to 20). The length of the stratum corneum was automatically measured through the green fluorescent signal from keratinocyte cells using the high-content screening system, Cell3iMager, with a slight modification in the region of interest set at 90%. The number of samples analyzed per well was 45 (*n* = 45/well).

### Cell viability

To assess the cell viability, spheroids were treated with Live/Dead Viability/Cytotoxicity Kit; 2 μM calcein AM, green fluorescent live cell stain, and 4 μM ethidium homodimer-1 were treated for 30 min in cell media at 37 °C. Samples were then washed, and fluorescent images were taken and analyzed via Cell3iMager duos 2 CC-8300 (SCREEN Holdings Co., Ltd., Kyoto, Japan).

### Hair growth-promoting substance treatments

The culture medium was replaced by removing 150 μl of old medium and adding 150 μl of fresh Williams maintenance medium at 24 h after seeding cells. Hair growth-promoting molecules were prepared and added to the culture medium at the concentrations listed in Table S2. Fluorescent images of the co-cultured cell complex were captured by HCS Cellimage3 before the medium was replaced with 50% new medium every 24 h. The monitoring process continued until the end of the sixth day of culture. All statistical analyses were performed in the Prism 8 software package (GraphPad Software), using the Kruskal–Wallis test with a post hoc test, one-way analysis of variance (ANOVA) with a post hoc test, or 2-way ANOVA with a post hoc test. All tests were 2-tailed, and *P* < 0.05 was considered statistically significant.

### Alkaline staining

DP spheroids were fixed in 4% paraformaldehyde (PFA) solution for 1 h at room temperature (RT). Samples were washed 3 times with PBS and then permeabilized with 0.3% Triton-X 100 for 30 min at RT. After washing 3 times in ALP buffer (100 mM NaCl, 100 mM Tris-Cl [pH 9.5], 50 mM MgCl_2_, 1% Tween 20), cells were stained in the same buffer in the presence of 4-nitro blue tetrazolium chloride and 5-bromo-4-chloro-3-indoyl phosphate for 20 min. Samples were washed by deionized water before further analysis.

### Flow cytometry

Spheroids were dissociated into single cells via Accutase at 37 °C for 10 min and fixed under 4% PFA for 20 min; 1% Triton-X 100 was used to permeabilize the cells at RT. Samples were blocked with EveryBlot Blocking (EBB) Buffer and then incubated with 6 different primary antibodies (anti-versican, anti-vascular endothelial growth factor [anti-VEGF], anti-platelet-derived growth factor receptor-alpha [anti-PDGFR-α], anti-platelet-derived growth factor receptor-beta [anti-PDGFR-β], anti-α-SMA, and anti-fibronectin; Table S3) for 2 h at RT. After being washed, samples were treated with corresponding secondary antibodies for 1 h at RT. The treated cells were analyzed using NovoCyte Flow Cytometer Systems (Agilent Technologies, Santa Clara, CA, USA).

### Immunocytochemistry

Co-culture spheroids were fixed with 4% PFA for 1 h at RT. After fixation and washing with PBS, spheroids were permeabilized with 0.3% Triton-X 100 for 30 min at RT. After spheroids were washed 3 times with PBS, they were then treated with EBB Buffer for 1 h at RT. Nine primary antibodies against versican, hair cortex cytokeratin (AE13), keratin 71, keratin 5, VEGF, PDGFR-α, PDGFR-β, α-SMA, and fibronectin (Table S3) were incubated overnight at 4 °C. After washing with EBB Buffer, secondary antibodies were incubated for 1 h at RT. The nuclei of the spheroids were stained with 4′,6-diamidino-2-phenylindole dihydrochloride (1:1,000 dilution in PBS) for 20 min. Fluorescent images were then captured using Leica Stellaris 5 (Leica Camera, Wetzlar, Germany).

### Microarray and RNA sequencing

Total RNA was extracted by TRIzol (Gibco-BRL, Grand Island, NY, USA). RNA sequencing and downstream data analysis were performed by E-Biogen Inc. (Seoul, Korea). The basic protocol was as follows: RNA quality was tested via Bioanalyzer 2100 System (Agilent Technologies, Amstelveen, Netherlands). Then, whole-RNA analysis was performed using Affymetrix GeneChip miRNA 4.0 arrays (Thermo Fisher Scientific) according to the manufacturer’s instructions. Briefly, 100 ng of total RNA was labeled and then hybridized for 18 h at 48 °C with an array, which was subsequently washed, stained, and read out with GeneChip Array Scanner 3000 7G (Applied Biosystems, Waltham, MA, USA). Acquired data were analyzed using the Transcriptome Analysis Console (TAC 4.0.1) software (Applied Biosystems). The differentially expressed gene master file was filtered via the Excel-based Differentially Expressed Gene Analysis (ExDEGA) GraphicPlus v2 software provided by E-Biogen Inc.

### Proteomic sample preparation

Samples were resuspended in lysis buffer containing 4% sodium deoxycholate, 4% sodium lauroyl sarcosinate, and 50 mM triethylammonium bicarbonate (Thermo Scientific) followed by incubation at 95 °C in 5 min. After that, samples were sonicated using a probe tip sonicator (Qsonica) on ice with a 30% amplitude, 5 s/5 s on/off cycle in 2 min. The protein lysates were then reduced and alkylated with 10 mM Tris(2-carboxyethyl)phosphine (Sigma) and 10 mM 2-chloracetamide final concentration at 95 °C in 5 min. Sample mixtures containing 100 μg of proteins were diluted 4 times using 50 mM triethylammonium bicarbonate prior to digestion with 2 μg of mix Trypsin/Lys-C (Promega) at 37 °C overnight. After protein digestion, sodium deoxycholate and sodium lauroyl sarcosinate were removed by phase transfer using ethyl acetate (Sigma). Tryptic peptides were desalted using Oasis HLB 1 cc Vac Cartridge (Waters) and dried using SpeedVAC. From each sample, 50 μg of peptides were labeled with TMTsixplex Isobaric Label Reagent (Thermo Scientific) following the manufacturer’s protocol. All labeled peptides were pulled together and fractionated using HPLC 1260 (Agilent) with mobile phase A composed of 10 mM ammonium formate pH 10.0 in water and mobile phase B composed of 10 mM ammonium formate pH 10.0 in 90% acetonitrile. A 95-min gradient was carried out on an XBridge Peptide BEH column (130 Å, 3.5 μm, 2.1 × 250 mm) (Waters) at a flow rate of 0.4 ml/min starting with stabilizing at 3% B in 3 min, then gradually increasing to 40% B in 80 min, and finishing up by ramping up to 90% B in 10 min and stabilizing in 3 min. A total of 95 fractions were further combined into 24 fractions and dried using SpeedVAC.

### Liquid chromatography–tandem mass spectrometry analysis

Approximately 2 μg of label peptides from each fraction was picked up and separated on an EASY-Spray PepMap Neo column (75 μm × 500 mm) (Thermo Scientific) using Vanquish Neo HPLC (Thermo Scientific) at a flow rate of 300 nl/min. The mobile phases A and B were 0.1% formic acid in water and 0.1% formic acid in acetonitrile, respectively. The liquid chromatography gradient was set as follows: 2 min of 4% B, 98 min of increasing to 20% B, 15 min of increasing to 40% B, 10 min of ramping up to 95% B, and 15 min of stabilizing at 95% B. Ionized peptides were analyzed by an Orbitrap Eclipse Tribrid mass spectrometer (Thermo Scientific) in TMT-SPS-MS3 mode. Precursor ions with a range of 400 to 1,600 *m*/*z* were analyzed at a resolution of 120,000 and a standard automatic gain control target. The top abundant precursor ions were selected for fragmentation using a collision-induced dissociation collision energy of 30% and then analyzed in a linear ion trap at a turbo scan rate, within a normal mass range, and with a standard automatic gain control target. Further 10 synchronous precursor selection precursor ions were extracted, fragmented at a higher-energy collision-induced dissociation collision energy of 55% and analyzed using Orbitrap at a resolution of 50,000.

### Database searching

Thermo raw files were first converted to the mzML file format using MSConvert [31]. Raw data were then searched again human proteome reference sequences from UniProt (version year 2023, 20,596 entries) using MSFragger v3.8 on the FragPipe v 20.0 interface with philosopher v5.0.0 and EasyPQP v0.1.42 [32–34]. The parameters for peptides were set with lengths from 7 to 50 and mass ranges from 200 to 5,000. Variable modifications contained methionine oxidation, protein N-terminal acetylation, and peptide N-terminal tandem mass tag labeling. The fixed modifications contained cysteine carbamidomethylation and lysine tandem mass tag labeling. MSBooster for rescoring was enabled [35]. Peptide spectrum match validation was done using Percolator with a minimum probability of 0.5. The peptide spectrum match output table was subjected to further analysis using MSStatsTMT v2.10.0 [36]. The protein summarization method was set as Tukey’s median polish. Normalization was done at both peptide and protein levels.

### Scanning electron microscopy

The details of the PAMCELL plate substrate were examined using a scanning electron microscope (JSM-7900F, JEOL Ltd., Seoul, South Korea). Scanning electron microscopy preparation of spheroids was carried out through the cell silicification method [37]. Briefly, spheroids were fixed in 4% PFA for 1 h and triple washed with distilled water. After immersing the spheroids in a solution of 100 mM tetramethyl orthosilicate in 1 mM hydrochloric acid at 40 °C for a minimum of 48 h, a sequential ethanol dehydration process was implemented at low to absolute concentration at 10-min intervals. The DP spheroids’ surface was then coated with platinum before scanning electron microscopy imaging.

### Statistical and bioinformatics analysis

The quantification values of the identified proteins were normalized by taking the fraction of the total, followed by multiplication by 106 and log2 transformation. All statistical analyses were performed using GraphPad Prism version 9.0.0 (GraphPad, CA, USA). Data are shown as mean ± standard error of the mean (SEM). The Student *t* test and one-way ANOVA were applied, and *P* values of *P* ≤ 0.05, 0.01, 0.001, and 0.0001 were considered significant. The proteins were annotated for biological processes and cellular components using the online tool DAVID (https://david.ncifcrf.gov/) [38], employing Gene Ontology analysis [39]. The Matrisome database (http://matrisomeproject.mit.edu/proteins/) was used to annotate the ECM and ECM-associated proteins [40,41]. The heatmap of protein quantitation values was displayed using the Perseus software (version 2.0.11) [42].

## Results and Discussion

### Design and characterization of a radial-patterned plate for DP spheroid formation

To promote efficient spheroid formation through dynamic cellular interactions, we developed a radial-patterned culture plate, termed PAMCELL (Fig. S1) [43]. The core functionality of PAMCELL lies in its radial design, which facilitates the formation of spheroids by guiding cell migration. The plate’s surface is coated with 700-nm silica particles bound to RGD peptides, creating weak integrin interactions on the cell membranes that encourage initial cell adhesion and migration. The radial arrangement of the peptides directs cells toward the center of each pattern. Each radial pattern consists of 48 lines, each 3 μm thick, which channel the cells into central aggregation, forming compact spheroids (Fig. S2). The 200-μm spacing among the patterns prevents spheroid fusion and allows for the development of a surrounding stratum corneum, maintaining spheroid structure and preventing cross-contamination among neighboring regions.

**Fig. 1.**
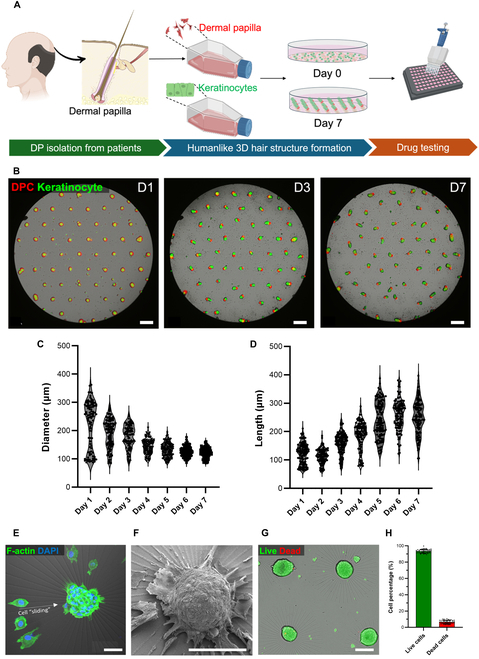
Three-dimensional co-culture system for dermal papilla (DP) spheroid formation. (A) Schematic illustration of the experimental design used in this study, outlining the key steps from cell isolation to spheroid formation. (B) Time-lapse images showing the formation and elongation of the hair-peg-like structure in the DP spheroids from day 1 (D1) to day 7 (D7). Scale bars indicate 500 μm. (C) Size distribution of DP spheroids from D1 to D7 (*n* = 100). (D) Keratinocytes’ outgrowth length from D1 to D7 (*n* = 100). (E) Spheroid formation through dynamic cell sliding toward the center on the radial pattern, facilitating compact spheroid formation. (F) Scanning electron microscopy image of a DP spheroid after 3 d of culture on a radial pattern. (G) Live/Dead assay of DP spheroids 2 d after spheroid formation. Scale bars indicate 100 μm. (H) Percentage of live and dead cells inside spheroids after 2-d formation (*n* = 50). 3D, 3-dimensional; DAPI, 4′,6-diamidino-2-phenylindole dihydrochloride.

This innovative design enabled the rapid and efficient formation of DPCs with keratinocyte spheroids (DP spheroids), as it provided precise control over cell positioning and movement. Human DPCs isolated from scalp HFs aggregated into compact spheroids within 24 h (Fig. 1A and Video S1). The rapid formation of these spheroids is crucial for maintaining their biological functions, particularly in regenerative medicine applications.

By day 3, the spheroids remained compact, with significant cellular polarization and migration toward the periphery, forming a tissue architecture reminiscent of in vivo HFs (Fig. 1B and Video S2). The size of DP spheroids was opposite of the outgrowth length of the keratinocytes; while DP spheroids decreased in area and formed a compact niche, the keratinocytes with nutrition supported from DP grew day by day. On day 1, the average diameter of DP spheroids was 221.08 ± 82.69 μm, and the average length of keratinocytes was 125.47 ± 37.97 μm. On the other hand, on day 7, the average diameter of DP spheroids was 120.69 ± 17.37 μm and the average length of keratinocytes was 257.21 ± 55.56 μm (Fig. 1C and D and Table S4). By day 7, this organization led to the elongation of keratinocytes into structures resembling early-stage hair pegs, a crucial feature in HF regeneration.

The 200-μm spacing between patterns was essential for preserving biochemical gradients and microenvironmental cues within each spheroid. Scanning electron microscopy analysis confirmed that the radial pattern design successfully supported the formation of well-defined cellular structures, crucial for reproducibility and scalability in tissue modeling for biomedical applications (Fig. 1F). The DP spheroids ranged in diameter from 150 to 180 μm, and Live/Dead assays on 50 spheroids confirmed that 93.42% ± 2.23% of cells were alive and only 6.58% ± 2.23% of cells were nonviable (Fig. 1G and H and Table S5). The results demonstrated that the radial pattern design provided the necessary mechanical cues to support cell viability and overall health.

In summary, the radial-patterned PAMCELL plate effectively promotes the formation of homogeneous and viable 3D DP spheroids. Additionally, it enhances the polarization of DP–keratinocyte mixtures, allowing for systematic investigation of complex tissue interactions under controlled conditions. Our findings align with previous studies demonstrating the advantages of 3D culture systems over 2D cultures for DPCs. The rapid formation of DP spheroids on the PAMCELL platform preserves the hair-inductive properties of DPCs, as they maintain higher expression levels of key markers such as ALP, versican, and α-SMA. This is consistent with previous reports showing the enhanced stemness and long-term expansion capacity of human HFDPCs in 3D spheroid cultures compared to those in 2D cultures [12,13]. The improved functional properties observed in our study further support the notion that 3D culture environments better mimic the in vivo conditions necessary for HF regeneration.

### Functional properties of DP spheroids on a PAMCELL plate

**Fig. 2.**
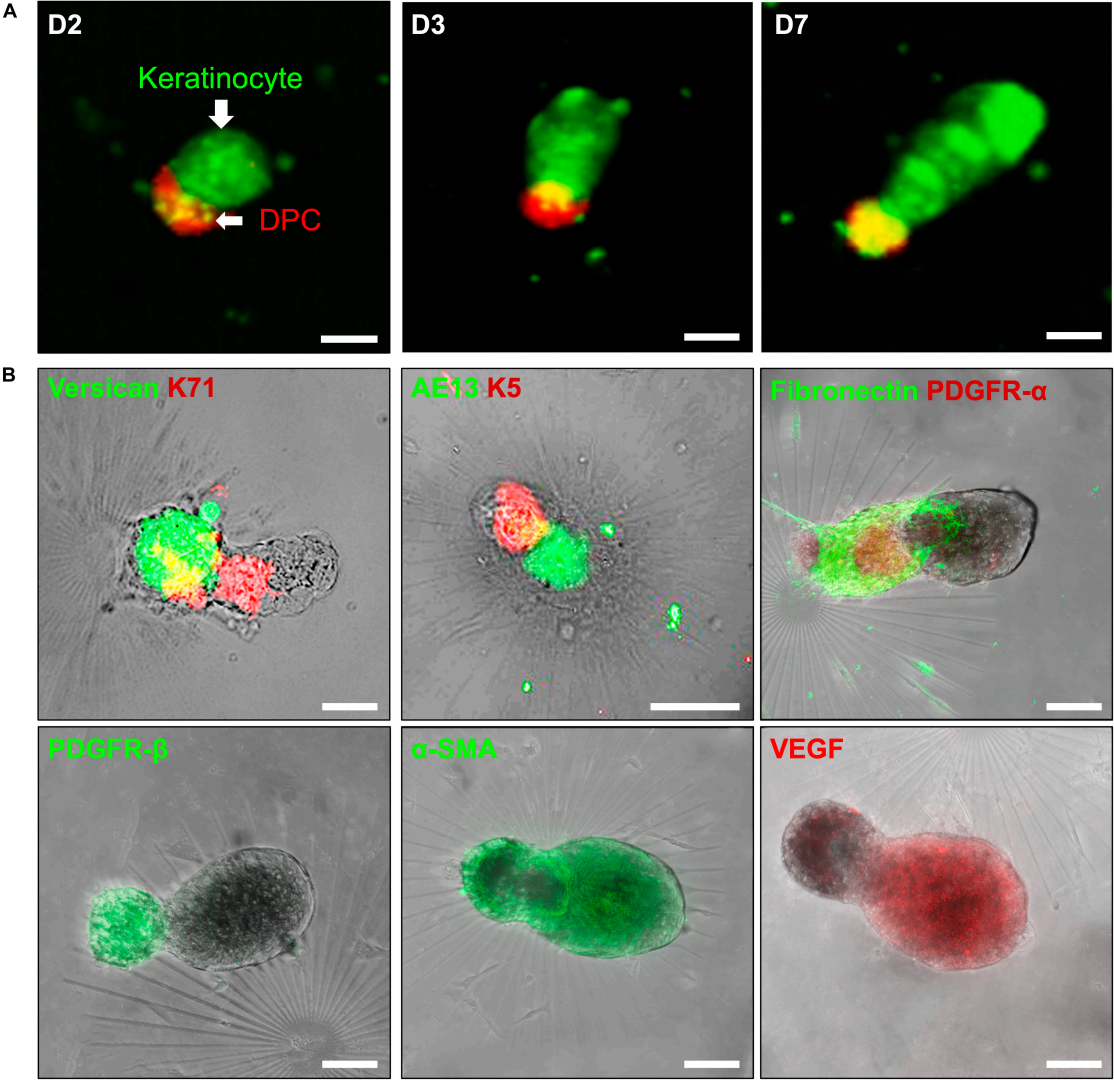
The functional protein expression of DP spheroids on a PAMCELL plate. (A) Changes in the length of the hair-peg-like structure in DP spheroids over days 2, 3, and 7. (B) The expressions of mesenchyme-associated markers (versican, platelet-derived growth factor receptor-alpha [PDGFR-α], and platelet-derived growth factor receptor-beta [PDGFR-β]), hair cortex cell marker (AE13), fibronectin, and vascular endothelial growth factor (VEGF) in DP spheroids at day 6. Scale bars indicate 100 μm. DPC, primary dermal papilla cell; K71, keratin 71; K5, keratin 5; α-SMA, α smooth muscle actin.

We next evaluated the structure and gene expression profiles of DP spheroids formed on the PAMCELL plate using DP cells with passage numbers less than 5, after 3 d of culture. ALP activity, a marker of DP functionality, was observed throughout the spheroids, with the highest concentration in the central region (Fig. S2). During the formation of DP spheroids, keratinocytes were initially concentrated in the spheroid’s center while DPCs surrounded them (Fig. 2A). Time-lapse imaging (Video S2) revealed that, over time, the keratinocyte layer migrated toward one pole, gradually elongating as the culture period progressed. By days 6 to 8, the length of the elongated keratinocytes reached a maximum of approximately 200 μm (Video S2).

Within 6 d of culture, DP spheroids expressed several mesenchyme-associated markers, including versican, PDGFR-α, and PDGFR-β (Fig. 2B). The expression of fibronectin, a component of the ECM characteristic of growing anagen HFs, was also detected, specifically within the germ region of the DP spheroids. Furthermore, the hair cortex marker AE13 was strongly expressed in the outgrowing structures. The formation of tissue-specific structures, such as the inner root sheath, confirmed the development of the cornified layer that envelops the hair shaft and forms the channel for the growing hair. VEGF expression was detected in both cultured DP cells and keratinocytes, further supporting the functional properties of the spheroids.

Overall, the PAMCELL platform supports not only the rapid formation of DP spheroids but also the maintenance of their functional properties, allowing for detailed investigations into HF biology and providing a promising tool for regenerative medicine applications.

### Comparative analysis of PAMCELL and conventional DP spheroid formation methods

**Fig. 3.**
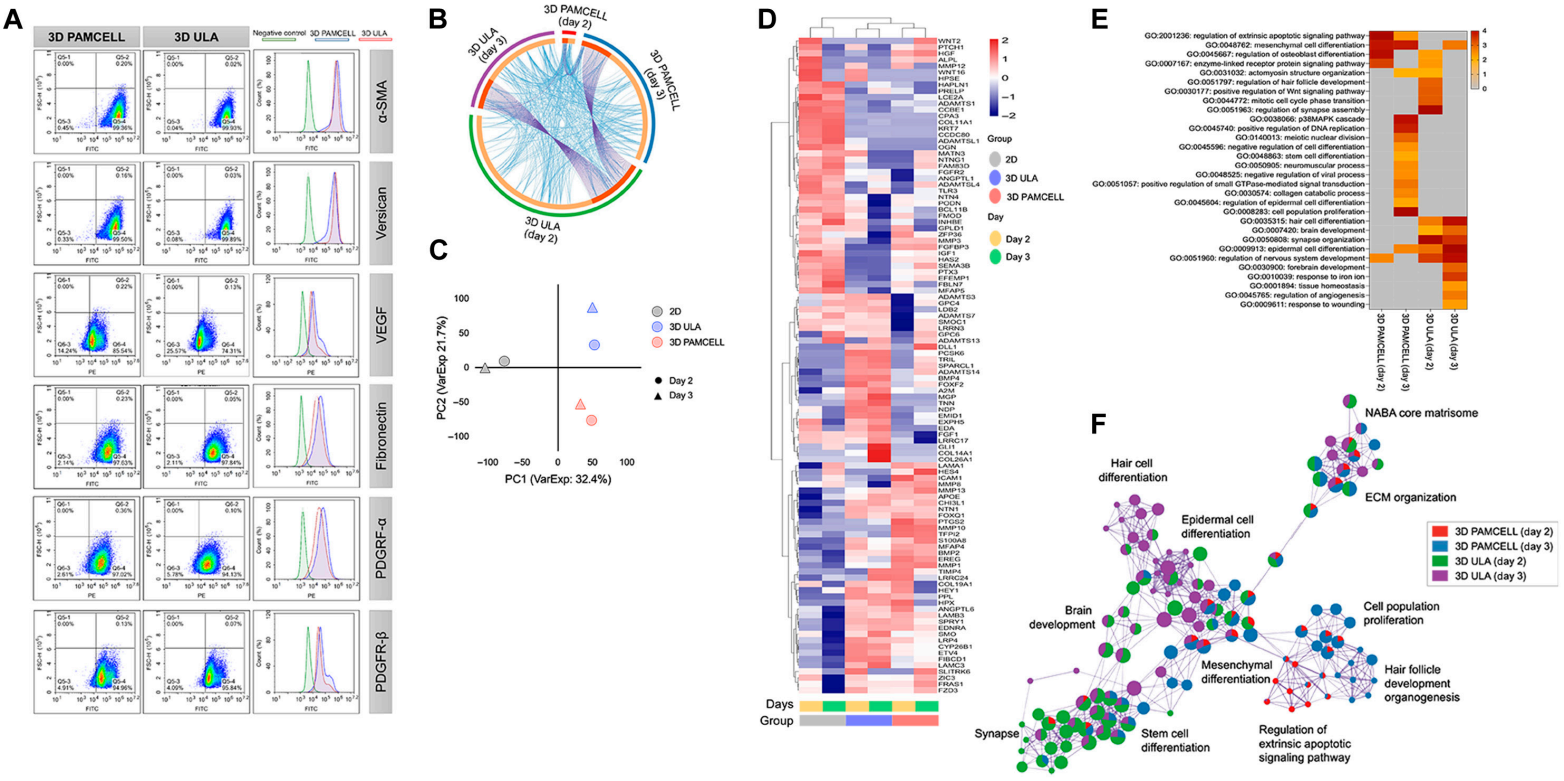
Comparative analysis of PAMCELL and ultralow attachment (ULA) methods for DP spheroid formation. (A) Flow cytometry analysis of DP spheroids cultured on PAMCELL and ULA plates showing expression levels of α-SMA, versican, VEGF, fibronectin, PDGFR-α, and PDGFR-β. (B) Circos plot illustrating gene expression profiles and their correlation between 2-dimensional (2D) and 3D culture platforms. (C) Principal component analysis (PCA) of gene expression profiles showing distinct clustering based on culture conditions. (D) Heatmap of differentially expressed genes in DP spheroids cultured on PAMCELL and ULA plates over days 2 and 3. (E) Gene Ontology (GO) analysis highlighting biological processes enriched in DP spheroids cultured in 3D environments. (F) Network map showing biological processes and pathways significantly enriched in 3D cultured DP spheroids, including extracellular matrix (ECM) organization and hair follicle development. FITC, fluorescein isothiocyanate; PE, phycoerythrin; FSC-H, forward scatter height; VarExp, variance explained; p38MAPK, p38 mitogen-activated protein kinases.

To assess the advantages of the PAMCELL plate over the widely used ULA plates for DP spheroid formation, we conducted a comparative analysis. Flow cytometry revealed no significant differences in mesenchyme-associated protein expression between spheroids formed using PAMCELL and ULA plates (Fig. 3A). However, messenger RNA (mRNA) microarray analysis showed that although fewer than 10% of genes were commonly expressed in DP spheroids cultured in both 3D ULA and PAMCELL conditions, the genetic correlation between the 2 methods was high (Fig. 3B). Principal component analysis further demonstrated distinct clustering between 2D, 3D ULA, and 3D PAMCELL groups based on PC1 and PC2, indicating that the gene expression profiles were more influenced by the culture method than the duration of cultivation (Fig. 3C). This highlights the remarkable impact of the culture environment on DP spheroid gene expression.

In comparison to 2D cultures, DP spheroids formed in 3D environments exhibited increased expression of genes related to ECM organization, hair cycle regulation, keratinocyte differentiation, EMI, and epidermal differentiation (Fig. 3D). These findings suggest that 3D culturing allows DP spheroids to more closely mimic the in vivo state. Notably, DP spheroids cultured on PAMCELL plates formed and polarized more rapidly than those on ULA plates, leading to elevated expression of genes associated with ECM organization, cell proliferation, HF development, and mesenchymal differentiation (Fig. 3E and F). The radial pattern design of PAMCELL facilitated initial cell adhesion and guided migration, which contributed to the efficient formation of spheroids. This superior performance in promoting ECM organization and gene expression highlights PAMCELL’s ability to create a more in vivo-like environment, making it an optimal platform for DP spheroid formation.

Our results are consistent with findings from Liu et al. [44], who highlighted that 3D culture systems enhance cell viability and promote the expression of hair-inductive genes. The enhanced ECM production and up-regulated gene expression related to HF development in PAMCELL-derived spheroids underscore the importance of adopting advanced 3D culture techniques for hair regeneration therapies. These results further validate the efficacy of the PAMCELL platform in providing a more favorable microenvironment compared to conventional ULA plates.

### Enhanced ECM protein expression in DP spheroids on PAMCELL plates

**Fig. 4.**
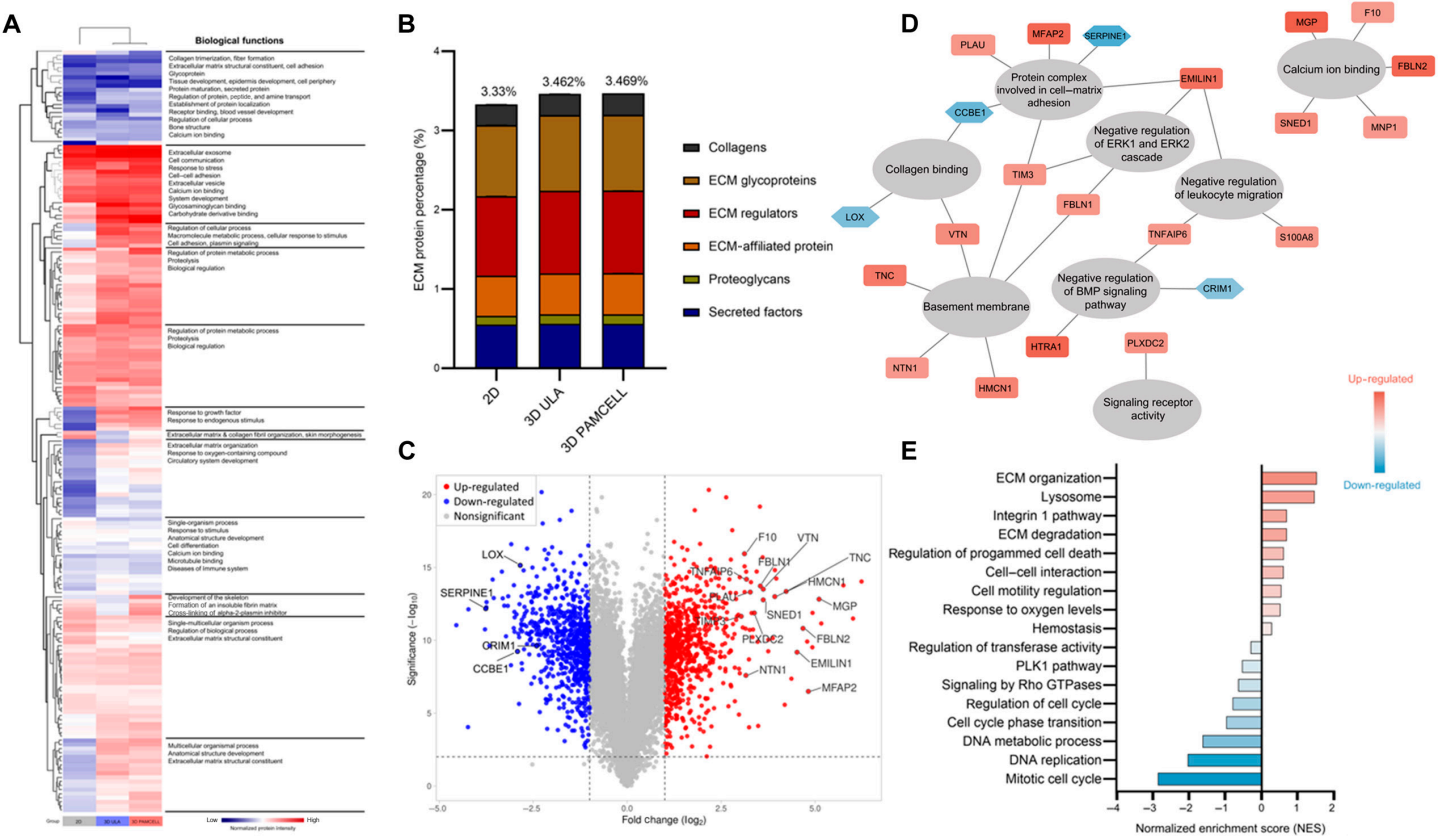
Proteomics for comparative analysis of PAMCELL and ULA culture methods. (A) Heatmap showing the differential expression of biological functions in DP spheroids across 2D, 3D ULA, and 3D PAMCELL culture platforms. (B) The percentage distribution of ECM protein components across the 3 culture conditions. (C) Volcano plot illustrating the up-regulated and down-regulated proteins in DP spheroids cultured on 3D PAMCELL compared to 2D culture. (D) Network diagram of significantly up-regulated and down-regulated ECM-related proteins in DP spheroids cultured on 3D PAMCELL compared to that for 2D culture. (E) Normalized enrichment score (NES) bar chart indicating significant pathways and biological processes enriched in DP spheroids cultured on 3D PAMCELL compared to that for 2D culture. ERK1, extracellular signal-regulated kinase 1; ERK2, extracellular signal-regulated kinase 2; BMP, bone morphogenetic protein; PLK-1, polo-like kinase 1.

Proteomic analysis identified 1,472 of 9,173 proteins as differentially expressed between 2D and 3D culture platforms (Fig. 4A and Tables S6 and S7). Among these, 319 proteins were classified as ECM related, comprising 3.48% of the total protein count. ECM-related proteins accounted for 3.33%, 3.46%, and 3.47% of the total protein abundance in 2D, 3D ULA, and 3D PAMCELL samples, respectively (Fig. 4B). Of the differentially expressed ECM-related proteins with a |log_2_FC| > 2.5, 18 were up-regulated in the 3D PAMCELL group compared to those in the 2D group, including 6 foundational components of the cellular membrane and 5 calcium-binding proteins on the cell surface (Fig. 4C). These findings align with previous analyses indicating that collagen-binding proteins, which play a role in cell proliferation, are up-regulated in 3D cultures (Fig. 4D).

One particularly notable result was the significant down-regulation of SERPINE1 (log_2_FC = −3.75), a protein involved in cell–matrix adhesion and known to stimulate keratinocyte migration. The up-regulation of pathways related to ECM organization, ECM degradation, integrin 1 signaling, cell–cell interactions, and cell motility regulation in the 3D PAMCELL platform underscores the complexity of the 3D microenvironment (Fig. 4E). In contrast, proteins involved in cell cycle processes such as mitosis, DNA replication, and phase transitions were down-regulated in the 3D PAMCELL group compared to those in the 2D group.

Overall, PAMCELL plates enhanced the expression of ECM-related proteins, which are crucial for tissue engineering and regenerative medicine applications. The increased expression of genes related to ECM organization, cell adhesion, and responses to growth factors in the 3D cultures emphasizes the importance of a supportive microenvironment for promoting cellular functions that closely resemble in vivo conditions. The differential expression of ECM-related proteins between 2D and 3D platforms, particularly the down-regulation of collagen-binding proteins and SERPINE1, suggests a nuanced interaction between cell–matrix adhesion and cellular signaling pathways. Additionally, the down-regulation of cell-cycle-related proteins in 3D cultures may indicate a shift toward a more differentiated state, enhancing the functional characteristics of DP spheroids.

### Comparative mRNA microarray analysis shows superior ECM production and cellular functionality

**Fig. 5.**
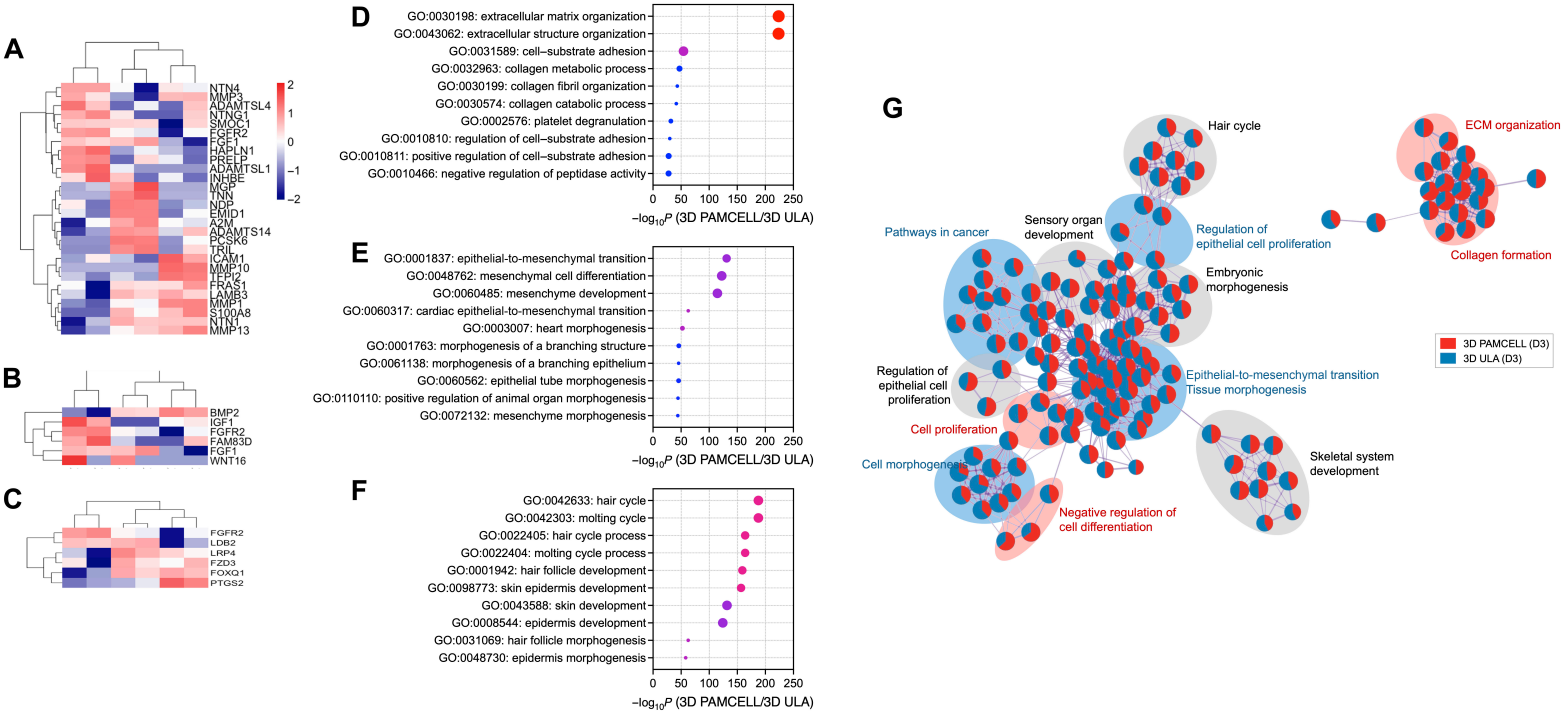
Hair cycle enrichment in messenger RNA (mRNA) profiles between PAMCELL and ULA methods. (A to C) Heatmaps displaying differential gene expression related to (A) the ECM, (B) epithelial–mesenchymal interactions (EMIs), and (C) the hair cycle in DP spheroids cultured on PAMCELL and ULA plates. (D to F) Gene expression enrichment analyses involved in (D) ECM, (E) EMI, and (F) hair cycle in DP spheroids cultured on PAMCELL and ULA plates. (G) Network map depicting the enriched biological processes and pathways in DP spheroids cultured on PAMCELL plates.

Both proteomic and transcriptomic analyses revealed that DP spheroids cultured on PAMCELL plates produced significantly more ECM-related proteins associated with cell adhesion and functionality compared to those in 2D cultures. This suggests that PAMCELL spheroids more closely resemble in vivo conditions. To further investigate the differences between DP spheroids generated on PAMCELL plates and those on conventional ULA plates, we conducted mRNA microarray analysis (Fig. 5).

The mRNA microarray analysis demonstrated significant differences in gene expression profiles between DP spheroids cultured on PAMCELL and ULA plates, particularly in genes related to ECM organization, EMI, and the hair growth cycle (Fig. 5A to C). DP spheroids on PAMCELL plates exhibited elevated expression of genes involved in ECM matrix organization and cell–substrate adhesion (Fig. 5D), which likely supports better structural integrity and functionality of the spheroids. Additionally, increased expression of genes related to DP cell differentiation (Fig. 5E) suggests that the microenvironment provided by PAMCELL plates promotes more effective cellular differentiation.

Moreover, genes associated with cell proliferation, the hair growth cycle, and molting were more highly expressed in DP spheroids cultured on PAMCELL plates (Fig. 5F), indicating enhanced biological processes. The network map (Fig. 5G) highlights enriched biological pathways, particularly those related to ECM organization, collagen formation, and epithelial-to-mesenchymal transition. These results suggest that PAMCELL plates create a more conducive environment for DP spheroid formation, closely mimicking in vivo conditions and enhancing overall cellular functionality.

### Evaluation of nutrients and growth factors on DP spheroid performance

To assess the functionality of DP spheroids cultured on PAMCELL plates, we evaluated the impact of various drugs and nutrients on hair peg length, in a concentration-dependent manner. PAMCELL plates, with their radial pattern design and capacity to generate approximately 60 spheroids per well, offer a powerful platform for high-throughput drug testing (Fig. S1).

**Fig. 6.**
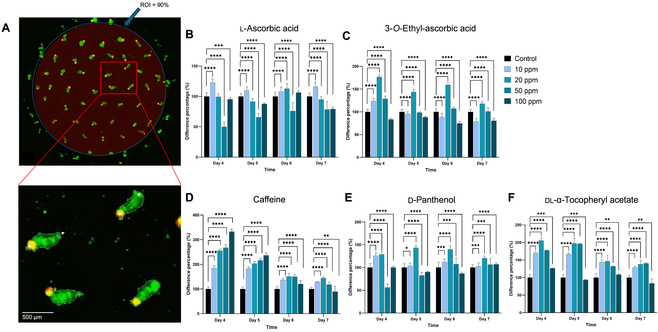
Concentration-dependent effects of nutrients and growth factors on DP spheroid performance on a PAMCELL plate. (A) Representative image of high-content screening (HCS) for hair growth analysis. (B to F) Concentration-dependent effects of (B) l-ascorbic acid, (C) 3-*O*-ethyl ascorbic acid, (D) caffeine, (E) d-panthenol, and (F) dl-α-tocopheryl acetate on hair peg length. Relative length of stratum corneum compared to that of control samples over 7 d (Student *t* test, *n* = 5, *P ≤ 0.05, **P ≤ 0.01, ***P ≤ 0.001, ****P ≤ 0.0001). ROI, region of interest.

The hair growth-promoting efficacy of l-ascorbic acid, 3-*O*-ethyl ascorbic acid, dl-α-tocopheryl acetate, d-panthenol, and caffeine was tested at concentrations of 10, 20, 50, and 100 ppm by measuring the relative length of the stratum corneum compared to that of control samples from day 4 to day 7 (Fig. 6 and Tables S2 and S8). All 5 substances showed positive effects on keratinocyte proliferation, as indicated by an increased stratum corneum length.

l-ascorbic acid was most effective at 20 ppm, producing a 177% increase over the control. 3-*O*-Ethyl ascorbic acid showed optimal efficacy at 10 ppm, enhancing the stratum corneum length by 123% compared to the control. dl-α-Tocopheryl acetate exhibited its highest efficacy at 20 ppm, with a 129% increase. d-Panthenol showed a significant 206% increase at 20 ppm, and caffeine demonstrated strong effectiveness across all concentrations, with a peak of 333% efficacy at 100 ppm. While l-ascorbic acid and 3-*O*-ethyl ascorbic acid exhibited toxicity at 100 ppm, the other substances showed no toxicity at this concentration.

Interestingly, although all treatments showed positive effects by day 4, measurements taken on days 5, 6, and 7 indicated a slight decline in elongation, likely due to the natural curvature of the spheroids over time rather than continued elongation along the axis (Fig. S1). This suggests that day 4 represents the optimal time point for assessing treatment effectiveness.

These findings highlight the concentration-dependent responses of each substance and provide valuable insights for optimizing conditions to maximize efficacy. Additionally, the results confirm the functional capability of PAMCELL DP spheroids in supporting keratinocyte proliferation and nutrient transfer, further validating the utility of the PAMCELL platform in simulating in vivo-like conditions.

### In vivo validation of hair growth induction by PAMCELL-derived spheroids

**Fig. 7.**
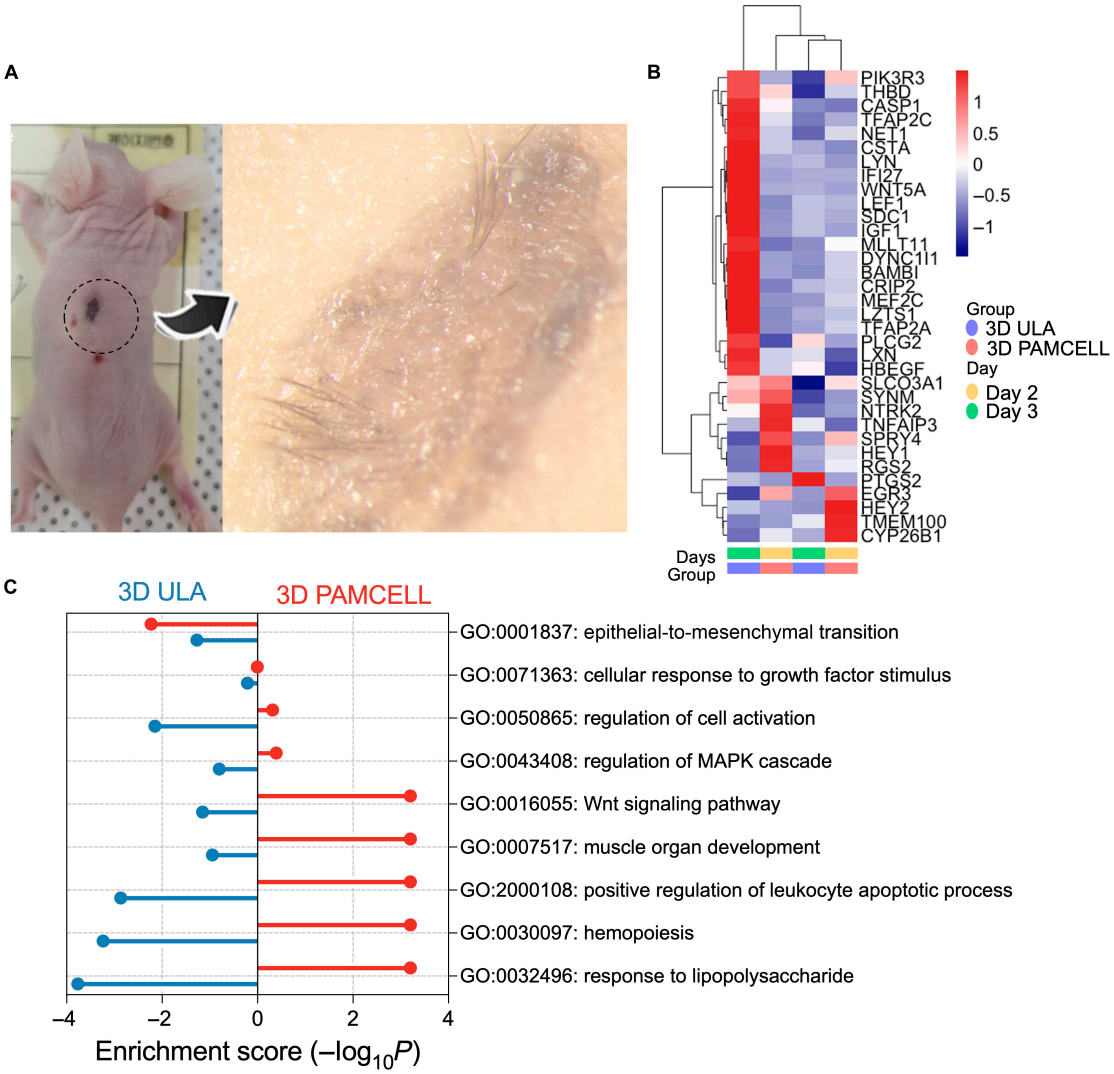
In vivo hair growth after PAMCELL DP spheroid transplantation. (A) Transplantation of DP spheroids into nude mice and subsequent hair growth. (B) Heatmap of in vivo-related gene expression in DP spheroids from ULA and PAMCELL plates. (C) GO term enrichment analysis showing differential pathways in DP spheroids from PAMCELL plates.

Lastly, to validate the hair growth potential of DP spheroids cultured on PAMCELL plates, we conducted transplantation experiments in animal models (Fig. 7A). Nude mice, which are genetically hairless, were used to assess the spheroids’ ability to induce hair growth. The results demonstrated that DP spheroids derived from PAMCELL plates successfully promoted hair growth after transplantation. This enhanced hair growth is likely attributed to the improved ECM production, along with increased regulation of key processes such as hair differentiation and cell proliferation.

To further investigate the underlying mechanisms, we compared the in vivo gene expression profiles of DP spheroids generated on PAMCELL plates with those formed on ULA plates. The analysis revealed significant differences between the 2 groups (Fig. 7B), with distinct Gene Ontology term enrichments (Fig. 7C). Notably, DP spheroids from PAMCELL plates showed enhanced expression of genes involved in the Wnt signaling pathway, muscle organ development, positive regulation of leukocyte apoptosis, hemopoiesis, and responses to lipopolysaccharides. These pathways are critical for the development of more mature and functional HFs.

These findings indicate that the PAMCELL platform not only facilitates rapid and efficient DP spheroid formation but also enhances the functional properties of the spheroids, making them more effective in promoting hair growth in vivo. The improved ECM production and the up-regulation of key signaling pathways contribute to the superior performance of PAMCELL-derived spheroids in HF regeneration.

## Conclusion

In this study, we introduced PAMCELL, a 3D culture system designed to enhance the assessment of potential hair loss treatments by closely replicating in vivo ECM dynamics. Our results demonstrated that the PAMCELL platform notably outperforms conventional methods, such as ULA plates, in promoting the formation, polarization, and functionality of DP spheroids.

The ability of PAMCELL to foster rapid spheroid formation, combined with enhanced ECM production and up-regulation of genes related to HF development, highlights its superiority in creating a physiologically relevant microenvironment. Proteomic and transcriptomic analyses revealed key differences between 2D and 3D culture platforms, with the PAMCELL system driving higher expression of genes involved in ECM organization, cell adhesion, and hair cycle regulation. These findings underscore the critical importance of the 3D microenvironment in maintaining cellular functions that more closely mimic in vivo conditions. The differential expression of ECM-related proteins and genes, particularly those involved in collagen formation and epithelial-to-mesenchymal transition, further emphasizes the role of substrate adhesion and ECM integrity in tissue morphogenesis and cellular differentiation.

Additionally, we evaluated the effects of various nutrients and growth factors on DP spheroid performance, with l-ascorbic acid, caffeine, and d-panthenol demonstrating strong positive effects on keratinocyte proliferation and hair peg formation. dl-α-Tocopheryl acetate also showed promising results, while 3-*O*-ethyl ascorbic acid displayed more limited effects and potential cytotoxicity at higher concentrations. These results suggest that PAMCELL is highly effective in screening compounds for hair growth-promoting properties, offering a valuable tool for optimizing hair loss treatments.

In conclusion, the PAMCELL 3D culture system provides a robust platform for the high-throughput screening of HF-like spheroids and enables efficient, high-throughput testing of hair growth-promoting compounds. By closely mimicking in vivo ECM dynamics, PAMCELL offers a comprehensive method for studying complex tissue interactions and validating therapeutic candidates, making it a promising tool for advancing regenerative medicine and hair loss treatment research.

## Data Availability

The mass spectrometry proteomics data have been deposited to the ProteomeXchange Consortium via the PRIDE partner repository with the dataset identifier PXD051461. All proteomic and transcriptomic data are attached as Supplementary Materials. Other data that support the findings of this study are available from the corresponding authors upon reasonable request.

## Supplementary Materials

Supplementary 1 Figs. S1 and S2 Tables S1 to S8 Videos S1 and S2
