# Supplementary material for: High-Throughput Screening of 3-Dimensional Co-culture Hair Follicle Mimetic Tissue with an Enhanced Extracellular Matrix for the Screening of Hair Growth-Promoting Compounds
Source: Biomater Res. 2024 Dec 27;28:0125. doi: 10.34133/bmr.0125 (PMC11675628; doi:10.34133/bmr.0125)
Supplement: Supplementary 1 — Figs. S1 and S2 Tables S1 to S8 Videos S1 and S2 [file bmr.0125.f1.zip › Supplementary Materials_revised.docx]

# Supporting Information

High-throughput screening of 3 dimensional co-culture hair follicle mimetic tissue with enhanced extracellular matrix for the screening of hair growth promoting compounds

Huyen T. M. Phama, Hyo-Sop Kima, Duc Long Nguyena, Hyun Woo Joob, Min Kyu Kimb, Young Kwan Sungb, Minh Hung Vuc, d, Heung Sik Hahmc, Woo Jung Kime, Jae-Ho Kima, *, Hyun-Ji Parka, *

a Department of Molecular Science and Technology, Ajou University, Suwon 16499, South Korea

b Department of Immunology, School of Medicine, Kyungpook National University, Daegu 41944, South Korea

c Uppthera, Incheon 21988, South Korea

d Center for Gene and Protein Research, Hanoi Medical University, Hanoi 100000, Vietnam

e Ellead Co. Ltd. Skin Bio Research, Seongnam 13590, South Korea*Corresponding author.

E-mail address: [hyunjipark@ajou.ac.kr](mailto:hyunjipark@ajou.ac.kr) (H-J Park), [jhkim@ajou.ac.kr](mailto:jhkim@ajou.ac.kr) (J-H Kim)


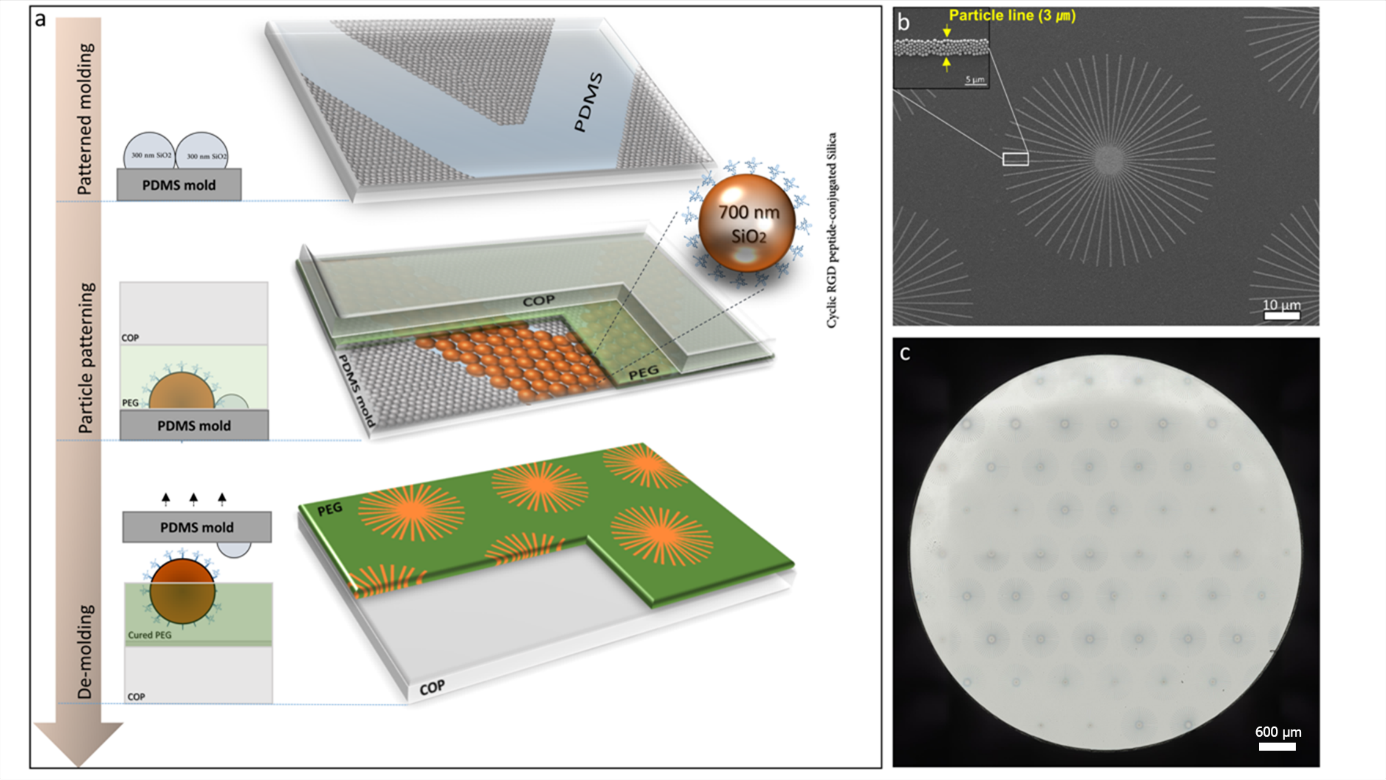


**Supplementary Figure S1**. Schematic diagram of 3D culture PAMCELL plate preparation and the substrate’s microstructure. (A) Process of arraying pattern on the substrate. **(B)** SEM image of the pattern structure on the culture plate and the dimensions of the pattern lines. **(C)** Bright-field image of a well in a 96-well plate, overall view of the patterns within a well.


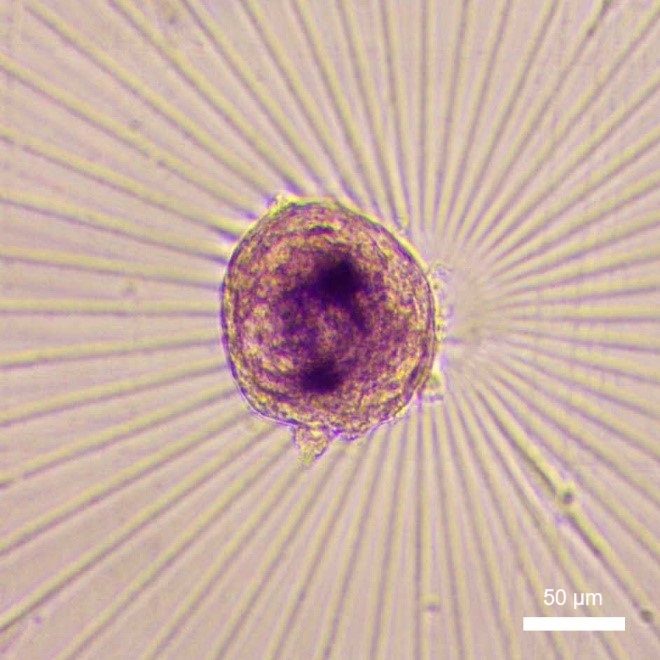


# **Supplementary Figure S2**. Staining images of the DP spheroid. BCIP/NBT indicated for Alkaline phosphatase activity.

# **Supplementary Table S1. Medium components and other consumables.**

| **Component** | **Cat. No.** | **Volume for 500 mL** | **Dilution** |
| --- | --- | --- | --- |
| **DMEM maintenance medium (DMD)** |  |  |  |
| DMEM, low glucose, pyruvate | #11885084; Gibco | 445 mL |  |
| Fetal Bovine Serum (FBS) | #16000044; Gibco | 50 mL | 1:10 |
| Penicillin-Streptomycin (PS) | #15140122; Gibco | 5 mL | 1:100 |
| hBFGF 4 ng/mL | #F0291-25UG; Sigma Aldrich | 20 µL | 1:25000 |
| **Williams maintenance medium (WMD)** |  |  |  |
| william E pure media | #A1217601; Gibco | 490 mL |  |
| 2mM L-glutamin (100X) | #25030149; Gibco | 5 mL | 1:100 |
| 10ng/mL hydrocortison | #H6909-10ML; Sigma Aldrich | 100 µL | 1:5000 |
| 10ug/mL insulin | #11070-73-8; Sigma Aldrich | 500 µL | 1:1000 |
| 1% PS | #15140122; Gibco | 5 mL | 1:100 |
| **Buffers and other reagents** |  |  |  |
| cyclic-RGD (Arg-Gly-Asp)  peptides | Peptron |  |  |
| Polyethylene glycol (PEG) | #25322-68-3; Sigma Aldrich |  |  |
| Sylgard 184 | #1023993; Dow Corning |  |  |
| Defined Trypsin Inhibitor (DTI) | #R-007-100; Gibco |  |  |
| Heat-inactivated fetal bovine serum (hi-FBS) | #16140071; Gibco |  |  |
| Tryple Express | #12605010; Gibco |  |  |
| 0.25% trypsin/10 mM EDTA | #25200056; Gibco |  |  |
| Keratinocyte Growth Kit | #PCS-200-040; ATCC |  |  |
| Dermal Cell Basal Medium | #PCS-200-030; ATCC |  |  |
| iMatrix-511 Silk (0.5 mg/ml solution) | #FNK-892021; NIPPI |  | 3:500 in PBS 1X |
| Live/dead™ Viability/Cytotoxicity Kit | # L3224; Invitrogen |  |  |
| StemPro Accutase Cell Dissociation Reagent | #A11105-01; Gibco |  |  |
| Formaldehyde solution 4%, buffered | # P2031; Biosesang |  |  |
| Hydrochloric Acid | #7647-01-0; Sigma Aldrich |  |  |
| Tetramethyl orthosilicate | #341436; Sigma Aldrich |  |  |
| Triton X-100 | #9036-19-5; Sigma Aldrich |  |  |
| NBT/BCIP Stock Solution | #11681451001; Roche |  | 1 ug/mL in no FBS DMD |
| CellTracker™ CM-DiI Dye | #C7000; Invitrogen |  | 1 ug/mL in no FBS DMD |
| CellTracker™ Green CMFDA | #C2925; Invitrogen |  |  |
| 4′,6-Diamidino-2-phenylindole dihydrochloride | #D1306; Invitrogen |  | 1:1000 in PBS 1X |
| Alexa Fluor 488 Phalloidin | #A12379; Invitrogen |  | 1:500 in PBS 1X |
| PBS, pH 7.4 | #10010023; Gibco |  |  |
| EveryBlot Blocking Buffer | #12010020; Bio-Rad |  |  |

# **Supplementary Table S2. Hair growth-promoting molecules treatments.**

|  | **Hair growth-promoting molecules** | **Common names** | **Solvent** | **Cat. No** | **Treatment concentration** |
| --- | --- | --- | --- | --- | --- |
| 1 | L-Ascorbic acid | Vitamin C | Water | #A4403, Sigma Aldrich | 10, 20, 50, 100 ppm |
| 2 | 3-O-ethyl ascorbic acid | Vitamin C | Water | #CDS009203, Sigma Aldrich | 10, 20, 50, 100 ppm |
| 3 | DL-a-tocophenyl acetate | Vitamin E | EtOH | #29992-50G-F, Sigma Aldrich | 10, 20, 50, 100 ppm |
| 4 | D-panthenol | Vitamin B5 | EtOH | #76200, Sigma Aldrich | 10, 20, 50, 100 ppm |
| 5 | Caffeine | Caffeine | Water | #C0750, Sigma Aldrich | 10, 20, 50, 100 ppm |

# **Supplementary Table S3. Antibodies for immunostaining and flow cytometry**

| **Name** | **Type** | **Host** | **Brand** | **Catalog number** | **Immunocytochemistry dilution rate** | **FACS dilution rate** |
| --- | --- | --- | --- | --- | --- | --- |
| Anti-Versican | Primary | Mouse | Invitrogen | MA5-27638 | 1/200 | 1/250 |
| Anti-VEGF | Primary | Mouse | BD Biosciences | 554539 | 1/200 | 1/250 |
| Anti-PDGFR-α | Primary | Mouse | R&D systems | AF1062 | 1/200 | 1/250 |
| Anti-PDGFR-β | Primary | Rabbit | Cell Signalling | 3169 | 1/200 | 1/250 |
| Anti-α smooth muscle actin | Primary | Rabbit | R&D systems | MAB1420 | 1/200 | 1/250 |
| Anti-fibronectin | Primary | Rabbit | Abcam | ab2413 | 1/200 | 1/250 |
| Anti-hair cortex Cytokeratin | Primary | Rabbit | Abcam | ab112444 | 1/200 | 1/250 |
| Anti-Keratin 71 | Primary | Rabbit | Abcam | ab247090 | 1/200 | 1/250 |
| Anti-Keratin 5 | Primary | Mouse | Abcam | ab259429 | 1/200 | 1/250 |
| Anti-mouse IgG Alexa Fluor™ 555 | Secondary | Goat | Invitrogen | A28180 | 1/1000 | 1/1500 |
| Anti-rabbit IgG Alexa Fluor™ 488 | Secondary | Goat | Invitrogen | A-11008 | 1/1000 | 1/1500 |
| Anti-mouse IgG Alexa Fluor™ 488 | Secondary | Goat | Invitrogen | A28175 | 1/1000 | 1/1500 |
| Anti-rabbit IgG Alexa Fluor™ 555 | Secondary | Goat | Invitrogen | A27039 | 1/1000 | 1/1500 |

# **Supplementary Table S4. DP spheroids diameter and Keratinocytes outgrowth length from Day 1 to Day 7**

| DP diameter ( µm) | | | | | | | Kera length (µm) | | | | | | |
| --- | --- | --- | --- | --- | --- | --- | --- | --- | --- | --- | --- | --- | --- |
| Day 1 | Day 2 | Day 3 | Day 4 | Day 5 | Day 6 | Day 7 | Day 1 | Day 2 | Day 3 | Day 4 | Day 5 | Day 6 | Day 7 |
| 116.96 | 133.27 | 120.87 | 190.37 | 169.68 | 161.02 | 137.50 | 67.94 | 62.83 | 168.27 | 275.32 | 325.35 | 213.36 | 396.90 |
| 105.36 | 269.49 | 174.06 | 117.26 | 160.79 | 156.73 | 161.46 | 166.60 | 152.96 | 241.09 | 183.21 | 281.95 | 380.53 | 345.80 |
| 90.36 | 261.53 | 251.76 | 179.72 | 99.78 | 168.62 | 136.20 | 129.15 | 127.04 | 205.39 | 244.86 | 202.05 | 258.13 | 187.10 |
| 298.97 | 281.06 | 197.02 | 167.56 | 168.62 | 144.37 | 158.55 | 150.76 | 177.61 | 228.16 | 197.47 | 189.05 | 331.69 | 242.41 |
| 257.94 | 169.26 | 231.61 | 210.21 | 140.34 | 156.73 | 143.87 | 137.62 | 110.41 | 223.90 | 270.29 | 294.50 | 279.20 | 198.17 |
| 295.96 | 207.99 | 258.22 | 163.66 | 150.20 | 177.11 | 137.24 | 84.63 | 118.08 | 192.19 | 260.18 | 230.84 | 297.07 | 342.49 |
| 113.23 | 257.25 | 221.49 | 192.98 | 195.75 | 136.98 | 145.35 | 72.25 | 157.87 | 211.40 | 211.21 | 309.10 | 357.51 | 379.27 |
| 245.42 | 287.99 | 233.15 | 183.27 | 157.87 | 152.57 | 87.13 | 147.34 | 146.15 | 182.42 | 226.32 | 246.30 | 365.75 | 283.52 |
| 109.36 | 258.91 | 94.24 | 152.10 | 122.35 | 129.73 | 128.62 | 84.44 | 152.54 | 176.24 | 243.32 | 260.16 | 377.52 | 369.45 |
| 255.57 | 85.05 | 198.29 | 146.09 | 133.27 | 133.27 | 132.19 | 166.41 | 117.40 | 84.99 | 213.41 | 233.92 | 293.08 | 329.47 |
| 118.78 | 203.81 | 230.53 | 155.13 | 143.87 | 134.88 | 141.36 | 153.48 | 134.58 | 180.69 | 238.80 | 269.09 | 155.79 | 313.69 |
| 292.92 | 244.84 | 209.53 | 150.20 | 136.72 | 125.81 | 133.54 | 162.05 | 112.13 | 198.35 | 248.40 | 265.06 | 295.21 | 356.69 |
| 307.59 | 97.23 | 97.23 | 158.78 | 143.12 | 93.48 | 128.90 | 162.83 | 110.27 | 189.03 | 216.89 | 248.61 | 289.68 | 284.42 |
| 291.81 | 195.20 | 218.56 | 153.03 | 126.38 | 132.46 | 131.92 | 64.57 | 112.98 | 179.63 | 193.15 | 212.92 | 337.73 | 291.95 |
| 299.80 | 203.64 | 211.06 | 132.73 | 114.17 | 138.80 | 88.35 | 150.97 | 121.14 | 171.53 | 213.41 | 206.08 | 290.82 | 281.96 |
| 290.95 | 216.26 | 194.83 | 165.84 | 130.83 | 124.95 | 131.11 | 189.15 | 123.61 | 168.70 | 193.48 | 229.75 | 282.74 | 308.84 |
| 96.49 | 224.70 | 205.04 | 143.37 | 87.54 | 132.19 | 122.93 | 130.01 | 138.43 | 173.90 | 212.24 | 245.13 | 125.16 | 316.95 |
| 333.72 | 202.22 | 185.99 | 101.55 | 141.61 | 136.20 | 130.83 | 178.23 | 85.00 | 120.20 | 141.30 | 221.66 | 313.92 | 213.19 |
| 224.86 | 237.11 | 204.34 | 158.78 | 137.50 | 139.83 | 133.81 | 144.61 | 113.96 | 166.55 | 218.47 | 238.02 | 221.48 | 283.52 |
| 288.11 | 126.66 | 185.99 | 153.50 | 135.67 | 126.94 | 125.24 | 164.39 | 115.07 | 190.55 | 199.67 | 233.00 | 286.23 | 286.10 |
| 317.78 | 212.58 | 144.12 | 164.54 | 141.61 | 148.04 | 126.94 | 178.14 | 127.29 | 182.02 | 217.18 | 207.30 | 283.65 | 310.51 |
| 323.92 | 208.50 | 188.48 | 142.62 | 130.28 | 130.01 | 121.17 | 145.93 | 119.28 | 171.81 | 200.29 | 225.02 | 290.69 | 253.67 |
| 97.23 | 197.20 | 199.37 | 161.02 | 136.20 | 122.93 | 139.32 | 82.15 | 136.81 | 161.11 | 185.07 | 239.22 | 270.92 | 299.41 |
| 301.23 | 112.27 | 196.29 | 144.37 | 124.38 | 102.95 | 96.49 | 66.52 | 106.30 | 181.23 | 201.69 | 211.91 | 257.27 | 251.49 |
| 263.98 | 223.59 | 216.26 | 146.58 | 107.05 | 117.57 | 122.05 | 193.89 | 122.44 | 181.62 | 199.67 | 228.50 | 281.70 | 311.92 |
| 124.09 | 192.80 | 187.52 | 128.90 | 127.79 | 128.90 | 126.66 | 159.08 | 111.85 | 127.39 | 190.72 | 230.84 | 309.56 | 258.41 |
| 305.37 | 194.28 | 187.14 | 149.72 | 131.11 | 124.67 | 154.20 | 157.67 | 121.14 | 171.53 | 208.38 | 212.25 | 237.04 | 320.53 |
| 288.98 | 163.66 | 188.48 | 153.27 | 112.27 | 112.27 | 115.73 | 65.80 | 120.21 | 166.26 | 215.88 | 146.58 | 308.49 | 138.28 |
| 284.23 | 224.39 | 174.67 | 130.83 | 116.34 | 116.34 | 115.73 | 160.47 | 114.24 | 151.88 | 215.30 | 222.78 | 263.34 | 314.51 |
| 126.66 | 223.59 | 189.42 | 129.73 | 125.24 | 119.68 | 114.48 | 161.07 | 116.58 | 162.45 | 199.20 | 238.47 | 297.81 | 277.35 |
| 258.36 | 205.56 | 108.05 | 142.62 | 117.57 | 153.74 | 117.87 | 154.30 | 113.40 | 187.88 | 195.24 | 206.08 | 151.72 | 292.58 |
| 279.53 | 192.05 | 179.32 | 126.38 | 124.67 | 101.91 | 121.76 | 177.07 | 88.67 | 139.31 | 98.97 | 131.11 | 290.94 | 252.22 |
| 298.61 | 161.68 | 208.16 | 193.91 | 168.62 | 111.31 | 99.42 | 148.52 | 120.74 | 175.42 | 202.30 | 217.08 | 190.41 | 169.57 |
| 265.74 | 215.93 | 165.62 | 134.08 | 97.60 | 118.48 | 116.04 | 181.33 | 126.41 | 187.50 | 136.58 | 220.52 | 263.21 | 230.91 |
| 305.60 | 174.47 | 168.62 | 110.02 | 120.58 | 118.48 | 104.68 | 133.99 | 117.81 | 102.23 | 186.75 | 170.31 | 289.17 | 297.19 |
| 87.54 | 226.29 | 166.27 | 121.46 | 110.02 | 114.48 | 114.48 | 211.18 | 89.21 | 135.45 | 146.92 | 200.45 | 253.96 | 293.21 |
| 296.80 | 187.33 | 164.54 | 134.88 | 113.23 | 117.57 | 108.71 | 142.40 | 92.02 | 150.13 | 110.58 | 191.68 | 282.09 | 276.96 |
| 244.69 | 194.28 | 100.85 | 127.22 | 111.95 | 121.76 | 116.96 | 130.13 | 103.73 | 120.20 | 172.15 | 168.62 | 272.67 | 222.14 |
| 335.97 | 134.88 | 209.70 | 121.17 | 113.86 | 118.48 | 100.13 | 136.46 | 101.08 | 72.78 | 170.87 | 163.01 | 301.98 | 232.34 |
| 236.20 | 158.10 | 139.32 | 124.09 | 115.11 | 100.13 | 97.60 | 75.27 | 96.57 | 146.23 | 88.66 | 189.42 | 239.81 | 261.66 |
| 228.34 | 149.96 | 156.28 | 127.51 | 116.96 | 96.86 | 86.30 | 192.82 | 95.08 | 156.41 | 179.42 | 163.01 | 254.97 | 254.25 |
| 255.43 | 153.03 | 155.59 | 101.20 | 103.65 | 94.62 | 116.34 | 135.05 | 103.88 | 158.09 | 190.88 | 223.91 | 254.83 | 238.12 |
| 302.66 | 160.79 | 168.20 | 116.34 | 91.15 | 122.35 | 92.32 | 69.33 | 79.19 | 159.00 | 162.83 | 213.59 | 262.65 | 239.66 |
| 263.85 | 157.19 | 154.66 | 127.51 | 102.26 | 120.87 | 98.33 | 67.47 | 86.30 | 131.12 | 207.63 | 200.45 | 265.01 | 242.71 |
| 92.32 | 147.55 | 160.12 | 108.05 | 113.23 | 110.99 | 113.86 | 140.93 | 98.21 | 136.87 | 81.70 | 166.48 | 262.51 | 223.63 |
| 240.71 | 113.23 | 162.12 | 131.38 | 120.28 | 103.30 | 116.96 | 67.23 | 110.56 | 70.43 | 154.98 | 183.27 | 252.66 | 293.71 |
| 283.35 | 151.86 | 109.04 | 116.04 | 97.60 | 112.59 | 106.04 | 153.99 | 104.95 | 107.28 | 184.90 | 186.76 | 122.49 | 241.65 |
| 267.48 | 106.38 | 162.12 | 92.71 | 106.04 | 85.89 | 109.69 | 116.72 | 99.18 | 155.17 | 177.15 | 203.11 | 231.07 | 228.02 |
| 198.83 | 145.11 | 140.08 | 133.81 | 102.61 | 100.85 | 110.02 | 134.58 | 65.07 | 126.82 | 188.41 | 204.69 | 290.18 | 242.41 |
| 220.68 | 154.90 | 131.38 | 123.80 | 85.89 | 128.62 | 93.86 | 154.61 | 82.73 | 152.51 | 165.68 | 161.68 | 228.83 | 257.84 |
| 235.75 | 113.54 | 118.78 | 115.11 | 97.60 | 111.63 | 88.76 | 139.80 | 90.80 | 165.24 | 189.57 | 168.41 | 243.92 | 183.73 |
| 173.64 | 142.37 | 163.23 | 118.78 | 95.00 | 101.91 | 96.12 | 106.00 | 69.33 | 155.79 | 195.56 | 142.62 | 230.59 | 266.67 |
| 208.33 | 120.87 | 155.13 | 97.60 | 88.35 | 106.71 | 102.26 | 122.44 | 81.17 | 78.20 | 149.03 | 129.18 | 229.63 | 231.54 |
| 122.64 | 85.47 | 161.90 | 96.49 | 106.04 | 129.73 | 97.23 | 67.70 | 101.08 | 90.48 | 139.52 | 134.34 | 276.16 | 161.12 |
| 98.33 | 141.61 | 149.24 | 102.61 | 153.50 | 111.63 | 103.30 | 107.49 | 64.57 | 125.30 | 79.38 | 183.08 | 192.71 | 247.06 |
| 141.86 | 95.75 | 148.76 | 118.78 | 122.93 | 120.87 | 103.99 | 74.21 | 86.30 | 134.92 | 124.14 | 190.18 | 222.97 | 237.04 |
| 231.92 | 116.96 | 116.34 | 125.24 | 174.88 | 148.76 | 137.24 | 92.19 | 95.91 | 112.54 | 85.07 | 176.71 | 191.57 | 199.28 |
| 162.12 | 109.36 | 132.46 | 100.85 | 147.31 | 135.93 | 113.86 | 86.86 | 100.29 | 128.15 | 126.38 | 207.64 | 246.62 | 194.61 |
| 93.86 | 90.36 | 138.54 | 119.68 | 148.76 | 140.60 | 121.46 | 85.75 | 111.56 | 93.36 | 79.38 | 136.72 | 245.72 | 170.65 |
| 276.05 | 135.93 | 142.87 | 95.37 | 161.02 | 121.76 | 128.35 | 140.26 | 98.69 | 85.56 | 178.20 | 160.57 | 202.76 | 175.96 |
| 214.09 | 149.72 | 129.18 | 166.27 | 155.59 | 131.92 | 123.51 | 153.58 | 103.57 | 153.30 | 97.06 | 163.23 | 213.19 | 177.21 |
| 199.91 | 181.70 | 127.79 | 178.12 | 153.27 | 135.67 | 118.78 | 129.27 | 142.06 | 145.23 | 181.15 | 174.47 | 214.05 | 208.13 |
| 138.28 | 127.51 | 164.32 | 137.24 | 156.51 | 119.68 | 143.87 | 65.55 | 135.05 | 144.07 | 163.22 | 315.86 | 222.80 | 214.05 |
| 287.24 | 88.76 | 164.54 | 159.23 | 146.82 | 122.93 | 125.24 | 68.64 | 113.68 | 167.99 | 186.75 | 300.40 | 203.48 | 240.43 |
| 107.38 | 216.09 | 182.10 | 184.25 | 115.11 | 147.80 | 131.92 | 73.13 | 137.50 | 123.94 | 92.11 | 143.37 | 250.46 | 178.86 |
| 87.54 | 236.35 | 136.46 | 166.27 | 170.10 | 146.58 | 137.77 | 129.03 | 130.25 | 176.65 | 229.74 | 295.72 | 249.29 | 277.88 |
| 207.64 | 242.04 | 135.93 | 165.40 | 157.19 | 122.05 | 150.91 | 94.24 | 132.19 | 185.30 | 208.98 | 320.58 | 228.67 | 165.62 |
| 146.58 | 171.78 | 108.71 | 160.35 | 162.57 | 127.22 | 145.85 | 177.88 | 143.62 | 153.46 | 80.16 | 289.72 | 211.80 | 304.53 |
| 123.80 | 244.25 | 238.02 | 161.68 | 169.05 | 127.51 | 120.87 | 73.99 | 118.48 | 183.60 | 190.55 | 309.21 | 285.46 | 238.43 |
| 174.26 | 238.17 | 228.81 | 163.01 | 142.37 | 101.91 | 137.77 | 91.15 | 135.41 | 187.62 | 219.89 | 296.80 | 172.80 | 231.38 |
| 362.32 | 245.57 | 92.71 | 154.20 | 163.66 | 108.38 | 126.09 | 62.57 | 100.93 | 165.82 | 219.89 | 324.36 | 309.08 | 287.89 |
| 308.28 | 221.49 | 160.12 | 178.32 | 172.19 | 149.72 | 128.35 | 73.99 | 139.91 | 161.56 | 202.77 | 320.47 | 276.69 | 284.42 |
| 312.09 | 224.39 | 210.04 | 116.04 | 142.62 | 137.50 | 109.36 | 143.51 | 136.23 | 165.82 | 232.17 | 320.14 | 252.80 | 242.71 |
| 254.17 | 226.29 | 223.10 | 178.72 | 182.49 | 130.01 | 147.31 | 90.62 | 137.74 | 168.70 | 216.89 | 268.02 | 308.61 | 253.82 |
| 100.85 | 153.74 | 221.17 | 160.12 | 152.10 | 127.22 | 133.81 | 95.75 | 137.04 | 197.62 | 240.23 | 306.65 | 299.41 | 282.61 |
| 358.95 | 248.18 | 221.01 | 168.20 | 153.03 | 105.02 | 106.04 | 73.78 | 157.57 | 181.49 | 205.67 | 353.82 | 299.04 | 335.66 |
| 303.60 | 224.54 | 225.18 | 165.40 | 142.62 | 141.10 | 122.93 | 175.44 | 145.71 | 164.37 | 225.77 | 325.02 | 294.21 | 280.91 |
| 335.54 | 242.19 | 225.34 | 177.52 | 142.87 | 151.15 | 125.52 | 149.59 | 115.35 | 212.99 | 240.10 | 330.48 | 189.80 | 333.35 |
| 101.91 | 225.18 | 208.16 | 184.64 | 151.15 | 114.48 | 118.78 | 146.69 | 122.44 | 190.55 | 260.18 | 283.60 | 305.25 | 157.66 |
| 290.83 | 233.30 | 207.30 | 157.42 | 137.24 | 125.52 | 135.93 | 124.25 | 134.93 | 142.73 | 244.22 | 124.09 | 341.31 | 299.54 |
| 275.27 | 235.29 | 202.76 | 168.62 | 185.22 | 149.72 | 137.77 | 178.77 | 135.17 | 201.37 | 231.09 | 122.35 | 195.18 | 281.56 |
| 99.78 | 215.43 | 243.37 | 145.11 | 148.28 | 134.34 | 112.27 | 151.18 | 130.38 | 157.33 | 179.77 | 175.08 | 174.20 | 261.66 |
| 268.69 | 259.60 | 161.90 | 162.79 | 135.93 | 145.85 | 130.56 | 137.74 | 128.04 | 180.69 | 220.74 | 332.54 | 275.36 | 150.99 |
| 153.03 | 221.33 | 217.25 | 141.10 | 137.77 | 114.17 | 140.85 | 131.23 | 134.70 | 192.94 | 222.15 | 275.40 | 203.66 | 253.38 |
| 90.75 | 239.22 | 227.08 | 149.48 | 126.09 | 120.58 | 140.08 | 152.02 | 131.95 | 189.16 | 231.09 | 289.97 | 305.37 | 253.09 |
| 247.31 | 214.60 | 199.01 | 155.13 | 152.57 | 135.67 | 116.96 | 87.40 | 185.41 | 176.52 | 213.99 | 306.19 | 180.39 | 294.33 |
| 250.33 | 208.50 | 86.72 | 142.12 | 119.98 | 139.32 | 120.28 | 127.16 | 135.41 | 170.97 | 236.83 | 337.56 | 279.20 | 226.57 |
| 173.85 | 249.90 | 227.55 | 190.37 | 150.68 | 94.24 | 140.08 | 70.47 | 111.85 | 170.12 | 237.22 | 311.06 | 285.46 | 297.07 |
| 281.95 | 246.30 | 198.65 | 138.28 | 145.11 | 112.27 | 123.51 | 114.66 | 143.40 | 194.55 | 213.70 | 312.90 | 281.70 | 313.57 |
| 288.11 | 275.79 | 226.45 | 162.79 | 131.65 | 129.73 | 129.46 | 132.67 | 136.46 | 156.10 | 226.32 | 329.62 | 297.57 | 169.36 |
| 250.76 | 206.43 | 231.61 | 138.54 | 101.20 | 126.38 | 114.48 | 106.75 | 128.04 | 186.72 | 200.76 | 301.71 | 223.63 | 241.80 |
| 291.69 | 183.08 | 201.69 | 171.36 | 136.20 | 122.05 | 97.60 | 82.53 | 97.72 | 149.80 | 223.41 | 349.44 | 292.33 | 225.10 |
| 259.74 | 237.11 | 190.18 | 122.05 | 105.02 | 96.12 | 131.92 | 123.61 | 106.75 | 151.24 | 186.08 | 288.73 | 243.01 | 219.48 |
| 98.33 | 235.44 | 210.04 | 154.20 | 135.67 | 139.83 | 125.52 | 137.16 | 99.18 | 236.15 | 289.14 | 280.30 | 307.17 | 134.23 |
| 95.75 | 203.28 | 187.52 | 106.71 | 93.48 | 130.28 | 102.61 | 144.06 | 112.27 | 156.72 | 190.06 | 288.73 | 236.10 | 240.27 |
| 92.71 | 172.19 | 193.72 | 138.80 | 168.20 | 110.67 | 87.54 | 131.23 | 96.08 | 177.60 | 193.96 | 388.28 | 282.87 | 239.51 |
| 260.57 | 178.72 | 165.84 | 159.45 | 127.22 | 109.04 | 116.96 | 126.03 | 118.48 | 201.96 | 211.80 | 286.99 | 280.78 | 192.33 |
| 95.75 | 166.91 | 163.44 | 104.68 | 132.46 | 105.70 | 93.48 | 130.86 | 116.99 | 105.24 | 228.79 | 329.94 | 269.42 | 269.15 |
| 259.33 | 177.72 | 236.66 | 142.12 | 101.20 | 134.34 | 90.36 | 138.77 | 88.31 | 153.61 | 171.78 | 167.13 | 359.46 | 297.57 |
| 284.36 | 248.76 | 199.37 | 138.28 | 145.11 | 112.27 | 123.51 | 125.40 | 132.19 | 179.49 | 228.65 | 243.52 | 267.23 | 358.95 |

**Supplementary Table S5. Percentages of Live/Dead cells of DP spheroids on Day 2**

| Number | Live Cells (%) | Dead Cells (%) |
| --- | --- | --- |
| 1 | 94.36 | 5.64 |
| 2 | 93.67 | 6.33 |
| 3 | 94.90 | 5.10 |
| 4 | 94.75 | 5.25 |
| 5 | 93.29 | 6.71 |
| 6 | 90.82 | 9.18 |
| 7 | 90.80 | 9.20 |
| 8 | 90.86 | 9.14 |
| 9 | 91.62 | 8.38 |
| 10 | 95.03 | 4.97 |
| 11 | 93.26 | 6.74 |
| 12 | 91.92 | 8.08 |
| 13 | 91.52 | 8.48 |
| 14 | 93.38 | 6.62 |
| 15 | 89.89 | 10.11 |
| 16 | 93.86 | 6.14 |
| 17 | 92.34 | 7.66 |
| 18 | 92.97 | 7.03 |
| 19 | 95.80 | 4.20 |
| 20 | 95.72 | 4.28 |
| 21 | 94.37 | 5.63 |
| 22 | 93.32 | 6.68 |
| 23 | 93.22 | 6.78 |
| 24 | 91.36 | 8.64 |
| 25 | 93.00 | 7.00 |
| 26 | 96.67 | 3.33 |
| 27 | 91.46 | 8.54 |
| 28 | 94.30 | 5.70 |
| 29 | 95.30 | 4.70 |
| 30 | 93.84 | 6.16 |
| 31 | 95.32 | 4.68 |
| 32 | 96.24 | 3.76 |
| 33 | 99.80 | 0.20 |
| 34 | 92.15 | 7.85 |
| 35 | 94.15 | 5.85 |
| 36 | 95.55 | 4.45 |
| 37 | 95.12 | 4.88 |
| 38 | 91.12 | 8.88 |
| 39 | 92.00 | 8.00 |
| 40 | 94.66 | 5.34 |
| 41 | 89.79 | 10.21 |
| 42 | 96.82 | 3.18 |
| 43 | 91.26 | 8.74 |
| 44 | 96.12 | 3.88 |
| 45 | 96.69 | 3.31 |
| 46 | 89.25 | 10.75 |
| 47 | 94.79 | 5.21 |
| 48 | 91.19 | 8.81 |
| 49 | 90.18 | 9.82 |
| 50 | 91.45 | 8.55 |

# **Supplementary Table S6. Expression abundance of all ECM proteins identified in DPCs cultured in 2D and 3D culture platforms expressed proteins with gene ontology.** The protein abundance values from each sample were log2 transformed and normalized**.**

| **No.** | **3D PAMCELL** | **3D ULA** | **2D** | **Annotated Matrisome Division** | **Annotated Matrisome Category** | **Cluster** | **Protein** | **Protein Description** | **GO term** | **Annotated Gene** | **Gene ontology 1** | | **Gene ontology 2** | | | **Gene ontology 3** | | **Gene ontology 4** |
| --- | --- | --- | --- | --- | --- | --- | --- | --- | --- | --- | --- | --- | --- | --- | --- | --- | --- | --- |
| 1 | 14.332 | 15.931 | 17.179 | Matrisome-associated | ECM Regulators | Cluster 112 | sp|P28300|LYOX_HUMAN | Protein-lysine 6-oxidase | GO:CC extracellular matrix (GO:0005576, GO:0005578 & GO:0031012) & GO:CC not extracellular matrix | LOX | Collagen trimerization | | fibre formation | | |  | |  |
| 2 | 14.11 | 14.275 | 13.817 | Core matrisome | ECM Glycoproteins | Cluster -145 | sp|Q13751|LAMB3_HUMAN | Laminin subunit beta-3 | GO:CC extracellular matrix (GO:0005576, GO:0005578 & GO:0031012) & GO:CC not extracellular matrix | LAMB3 | extracellular matrix structural constituent | | cell adhesion | | | Glycoprotein | |  |
| 3 | 13.688 | 13.807 | 13.581 | Core matrisome | Collagens | Cluster -145 | sp|Q96P44|COLA1_HUMAN | Collagen alpha-1(XXI) chain | GO:CC extracellular matrix (GO:0005576, GO:0005578 & GO:0031012) & GO:CC not extracellular matrix | COL21A1 | extracellular matrix structural constituent | | cell adhesion | | | Glycoprotein | |  |
| 4 | 14.735 | 14.475 | 13.21 | Matrisome-associated | ECM-affiliated Proteins | Cluster -145 | sp|O43157|PLXB1_HUMAN | Plexin-B1 | GO:CC extracellular matrix (GO:0005576, GO:0005578 & GO:0031012) & GO:CC not extracellular matrix | PLXNB1 | extracellular matrix structural constituent | | cell adhesion | | | Glycoprotein | |  |
| 5 | 14.726 | 14.375 | 14.18 | Core matrisome | ECM Glycoproteins | Cluster -145 | sp|O15230|LAMA5_HUMAN | Laminin subunit alpha-5 | GO:CC extracellular matrix (GO:0005576, GO:0005578 & GO:0031012) & GO:CC not extracellular matrix | LAMA5 | extracellular matrix structural constituent | | cell adhesion | | | Glycoprotein | |  |
| 6 | 14.64 | 14.944 | 14.26 | Core matrisome | ECM Glycoproteins | Cluster -145 | sp|Q16787|LAMA3_HUMAN | Laminin subunit alpha-3 | GO:CC extracellular matrix (GO:0005576, GO:0005578 & GO:0031012) & GO:CC not extracellular matrix | LAMA3 | extracellular matrix structural constituent | | cell adhesion | | | Glycoprotein | |  |
| 7 | 14.05 | 12.461 | 13.595 | Matrisome-associated | Secreted Factors | Cluster -160 | sp|Q86YZ3|HORN_HUMAN | Hornerin | GO:CC extracellular matrix (GO:0005576, GO:0005578 & GO:0031012) & GO:CC not extracellular matrix | HRNR | tissue development | | epidermis development | | | cell periphery | |  |
| 8 | 13.14 | 13.486 | 14.301 | Core matrisome | ECM Glycoproteins | Cluster -160 | sp|Q9HB63|NET4_HUMAN | Netrin-4 | GO:CC extracellular matrix (GO:0005576, GO:0005578 & GO:0031012) & GO:CC not extracellular matrix | NTN4 | tissue development | | epidermis development | | | cell periphery | |  |
| 9 | 13.207 | 13.128 | 14.438 | Core matrisome | ECM Glycoproteins | Cluster -160 | sp|Q13753|LAMC2_HUMAN | Laminin subunit gamma-2 | GO:CC extracellular matrix (GO:0005576, GO:0005578 & GO:0031012) & GO:CC not extracellular matrix | LAMC2 | tissue development | | epidermis development | | | cell periphery | |  |
| 10 | 16.168 | 15.263 | 14.119 | Core matrisome | ECM Glycoproteins | Cluster -128 | sp|Q9Y6N6|LAMC3_HUMAN | Laminin subunit gamma-3 | GO:CC extracellular matrix (GO:0005576, GO:0005578 & GO:0031012) & GO:CC not extracellular matrix | LAMC3 | cell adhesion | | protein maturation | | | Secreted protein | |  |
| 11 | 15.552 | 15.665 | 13.415 | Matrisome-associated | ECM Regulators | Cluster -128 | sp|P00740|FA9_HUMAN | Coagulation factor IX | GO:CC extracellular matrix (GO:0005576, GO:0005578 & GO:0031012) & GO:CC not extracellular matrix | F9 | cell adhesion | | protein maturation | | | Secreted protein | |  |
| 12 | 15.795 | 15.827 | 13.717 | Core matrisome | ECM Glycoproteins | Cluster -128 | sp|Q9HCB6|SPON1_HUMAN | Spondin-1 | GO:CC extracellular matrix (GO:0005576, GO:0005578 & GO:0031012) & GO:CC not extracellular matrix | SPON1 | cell adhesion | | protein maturation | | | Secreted protein | |  |
| 13 | 15.405 | 15.629 | 14.105 | Matrisome-associated | ECM Regulators | Cluster -128 | sp|Q6UY14|ATL4_HUMAN | ADAMTS-like protein 4 | GO:CC extracellular matrix (GO:0005576, GO:0005578 & GO:0031012) & GO:CC not extracellular matrix | ADAMTSL4 | cell adhesion | | protein maturation | | | Secreted protein | |  |
| 14 | 15.262 | 14.045 | 15.15 | Core matrisome | ECM Glycoproteins | Cluster -149 | sp|O75339|CILP1_HUMAN | Cartilage intermediate layer protein 1 | GO:CC not extracellular matrix | CILP | regulation of protein, peptide and amine transport | | | establishment of protein localization | | |  | |
| 15 | 15.427 | 12.982 | 14.689 | Core matrisome | ECM Glycoproteins | Cluster -149 | sp|P02679|FIBG_HUMAN | Fibrinogen gamma chain | GO:CC extracellular matrix (GO:0005576, GO:0005578 & GO:0031012) & GO:CC not extracellular matrix | FGG | regulation of protein, peptide and amine transport | | | establishment of protein localization | | |  | |
| 16 | 16.172 | 14.313 | 16.167 | Core matrisome | ECM Glycoproteins | Cluster -164 | sp|P04275|VWF_HUMAN | von Willebrand factor | GO:CC extracellular matrix (GO:0005576, GO:0005578 & GO:0031012) & GO:CC not extracellular matrix | VWF | receptor binding | | | blood vessel development | | | regulation of cellular process | |
| 17 | 14.409 | 14.697 | 15.9 | Core matrisome | Collagens | Cluster -164 | sp|P02462|CO4A1_HUMAN | Collagen alpha-1(IV) chain | GO:CC extracellular matrix (GO:0005576, GO:0005578 & GO:0031012) & GO:CC not extracellular matrix | COL4A1 | receptor binding | | | blood vessel development | | | regulation of cellular process | |
| 18 | 15.483 | 15.561 | 14.961 | Matrisome-associated | ECM Regulators | Cluster -164 | sp|O75635|SPB7_HUMAN | Serpin B7 | GO:CC not extracellular matrix | SERPINB7 | receptor binding | | | blood vessel development | | | regulation of cellular process | |
| 19 | 15.328 | 15.417 | 15.065 | Matrisome-associated | ECM-affiliated Proteins | Cluster -164 | sp|Q9NPR2|SEM4B_HUMAN | Semaphorin-4B | GO:CC not extracellular matrix | SEMA4B | receptor binding | | | blood vessel development | | | regulation of cellular process | |
| 20 | 15.412 | 15.63 | 15.598 | Matrisome-associated | Secreted Factors | Cluster -164 | sp|P33764|S10A3_HUMAN | Protein S100-A3 | GO:CC not extracellular matrix | S100A3 | receptor binding | | | blood vessel development | | | regulation of cellular process | |
| 21 | 15.318 | 15.289 | 15.731 | Core matrisome | ECM Glycoproteins | Cluster -164 | sp|P49747|COMP_HUMAN | Cartilage oligomeric matrix protein | GO:CC extracellular matrix (GO:0005576, GO:0005578 & GO:0031012) & GO:CC not extracellular matrix | COMP | receptor binding | | | blood vessel development | | | regulation of cellular process | |
| 22 | 15.32 | 15.214 | 16.195 | Matrisome-associated | ECM Regulators | Cluster -164 | sp|Q13444|ADA15_HUMAN | Disintegrin and metalloproteinase domain-containing protein 15 | GO:CC not extracellular matrix | ADAM15 | receptor binding | | | blood vessel development | | | regulation of cellular process | |
| 23 | 17.258 | 16.11 | 12.158 | Core matrisome | ECM Regulators | Cluster 120 | sp|P08493|MGP_HUMAN | Matrix Gla protein | GO:CC extracellular matrix (GO:0005576, GO:0005578 & GO:0031012) & GO:CC not extracellular matrix | MGP | Bone structure | | calcium ion binding | | |  | |  |
| 24 | 20.8 | 20.846 | 20.566 | Matrisome-associated | ECM-affiliated Proteins | Cluster -137 | sp|P08133|ANXA6_HUMAN | Annexin A6 | GO:CC not extracellular matrix | ANXA6 | Extracellular exosome | | cell communication | | |  | |  |
| 25 | 21.215 | 21.237 | 20.454 | Matrisome-associated | Secreted Factors | Cluster -137 | sp|P26447|S10A4_HUMAN | Protein S100-A4 | GO:CC extracellular matrix (GO:0005576, GO:0005578 & GO:0031012) & GO:CC not extracellular matrix | S100A4 | Extracellular exosome | | cell communication | | |  | |  |
| 26 | 21.028 | 21.127 | 20.828 | Matrisome-associated | ECM-affiliated Proteins | Cluster -137 | sp|P08758|ANXA5_HUMAN | Annexin A5 | GO:CC extracellular matrix (GO:0005576, GO:0005578 & GO:0031012) & GO:CC not extracellular matrix | ANXA5 | Extracellular exosome | | cell communication | | |  | |  |
| 27 | 20.422 | 20.306 | 20.303 | Matrisome-associated | Secreted Factors | Cluster -137 | sp|Q99584|S10AD_HUMAN | Protein S100-A13 | GO:CC extracellular matrix (GO:0005576, GO:0005578 & GO:0031012) & GO:CC not extracellular matrix | S100A13 | Extracellular exosome | | cell communication | | |  | |  |
| 28 | 20.095 | 19.284 | 20.357 | Matrisome-associated | ECM Regulators | Cluster -157 | sp|Q14624|ITIH4_HUMAN | Inter-alpha-trypsin inhibitor heavy chain H4 | GO:CC extracellular matrix (GO:0005576, GO:0005578 & GO:0031012) & GO:CC not extracellular matrix | ITIH4 | response to stress | | cell-cell adhesion | | | extracellular vesicle | | calcium ion binding |
| 29 | 20.392 | 19.157 | 20.062 | Matrisome-associated | ECM Regulators | Cluster -157 | sp|Q06033|ITIH3_HUMAN | Inter-alpha-trypsin inhibitor heavy chain H3 | GO:CC extracellular matrix (GO:0005576, GO:0005578 & GO:0031012) & GO:CC not extracellular matrix | ITIH3 | response to stress | | cell-cell adhesion | | | extracellular vesicle | | calcium ion binding |
| 30 | 20.251 | 20.524 | 19.073 | Matrisome-associated | ECM-affiliated Proteins | Cluster -157 | sp|P17931|LEG3_HUMAN | Galectin-3 | GO:CC extracellular matrix (GO:0005576, GO:0005578 & GO:0031012) & GO:CC not extracellular matrix | LGALS3 | response to stress | | cell-cell adhesion | | | extracellular vesicle | | calcium ion binding |
| 31 | 19.931 | 19.859 | 18.776 | Matrisome-associated | ECM-affiliated Proteins | Cluster -157 | sp|P09525|ANXA4_HUMAN | Annexin A4 | GO:CC not extracellular matrix | ANXA4 | response to stress | | cell-cell adhesion | | | extracellular vesicle | | calcium ion binding |
| 32 | 19.416 | 19.458 | 18.916 | Matrisome-associated | ECM Regulators | Cluster -157 | sp|P35237|SPB6_HUMAN | Serpin B6 | GO:CC extracellular matrix (GO:0005576, GO:0005578 & GO:0031012) & GO:CC not extracellular matrix | SERPINB6 | response to stress | | cell-cell adhesion | | | extracellular vesicle | | calcium ion binding |
| 33 | 19.63 | 19.591 | 18.871 | Matrisome-associated | ECM-affiliated Proteins | Cluster -157 | sp|P50995|ANX11_HUMAN | Annexin A11 | GO:CC not extracellular matrix | ANXA11 | response to stress | | cell-cell adhesion | | | extracellular vesicle | | calcium ion binding |
| 34 | 19.899 | 19.8 | 19.618 | Matrisome-associated | Secreted Factors | Cluster -157 | sp|P31949|S10AB_HUMAN | Protein S100-A11 | GO:CC extracellular matrix (GO:0005576, GO:0005578 & GO:0031012) & GO:CC not extracellular matrix | S100A11 | response to stress | | cell-cell adhesion | | | extracellular vesicle | | calcium ion binding |
| 35 | 19.809 | 19.79 | 19.36 | Matrisome-associated | ECM-affiliated Proteins | Cluster -157 | sp|P09382|LEG1_HUMAN | Galectin-1 | GO:CC extracellular matrix (GO:0005576, GO:0005578 & GO:0031012) & GO:CC not extracellular matrix | LGALS1 | response to stress | | cell-cell adhesion | | | extracellular vesicle | | calcium ion binding |
| 36 | 19.538 | 19.945 | 19.678 | Matrisome-associated | ECM-affiliated Proteins | Cluster -157 | sp|P07355|ANXA2_HUMAN | Annexin A2 | GO:CC extracellular matrix (GO:0005576, GO:0005578 & GO:0031012) & GO:CC not extracellular matrix | ANXA2 | response to stress | | cell-cell adhesion | | | extracellular vesicle | | calcium ion binding |
| 37 | 19.644 | 20.232 | 19.84 | Matrisome-associated | ECM-affiliated Proteins | Cluster -157 | sp|P04083|ANXA1_HUMAN | Annexin A1 | GO:CC extracellular matrix (GO:0005576, GO:0005578 & GO:0031012) & GO:CC not extracellular matrix | ANXA1 | response to stress | | cell-cell adhesion | | | extracellular vesicle | | calcium ion binding |
| 38 | 20.319 | 20.954 | 17.425 | Core matrisome | ECM Glycoproteins | Cluster -162 | sp|Q15582|BGH3_HUMAN | Transforming growth factor-beta-induced protein ig-h3 | GO:CC extracellular matrix (GO:0005576, GO:0005578 & GO:0031012) & GO:CC not extracellular matrix | TGFBI | system development | | | glycosaminoglycan binding | | | carbohydrate derivative binding | |
| 39 | 19.571 | 19.985 | 17.966 | Core matrisome | Collagens | Cluster -162 | sp|P12111|CO6A3_HUMAN | Collagen alpha-3(VI) chain | GO:CC extracellular matrix (GO:0005576, GO:0005578 & GO:0031012) & GO:CC not extracellular matrix | COL6A3 | system development | | | glycosaminoglycan binding | | | carbohydrate derivative binding | |
| 40 | 19.919 | 19.99 | 17.876 | Core matrisome | Proteoglycans | Cluster -162 | sp|P21810|PGS1_HUMAN | Biglycan | GO:CC extracellular matrix (GO:0005576, GO:0005578 & GO:0031012) & GO:CC not extracellular matrix | BGN | system development | | | glycosaminoglycan binding | | | carbohydrate derivative binding | |
| 41 | 21.043 | 20.734 | 18.416 | Matrisome-associated | ECM Regulators | Cluster -162 | sp|Q14520|HABP2_HUMAN | Hyaluronan-binding protein 2 | GO:CC extracellular matrix (GO:0005576, GO:0005578 & GO:0031012) & GO:CC not extracellular matrix | HABP2 | system development | | | glycosaminoglycan binding | | | carbohydrate derivative binding | |
| 42 | 21.487 | 20.204 | 17.872 | Core matrisome | ECM Glycoproteins | Cluster -162 | sp|P04004|VTNC_HUMAN | Vitronectin | GO:CC extracellular matrix (GO:0005576, GO:0005578 & GO:0031012) & GO:CC not extracellular matrix | VTN | system development | | | glycosaminoglycan binding | | | carbohydrate derivative binding | |
| 43 | 19.456 | 19.583 | 15.918 | Core matrisome | ECM Glycoproteins | Cluster -156 | sp|P23142|FBLN1_HUMAN | Fibulin-1 | GO:CC extracellular matrix (GO:0005576, GO:0005578 & GO:0031012) & GO:CC not extracellular matrix | FBLN1 | regulation of cellular process | | | macromolecule metabolic process | | | cellular response to stimulus | |
| 44 | 18.928 | 19.99 | 15.809 | Matrisome-associated | ECM Regulators | Cluster -156 | sp|P03956|MMP1_HUMAN | Interstitial collagenase | GO:CC extracellular matrix (GO:0005576, GO:0005578 & GO:0031012) & GO:CC not extracellular matrix | MMP1 | regulation of cellular process | | | macromolecule metabolic process | | | cellular response to stimulus | |
| 45 | 19.551 | 20.062 | 16.837 | Core matrisome | Proteoglycans | Cluster -156 | sp|P07585|PGS2_HUMAN | Decorin | GO:CC extracellular matrix (GO:0005576, GO:0005578 & GO:0031012) & GO:CC not extracellular matrix | DCN | regulation of cellular process | | | macromolecule metabolic process | | | cellular response to stimulus | |
| 46 | 19.324 | 19.233 | 16.896 | Matrisome-associated | ECM Regulators | Cluster -156 | sp|P21980|TGM2_HUMAN | Protein-glutamine gamma-glutamyltransferase 2 | GO:CC extracellular matrix (GO:0005576, GO:0005578 & GO:0031012) & GO:CC not extracellular matrix | TGM2 | regulation of cellular process | | | macromolecule metabolic process | | | cellular response to stimulus | |
| 47 | 19.26 | 18.971 | 16.231 | Matrisome-associated | ECM Regulators | Cluster -156 | sp|P35625|TIMP3_HUMAN | Metalloproteinase inhibitor 3 | GO:CC extracellular matrix (GO:0005576, GO:0005578 & GO:0031012) & GO:CC not extracellular matrix | TIMP3 | regulation of cellular process | | | macromolecule metabolic process | | | cellular response to stimulus | |
| 48 | 18.189 | 19.157 | 16.116 | Core matrisome | ECM Glycoproteins | Cluster -156 | sp|P24593|IBP5_HUMAN | Insulin-like growth factor-binding protein 5 | GO:CC extracellular matrix (GO:0005576, GO:0005578 & GO:0031012) & GO:CC not extracellular matrix | IGFBP5 | regulation of cellular process | | | macromolecule metabolic process | | | cellular response to stimulus | |
| 49 | 19.997 | 17.6 | 19.114 | Matrisome-associated | ECM Regulators | Cluster 142 | sp|P20742|PZP_HUMAN | Pregnancy zone protein | GO:CC extracellular matrix (GO:0005576, GO:0005578 & GO:0031012) & GO:CC not extracellular matrix | PZP | cell adhesion | | plasmin signaling | | |  | |  |
| 50 | 19.847 | 18.3 | 17.668 | Matrisome-associated | ECM Regulators | Cluster -163 | sp|P00734|THRB_HUMAN | Prothrombin | GO:CC extracellular matrix (GO:0005576, GO:0005578 & GO:0031012) & GO:CC not extracellular matrix | F2 | regulation of protein metabolic process | | proteolysis | | | biological regulation | |  |
| 51 | 18.407 | 18.364 | 17.334 | Matrisome-associated | ECM-affiliated Proteins | Cluster -163 | sp|P35052|GPC1_HUMAN | Glypican-1 | GO:CC extracellular matrix (GO:0005576, GO:0005578 & GO:0031012) & GO:CC not extracellular matrix | GPC1 | regulation of protein metabolic process | | proteolysis | | | biological regulation | |  |
| 52 | 18.604 | 18.324 | 17.033 | Core matrisome | ECM Glycoproteins | Cluster -163 | sp|P02751|FINC_HUMAN | Fibronectin | GO:CC extracellular matrix (GO:0005576, GO:0005578 & GO:0031012) & GO:CC not extracellular matrix | FN1 | regulation of protein metabolic process | | proteolysis | | | biological regulation | |  |
| 53 | 18.589 | 17.955 | 17.794 | Matrisome-associated | ECM Regulators | Cluster -163 | sp|P00747|PLMN_HUMAN | Plasminogen | GO:CC extracellular matrix (GO:0005576, GO:0005578 & GO:0031012) & GO:CC not extracellular matrix | PLG | regulation of protein metabolic process | | proteolysis | | | biological regulation | |  |
| 54 | 18.123 | 18.089 | 17.648 | Matrisome-associated | ECM-affiliated Proteins | Cluster -163 | sp|P49257|LMAN1_HUMAN | Protein ERGIC-53 | GO:CC not extracellular matrix | LMAN1 | regulation of protein metabolic process | | proteolysis | | | biological regulation | |  |
| 55 | 18.371 | 18.022 | 17.591 | Matrisome-associated | ECM-affiliated Proteins | Cluster -163 | sp|Q6UVK1|CSPG4_HUMAN | Chondroitin sulfate proteoglycan 4 | GO:CC extracellular matrix (GO:0005576, GO:0005578 & GO:0031012) & GO:CC not extracellular matrix | CSPG4 | regulation of protein metabolic process | | proteolysis | | | biological regulation | |  |
| 56 | 18.085 | 18.709 | 17.025 | Matrisome-associated | ECM Regulators | Cluster -163 | sp|P50281|MMP14_HUMAN | Matrix metalloproteinase-14 | GO:CC extracellular matrix (GO:0005576, GO:0005578 & GO:0031012) & GO:CC not extracellular matrix | MMP14 | regulation of protein metabolic process | | proteolysis | | | biological regulation | |  |
| 57 | 18.262 | 19.061 | 17.023 | Core matrisome | Collagens | Cluster -163 | sp|Q99715|COCA1_HUMAN | Collagen alpha-1(XII) chain | GO:CC extracellular matrix (GO:0005576, GO:0005578 & GO:0031012) & GO:CC not extracellular matrix | COL12A1 | regulation of protein metabolic process | | proteolysis | | | biological regulation | |  |
| 58 | 19.399 | 18.916 | 17.656 | Matrisome-associated | ECM Regulators | Cluster -163 | sp|P07858|CATB_HUMAN | Cathepsin B | GO:CC extracellular matrix (GO:0005576, GO:0005578 & GO:0031012) & GO:CC not extracellular matrix | CTSB | regulation of protein metabolic process | | proteolysis | | | biological regulation | |  |
| 59 | 18.635 | 19.323 | 17.707 | Matrisome-associated | ECM Regulators | Cluster -163 | sp|P07093|GDN_HUMAN | Glia-derived nexin | GO:CC extracellular matrix (GO:0005576, GO:0005578 & GO:0031012) & GO:CC not extracellular matrix | SERPINE2 | regulation of protein metabolic process | | proteolysis | | | biological regulation | |  |
| 60 | 18.772 | 19.108 | 17.542 | Core matrisome | Proteoglycans | Cluster -163 | sp|P51884|LUM_HUMAN | Lumican | GO:CC extracellular matrix (GO:0005576, GO:0005578 & GO:0031012) & GO:CC not extracellular matrix | LUM | regulation of protein metabolic process | | proteolysis | | | biological regulation | |  |
| 61 | 18.872 | 18.968 | 17.308 | Matrisome-associated | ECM Regulators | Cluster -163 | sp|P07339|CATD_HUMAN | Cathepsin D | GO:CC extracellular matrix (GO:0005576, GO:0005578 & GO:0031012) & GO:CC not extracellular matrix | CTSD | regulation of protein metabolic process | | proteolysis | | | biological regulation | |  |
| 62 | 18.985 | 18.589 | 17.402 | Matrisome-associated | ECM Regulators | Cluster -163 | sp|Q9UBR2|CATZ_HUMAN | Cathepsin Z | GO:CC extracellular matrix (GO:0005576, GO:0005578 & GO:0031012) & GO:CC not extracellular matrix | CTSZ | regulation of protein metabolic process | | proteolysis | | | biological regulation | |  |
| 63 | 19.047 | 18.649 | 17.414 | Matrisome-associated | ECM Regulators | Cluster -163 | sp|P10619|PPGB_HUMAN | Lysosomal protective protein | GO:CC extracellular matrix (GO:0005576, GO:0005578 & GO:0031012) & GO:CC not extracellular matrix | CTSA | regulation of protein metabolic process | | proteolysis | | | biological regulation | |  |
| 64 | 18.798 | 18.884 | 17.004 | Core matrisome | Collagens | Cluster -163 | sp|P12109|CO6A1_HUMAN | Collagen alpha-1(VI) chain | GO:CC extracellular matrix (GO:0005576, GO:0005578 & GO:0031012) & GO:CC not extracellular matrix | COL6A1 | regulation of protein metabolic process | | proteolysis | | | biological regulation | |  |
| 65 | 19.347 | 19.477 | 17.691 | Core matrisome | Collagens | Cluster -163 | sp|P12110|CO6A2_HUMAN | Collagen alpha-2(VI) chain | GO:CC extracellular matrix (GO:0005576, GO:0005578 & GO:0031012) & GO:CC not extracellular matrix | COL6A2 | regulation of protein metabolic process | | proteolysis | | | biological regulation | |  |
| 66 | 18.936 | 19.575 | 17.93 | Matrisome-associated | ECM Regulators | Cluster -163 | sp|P16035|TIMP2_HUMAN | Metalloproteinase inhibitor 2 | GO:CC extracellular matrix (GO:0005576, GO:0005578 & GO:0031012) & GO:CC not extracellular matrix | TIMP2 | regulation of protein metabolic process | | proteolysis | | | biological regulation | |  |
| 67 | 18.941 | 19.661 | 17.584 | Matrisome-associated | ECM Regulators | Cluster -163 | sp|Q6YHK3|CD109_HUMAN | CD109 antigen | GO:CC extracellular matrix (GO:0005576, GO:0005578 & GO:0031012) & GO:CC not extracellular matrix | CD109 | regulation of protein metabolic process | | proteolysis | | | biological regulation | |  |
| 68 | 19.309 | 18.903 | 19.213 | Matrisome-associated | Secreted Factors | Cluster -163 | sp|Q96FQ6|S10AG_HUMAN | Protein S100-A16 | GO:CC not extracellular matrix | S100A16 | regulation of protein metabolic process | | proteolysis | | | biological regulation | |  |
| 69 | 18.781 | 18.933 | 19.29 | Matrisome-associated | ECM Regulators | Cluster -163 | sp|P50454|SERPH_HUMAN | Serpin H1 | GO:CC not extracellular matrix | SERPINH1 | regulation of protein metabolic process | | proteolysis | | | biological regulation | |  |
| 70 | 18.79 | 18.659 | 19.056 | Core matrisome | ECM Glycoproteins | Cluster -163 | sp|Q6UXH1|CREL2_HUMAN | Protein disulfide isomerase CRELD2 | GO:CC not extracellular matrix | CRELD2 | regulation of protein metabolic process | | proteolysis | | | biological regulation | |  |
| 71 | 18.392 | 19.008 | 18.286 | Matrisome-associated | ECM Regulators | Cluster -163 | sp|P13674|P4HA1_HUMAN | Prolyl 4-hydroxylase subunit alpha-1 | GO:CC not extracellular matrix | P4HA1 | regulation of protein metabolic process | | proteolysis | | | biological regulation | |  |
| 72 | 18.501 | 18.65 | 18.34 | Core matrisome | ECM Glycoproteins | Cluster -163 | sp|P07996|TSP1_HUMAN | Thrombospondin-1 | GO:CC extracellular matrix (GO:0005576, GO:0005578 & GO:0031012) & GO:CC not extracellular matrix | THBS1 | regulation of protein metabolic process | | proteolysis | | | biological regulation | |  |
| 73 | 18.184 | 18.63 | 18.256 | Matrisome-associated | ECM Regulators | Cluster -163 | sp|Q9GZT9|EGLN1_HUMAN | Egl nine homolog 1 | GO:CC not extracellular matrix | EGLN1 | regulation of protein metabolic process | | proteolysis | | | biological regulation | |  |
| 74 | 18.806 | 18.833 | 18.2 | Matrisome-associated | Secreted Factors | Cluster -163 | sp|P06703|S10A6_HUMAN | Protein S100-A6 | GO:CC extracellular matrix (GO:0005576, GO:0005578 & GO:0031012) & GO:CC not extracellular matrix | S100A6 | regulation of protein metabolic process | | proteolysis | | | biological regulation | |  |
| 75 | 18.95 | 18.869 | 18.517 | Matrisome-associated | ECM Regulators | Cluster -163 | sp|P30740|ILEU_HUMAN | Leukocyte elastase inhibitor | GO:CC extracellular matrix (GO:0005576, GO:0005578 & GO:0031012) & GO:CC not extracellular matrix | SERPINB1 | regulation of protein metabolic process | | proteolysis | | | biological regulation | |  |
| 76 | 18.844 | 18.7 | 18.457 | Core matrisome | ECM Glycoproteins | Cluster -163 | sp|P07942|LAMB1_HUMAN | Laminin subunit beta-1 | GO:CC extracellular matrix (GO:0005576, GO:0005578 & GO:0031012) & GO:CC not extracellular matrix | LAMB1 | regulation of protein metabolic process | | proteolysis | | | biological regulation | |  |
| 77 | 18.377 | 18.856 | 18.901 | Matrisome-associated | ECM Regulators | Cluster -163 | sp|O60568|PLOD3_HUMAN | Multifunctional procollagen lysine hydroxylase and glycosyltransferase LH3 | GO:CC not extracellular matrix | PLOD3 | regulation of protein metabolic process | | proteolysis | | | biological regulation | |  |
| 78 | 18.394 | 18.797 | 18.631 | Matrisome-associated | ECM Regulators | Cluster -163 | sp|Q02809|PLOD1_HUMAN | Procollagen-lysine,2-oxoglutarate 5-dioxygenase 1 | GO:CC not extracellular matrix | PLOD1 | regulation of protein metabolic process | | proteolysis | | | biological regulation | |  |
| 79 | 18.744 | 18.594 | 18.718 | Matrisome-associated | ECM Regulators | Cluster -163 | sp|P50452|SPB8_HUMAN | Serpin B8 | GO:CC not extracellular matrix | SERPINB8 | regulation of protein metabolic process | | proteolysis | | | biological regulation | |  |
| 80 | 18.658 | 18.807 | 18.643 | Matrisome-associated | ECM Regulators | Cluster -163 | sp|P04080|CYTB_HUMAN | Cystatin-B | GO:CC extracellular matrix (GO:0005576, GO:0005578 & GO:0031012) & GO:CC not extracellular matrix | CSTB | regulation of protein metabolic process | | proteolysis | | | biological regulation | |  |
| 81 | 18.941 | 19.377 | 18.649 | Matrisome-associated | Secreted Factors | Cluster -163 | sp|P60903|S10AA_HUMAN | Protein S100-A10 | GO:CC extracellular matrix (GO:0005576, GO:0005578 & GO:0031012) & GO:CC not extracellular matrix | S100A10 | regulation of protein metabolic process | | proteolysis | | | biological regulation | |  |
| 82 | 18.965 | 18.166 | 18.311 | Matrisome-associated | ECM Regulators | Cluster -163 | sp|P01008|ANT3_HUMAN | Antithrombin-III | GO:CC extracellular matrix (GO:0005576, GO:0005578 & GO:0031012) & GO:CC not extracellular matrix | SERPINC1 | regulation of protein metabolic process | | proteolysis | | | biological regulation | |  |
| 83 | 17.991 | 17.623 | 19.308 | Core matrisome | ECM Glycoproteins | Cluster -163 | sp|P09486|SPRC_HUMAN | SPARC | GO:CC extracellular matrix (GO:0005576, GO:0005578 & GO:0031012) & GO:CC not extracellular matrix | SPARC | regulation of protein metabolic process | | proteolysis | | | biological regulation | |  |
| 84 | 18.34 | 18.386 | 19.151 | Matrisome-associated | ECM Regulators | Cluster -163 | sp|O15460|P4HA2_HUMAN | Prolyl 4-hydroxylase subunit alpha-2 | GO:CC not extracellular matrix | P4HA2 | regulation of protein metabolic process | | proteolysis | | | biological regulation | |  |
| 85 | 17.598 | 18.186 | 18.75 | Core matrisome | Proteoglycans | Cluster -163 | sp|P10915|HPLN1_HUMAN | Hyaluronan and proteoglycan link protein 1 | GO:CC extracellular matrix (GO:0005576, GO:0005578 & GO:0031012) & GO:CC not extracellular matrix | HAPLN1 | regulation of protein metabolic process | | proteolysis | | | biological regulation | |  |
| 86 | 18.011 | 18.1 | 19.006 | Matrisome-associated | ECM Regulators | Cluster -163 | sp|P01042|KNG1_HUMAN | Kininogen-1 | GO:CC extracellular matrix (GO:0005576, GO:0005578 & GO:0031012) & GO:CC not extracellular matrix | KNG1 | regulation of protein metabolic process | | proteolysis | | | biological regulation | |  |
| 87 | 18.003 | 18.252 | 18.713 | Core matrisome | ECM Glycoproteins | Cluster -163 | sp|Q96CG8|CTHR1_HUMAN | Collagen triple helix repeat-containing protein 1 | GO:CC extracellular matrix (GO:0005576, GO:0005578 & GO:0031012) & GO:CC not extracellular matrix | CTHRC1 | regulation of protein metabolic process | | proteolysis | | | biological regulation | |  |
| 88 | 19.656 | 18.69 | 14.734 | Matrisome-associated | ECM Regulators | Cluster -165 | sp|Q92743|HTRA1_HUMAN | Serine protease HTRA1 | GO:CC extracellular matrix (GO:0005576, GO:0005578 & GO:0031012) & GO:CC not extracellular matrix | HTRA1 | response to growth factor | | | response to endogenous stimulus | | |  | |
| 89 | 18.986 | 19.103 | 14.17 | Core matrisome | ECM Glycoproteins | Cluster -165 | sp|P55001|MFAP2_HUMAN | Microfibrillar-associated protein 2 | GO:CC extracellular matrix (GO:0005576, GO:0005578 & GO:0031012) & GO:CC not extracellular matrix | MFAP2 | response to growth factor | | | response to endogenous stimulus | | |  | |
| 90 | 18.831 | 18.384 | 14.316 | Core matrisome | ECM Glycoproteins | Cluster -165 | sp|Q9Y6C2|EMIL1_HUMAN | EMILIN-1 | GO:CC extracellular matrix (GO:0005576, GO:0005578 & GO:0031012) & GO:CC not extracellular matrix | EMILIN1 | response to growth factor | | | response to endogenous stimulus | | |  | |
| 91 | 18.696 | 18.758 | 14.471 | Core matrisome | ECM Glycoproteins | Cluster -165 | sp|P24821|TENA_HUMAN | Tenascin | GO:CC extracellular matrix (GO:0005576, GO:0005578 & GO:0031012) & GO:CC not extracellular matrix | TNC | response to growth factor | | | response to endogenous stimulus | | |  | |
| 92 | 18.469 | 17.926 | 13.796 | Core matrisome | ECM Glycoproteins | Cluster -165 | sp|P98095|FBLN2_HUMAN | Fibulin-2 | GO:CC extracellular matrix (GO:0005576, GO:0005578 & GO:0031012) & GO:CC not extracellular matrix | FBLN2 | response to growth factor | | | response to endogenous stimulus | | |  | |
| 93 | 17.668 | 17.411 | 13.745 | Core matrisome | ECM Glycoproteins | Cluster -165 | sp|Q96RW7|HMCN1_HUMAN | Hemicentin-1 | GO:CC not extracellular matrix | HMCN1 | response to growth factor | | | response to endogenous stimulus | | |  | |
| 94 | 16.924 | 15.743 | 18.19 | Core matrisome | Collagens | Cluster -110 | sp|P08123|CO1A2_HUMAN | Collagen alpha-2(I) chain | GO:CC extracellular matrix (GO:0005576, GO:0005578 & GO:0031012) & GO:CC not extracellular matrix | COL1A2 | extracellular matrix organization | | collagen fibril organization | | | skin morphogenesis | |  |
| 95 | 17.169 | 16.036 | 18.796 | Core matrisome | Collagens | Cluster -110 | sp|P02452|CO1A1_HUMAN | Collagen alpha-1(I) chain | GO:CC extracellular matrix (GO:0005576, GO:0005578 & GO:0031012) & GO:CC not extracellular matrix | COL1A1 | extracellular matrix organization | | collagen fibril organization | | | skin morphogenesis | |  |
| 96 | 17.652 | 15.633 | 14.413 | Matrisome-associated | Secreted Factors | Cluster -166 | sp|P05109|S10A8_HUMAN | Protein S100-A8 | GO:CC extracellular matrix (GO:0005576, GO:0005578 & GO:0031012) & GO:CC not extracellular matrix | S100A8 | extracellular matrix organization | | | response to oxygen-containing compound | | | circulatory system development | |
| 97 | 17.401 | 17.825 | 14.662 | Core matrisome | ECM Glycoproteins | Cluster -166 | sp|O95967|FBLN4_HUMAN | EGF-containing fibulin-like extracellular matrix protein 2 | GO:CC extracellular matrix (GO:0005576, GO:0005578 & GO:0031012) & GO:CC not extracellular matrix | EFEMP2 | extracellular matrix organization | | | response to oxygen-containing compound | | | circulatory system development | |
| 98 | 17.03 | 17.412 | 14.98 | Core matrisome | ECM Glycoproteins | Cluster -166 | sp|Q14112|NID2_HUMAN | Nidogen-2 | GO:CC extracellular matrix (GO:0005576, GO:0005578 & GO:0031012) & GO:CC not extracellular matrix | NID2 | extracellular matrix organization | | | response to oxygen-containing compound | | | circulatory system development | |
| 99 | 16.839 | 17.667 | 14.976 | Matrisome-associated | ECM Regulators | Cluster -166 | sp|P08253|MMP2_HUMAN | 72 kDa type IV collagenase | GO:CC extracellular matrix (GO:0005576, GO:0005578 & GO:0031012) & GO:CC not extracellular matrix | MMP2 | extracellular matrix organization | | | response to oxygen-containing compound | | | circulatory system development | |
| 100 | 17.073 | 17.008 | 15.297 | Matrisome-associated | ECM Regulators | Cluster -166 | sp|P36955|PEDF_HUMAN | Pigment epithelium-derived factor | GO:CC extracellular matrix (GO:0005576, GO:0005578 & GO:0031012) & GO:CC not extracellular matrix | SERPINF1 | extracellular matrix organization | | | response to oxygen-containing compound | | | circulatory system development | |
| 101 | 16.62 | 17.209 | 15.296 | Matrisome-associated | ECM Regulators | Cluster -166 | sp|Q08397|LOXL1_HUMAN | Lysyl oxidase homolog 1 | GO:CC extracellular matrix (GO:0005576, GO:0005578 & GO:0031012) & GO:CC not extracellular matrix | LOXL1 | extracellular matrix organization | | | response to oxygen-containing compound | | | circulatory system development | |
| 102 | 16.879 | 17.227 | 15.297 | Core matrisome | ECM Glycoproteins | Cluster -166 | sp|P35555|FBN1_HUMAN | Fibrillin-1 | GO:CC extracellular matrix (GO:0005576, GO:0005578 & GO:0031012) & GO:CC not extracellular matrix | FBN1 | extracellular matrix organization | | | response to oxygen-containing compound | | | circulatory system development | |
| 103 | 17.517 | 16.679 | 14.912 | Core matrisome | ECM Glycoproteins | Cluster -166 | sp|Q6ZMP0|THSD4_HUMAN | Thrombospondin type-1 domain-containing protein 4 | GO:CC not extracellular matrix | THSD4 | extracellular matrix organization | | | response to oxygen-containing compound | | | circulatory system development | |
| 104 | 17.25 | 16.739 | 15.067 | Matrisome-associated | ECM-affiliated Proteins | Cluster -166 | sp|O00182|LEG9_HUMAN | Galectin-9 | GO:CC not extracellular matrix | LGALS9 | extracellular matrix organization | | | response to oxygen-containing compound | | | circulatory system development | |
| 105 | 17.174 | 16.603 | 15.241 | Core matrisome | Collagens | Cluster -166 | sp|P39060|COIA1_HUMAN | Collagen alpha-1(XVIII) chain | GO:CC extracellular matrix (GO:0005576, GO:0005578 & GO:0031012) & GO:CC not extracellular matrix | COL18A1 | extracellular matrix organization | | | response to oxygen-containing compound | | | circulatory system development | |
| 106 | 17.086 | 16.573 | 14.226 | Core matrisome | Collagens | Cluster -166 | sp|P39059|COFA1_HUMAN | Collagen alpha-1(XV) chain | GO:CC extracellular matrix (GO:0005576, GO:0005578 & GO:0031012) & GO:CC not extracellular matrix | COL15A1 | extracellular matrix organization | | | response to oxygen-containing compound | | | circulatory system development | |
| 107 | 16.159 | 16.722 | 13.987 | Core matrisome | ECM Glycoproteins | Cluster -166 | sp|P35442|TSP2_HUMAN | Thrombospondin-2 | GO:CC extracellular matrix (GO:0005576, GO:0005578 & GO:0031012) & GO:CC not extracellular matrix | THBS2 | extracellular matrix organization | | | response to oxygen-containing compound | | | circulatory system development | |
| 108 | 15.946 | 16.79 | 13.847 | Core matrisome | ECM Glycoproteins | Cluster -166 | sp|O00339|MATN2_HUMAN | Matrilin-2 | GO:CC extracellular matrix (GO:0005576, GO:0005578 & GO:0031012) & GO:CC not extracellular matrix | MATN2 | extracellular matrix organization | | | response to oxygen-containing compound | | | circulatory system development | |
| 109 | 16.487 | 17.383 | 14.059 | Matrisome-associated | Secreted Factors | Cluster -166 | sp|Q9UKU9|ANGL2_HUMAN | Angiopoietin-related protein 2 | GO:CC not extracellular matrix | ANGPTL2 | extracellular matrix organization | | | response to oxygen-containing compound | | | circulatory system development | |
| 110 | 16.444 | 16.005 | 14.803 | Core matrisome | Collagens | Cluster -166 | sp|Q05707|COEA1_HUMAN | Collagen alpha-1(XIV) chain | GO:CC extracellular matrix (GO:0005576, GO:0005578 & GO:0031012) & GO:CC not extracellular matrix | COL14A1 | extracellular matrix organization | | | response to oxygen-containing compound | | | circulatory system development | |
| 111 | 15.868 | 16.041 | 14.764 | Core matrisome | ECM Glycoproteins | Cluster -166 | sp|Q6PCB0|VWA1_HUMAN | von Willebrand factor A domain-containing protein 1 | GO:CC not extracellular matrix | VWA1 | extracellular matrix organization | | | response to oxygen-containing compound | | | circulatory system development | |
| 112 | 15.975 | 16.439 | 14.556 | Matrisome-associated | ECM-affiliated Proteins | Cluster -166 | sp|Q92478|CLC2B_HUMAN | C-type lectin domain family 2 member B | GO:CC not extracellular matrix | CLEC2B | extracellular matrix organization | | | response to oxygen-containing compound | | | circulatory system development | |
| 113 | 16.468 | 16.69 | 14.854 | Core matrisome | ECM Glycoproteins | Cluster -166 | sp|P78539|SRPX_HUMAN | Sushi repeat-containing protein SRPX | GO:CC not extracellular matrix | SRPX | extracellular matrix organization | | | response to oxygen-containing compound | | | circulatory system development | |
| 114 | 16.228 | 16.548 | 15.048 | Core matrisome | ECM Glycoproteins | Cluster -166 | sp|O00468|AGRIN_HUMAN | Agrin | GO:CC extracellular matrix (GO:0005576, GO:0005578 & GO:0031012) & GO:CC not extracellular matrix | AGRN | extracellular matrix organization | | | response to oxygen-containing compound | | | circulatory system development | |
| 115 | 15.828 | 15.624 | 16.762 | Matrisome-associated | ECM Regulators | Cluster -154 | sp|Q13443|ADAM9_HUMAN | Disintegrin and metalloproteinase domain-containing protein 9 | GO:CC not extracellular matrix | ADAM9 | single-organism process | | response to stimulus | | | anatomical structure development | | cell differentiation |
| 116 | 16.441 | 16.258 | 17.208 | Matrisome-associated | ECM Regulators | Cluster -154 | sp|P05543|THBG_HUMAN | Thyroxine-binding globulin | GO:CC extracellular matrix (GO:0005576, GO:0005578 & GO:0031012) & GO:CC not extracellular matrix | SERPINA7 | single-organism process | | response to stimulus | | | anatomical structure development | | cell differentiation |
| 117 | 16.425 | 16.476 | 17.038 | Core matrisome | Collagens | Cluster -154 | sp|P05997|CO5A2_HUMAN | Collagen alpha-2(V) chain | GO:CC extracellular matrix (GO:0005576, GO:0005578 & GO:0031012) & GO:CC not extracellular matrix | COL5A2 | single-organism process | | response to stimulus | | | anatomical structure development | | cell differentiation |
| 118 | 16.763 | 17.045 | 16.711 | Matrisome-associated | ECM Regulators | Cluster -154 | sp|P05155|IC1_HUMAN | Plasma protease C1 inhibitor | GO:CC extracellular matrix (GO:0005576, GO:0005578 & GO:0031012) & GO:CC not extracellular matrix | SERPING1 | single-organism process | | response to stimulus | | | anatomical structure development | | cell differentiation |
| 119 | 16.768 | 16.761 | 16.866 | Core matrisome | Collagens | Cluster -154 | sp|Q02388|CO7A1_HUMAN | Collagen alpha-1(VII) chain | GO:CC extracellular matrix (GO:0005576, GO:0005578 & GO:0031012) & GO:CC not extracellular matrix | COL7A1 | single-organism process | | response to stimulus | | | anatomical structure development | | cell differentiation |
| 120 | 16.385 | 16.808 | 16.949 | Core matrisome | Collagens | Cluster -154 | sp|P08572|CO4A2_HUMAN | Collagen alpha-2(IV) chain | GO:CC extracellular matrix (GO:0005576, GO:0005578 & GO:0031012) & GO:CC not extracellular matrix | COL4A2 | single-organism process | | response to stimulus | | | anatomical structure development | | cell differentiation |
| 121 | 16.979 | 16.13 | 16.675 | Core matrisome | Collagens | Cluster -154 | sp|P02461|CO3A1_HUMAN | Collagen alpha-1(III) chain | GO:CC extracellular matrix (GO:0005576, GO:0005578 & GO:0031012) & GO:CC not extracellular matrix | COL3A1 | single-organism process | | response to stimulus | | | anatomical structure development | | cell differentiation |
| 122 | 16.784 | 16.444 | 16.324 | Matrisome-associated | ECM-affiliated Proteins | Cluster -154 | sp|P18827|SDC1_HUMAN | Syndecan-1 | GO:CC not extracellular matrix | SDC1 | single-organism process | | response to stimulus | | | anatomical structure development | | cell differentiation |
| 123 | 16.372 | 16.415 | 16.58 | Matrisome-associated | ECM Regulators | Cluster -154 | sp|P50453|SPB9_HUMAN | Serpin B9 | GO:CC not extracellular matrix | SERPINB9 | single-organism process | | response to stimulus | | | anatomical structure development | | cell differentiation |
| 124 | 15.969 | 15.813 | 15.529 | Core matrisome | ECM Glycoproteins | Cluster -154 | sp|P24043|LAMA2_HUMAN | Laminin subunit alpha-2 | GO:CC extracellular matrix (GO:0005576, GO:0005578 & GO:0031012) & GO:CC not extracellular matrix | LAMA2 | single-organism process | | response to stimulus | | | anatomical structure development | | cell differentiation |
| 125 | 16.068 | 16.291 | 16.123 | Core matrisome | ECM Glycoproteins | Cluster -154 | sp|P49746|TSP3_HUMAN | Thrombospondin-3 | GO:CC extracellular matrix (GO:0005576, GO:0005578 & GO:0031012) & GO:CC not extracellular matrix | THBS3 | single-organism process | | response to stimulus | | | anatomical structure development | | cell differentiation |
| 126 | 15.777 | 16.285 | 16.022 | Core matrisome | ECM Glycoproteins | Cluster -154 | sp|Q9UBX5|FBLN5_HUMAN | Fibulin-5 | GO:CC extracellular matrix (GO:0005576, GO:0005578 & GO:0031012) & GO:CC not extracellular matrix | FBLN5 | single-organism process | | response to stimulus | | | anatomical structure development | | cell differentiation |
| 127 | 15.883 | 16.283 | 16.038 | Core matrisome | ECM Glycoproteins | Cluster -154 | sp|Q9UGM3|DMBT1_HUMAN | Deleted in malignant brain tumors 1 protein | GO:CC extracellular matrix (GO:0005576, GO:0005578 & GO:0031012) & GO:CC not extracellular matrix | DMBT1 | single-organism process | | response to stimulus | | | anatomical structure development | | cell differentiation |
| 128 | 15.94 | 16.429 | 16.322 | Matrisome-associated | ECM-affiliated Proteins | Cluster -154 | sp|P05452|TETN_HUMAN | Tetranectin | GO:CC extracellular matrix (GO:0005576, GO:0005578 & GO:0031012) & GO:CC not extracellular matrix | CLEC3B | single-organism process | | response to stimulus | | | anatomical structure development | | cell differentiation |
| 129 | 16.579 | 16.896 | 16.055 | Core matrisome | ECM Glycoproteins | Cluster -154 | sp|Q14767|LTBP2_HUMAN | Latent-transforming growth factor beta-binding protein 2 | GO:CC extracellular matrix (GO:0005576, GO:0005578 & GO:0031012) & GO:CC not extracellular matrix | LTBP2 | single-organism process | | response to stimulus | | | anatomical structure development | | cell differentiation |
| 130 | 16.745 | 17.128 | 16.212 | Core matrisome | ECM Glycoproteins | Cluster -154 | sp|Q12805|FBLN3_HUMAN | EGF-containing fibulin-like extracellular matrix protein 1 | GO:CC extracellular matrix (GO:0005576, GO:0005578 & GO:0031012) & GO:CC not extracellular matrix | EFEMP1 | single-organism process | | response to stimulus | | | anatomical structure development | | cell differentiation |
| 131 | 16.395 | 16.605 | 15.879 | Core matrisome | ECM Glycoproteins | Cluster -154 | sp|Q15063|POSTN_HUMAN | Periostin | GO:CC extracellular matrix (GO:0005576, GO:0005578 & GO:0031012) & GO:CC not extracellular matrix | POSTN | single-organism process | | response to stimulus | | | anatomical structure development | | cell differentiation |
| 132 | 16.512 | 16.88 | 15.572 | Core matrisome | Collagens | Cluster -154 | sp|P25940|CO5A3_HUMAN | Collagen alpha-3(V) chain | GO:CC extracellular matrix (GO:0005576, GO:0005578 & GO:0031012) & GO:CC not extracellular matrix | COL5A3 | single-organism process | | response to stimulus | | | anatomical structure development | | cell differentiation |
| 133 | 16.078 | 17.09 | 16.558 | Core matrisome | ECM Glycoproteins | Cluster -154 | sp|Q7Z7G0|TARSH_HUMAN | Target of Nesh-SH3 | GO:CC extracellular matrix (GO:0005576, GO:0005578 & GO:0031012) & GO:CC not extracellular matrix | ABI3BP | single-organism process | | response to stimulus | | | anatomical structure development | | cell differentiation |
| 134 | 18.887 | 16.282 | 16.287 | Matrisome-associated | Secreted Factors | Cluster 152 | sp|P06702|S10A9_HUMAN | Protein S100-A9 | GO:CC extracellular matrix (GO:0005576, GO:0005578 & GO:0031012) & GO:CC not extracellular matrix | S100A9 | calcium ion binding | | | microtubule binding | | | Diseases of Immune System | |
| 135 | 17.508 | 16.222 | 17.341 | Core matrisome | Collagens | Cluster -161 | sp|P02458|CO2A1_HUMAN | Collagen alpha-1(II) chain | GO:CC extracellular matrix (GO:0005576, GO:0005578 & GO:0031012) & GO:CC not extracellular matrix | COL2A1 | single-multicellular organism process | | regulation of biological process | | | protein binding | |  |
| 136 | 18.227 | 16.525 | 17.625 | Core matrisome | ECM Glycoproteins | Cluster -161 | sp|P02671|FIBA_HUMAN | Fibrinogen alpha chain | GO:CC extracellular matrix (GO:0005576, GO:0005578 & GO:0031012) & GO:CC not extracellular matrix | FGA | single-multicellular organism process | | regulation of biological process | | | protein binding | |  |
| 137 | 18.549 | 16.595 | 17.948 | Core matrisome | ECM Glycoproteins | Cluster -161 | sp|P02675|FIBB_HUMAN | Fibrinogen beta chain | GO:CC extracellular matrix (GO:0005576, GO:0005578 & GO:0031012) & GO:CC not extracellular matrix | FGB | single-multicellular organism process | | regulation of biological process | | | protein binding | |  |
| 138 | 18.774 | 16.39 | 18.211 | Matrisome-associated | ECM Regulators | Cluster -161 | sp|P00488|F13A_HUMAN | Coagulation factor XIII A chain | GO:CC extracellular matrix (GO:0005576, GO:0005578 & GO:0031012) & GO:CC not extracellular matrix | F13A1 | single-multicellular organism process | | regulation of biological process | | | protein binding | |  |
| 139 | 18 | 17.804 | 18.274 | Matrisome-associated | ECM Regulators | Cluster -161 | sp|P01034|CYTC_HUMAN | Cystatin-C | GO:CC extracellular matrix (GO:0005576, GO:0005578 & GO:0031012) & GO:CC not extracellular matrix | CST3 | single-multicellular organism process | | regulation of biological process | | | protein binding | |  |
| 140 | 17.727 | 17.347 | 17.94 | Core matrisome | ECM Glycoproteins | Cluster -161 | sp|Q15113|PCOC1_HUMAN | Procollagen C-endopeptidase enhancer 1 | GO:CC extracellular matrix (GO:0005576, GO:0005578 & GO:0031012) & GO:CC not extracellular matrix | PCOLCE | single-multicellular organism process | | regulation of biological process | | | protein binding | |  |
| 141 | 17.386 | 17.566 | 18.429 | Matrisome-associated | ECM Regulators | Cluster -161 | sp|P05120|PAI2_HUMAN | Plasminogen activator inhibitor 2 | GO:CC extracellular matrix (GO:0005576, GO:0005578 & GO:0031012) & GO:CC not extracellular matrix | SERPINB2 | single-multicellular organism process | | regulation of biological process | | | protein binding | |  |
| 142 | 17.369 | 17.416 | 18.229 | Core matrisome | Collagens | Cluster -161 | sp|P20908|CO5A1_HUMAN | Collagen alpha-1(V) chain | GO:CC extracellular matrix (GO:0005576, GO:0005578 & GO:0031012) & GO:CC not extracellular matrix | COL5A1 | single-multicellular organism process | | regulation of biological process | | | protein binding | |  |
| 143 | 16.679 | 16.827 | 17.579 | Core matrisome | ECM Glycoproteins | Cluster -161 | sp|P35556|FBN2_HUMAN | Fibrillin-2 | GO:CC extracellular matrix (GO:0005576, GO:0005578 & GO:0031012) & GO:CC not extracellular matrix | FBN2 | single-multicellular organism process | | regulation of biological process | | | protein binding | |  |
| 144 | 16.818 | 17.455 | 18.053 | Matrisome-associated | ECM-affiliated Proteins | Cluster -161 | sp|P31431|SDC4_HUMAN | Syndecan-4 | GO:CC not extracellular matrix | SDC4 | single-multicellular organism process | | regulation of biological process | | | protein binding | |  |
| 145 | 16.959 | 17.033 | 18.229 | Matrisome-associated | ECM Regulators | Cluster -161 | sp|P02760|AMBP_HUMAN | Protein AMBP | GO:CC extracellular matrix (GO:0005576, GO:0005578 & GO:0031012) & GO:CC not extracellular matrix | AMBP | single-multicellular organism process | | regulation of biological process | | | protein binding | |  |
| 146 | 17.779 | 17.127 | 17.407 | Matrisome-associated | ECM Regulators | Cluster -161 | sp|P01040|CYTA_HUMAN | Cystatin-A | GO:CC not extracellular matrix | CSTA | single-multicellular organism process | | regulation of biological process | | | protein binding | |  |
| 147 | 17.528 | 17.757 | 17.22 | Matrisome-associated | ECM-affiliated Proteins | Cluster -161 | sp|O15031|PLXB2_HUMAN | Plexin-B2 | GO:CC not extracellular matrix | PLXNB2 | single-multicellular organism process | | regulation of biological process | | | protein binding | |  |
| 148 | 18.113 | 17.503 | 17.703 | Matrisome-associated | ECM-affiliated Proteins | Cluster -161 | sp|Q5KU26|COL12_HUMAN | Collectin-12 | GO:CC not extracellular matrix | COLEC12 | single-multicellular organism process | | regulation of biological process | | | protein binding | |  |
| 149 | 17.848 | 17.845 | 17.576 | Matrisome-associated | Secreted Factors | Cluster -161 | sp|P01137|TGFB1_HUMAN | Transforming growth factor beta-1 proprotein | GO:CC extracellular matrix (GO:0005576, GO:0005578 & GO:0031012) & GO:CC not extracellular matrix | TGFB1 | single-multicellular organism process | | regulation of biological process | | | protein binding | |  |
| 150 | 17.883 | 17.84 | 17.679 | Matrisome-associated | ECM Regulators | Cluster -161 | sp|P19823|ITIH2_HUMAN | Inter-alpha-trypsin inhibitor heavy chain H2 | GO:CC extracellular matrix (GO:0005576, GO:0005578 & GO:0031012) & GO:CC not extracellular matrix | ITIH2 | single-multicellular organism process | | regulation of biological process | | | protein binding | |  |
| 151 | 17.771 | 17.558 | 17.445 | Matrisome-associated | ECM Regulators | Cluster -161 | sp|Q9NXG6|P4HTM_HUMAN | Transmembrane prolyl 4-hydroxylase | GO:CC not extracellular matrix | P4HTM | single-multicellular organism process | | regulation of biological process | | | protein binding | |  |
| 152 | 17.796 | 17.49 | 17.532 | Core matrisome | ECM Glycoproteins | Cluster -161 | sp|P11047|LAMC1_HUMAN | Laminin subunit gamma-1 | GO:CC extracellular matrix (GO:0005576, GO:0005578 & GO:0031012) & GO:CC not extracellular matrix | LAMC1 | single-multicellular organism process | | regulation of biological process | | | protein binding | |  |
| 153 | 17.689 | 17.709 | 17.643 | Matrisome-associated | Secreted Factors | Cluster -161 | sp|P51610|HCFC1_HUMAN | Host cell factor 1 | GO:CC not extracellular matrix | HCFC1 | single-multicellular organism process | | regulation of biological process | | | protein binding | |  |
| 154 | 17.732 | 17.68 | 17.545 | Core matrisome | ECM Glycoproteins | Cluster -161 | sp|Q96HD1|CREL1_HUMAN | Protein disulfide isomerase CRELD1 | GO:CC not extracellular matrix | CRELD1 | single-multicellular organism process | | regulation of biological process | | | protein binding | |  |
| 155 | 17.777 | 17.715 | 17.458 | Matrisome-associated | ECM-affiliated Proteins | Cluster -161 | sp|P20073|ANXA7_HUMAN | Annexin A7 | GO:CC not extracellular matrix | ANXA7 | single-multicellular organism process | | regulation of biological process | | | protein binding | |  |
| 156 | 17.058 | 17.124 | 17.048 | Matrisome-associated | Secreted Factors | Cluster -161 | sp|P29034|S10A2_HUMAN | Protein S100-A2 | GO:CC not extracellular matrix | S100A2 | single-multicellular organism process | | regulation of biological process | | | protein binding | |  |
| 157 | 17.344 | 17.156 | 17.079 | Core matrisome | ECM Glycoproteins | Cluster -161 | sp|Q16610|ECM1_HUMAN | Extracellular matrix protein 1 | GO:CC extracellular matrix (GO:0005576, GO:0005578 & GO:0031012) & GO:CC not extracellular matrix | ECM1 | single-multicellular organism process | | regulation of biological process | | | protein binding | |  |
| 158 | 17.207 | 17.179 | 17.519 | Core matrisome | ECM Glycoproteins | Cluster -161 | sp|Q92626|PXDN_HUMAN | Peroxidasin homolog | GO:CC extracellular matrix (GO:0005576, GO:0005578 & GO:0031012) & GO:CC not extracellular matrix | PXDN | single-multicellular organism process | | regulation of biological process | | | protein binding | |  |
| 159 | 17.087 | 17.214 | 17.567 | Matrisome-associated | Secreted Factors | Cluster -161 | sp|Q8IUI8|CRLF3_HUMAN | Cytokine receptor-like factor 3 | GO:CC not extracellular matrix | CRLF3 | single-multicellular organism process | | regulation of biological process | | | protein binding | |  |
| 160 | 17.039 | 17.333 | 17.543 | Core matrisome | Collagens | Cluster -161 | sp|P27658|CO8A1_HUMAN | Collagen alpha-1(VIII) chain | GO:CC extracellular matrix (GO:0005576, GO:0005578 & GO:0031012) & GO:CC not extracellular matrix | COL8A1 | single-multicellular organism process | | regulation of biological process | | | protein binding | |  |
| 161 | 17.477 | 17.425 | 17.525 | Core matrisome | ECM Glycoproteins | Cluster -161 | sp|Q16270|IBP7_HUMAN | Insulin-like growth factor-binding protein 7 | GO:CC extracellular matrix (GO:0005576, GO:0005578 & GO:0031012) & GO:CC not extracellular matrix | IGFBP7 | single-multicellular organism process | | regulation of biological process | | | protein binding | |  |
| 162 | 17.356 | 17.481 | 17.435 | Matrisome-associated | ECM-affiliated Proteins | Cluster -161 | sp|P12429|ANXA3_HUMAN | Annexin A3 | GO:CC not extracellular matrix | ANXA3 | single-multicellular organism process | | regulation of biological process | | | protein binding | |  |
| 163 | 17.675 | 17.756 | 16.621 | Core matrisome | Proteoglycans | Cluster -161 | sp|P20774|MIME_HUMAN | Mimecan | GO:CC extracellular matrix (GO:0005576, GO:0005578 & GO:0031012) & GO:CC not extracellular matrix | OGN | single-multicellular organism process | | regulation of biological process | | | protein binding | |  |
| 164 | 17.394 | 17.823 | 16.868 | Core matrisome | ECM Glycoproteins | Cluster -161 | sp|P55268|LAMB2_HUMAN | Laminin subunit beta-2 | GO:CC extracellular matrix (GO:0005576, GO:0005578 & GO:0031012) & GO:CC not extracellular matrix | LAMB2 | single-multicellular organism process | | regulation of biological process | | | protein binding | |  |
| 165 | 18.166 | 17.734 | 16.652 | Matrisome-associated | ECM-affiliated Proteins | Cluster -161 | sp|O60565|GREM1_HUMAN | Gremlin-1 | GO:CC not extracellular matrix | GREM1 | single-multicellular organism process | | regulation of biological process | | | protein binding | |  |
| 166 | 18.078 | 17.777 | 17.013 | Matrisome-associated | ECM Regulators | Cluster -161 | sp|P78536|ADA17_HUMAN | Disintegrin and metalloproteinase domain-containing protein 17 | GO:CC not extracellular matrix | ADAM17 | single-multicellular organism process | | regulation of biological process | | | protein binding | |  |
| 167 | 17.777 | 17.631 | 16.966 | Matrisome-associated | ECM Regulators | Cluster -161 | sp|P53634|CATC_HUMAN | Dipeptidyl peptidase 1 | GO:CC extracellular matrix (GO:0005576, GO:0005578 & GO:0031012) & GO:CC not extracellular matrix | CTSC | single-multicellular organism process | | regulation of biological process | | | protein binding | |  |
| 168 | 17.87 | 17.509 | 16.88 | Matrisome-associated | ECM Regulators | Cluster -161 | sp|O14672|ADA10_HUMAN | Disintegrin and metalloproteinase domain-containing protein 10 | GO:CC not extracellular matrix | ADAM10 | single-multicellular organism process | | regulation of biological process | | | protein binding | |  |
| 169 | 18.455 | 18.11 | 15.078 | Matrisome-associated | ECM-affiliated Proteins | Cluster -147 | sp|Q6UX71|PXDC2_HUMAN | Plexin domain-containing protein 2 | GO:CC not extracellular matrix | PLXDC2 | multicellular organismal process | | | anatomical structure development | | | extracellular matrix structural constituent | |
| 170 | 18.373 | 18.333 | 15.456 | Core matrisome | ECM Glycoproteins | Cluster -147 | sp|Q9BXX0|EMIL2_HUMAN | EMILIN-2 | GO:CC extracellular matrix (GO:0005576, GO:0005578 & GO:0031012) & GO:CC not extracellular matrix | EMILIN2 | multicellular organismal process | | | anatomical structure development | | | extracellular matrix structural constituent | |
| 171 | 17.887 | 18.351 | 16.32 | Core matrisome | Proteoglycans | Cluster -147 | sp|P13611|CSPG2_HUMAN | Versican core protein | GO:CC extracellular matrix (GO:0005576, GO:0005578 & GO:0031012) & GO:CC not extracellular matrix | VCAN | multicellular organismal process | | | anatomical structure development | | | extracellular matrix structural constituent | |
| 172 | 17.671 | 18.225 | 16.008 | Core matrisome | ECM Glycoproteins | Cluster -147 | sp|Q9H0B8|CRLD2_HUMAN | Cysteine-rich secretory protein LCCL domain-containing 2 | GO:CC extracellular matrix (GO:0005576, GO:0005578 & GO:0031012) & GO:CC not extracellular matrix | CRISPLD2 | multicellular organismal process | | | anatomical structure development | | | extracellular matrix structural constituent | |
| 173 | 17.693 | 17.658 | 15.469 | Core matrisome | ECM Glycoproteins | Cluster -147 | sp|Q9NR99|MXRA5_HUMAN | Matrix-remodeling-associated protein 5 | GO:CC extracellular matrix (GO:0005576, GO:0005578 & GO:0031012) & GO:CC not extracellular matrix | MXRA5 | multicellular organismal process | | | anatomical structure development | | | extracellular matrix structural constituent | |
| 174 | 17.983 | 17.787 | 15.426 | Core matrisome | ECM Glycoproteins | Cluster -147 | sp|O00534|VMA5A_HUMAN | von Willebrand factor A domain-containing protein 5A | GO:CC not extracellular matrix | VWA5A | multicellular organismal process | | | anatomical structure development | | | extracellular matrix structural constituent | |
| 175 | 17.557 | 18.136 | 15.568 | Core matrisome | ECM Glycoproteins | Cluster -147 | sp|Q16363|LAMA4_HUMAN | Laminin subunit alpha-4 | GO:CC extracellular matrix (GO:0005576, GO:0005578 & GO:0031012) & GO:CC not extracellular matrix | LAMA4 | multicellular organismal process | | | anatomical structure development | | | extracellular matrix structural constituent | |
| 176 | 17.772 | 18.046 | 15.454 | Core matrisome | Proteoglycans | Cluster -147 | sp|P98160|PGBM_HUMAN | Basement membrane-specific heparan sulfate proteoglycan core protein | GO:CC extracellular matrix (GO:0005576, GO:0005578 & GO:0031012) & GO:CC not extracellular matrix | HSPG2 | multicellular organismal process | | | anatomical structure development | | | extracellular matrix structural constituent | |
| 177 | 17.779 | 18.439 | 15.645 | Core matrisome | ECM Glycoproteins | Cluster -147 | sp|Q8IUX7|AEBP1_HUMAN | Adipocyte enhancer-binding protein 1 | GO:CC extracellular matrix (GO:0005576, GO:0005578 & GO:0031012) & GO:CC not extracellular matrix | AEBP1 | multicellular organismal process | | | anatomical structure development | | | extracellular matrix structural constituent | |
| 178 | 17.101 | 17.674 | 15.73 | Core matrisome | ECM Glycoproteins | Cluster -147 | sp|P14543|NID1_HUMAN | Nidogen-1 | GO:CC extracellular matrix (GO:0005576, GO:0005578 & GO:0031012) & GO:CC not extracellular matrix | NID1 | multicellular organismal process | | | anatomical structure development | | | extracellular matrix structural constituent | |
| 179 | 17.519 | 17.331 | 16.251 | Matrisome-associated | ECM Regulators | Cluster -147 | sp|Q6UXH9|PAMR1_HUMAN | Inactive serine protease PAMR1 | GO:CC extracellular matrix (GO:0005576, GO:0005578 & GO:0031012) & GO:CC not extracellular matrix | PAMR1 | multicellular organismal process | | | anatomical structure development | | | extracellular matrix structural constituent | |
| 180 | 17.243 | 17.142 | 16.233 | Core matrisome | ECM Glycoproteins | Cluster -147 | sp|Q14766|LTBP1_HUMAN | Latent-transforming growth factor beta-binding protein 1 | GO:CC extracellular matrix (GO:0005576, GO:0005578 & GO:0031012) & GO:CC not extracellular matrix | LTBP1 | multicellular organismal process | | | anatomical structure development | | | extracellular matrix structural constituent | |
| 181 | 17.294 | 17.227 | 15.89 | Matrisome-associated | ECM Regulators | Cluster -147 | sp|P07711|CATL1_HUMAN | Procathepsin L | GO:CC extracellular matrix (GO:0005576, GO:0005578 & GO:0031012) & GO:CC not extracellular matrix | CTSL | multicellular organismal process | | | anatomical structure development | | | extracellular matrix structural constituent | |
| 182 | 18.099 | 17.273 | 16.296 | Core matrisome | Collagens | Cluster -147 | sp|Q07092|COGA1_HUMAN | Collagen alpha-1(XVI) chain | GO:CC extracellular matrix (GO:0005576, GO:0005578 & GO:0031012) & GO:CC not extracellular matrix | COL16A1 | multicellular organismal process | | | anatomical structure development | | | extracellular matrix structural constituent | |
| 183 | 18.149 | 16.929 | 16.037 | Core matrisome | ECM Glycoproteins | Cluster -147 | sp|O43405|COCH_HUMAN | Cochlin | GO:CC extracellular matrix (GO:0005576, GO:0005578 & GO:0031012) & GO:CC not extracellular matrix | COCH | multicellular organismal process | | | anatomical structure development | | | extracellular matrix structural constituent | |
| 184 | 17.769 | 17.554 | 16.002 | Core matrisome | ECM Glycoproteins | Cluster -147 | sp|Q08431|MFGM_HUMAN | Lactadherin | GO:CC extracellular matrix (GO:0005576, GO:0005578 & GO:0031012) & GO:CC not extracellular matrix | MFGE8 | multicellular organismal process | | | anatomical structure development | | | extracellular matrix structural constituent | |
| 185 | 17.997 | 17.235 | 15.929 | Matrisome-associated | ECM Regulators | Cluster -147 | sp|P25774|CATS_HUMAN | Cathepsin S | GO:CC extracellular matrix (GO:0005576, GO:0005578 & GO:0031012) & GO:CC not extracellular matrix | CTSS | multicellular organismal process | anatomical structure development | | | extracellular matrix structural constituent | | | |
| 186 | 17.794 | 17.317 | 15.937 | Matrisome-associated | ECM Regulators | Cluster -147 | sp|P01023|A2MG_HUMAN | Alpha-2-macroglobulin | GO:CC extracellular matrix (GO:0005576, GO:0005578 & GO:0031012) & GO:CC not extracellular matrix | A2M | multicellular organismal process | anatomical structure development | | | extracellular matrix structural constituent | | | |

# **Supplementary Table S7. Expression abundance of all ECM proteins identified in DPC cultured in 2D and 3D culture platforms expressed proteins with ECM annotation.** The protein abundance values from each sample were log2 transformed and normalized.

| **No.** | **Annotated Gene** | **Annotated  Matrisome  Division** | **Annotated  Matrisome  Category** | **GO term** | **Protein** | **Protein Description** | **2D** | **3D ULA** | **3D PAMCELL** |
| --- | --- | --- | --- | --- | --- | --- | --- | --- | --- |
| 1 | A1BG | Non-matrisome | Non-matrisome | GO:CC extracellular matrix (GO:0005576, GO:0005578 & GO:0031012) & GO:CC not extracellular matrix | sp|P04217|A1BG_HUMAN | Alpha-1B-glycoprotein | 17.81 | 17.34 | 17.05 |
| 2 | A2M | Matrisome-associated | ECM Regulators | GO:CC extracellular matrix (GO:0005576, GO:0005578 & GO:0031012) & GO:CC not extracellular matrix | sp|P01023|A2MG_HUMAN | Alpha-2-macroglobulin | 15.94 | 17.32 | 17.79 |
| 3 | AAAS | Non-matrisome | Non-matrisome | GO:CC not extracellular matrix | sp|Q9NRG9|AAAS_HUMAN | Aladin | 17.79 | 18.07 | 17.94 |
| 4 | AACS | Non-matrisome | Non-matrisome | GO:CC not extracellular matrix | sp|Q86V21|AACS_HUMAN | Acetoacetyl-CoA synthetase | 19.1 | 17.33 | 17.58 |
| 5 | AAK1 | Non-matrisome | Non-matrisome | GO:CC not extracellular matrix | sp|Q2M2I8|AAK1_HUMAN | AP2-associated protein kinase 1 | 17.17 | 17.11 | 17.16 |
| 6 | AAMDC | Non-matrisome | Non-matrisome | GO:CC not extracellular matrix | sp|Q9H7C9|AAMDC_HUMAN | Mth938 domain-containing protein | 18.94 | 18.58 | 18.55 |
| 7 | AAMP | Non-matrisome | Non-matrisome | GO:CC not extracellular matrix | sp|Q13685|AAMP_HUMAN | Angio-associated migratory cell protein | 18.7 | 18.36 | 18.15 |
| 8 | AARS1 | Non-matrisome | Non-matrisome | GO:CC not extracellular matrix | sp|P49588|SYAC_HUMAN | Alanine--tRNA ligase, cytoplasmic | 19.27 | 19 | 18.76 |
| 9 | AARS2 | Non-matrisome | Non-matrisome | GO:CC not extracellular matrix | sp|Q5JTZ9|SYAM_HUMAN | Alanine--tRNA ligase, mitochondrial | 17.12 | 17.29 | 16.88 |
| 10 | AARSD1 | Non-matrisome | Non-matrisome | GO:CC not extracellular matrix | sp|Q9BTE6|AASD1_HUMAN | Alanyl-tRNA editing protein Aarsd1 | 17.42 | 17.21 | 17.11 |
| 11 | AASDHPPT | Non-matrisome | Non-matrisome | GO:CC not extracellular matrix | sp|Q9NRN7|ADPPT_HUMAN | L-aminoadipate-semialdehyde dehydrogenase-phosphopantetheinyl transferase | 17.47 | 17 | 17.15 |
| 12 | AASS | Non-matrisome | Non-matrisome | GO:CC not extracellular matrix | sp|Q9UDR5|AASS_HUMAN | Alpha-aminoadipic semialdehyde synthase, mitochondrial | 17.76 | 18.65 | 18.33 |
| 13 | AATF | Non-matrisome | Non-matrisome | GO:CC not extracellular matrix | sp|Q9NY61|AATF_HUMAN | Protein AATF | 17.06 | 16.6 | 16.45 |
| 14 | ABAT | Non-matrisome | Non-matrisome | GO:CC not extracellular matrix | sp|P80404|GABT_HUMAN | 4-aminobutyrate aminotransferase, mitochondrial | 15.32 | 16.61 | 16.37 |
| 15 | ABCB8 | Non-matrisome | Non-matrisome | GO:CC not extracellular matrix | sp|Q9NUT2|MITOS_HUMAN | Mitochondrial potassium channel ATP-binding subunit | 16.26 | 16.81 | 16.54 |
| 16 | ABCD1 | Non-matrisome | Non-matrisome | GO:CC not extracellular matrix | sp|P33897|ABCD1_HUMAN | ATP-binding cassette sub-family D member 1 | 15.65 | 16.38 | 16.65 |
| 17 | ABCD3 | Non-matrisome | Non-matrisome | GO:CC not extracellular matrix | sp|P28288|ABCD3_HUMAN | ATP-binding cassette sub-family D member 3 | 18.48 | 18.46 | 18.43 |
| 18 | ABCE1 | Non-matrisome | Non-matrisome | GO:CC not extracellular matrix | sp|P61221|ABCE1_HUMAN | ATP-binding cassette sub-family E member 1 | 19.03 | 18.6 | 18.45 |
| 19 | ABCF1 | Non-matrisome | Non-matrisome | GO:CC not extracellular matrix | sp|Q8NE71|ABCF1_HUMAN | ATP-binding cassette sub-family F member 1 | 17.93 | 17.66 | 17.66 |
| 20 | ABCF2 | Non-matrisome | Non-matrisome | GO:CC not extracellular matrix | sp|Q9UG63|ABCF2_HUMAN | ATP-binding cassette sub-family F member 2 | 19.36 | 17.92 | 18.04 |
| 21 | ABCF3 | Non-matrisome | Non-matrisome | GO:CC not extracellular matrix | sp|Q9NUQ8|ABCF3_HUMAN | ATP-binding cassette sub-family F member 3 | 17.87 | 17.83 | 17.86 |
| 22 | ABHD10 | Non-matrisome | Non-matrisome | GO:CC not extracellular matrix | sp|Q9NUJ1|ABHDA_HUMAN | Palmitoyl-protein thioesterase ABHD10, mitochondrial | 17.87 | 18.52 | 18.27 |
| 23 | ABHD11 | Non-matrisome | Non-matrisome | GO:CC not extracellular matrix | sp|Q8NFV4|ABHDB_HUMAN | Protein ABHD11 | 18.39 | 18.42 | 18.4 |
| 24 | ABHD12 | Non-matrisome | Non-matrisome | GO:CC not extracellular matrix | sp|Q8N2K0|ABD12_HUMAN | Lysophosphatidylserine lipase ABHD12 | 17.94 | 18.12 | 17.98 |
| 25 | ABHD14B | Non-matrisome | Non-matrisome | GO:CC not extracellular matrix | sp|Q96IU4|ABHEB_HUMAN | Putative protein-lysine deacylase ABHD14B | 17.75 | 18.04 | 18.07 |
| 26 | ABHD16A | Non-matrisome | Non-matrisome | GO:CC not extracellular matrix | sp|O95870|ABHGA_HUMAN | Phosphatidylserine lipase ABHD16A | 16.83 | 17.39 | 17.4 |
| 27 | ABHD5 | Non-matrisome | Non-matrisome | GO:CC not extracellular matrix | sp|Q8WTS1|ABHD5_HUMAN | 1-acylglycerol-3-phosphate O-acyltransferase ABHD5 | 17.03 | 16.76 | 16.78 |
| 28 | ABI1 | Non-matrisome | Non-matrisome | GO:CC not extracellular matrix | sp|Q8IZP0|ABI1_HUMAN | Abl interactor 1 | 17.86 | 17.92 | 17.73 |
| 29 | ABI3BP | Core matrisome | ECM Glycoproteins | GO:CC extracellular matrix (GO:0005576, GO:0005578 & GO:0031012) & GO:CC not extracellular matrix | sp|Q7Z7G0|TARSH_HUMAN | Target of Nesh-SH3 | 16.56 | 17.09 | 16.08 |
| 30 | ABLIM1 | Non-matrisome | Non-matrisome | GO:CC not extracellular matrix | sp|O14639|ABLM1_HUMAN | Actin-binding LIM protein 1 | 15.91 | 14.59 | 14.83 |
| 31 | ABRACL | Non-matrisome | Non-matrisome | GO:CC not extracellular matrix | sp|Q9P1F3|ABRAL_HUMAN | Costars family protein ABRACL | 21.13 | 20.55 | 21.07 |
| 32 | ABRAXAS2 | Non-matrisome | Non-matrisome | GO:CC not extracellular matrix | sp|Q15018|ABRX2_HUMAN | BRISC complex subunit Abraxas 2 | 16.96 | 17.06 | 17.01 |
| 33 | ACAA1 | Non-matrisome | Non-matrisome | GO:CC extracellular matrix (GO:0005576, GO:0005578 & GO:0031012) & GO:CC not extracellular matrix | sp|P09110|THIK_HUMAN | 3-ketoacyl-CoA thiolase, peroxisomal | 17.44 | 17.87 | 17.83 |
| 34 | ACAA2 | Non-matrisome | Non-matrisome | GO:CC not extracellular matrix | sp|P42765|THIM_HUMAN | 3-ketoacyl-CoA thiolase, mitochondrial | 17.15 | 17.91 | 17.53 |
| 35 | ACACA | Non-matrisome | Non-matrisome | GO:CC not extracellular matrix | sp|Q13085|ACACA_HUMAN | Acetyl-CoA carboxylase 1 | 17.45 | 16.75 | 16.66 |
| 36 | ACAD8 | Non-matrisome | Non-matrisome | GO:CC not extracellular matrix | sp|Q9UKU7|ACAD8_HUMAN | Isobutyryl-CoA dehydrogenase, mitochondrial | 15.52 | 16.31 | 16.35 |
| 37 | ACAD9 | Non-matrisome | Non-matrisome | GO:CC not extracellular matrix | sp|Q9H845|ACAD9_HUMAN | Complex I assembly factor ACAD9, mitochondrial | 17.79 | 18.24 | 18.05 |
| 38 | ACADM | Non-matrisome | Non-matrisome | GO:CC not extracellular matrix | sp|P11310|ACADM_HUMAN | Medium-chain specific acyl-CoA dehydrogenase, mitochondrial | 19.07 | 19.19 | 19.08 |
| 39 | ACADS | Non-matrisome | Non-matrisome | GO:CC not extracellular matrix | sp|P16219|ACADS_HUMAN | Short-chain specific acyl-CoA dehydrogenase, mitochondrial | 16.82 | 17.89 | 17.78 |
| 40 | ACADSB | Non-matrisome | Non-matrisome | GO:CC not extracellular matrix | sp|P45954|ACDSB_HUMAN | Short/branched chain specific acyl-CoA dehydrogenase, mitochondrial | 16.6 | 17.51 | 17.37 |
| 41 | ACADVL | Non-matrisome | Non-matrisome | GO:CC not extracellular matrix | sp|P49748|ACADV_HUMAN | Very long-chain specific acyl-CoA dehydrogenase, mitochondrial | 19.17 | 19.83 | 19.69 |
| 42 | ACAP2 | Non-matrisome | Non-matrisome | GO:CC not extracellular matrix | sp|Q15057|ACAP2_HUMAN | Arf-GAP with coiled-coil, ANK repeat and PH domain-containing protein 2 | 17.32 | 17.78 | 17.87 |
| 43 | ACAT1 | Non-matrisome | Non-matrisome | GO:CC not extracellular matrix | sp|P24752|THIL_HUMAN | Acetyl-CoA acetyltransferase, mitochondrial | 18.77 | 19.38 | 19.14 |
| 44 | ACAT2 | Non-matrisome | Non-matrisome | GO:CC not extracellular matrix | sp|Q9BWD1|THIC_HUMAN | Acetyl-CoA acetyltransferase, cytosolic | 20.2 | 19.72 | 19.36 |
| 45 | ACBD3 | Non-matrisome | Non-matrisome | GO:CC not extracellular matrix | sp|Q9H3P7|GCP60_HUMAN | Golgi resident protein GCP60 | 18 | 17.46 | 17.68 |
| 46 | ACE | Non-matrisome | Non-matrisome | GO:CC extracellular matrix (GO:0005576, GO:0005578 & GO:0031012) & GO:CC not extracellular matrix | sp|P12821|ACE_HUMAN | Angiotensin-converting enzyme | 17.5 | 18.85 | 19.83 |
| 47 | ACIN1 | Non-matrisome | Non-matrisome | GO:CC not extracellular matrix | sp|Q9UKV3|ACINU_HUMAN | Apoptotic chromatin condensation inducer in the nucleus | 18.17 | 18.23 | 18.2 |
| 48 | ACLY | Non-matrisome | Non-matrisome | GO:CC extracellular matrix (GO:0005576, GO:0005578 & GO:0031012) & GO:CC not extracellular matrix | sp|P53396|ACLY_HUMAN | ATP-citrate synthase | 19.92 | 19.51 | 19.3 |
| 49 | ACO1 | Non-matrisome | Non-matrisome | GO:CC not extracellular matrix | sp|P21399|ACOHC_HUMAN | Cytoplasmic aconitate hydratase | 18.24 | 18.75 | 18.38 |
| 50 | ACO2 | Non-matrisome | Non-matrisome | GO:CC not extracellular matrix | sp|Q99798|ACON_HUMAN | Aconitate hydratase, mitochondrial | 18.67 | 19.23 | 18.96 |
| 51 | ACOT11 | Non-matrisome | Non-matrisome | GO:CC not extracellular matrix | sp|Q8WXI4|ACO11_HUMAN | Acyl-coenzyme A thioesterase 11 | 17.27 | 17.36 | 17.14 |
| 52 | ACOT13 | Non-matrisome | Non-matrisome | GO:CC not extracellular matrix | sp|Q9NPJ3|ACO13_HUMAN | Acyl-coenzyme A thioesterase 13 | 17.62 | 17.96 | 17.79 |
| 53 | ACOT7 | Non-matrisome | Non-matrisome | GO:CC not extracellular matrix | sp|O00154|BACH_HUMAN | Cytosolic acyl coenzyme A thioester hydrolase | 18.04 | 17.77 | 17.51 |
| 54 | ACOT9 | Non-matrisome | Non-matrisome | GO:CC not extracellular matrix | sp|Q9Y305|ACOT9_HUMAN | Acyl-coenzyme A thioesterase 9, mitochondrial | 18.18 | 18.28 | 17.94 |
| 55 | ACOX1 | Non-matrisome | Non-matrisome | GO:CC extracellular matrix (GO:0005576, GO:0005578 & GO:0031012) & GO:CC not extracellular matrix | sp|Q15067|ACOX1_HUMAN | Peroxisomal acyl-coenzyme A oxidase 1 | 17.63 | 17.61 | 17.77 |
| 56 | ACOX3 | Non-matrisome | Non-matrisome | GO:CC not extracellular matrix | sp|O15254|ACOX3_HUMAN | Peroxisomal acyl-coenzyme A oxidase 3 | 17.95 | 18.31 | 18.09 |
| 57 | ACP1 | Non-matrisome | Non-matrisome | GO:CC not extracellular matrix | sp|P24666|PPAC_HUMAN | Low molecular weight phosphotyrosine protein phosphatase | 17.73 | 17.68 | 17.6 |
| 58 | ACP2 | Non-matrisome | Non-matrisome | GO:CC not extracellular matrix | sp|P11117|PPAL_HUMAN | Lysosomal acid phosphatase | 16.86 | 17.9 | 18.23 |
| 59 | ACP6 | Non-matrisome | Non-matrisome | GO:CC not extracellular matrix | sp|Q9NPH0|PPA6_HUMAN | Lysophosphatidic acid phosphatase type 6 | 15.27 | 15.98 | 15.61 |
| 60 | ACSF2 | Non-matrisome | Non-matrisome | GO:CC not extracellular matrix | sp|Q96CM8|ACSF2_HUMAN | Medium-chain acyl-CoA ligase ACSF2, mitochondrial | 16.79 | 17.91 | 17.89 |
| 61 | ACSL1 | Non-matrisome | Non-matrisome | GO:CC not extracellular matrix | sp|P33121|ACSL1_HUMAN | Long-chain-fatty-acid--CoA ligase 1 | 18.83 | 18.91 | 18.87 |
| 62 | ACSL3 | Non-matrisome | Non-matrisome | GO:CC not extracellular matrix | sp|O95573|ACSL3_HUMAN | Fatty acid CoA ligase Acsl3 | 18.67 | 18.01 | 17.93 |
| 63 | ACSL4 | Non-matrisome | Non-matrisome | GO:CC not extracellular matrix | sp|O60488|ACSL4_HUMAN | Long-chain-fatty-acid--CoA ligase 4 | 17 | 18.11 | 18.36 |
| 64 | ACSS1 | Non-matrisome | Non-matrisome | GO:CC not extracellular matrix | sp|Q9NUB1|ACS2L_HUMAN | Acetyl-coenzyme A synthetase 2-like, mitochondrial | 16.57 | 16.39 | 16.35 |
| 65 | ACSS2 | Non-matrisome | Non-matrisome | GO:CC not extracellular matrix | sp|Q9NR19|ACSA_HUMAN | Acetyl-coenzyme A synthetase, cytoplasmic | 17.28 | 17.59 | 17.73 |
| 66 | ACSS3 | Non-matrisome | Non-matrisome | GO:CC not extracellular matrix | sp|Q9H6R3|ACSS3_HUMAN | Acyl-CoA synthetase short-chain family member 3, mitochondrial | 16.7 | 17.95 | 18.05 |
| 67 | ACTA1 | Non-matrisome | Non-matrisome | GO:CC not extracellular matrix | sp|P68133|ACTS_HUMAN | Actin, alpha skeletal muscle | 15.22 | 14.61 | 14.68 |
| 68 | ACTA2 | Non-matrisome | Non-matrisome | GO:CC not extracellular matrix | sp|P62736|ACTA_HUMAN | Actin, aortic smooth muscle | 17.74 | 13.67 | 14.99 |
| 69 | ACTB | Non-matrisome | Non-matrisome | GO:CC not extracellular matrix | sp|P60709|ACTB_HUMAN | Actin, cytoplasmic 1 | 21.64 | 21.17 | 21.16 |
| 70 | ACTBL2 | Non-matrisome | Non-matrisome | GO:CC not extracellular matrix | sp|Q562R1|ACTBL_HUMAN | Beta-actin-like protein 2 | 14.95 | 14.79 | 14.53 |
| 71 | ACTG1 | Non-matrisome | Non-matrisome | GO:CC not extracellular matrix | sp|P63261|ACTG_HUMAN | Actin, cytoplasmic 2 | 19.91 | 18.95 | 19.14 |
| 72 | ACTG2 | Non-matrisome | Non-matrisome | GO:CC not extracellular matrix | sp|P63267|ACTH_HUMAN | Actin, gamma-enteric smooth muscle | 14.25 | 13.18 | 13.11 |
| 73 | ACTL6A | Non-matrisome | Non-matrisome | GO:CC not extracellular matrix | sp|O96019|ACL6A_HUMAN | Actin-like protein 6A | 17.8 | 17.34 | 17.54 |
| 74 | ACTN1 | Non-matrisome | Non-matrisome | GO:CC extracellular matrix (GO:0005576, GO:0005578 & GO:0031012) & GO:CC not extracellular matrix | sp|P12814|ACTN1_HUMAN | Alpha-actinin-1 | 19.59 | 19.26 | 19.1 |
| 75 | ACTN4 | Non-matrisome | Non-matrisome | GO:CC extracellular matrix (GO:0005576, GO:0005578 & GO:0031012) & GO:CC not extracellular matrix | sp|O43707|ACTN4_HUMAN | Alpha-actinin-4 | 19.17 | 19.16 | 18.95 |
| 76 | ACTR10 | Non-matrisome | Non-matrisome | GO:CC extracellular matrix (GO:0005576, GO:0005578 & GO:0031012) & GO:CC not extracellular matrix | sp|Q9NZ32|ARP10_HUMAN | Actin-related protein 10 | 18.64 | 18.49 | 18.43 |
| 77 | ACTR1A | Non-matrisome | Non-matrisome | GO:CC not extracellular matrix | sp|P61163|ACTZ_HUMAN | Alpha-centractin | 16.56 | 16.55 | 16.52 |
| 78 | ACTR1B | Non-matrisome | Non-matrisome | GO:CC extracellular matrix (GO:0005576, GO:0005578 & GO:0031012) & GO:CC not extracellular matrix | sp|P42025|ACTY_HUMAN | Beta-centractin | 15.95 | 16.28 | 16.06 |
| 79 | ACTR2 | Non-matrisome | Non-matrisome | GO:CC extracellular matrix (GO:0005576, GO:0005578 & GO:0031012) & GO:CC not extracellular matrix | sp|P61160|ARP2_HUMAN | Actin-related protein 2 | 18.15 | 18.24 | 18.09 |
| 80 | ACTR3 | Non-matrisome | Non-matrisome | GO:CC not extracellular matrix | sp|P61158|ARP3_HUMAN | Actin-related protein 3 | 18.13 | 18.28 | 18.22 |
| 81 | ACTR6 | Non-matrisome | Non-matrisome | GO:CC not extracellular matrix | sp|Q9GZN1|ARP6_HUMAN | Actin-related protein 6 | 15.87 | 16.22 | 16.07 |
| 82 | ACTR8 | Non-matrisome | Non-matrisome | GO:CC not extracellular matrix | sp|Q9H981|ARP8_HUMAN | Actin-related protein 8 | 17.27 | 16.77 | 16.82 |
| 83 | ACY1 | Non-matrisome | Non-matrisome | GO:CC not extracellular matrix | sp|Q03154|ACY1_HUMAN | Aminoacylase-1 | 17.36 | 18.07 | 18.34 |
| 84 | ACYP1 | Non-matrisome | Non-matrisome | GO:CC not extracellular matrix | sp|P07311|ACYP1_HUMAN | Acylphosphatase-1 | 17.22 | 18.08 | 18.41 |
| 85 | ACYP2 | Non-matrisome | Non-matrisome | GO:CC not extracellular matrix | sp|P14621|ACYP2_HUMAN | Acylphosphatase-2 | 17 | 17.34 | 17.2 |
| 86 | ADA | Non-matrisome | Non-matrisome | GO:CC not extracellular matrix | sp|P00813|ADA_HUMAN | Adenosine deaminase | 17.3 | 18.38 | 18.56 |
| 87 | ADAM10 | Matrisome-associated | ECM Regulators | GO:CC not extracellular matrix | sp|O14672|ADA10_HUMAN | Disintegrin and metalloproteinase domain-containing protein 10 | 16.88 | 17.51 | 17.87 |
| 88 | ADAM15 | Matrisome-associated | ECM Regulators | GO:CC not extracellular matrix | sp|Q13444|ADA15_HUMAN | Disintegrin and metalloproteinase domain-containing protein 15 | 16.2 | 15.21 | 15.32 |
| 89 | ADAM17 | Matrisome-associated | ECM Regulators | GO:CC not extracellular matrix | sp|P78536|ADA17_HUMAN | Disintegrin and metalloproteinase domain-containing protein 17 | 17.01 | 17.78 | 18.08 |
| 90 | ADAM9 | Matrisome-associated | ECM Regulators | GO:CC not extracellular matrix | sp|Q13443|ADAM9_HUMAN | Disintegrin and metalloproteinase domain-containing protein 9 | 16.76 | 15.62 | 15.83 |
| 91 | ADAMTSL4 | Matrisome-associated | ECM Regulators | GO:CC extracellular matrix (GO:0005576, GO:0005578 & GO:0031012) & GO:CC not extracellular matrix | sp|Q6UY14|ATL4_HUMAN | ADAMTS-like protein 4 | 14.11 | 15.63 | 15.41 |
| 92 | ADAR | Non-matrisome | Non-matrisome | GO:CC not extracellular matrix | sp|P55265|DSRAD_HUMAN | Double-stranded RNA-specific adenosine deaminase | 17.83 | 18.1 | 18.15 |
| 93 | ADD1 | Non-matrisome | Non-matrisome | GO:CC extracellular matrix (GO:0005576, GO:0005578 & GO:0031012) & GO:CC not extracellular matrix | sp|P35611|ADDA_HUMAN | Alpha-adducin | 17.23 | 17.51 | 17.52 |
| 94 | ADD3 | Non-matrisome | Non-matrisome | GO:CC not extracellular matrix | sp|Q9UEY8|ADDG_HUMAN | Gamma-adducin | 17.02 | 17.68 | 17.58 |
| 95 | ADGRE5 | Non-matrisome | Non-matrisome | GO:CC not extracellular matrix | sp|P48960|AGRE5_HUMAN | Adhesion G protein-coupled receptor E5 | 16.34 | 15.78 | 15.99 |
| 96 | ADH5 | Non-matrisome | Non-matrisome | GO:CC not extracellular matrix | sp|P11766|ADHX_HUMAN | Alcohol dehydrogenase class-3 | 20.33 | 20.83 | 21.03 |
| 97 | ADI1 | Non-matrisome | Non-matrisome | GO:CC not extracellular matrix | sp|Q9BV57|MTND_HUMAN | Acireductone dioxygenase | 17.62 | 17.39 | 17.28 |
| 98 | ADIRF | Non-matrisome | Non-matrisome | GO:CC not extracellular matrix | sp|Q15847|ADIRF_HUMAN | Adipogenesis regulatory factor | 16.95 | 17.14 | 17.21 |
| 99 | ADK | Non-matrisome | Non-matrisome | GO:CC not extracellular matrix | sp|P55263|ADK_HUMAN | Adenosine kinase | 19.47 | 19.55 | 19.37 |
| 100 | ADO | Non-matrisome | Non-matrisome | GO:CC not extracellular matrix | sp|Q96SZ5|AEDO_HUMAN | 2-aminoethanethiol dioxygenase | 17.38 | 17.72 | 18.06 |
| 101 | ADPGK | Non-matrisome | Non-matrisome | GO:CC extracellular matrix (GO:0005576, GO:0005578 & GO:0031012) & GO:CC not extracellular matrix | sp|Q9BRR6|ADPGK_HUMAN | ADP-dependent glucokinase | 17.59 | 17.9 | 17.78 |
| 102 | ADPRS | Non-matrisome | Non-matrisome | GO:CC not extracellular matrix | sp|Q9NX46|ADPRS_HUMAN | ADP-ribosylhydrolase ARH3 | 18.17 | 18.5 | 18.39 |
| 103 | ADRM1 | Non-matrisome | Non-matrisome | GO:CC not extracellular matrix | sp|Q16186|ADRM1_HUMAN | Proteasomal ubiquitin receptor ADRM1 | 17.45 | 17.08 | 17.18 |
| 104 | ADSL | Non-matrisome | Non-matrisome | GO:CC not extracellular matrix | sp|P30566|PUR8_HUMAN | Adenylosuccinate lyase | 18.9 | 18.66 | 18.44 |
| 105 | ADSS2 | Non-matrisome | Non-matrisome | GO:CC not extracellular matrix | sp|P30520|PURA2_HUMAN | Adenylosuccinate synthetase isozyme 2 | 18.73 | 18.2 | 18.18 |
| 106 | AEBP1 | Core matrisome | ECM Glycoproteins | GO:CC extracellular matrix (GO:0005576, GO:0005578 & GO:0031012) & GO:CC not extracellular matrix | sp|Q8IUX7|AEBP1_HUMAN | Adipocyte enhancer-binding protein 1 | 15.65 | 18.44 | 17.78 |
| 107 | AFDN | Non-matrisome | Non-matrisome | GO:CC not extracellular matrix | sp|P55196|AFAD_HUMAN | Afadin | 17.51 | 17.31 | 17.49 |
| 108 | AFG3L2 | Non-matrisome | Non-matrisome | GO:CC not extracellular matrix | sp|Q9Y4W6|AFG32_HUMAN | AFG3-like protein 2 | 18.05 | 18.53 | 18.37 |
| 109 | AFM | Non-matrisome | Non-matrisome | GO:CC extracellular matrix (GO:0005576, GO:0005578 & GO:0031012) & GO:CC not extracellular matrix | sp|P43652|AFAM_HUMAN | Afamin | 18.61 | 17.99 | 17.63 |
| 110 | AGA | Non-matrisome | Non-matrisome | GO:CC extracellular matrix (GO:0005576, GO:0005578 & GO:0031012) & GO:CC not extracellular matrix | sp|P20933|ASPG_HUMAN | N(4)-(beta-N-acetylglucosaminyl)-L-asparaginase | 15.4 | 16.95 | 17.48 |
| 111 | AGAP3 | Non-matrisome | Non-matrisome | GO:CC not extracellular matrix | sp|Q96P47|AGAP3_HUMAN | Arf-GAP with GTPase, ANK repeat and PH domain-containing protein 3 | 17.4 | 17.8 | 17.84 |
| 112 | AGFG1 | Non-matrisome | Non-matrisome | GO:CC not extracellular matrix | sp|P52594|AGFG1_HUMAN | Arf-GAP domain and FG repeat-containing protein 1 | 18.34 | 18.25 | 18.43 |
| 113 | AGK | Non-matrisome | Non-matrisome | GO:CC not extracellular matrix | sp|Q53H12|AGK_HUMAN | Acylglycerol kinase, mitochondrial | 18.54 | 19.03 | 18.83 |
| 114 | AGL | Non-matrisome | Non-matrisome | GO:CC extracellular matrix (GO:0005576, GO:0005578 & GO:0031012) & GO:CC not extracellular matrix | sp|P35573|GDE_HUMAN | Glycogen debranching enzyme | 17.49 | 17.95 | 17.94 |
| 115 | AGO1 | Non-matrisome | Non-matrisome | GO:CC extracellular matrix (GO:0005576, GO:0005578 & GO:0031012) & GO:CC not extracellular matrix | sp|Q9UL18|AGO1_HUMAN | Protein argonaute-1 | 17.73 | 18.44 | 18.41 |
| 116 | AGO2 | Non-matrisome | Non-matrisome | GO:CC extracellular matrix (GO:0005576, GO:0005578 & GO:0031012) & GO:CC not extracellular matrix | sp|Q9UKV8|AGO2_HUMAN | Protein argonaute-2 | 17.72 | 17.72 | 17.69 |
| 117 | AGO3 | Non-matrisome | Non-matrisome | GO:CC extracellular matrix (GO:0005576, GO:0005578 & GO:0031012) & GO:CC not extracellular matrix | sp|Q9H9G7|AGO3_HUMAN | Protein argonaute-3 | 17.63 | 18.23 | 18.27 |
| 118 | AGPAT3 | Non-matrisome | Non-matrisome | GO:CC not extracellular matrix | sp|Q9NRZ7|PLCC_HUMAN | 1-acyl-sn-glycerol-3-phosphate acyltransferase gamma | 16.88 | 17.2 | 16.94 |
| 119 | AGPS | Non-matrisome | Non-matrisome | GO:CC not extracellular matrix | sp|O00116|ADAS_HUMAN | Alkyldihydroxyacetonephosphate synthase, peroxisomal | 17.62 | 17.3 | 17.18 |
| 120 | AGRN | Core matrisome | ECM Glycoproteins | GO:CC extracellular matrix (GO:0005576, GO:0005578 & GO:0031012) & GO:CC not extracellular matrix | sp|O00468|AGRIN_HUMAN | Agrin | 15.05 | 16.55 | 16.23 |
| 121 | AHCTF1 | Non-matrisome | Non-matrisome | GO:CC not extracellular matrix | sp|Q8WYP5|ELYS_HUMAN | Protein ELYS | 17.16 | 17.14 | 17.12 |
| 122 | AHCY | Non-matrisome | Non-matrisome | GO:CC not extracellular matrix | sp|P23526|SAHH_HUMAN | Adenosylhomocysteinase | 19.68 | 19.52 | 19.47 |
| 123 | AHCYL1 | Non-matrisome | Non-matrisome | GO:CC not extracellular matrix | sp|O43865|SAHH2_HUMAN | S-adenosylhomocysteine hydrolase-like protein 1 | 18.07 | 18.31 | 18.55 |
| 124 | AHCYL2 | Non-matrisome | Non-matrisome | GO:CC not extracellular matrix | sp|Q96HN2|SAHH3_HUMAN | Adenosylhomocysteinase 3 | 17.28 | 17.95 | 18.15 |
| 125 | AHNAK | Non-matrisome | Non-matrisome | GO:CC not extracellular matrix | sp|Q09666|AHNK_HUMAN | Neuroblast differentiation-associated protein AHNAK | 20.16 | 19.37 | 19.3 |
| 126 | AHNAK2 | Non-matrisome | Non-matrisome | GO:CC not extracellular matrix | sp|Q8IVF2|AHNK2_HUMAN | Protein AHNAK2 | 18.32 | 17.2 | 16.97 |
| 127 | AHSA1 | Non-matrisome | Non-matrisome | GO:CC not extracellular matrix | sp|O95433|AHSA1_HUMAN | Activator of 90 kDa heat shock protein ATPase homolog 1 | 18.48 | 17.84 | 18.15 |
| 128 | AHSG | Non-matrisome | Non-matrisome | GO:CC extracellular matrix (GO:0005576, GO:0005578 & GO:0031012) & GO:CC not extracellular matrix | sp|P02765|FETUA_HUMAN | Alpha-2-HS-glycoprotein | 23.48 | 22.24 | 21.66 |
| 129 | AIDA | Non-matrisome | Non-matrisome | GO:CC not extracellular matrix | sp|Q96BJ3|AIDA_HUMAN | Axin interactor, dorsalization-associated protein | 16.96 | 17.23 | 17.35 |
| 130 | AIFM1 | Non-matrisome | Non-matrisome | GO:CC not extracellular matrix | sp|O95831|AIFM1_HUMAN | Apoptosis-inducing factor 1, mitochondrial | 18.6 | 19.21 | 19.14 |
| 131 | AIMP1 | Non-matrisome | Non-matrisome | GO:CC extracellular matrix (GO:0005576, GO:0005578 & GO:0031012) & GO:CC not extracellular matrix | sp|Q12904|AIMP1_HUMAN | Aminoacyl tRNA synthase complex-interacting multifunctional protein 1 | 19.3 | 18.82 | 18.87 |
| 132 | AIMP2 | Non-matrisome | Non-matrisome | GO:CC extracellular matrix (GO:0005576, GO:0005578 & GO:0031012) & GO:CC not extracellular matrix | sp|Q13155|AIMP2_HUMAN | Aminoacyl tRNA synthase complex-interacting multifunctional protein 2 | 19.26 | 18.82 | 18.67 |
| 133 | AIP | Non-matrisome | Non-matrisome | GO:CC not extracellular matrix | sp|O00170|AIP_HUMAN | AH receptor-interacting protein | 18.09 | 18.5 | 18.53 |
| 134 | AJUBA | Non-matrisome | Non-matrisome | GO:CC not extracellular matrix | sp|Q96IF1|AJUBA_HUMAN | LIM domain-containing protein ajuba | 15.52 | 15.59 | 15.69 |
| 135 | AK1 | Non-matrisome | Non-matrisome | GO:CC not extracellular matrix | sp|P00568|KAD1_HUMAN | Adenylate kinase isoenzyme 1 | 17.45 | 18.27 | 18.52 |
| 136 | AK2 | Non-matrisome | Non-matrisome | GO:CC not extracellular matrix | sp|P54819|KAD2_HUMAN | Adenylate kinase 2, mitochondrial | 18.69 | 19.04 | 18.92 |
| 137 | AK3 | Non-matrisome | Non-matrisome | GO:CC not extracellular matrix | sp|Q9UIJ7|KAD3_HUMAN | GTP:AMP phosphotransferase AK3, mitochondrial | 18.4 | 19.29 | 19.31 |
| 138 | AK4 | Non-matrisome | Non-matrisome | GO:CC not extracellular matrix | sp|P27144|KAD4_HUMAN | Adenylate kinase 4, mitochondrial | 17.04 | 20 | 18.44 |
| 139 | AKAP12 | Non-matrisome | Non-matrisome | GO:CC not extracellular matrix | sp|Q02952|AKA12_HUMAN | A-kinase anchor protein 12 | 18.76 | 16.78 | 17.26 |
| 140 | AKAP13 | Non-matrisome | Non-matrisome | GO:CC not extracellular matrix | sp|Q12802|AKP13_HUMAN | A-kinase anchor protein 13 | 17.14 | 16.81 | 16.85 |
| 141 | AKAP8 | Non-matrisome | Non-matrisome | GO:CC not extracellular matrix | sp|O43823|AKAP8_HUMAN | A-kinase anchor protein 8 | 18.07 | 18.02 | 18.01 |
| 142 | AKR1A1 | Non-matrisome | Non-matrisome | GO:CC not extracellular matrix | sp|P14550|AK1A1_HUMAN | Aldo-keto reductase family 1 member A1 | 18.65 | 19 | 19.24 |
| 143 | AKR1B1 | Non-matrisome | Non-matrisome | GO:CC not extracellular matrix | sp|P15121|ALDR_HUMAN | Aldo-keto reductase family 1 member B1 | 19.3 | 20.46 | 21.11 |
| 144 | AKR1B10 | Non-matrisome | Non-matrisome | GO:CC extracellular matrix (GO:0005576, GO:0005578 & GO:0031012) & GO:CC not extracellular matrix | sp|O60218|AK1BA_HUMAN | Aldo-keto reductase family 1 member B10 | 17.07 | 17.32 | 18.73 |
| 145 | AKR1C1 | Non-matrisome | Non-matrisome | GO:CC not extracellular matrix | sp|Q04828|AK1C1_HUMAN | Aldo-keto reductase family 1 member C1 | 16.82 | 16.76 | 16.77 |
| 146 | AKR1C2 | Non-matrisome | Non-matrisome | GO:CC not extracellular matrix | sp|P52895|AK1C2_HUMAN | Aldo-keto reductase family 1 member C2 | 17.48 | 18.31 | 18.59 |
| 147 | AKR1C3 | Non-matrisome | Non-matrisome | GO:CC not extracellular matrix | sp|P42330|AK1C3_HUMAN | Aldo-keto reductase family 1 member C3 | 17.28 | 18.31 | 18.64 |
| 148 | AKR7A2 | Non-matrisome | Non-matrisome | GO:CC not extracellular matrix | sp|O43488|ARK72_HUMAN | Aflatoxin B1 aldehyde reductase member 2 | 17.87 | 18.73 | 19.01 |
| 149 | AKT1 | Non-matrisome | Non-matrisome | GO:CC not extracellular matrix | sp|P31749|AKT1_HUMAN | RAC-alpha serine/threonine-protein kinase | 18.3 | 18.26 | 18.37 |
| 150 | AKT2 | Non-matrisome | Non-matrisome | GO:CC not extracellular matrix | sp|P31751|AKT2_HUMAN | RAC-beta serine/threonine-protein kinase | 17.73 | 17.47 | 17.63 |
| 151 | ALAD | Non-matrisome | Non-matrisome | GO:CC extracellular matrix (GO:0005576, GO:0005578 & GO:0031012) & GO:CC not extracellular matrix | sp|P13716|HEM2_HUMAN | Delta-aminolevulinic acid dehydratase | 17.23 | 18.12 | 18.14 |
| 152 | ALCAM | Non-matrisome | Non-matrisome | GO:CC not extracellular matrix | sp|Q13740|CD166_HUMAN | CD166 antigen | 19.52 | 17.89 | 17.42 |
| 153 | ALDH16A1 | Non-matrisome | Non-matrisome | GO:CC not extracellular matrix | sp|Q8IZ83|A16A1_HUMAN | Aldehyde dehydrogenase family 16 member A1 | 18.62 | 18.66 | 18.44 |
| 154 | ALDH18A1 | Non-matrisome | Non-matrisome | GO:CC not extracellular matrix | sp|P54886|P5CS_HUMAN | Delta-1-pyrroline-5-carboxylate synthase | 18.46 | 18.29 | 18.25 |
| 155 | ALDH1A2 | Non-matrisome | Non-matrisome | GO:CC not extracellular matrix | sp|O94788|AL1A2_HUMAN | Retinal dehydrogenase 2 | 16.92 | 14.79 | 14.81 |
| 156 | ALDH1B1 | Non-matrisome | Non-matrisome | GO:CC not extracellular matrix | sp|P30837|AL1B1_HUMAN | Aldehyde dehydrogenase X, mitochondrial | 18.22 | 17.72 | 17.29 |
| 157 | ALDH1L2 | Non-matrisome | Non-matrisome | GO:CC not extracellular matrix | sp|Q3SY69|AL1L2_HUMAN | Mitochondrial 10-formyltetrahydrofolate dehydrogenase | 18.63 | 18.38 | 17.96 |
| 158 | ALDH2 | Non-matrisome | Non-matrisome | GO:CC not extracellular matrix | sp|P05091|ALDH2_HUMAN | Aldehyde dehydrogenase, mitochondrial | 17.59 | 18.28 | 17.95 |
| 159 | ALDH3A1 | Non-matrisome | Non-matrisome | GO:CC not extracellular matrix | sp|P30838|AL3A1_HUMAN | Aldehyde dehydrogenase, dimeric NADP-preferring | 15.83 | 16.97 | 17.94 |
| 160 | ALDH3A2 | Non-matrisome | Non-matrisome | GO:CC not extracellular matrix | sp|P51648|AL3A2_HUMAN | Aldehyde dehydrogenase family 3 member A2 | 17.32 | 18.44 | 19.18 |
| 161 | ALDH3B1 | Non-matrisome | Non-matrisome | GO:CC not extracellular matrix | sp|P43353|AL3B1_HUMAN | Aldehyde dehydrogenase family 3 member B1 | 15.52 | 15.82 | 15.65 |
| 162 | ALDH4A1 | Non-matrisome | Non-matrisome | GO:CC not extracellular matrix | sp|P30038|AL4A1_HUMAN | Delta-1-pyrroline-5-carboxylate dehydrogenase, mitochondrial | 18.03 | 18.64 | 18.17 |
| 163 | ALDH6A1 | Non-matrisome | Non-matrisome | GO:CC not extracellular matrix | sp|Q02252|MMSA_HUMAN | Methylmalonate-semialdehyde/malonate-semialdehyde dehydrogenase [acylating], mitochondrial | 17 | 18.43 | 17.9 |
| 164 | ALDH7A1 | Non-matrisome | Non-matrisome | GO:CC not extracellular matrix | sp|P49419|AL7A1_HUMAN | Alpha-aminoadipic semialdehyde dehydrogenase | 18.27 | 18.75 | 18.44 |
| 165 | ALDH9A1 | Non-matrisome | Non-matrisome | GO:CC not extracellular matrix | sp|P49189|AL9A1_HUMAN | 4-trimethylaminobutyraldehyde dehydrogenase | 17.59 | 18.1 | 17.9 |
| 166 | ALDOA | Non-matrisome | Non-matrisome | GO:CC extracellular matrix (GO:0005576, GO:0005578 & GO:0031012) & GO:CC not extracellular matrix | sp|P04075|ALDOA_HUMAN | Fructose-bisphosphate aldolase A | 17.81 | 18.28 | 17.85 |
| 167 | ALDOC | Non-matrisome | Non-matrisome | GO:CC extracellular matrix (GO:0005576, GO:0005578 & GO:0031012) & GO:CC not extracellular matrix | sp|P09972|ALDOC_HUMAN | Fructose-bisphosphate aldolase C | 17.68 | 19.06 | 18.25 |
| 168 | ALG1 | Non-matrisome | Non-matrisome | GO:CC not extracellular matrix | sp|Q9BT22|ALG1_HUMAN | Chitobiosyldiphosphodolichol beta-mannosyltransferase | 17.4 | 17.43 | 17.44 |
| 169 | ALG12 | Non-matrisome | Non-matrisome | GO:CC not extracellular matrix | sp|Q9BV10|ALG12_HUMAN | Dol-P-Man:Man(7)GlcNAc(2)-PP-Dol alpha-1,6-mannosyltransferase | 15.69 | 16.38 | 16.37 |
| 170 | ALG2 | Non-matrisome | Non-matrisome | GO:CC not extracellular matrix | sp|Q9H553|ALG2_HUMAN | Alpha-1,3/1,6-mannosyltransferase ALG2 | 18.1 | 17.98 | 17.94 |
| 171 | ALG5 | Non-matrisome | Non-matrisome | GO:CC not extracellular matrix | sp|Q9Y673|ALG5_HUMAN | Dolichyl-phosphate beta-glucosyltransferase | 18.25 | 18.32 | 18.28 |
| 172 | ALOX5 | Non-matrisome | Non-matrisome | GO:CC extracellular matrix (GO:0005576, GO:0005578 & GO:0031012) & GO:CC not extracellular matrix | sp|P09917|LOX5_HUMAN | Polyunsaturated fatty acid 5-lipoxygenase | 13.72 | 13.76 | 13.68 |
| 173 | ALYREF | Non-matrisome | Non-matrisome | GO:CC not extracellular matrix | sp|Q86V81|THOC4_HUMAN | THO complex subunit 4 | 17.7 | 17.32 | 17.53 |
| 174 | AMACR | Non-matrisome | Non-matrisome | GO:CC not extracellular matrix | sp|Q9UHK6|AMACR_HUMAN | Alpha-methylacyl-CoA racemase | 15.09 | 15.03 | 15.1 |
| 175 | AMBP | Matrisome-associated | ECM Regulators | GO:CC extracellular matrix (GO:0005576, GO:0005578 & GO:0031012) & GO:CC not extracellular matrix | sp|P02760|AMBP_HUMAN | Protein AMBP | 18.23 | 17.03 | 16.96 |
| 176 | AMDHD2 | Non-matrisome | Non-matrisome | GO:CC not extracellular matrix | sp|Q9Y303|NAGA_HUMAN | N-acetylglucosamine-6-phosphate deacetylase | 16.85 | 16.99 | 17.04 |
| 177 | AMPD2 | Non-matrisome | Non-matrisome | GO:CC not extracellular matrix | sp|Q01433|AMPD2_HUMAN | AMP deaminase 2 | 18.12 | 18.53 | 18.49 |
| 178 | AMPD3 | Non-matrisome | Non-matrisome | GO:CC extracellular matrix (GO:0005576, GO:0005578 & GO:0031012) & GO:CC not extracellular matrix | sp|Q01432|AMPD3_HUMAN | AMP deaminase 3 | 16.11 | 17.8 | 18.44 |
| 179 | ANAPC5 | Non-matrisome | Non-matrisome | GO:CC not extracellular matrix | sp|Q9UJX4|APC5_HUMAN | Anaphase-promoting complex subunit 5 | 17.74 | 17.5 | 17.5 |
| 180 | ANAPC7 | Non-matrisome | Non-matrisome | GO:CC not extracellular matrix | sp|Q9UJX3|APC7_HUMAN | Anaphase-promoting complex subunit 7 | 18.82 | 18.32 | 18.52 |
| 181 | ANGPTL2 | Matrisome-associated | Secreted Factors | GO:CC not extracellular matrix | sp|Q9UKU9|ANGL2_HUMAN | Angiopoietin-related protein 2 | 14.06 | 17.38 | 16.49 |
| 182 | ANK1 | Non-matrisome | Non-matrisome | GO:CC not extracellular matrix | sp|P16157|ANK1_HUMAN | Ankyrin-1 | 14.93 | 15.69 | 15.68 |
| 183 | ANK2 | Non-matrisome | Non-matrisome | GO:CC not extracellular matrix | sp|Q01484|ANK2_HUMAN | Ankyrin-2 | 15.57 | 16.07 | 15.64 |
| 184 | ANK3 | Non-matrisome | Non-matrisome | GO:CC not extracellular matrix | sp|Q12955|ANK3_HUMAN | Ankyrin-3 | 16.56 | 16.93 | 16.9 |
| 185 | ANKFY1 | Non-matrisome | Non-matrisome | GO:CC not extracellular matrix | sp|Q9P2R3|ANFY1_HUMAN | Rabankyrin-5 | 17.9 | 18.09 | 18.06 |
| 186 | ANKHD1 | Non-matrisome | Non-matrisome | GO:CC not extracellular matrix | sp|Q8IWZ3|ANKH1_HUMAN | Ankyrin repeat and KH domain-containing protein 1 | 17.31 | 16.81 | 16.85 |
| 187 | ANKRD26 | Non-matrisome | Non-matrisome | GO:CC not extracellular matrix | sp|Q9UPS8|ANR26_HUMAN | Ankyrin repeat domain-containing protein 26 | 15.79 | 15.54 | 15.47 |
| 188 | ANKRD35 | Non-matrisome | Non-matrisome | GO:CC not extracellular matrix | sp|Q8N283|ANR35_HUMAN | Ankyrin repeat domain-containing protein 35 | 14.86 | 15.21 | 15.18 |
| 189 | ANKRD44 | Non-matrisome | Non-matrisome | GO:CC not extracellular matrix | sp|Q8N8A2|ANR44_HUMAN | Serine/threonine-protein phosphatase 6 regulatory ankyrin repeat subunit B | 15.8 | 16.17 | 16.43 |
| 190 | ANO10 | Non-matrisome | Non-matrisome | GO:CC not extracellular matrix | sp|Q9NW15|ANO10_HUMAN | Anoctamin-10 | 18.39 | 18.51 | 18.45 |
| 191 | ANO6 | Non-matrisome | Non-matrisome | GO:CC not extracellular matrix | sp|Q4KMQ2|ANO6_HUMAN | Anoctamin-6 | 16.94 | 17.63 | 17.47 |
| 192 | ANP32A | Non-matrisome | Non-matrisome | GO:CC not extracellular matrix | sp|P39687|AN32A_HUMAN | Acidic leucine-rich nuclear phosphoprotein 32 family member A | 18.97 | 19.29 | 19.32 |
| 193 | ANP32B | Non-matrisome | Non-matrisome | GO:CC not extracellular matrix | sp|Q92688|AN32B_HUMAN | Acidic leucine-rich nuclear phosphoprotein 32 family member B | 17.51 | 17.5 | 17.46 |
| 194 | ANP32E | Non-matrisome | Non-matrisome | GO:CC not extracellular matrix | sp|Q9BTT0|AN32E_HUMAN | Acidic leucine-rich nuclear phosphoprotein 32 family member E | 18.33 | 18.42 | 18.35 |
| 195 | ANPEP | Non-matrisome | Non-matrisome | GO:CC not extracellular matrix | sp|P15144|AMPN_HUMAN | Aminopeptidase N | 18.5 | 19.66 | 19.79 |
| 196 | ANXA1 | Matrisome-associated | ECM-affiliated Proteins | GO:CC extracellular matrix (GO:0005576, GO:0005578 & GO:0031012) & GO:CC not extracellular matrix | sp|P04083|ANXA1_HUMAN | Annexin A1 | 19.84 | 20.23 | 19.64 |
| 197 | ANXA11 | Matrisome-associated | ECM-affiliated Proteins | GO:CC not extracellular matrix | sp|P50995|ANX11_HUMAN | Annexin A11 | 18.87 | 19.59 | 19.63 |
| 198 | ANXA2 | Matrisome-associated | ECM-affiliated Proteins | GO:CC extracellular matrix (GO:0005576, GO:0005578 & GO:0031012) & GO:CC not extracellular matrix | sp|P07355|ANXA2_HUMAN | Annexin A2 | 19.68 | 19.95 | 19.54 |
| 199 | ANXA3 | Matrisome-associated | ECM-affiliated Proteins | GO:CC not extracellular matrix | sp|P12429|ANXA3_HUMAN | Annexin A3 | 17.43 | 17.48 | 17.36 |
| 200 | ANXA4 | Matrisome-associated | ECM-affiliated Proteins | GO:CC not extracellular matrix | sp|P09525|ANXA4_HUMAN | Annexin A4 | 18.78 | 19.86 | 19.93 |
| 201 | ANXA5 | Matrisome-associated | ECM-affiliated Proteins | GO:CC extracellular matrix (GO:0005576, GO:0005578 & GO:0031012) & GO:CC not extracellular matrix | sp|P08758|ANXA5_HUMAN | Annexin A5 | 20.83 | 21.13 | 21.03 |
| 202 | ANXA6 | Matrisome-associated | ECM-affiliated Proteins | GO:CC not extracellular matrix | sp|P08133|ANXA6_HUMAN | Annexin A6 | 20.57 | 20.85 | 20.8 |
| 203 | ANXA7 | Matrisome-associated | ECM-affiliated Proteins | GO:CC not extracellular matrix | sp|P20073|ANXA7_HUMAN | Annexin A7 | 17.46 | 17.71 | 17.78 |
| 204 | AOX1 | Non-matrisome | Non-matrisome | GO:CC extracellular matrix (GO:0005576, GO:0005578 & GO:0031012) & GO:CC not extracellular matrix | sp|Q06278|AOXA_HUMAN | Aldehyde oxidase | 17.77 | 16.21 | 16.36 |
| 205 | AP1B1 | Non-matrisome | Non-matrisome | GO:CC not extracellular matrix | sp|Q10567|AP1B1_HUMAN | AP-1 complex subunit beta-1 | 18.14 | 18.04 | 18.07 |
| 206 | AP1G1 | Non-matrisome | Non-matrisome | GO:CC not extracellular matrix | sp|O43747|AP1G1_HUMAN | AP-1 complex subunit gamma-1 | 18.31 | 18.27 | 18.15 |
| 207 | AP1G2 | Non-matrisome | Non-matrisome | GO:CC not extracellular matrix | sp|O75843|AP1G2_HUMAN | AP-1 complex subunit gamma-like 2 | 15.3 | 15.53 | 15.39 |
| 208 | AP1M1 | Non-matrisome | Non-matrisome | GO:CC not extracellular matrix | sp|Q9BXS5|AP1M1_HUMAN | AP-1 complex subunit mu-1 | 18.05 | 17.9 | 17.85 |
| 209 | AP1S1 | Non-matrisome | Non-matrisome | GO:CC not extracellular matrix | sp|P61966|AP1S1_HUMAN | AP-1 complex subunit sigma-1A | 18.75 | 18.63 | 18.55 |
| 210 | AP2A1 | Non-matrisome | Non-matrisome | GO:CC not extracellular matrix | sp|O95782|AP2A1_HUMAN | AP-2 complex subunit alpha-1 | 18.32 | 18.61 | 18.53 |
| 211 | AP2A2 | Non-matrisome | Non-matrisome | GO:CC not extracellular matrix | sp|O94973|AP2A2_HUMAN | AP-2 complex subunit alpha-2 | 17.64 | 18.2 | 18.17 |
| 212 | AP2B1 | Non-matrisome | Non-matrisome | GO:CC not extracellular matrix | sp|P63010|AP2B1_HUMAN | AP-2 complex subunit beta | 19.37 | 19.59 | 19.57 |
| 213 | AP2M1 | Non-matrisome | Non-matrisome | GO:CC not extracellular matrix | sp|Q96CW1|AP2M1_HUMAN | AP-2 complex subunit mu | 19.37 | 19.78 | 19.63 |
| 214 | AP2S1 | Non-matrisome | Non-matrisome | GO:CC not extracellular matrix | sp|P53680|AP2S1_HUMAN | AP-2 complex subunit sigma | 18.93 | 19.24 | 19.13 |
| 215 | AP3B1 | Non-matrisome | Non-matrisome | GO:CC not extracellular matrix | sp|O00203|AP3B1_HUMAN | AP-3 complex subunit beta-1 | 19.27 | 19.38 | 19.33 |
| 216 | AP3D1 | Non-matrisome | Non-matrisome | GO:CC not extracellular matrix | sp|O14617|AP3D1_HUMAN | AP-3 complex subunit delta-1 | 18.31 | 18.35 | 18.35 |
| 217 | AP3M1 | Non-matrisome | Non-matrisome | GO:CC not extracellular matrix | sp|Q9Y2T2|AP3M1_HUMAN | AP-3 complex subunit mu-1 | 17.79 | 18.02 | 17.93 |
| 218 | AP3S1 | Non-matrisome | Non-matrisome | GO:CC not extracellular matrix | sp|Q92572|AP3S1_HUMAN | AP-3 complex subunit sigma-1 | 19.38 | 19.38 | 19.44 |
| 219 | AP5Z1 | Non-matrisome | Non-matrisome | GO:CC not extracellular matrix | sp|O43299|AP5Z1_HUMAN | AP-5 complex subunit zeta-1 | 17 | 17.21 | 17.15 |
| 220 | APBA1 | Non-matrisome | Non-matrisome | GO:CC not extracellular matrix | sp|Q02410|APBA1_HUMAN | Amyloid-beta A4 precursor protein-binding family A member 1 | 15.96 | 16.02 | 16.19 |
| 221 | APBB1IP | Non-matrisome | Non-matrisome | GO:CC not extracellular matrix | sp|Q7Z5R6|AB1IP_HUMAN | Amyloid beta A4 precursor protein-binding family B member 1-interacting protein | 17.98 | 16.9 | 16.93 |
| 222 | APEH | Non-matrisome | Non-matrisome | GO:CC extracellular matrix (GO:0005576, GO:0005578 & GO:0031012) & GO:CC not extracellular matrix | sp|P13798|ACPH_HUMAN | Acylamino-acid-releasing enzyme | 17.74 | 18.07 | 18.02 |
| 223 | APEX1 | Non-matrisome | Non-matrisome | GO:CC not extracellular matrix | sp|P27695|APEX1_HUMAN | DNA-(apurinic or apyrimidinic site) endonuclease | 18.06 | 18.41 | 18.41 |
| 224 | API5 | Non-matrisome | Non-matrisome | GO:CC not extracellular matrix | sp|Q9BZZ5|API5_HUMAN | Apoptosis inhibitor 5 | 18.48 | 18.42 | 18.38 |
| 225 | APIP | Non-matrisome | Non-matrisome | GO:CC not extracellular matrix | sp|Q96GX9|MTNB_HUMAN | Methylthioribulose-1-phosphate dehydratase | 17.41 | 18.4 | 18.47 |
| 226 | APMAP | Non-matrisome | Non-matrisome | GO:CC not extracellular matrix | sp|Q9HDC9|APMAP_HUMAN | Adipocyte plasma membrane-associated protein | 18.47 | 18.88 | 18.69 |
| 227 | APOB | Non-matrisome | Non-matrisome | GO:CC extracellular matrix (GO:0005576, GO:0005578 & GO:0031012) & GO:CC not extracellular matrix | sp|P04114|APOB_HUMAN | Apolipoprotein B-100 | 17.91 | 17.21 | 19.85 |
| 228 | APOBEC3B | Non-matrisome | Non-matrisome | GO:CC not extracellular matrix | sp|Q9UH17|ABC3B_HUMAN | DNA dC->dU-editing enzyme APOBEC-3B | 16.86 | 16.9 | 17 |
| 229 | APOBEC3C | Non-matrisome | Non-matrisome | GO:CC not extracellular matrix | sp|Q9NRW3|ABC3C_HUMAN | DNA dC->dU-editing enzyme APOBEC-3C | 17.26 | 17.48 | 17.47 |
| 230 | APOBEC3F | Non-matrisome | Non-matrisome | GO:CC not extracellular matrix | sp|Q8IUX4|ABC3F_HUMAN | DNA dC->dU-editing enzyme APOBEC-3F | 15.74 | 16.33 | 16.16 |
| 231 | APOC3 | Non-matrisome | Non-matrisome | GO:CC extracellular matrix (GO:0005576, GO:0005578 & GO:0031012) & GO:CC not extracellular matrix | sp|P02656|APOC3_HUMAN | Apolipoprotein C-III | 15.42 | 19.4 | 18.41 |
| 232 | APOD | Non-matrisome | Non-matrisome | GO:CC extracellular matrix (GO:0005576, GO:0005578 & GO:0031012) & GO:CC not extracellular matrix | sp|P05090|APOD_HUMAN | Apolipoprotein D | 15.1 | 17.73 | 18.22 |
| 233 | APOE | Non-matrisome | Non-matrisome | GO:CC extracellular matrix (GO:0005576, GO:0005578 & GO:0031012) & GO:CC not extracellular matrix | sp|P02649|APOE_HUMAN | Apolipoprotein E | 14.89 | 18.53 | 19.08 |
| 234 | APOH | Non-matrisome | Non-matrisome | GO:CC extracellular matrix (GO:0005576, GO:0005578 & GO:0031012) & GO:CC not extracellular matrix | sp|P02749|APOH_HUMAN | Beta-2-glycoprotein 1 | 18.47 | 20.85 | 20.55 |
| 235 | APOL2 | Non-matrisome | Non-matrisome | GO:CC extracellular matrix (GO:0005576, GO:0005578 & GO:0031012) & GO:CC not extracellular matrix | sp|Q9BQE5|APOL2_HUMAN | Apolipoprotein L2 | 18.57 | 18.67 | 18.47 |
| 236 | APOM | Non-matrisome | Non-matrisome | GO:CC extracellular matrix (GO:0005576, GO:0005578 & GO:0031012) & GO:CC not extracellular matrix | sp|O95445|APOM_HUMAN | Apolipoprotein M | 16.41 | 20.1 | 18.55 |
| 237 | APOO | Non-matrisome | Non-matrisome | GO:CC extracellular matrix (GO:0005576, GO:0005578 & GO:0031012) & GO:CC not extracellular matrix | sp|Q9BUR5|MIC26_HUMAN | MICOS complex subunit MIC26 | 17.52 | 18.32 | 18.16 |
| 238 | APOOL | Non-matrisome | Non-matrisome | GO:CC extracellular matrix (GO:0005576, GO:0005578 & GO:0031012) & GO:CC not extracellular matrix | sp|Q6UXV4|MIC27_HUMAN | MICOS complex subunit MIC27 | 17.35 | 17.89 | 17.88 |
| 239 | APP | Non-matrisome | Non-matrisome | GO:CC extracellular matrix (GO:0005576, GO:0005578 & GO:0031012) & GO:CC not extracellular matrix | sp|P05067|A4_HUMAN | Amyloid-beta precursor protein | 17.43 | 18.99 | 18.76 |
| 240 | APPL1 | Non-matrisome | Non-matrisome | GO:CC not extracellular matrix | sp|Q9UKG1|DP13A_HUMAN | DCC-interacting protein 13-alpha | 16.7 | 16.94 | 16.87 |
| 241 | APRT | Non-matrisome | Non-matrisome | GO:CC extracellular matrix (GO:0005576, GO:0005578 & GO:0031012) & GO:CC not extracellular matrix | sp|P07741|APT_HUMAN | Adenine phosphoribosyltransferase | 20.69 | 21.21 | 21.3 |
| 242 | ARAF | Non-matrisome | Non-matrisome | GO:CC not extracellular matrix | sp|P10398|ARAF_HUMAN | Serine/threonine-protein kinase A-Raf | 15.9 | 15.95 | 15.86 |
| 243 | ARAP1 | Non-matrisome | Non-matrisome | GO:CC not extracellular matrix | sp|Q96P48|ARAP1_HUMAN | Arf-GAP with Rho-GAP domain, ANK repeat and PH domain-containing protein 1 | 17.34 | 17.53 | 17.44 |
| 244 | ARCN1 | Non-matrisome | Non-matrisome | GO:CC not extracellular matrix | sp|P48444|COPD_HUMAN | Coatomer subunit delta | 20.42 | 19.73 | 19.98 |
| 245 | ARF1 | Non-matrisome | Non-matrisome | GO:CC not extracellular matrix | sp|P84077|ARF1_HUMAN | ADP-ribosylation factor 1 | 14.58 | 13.65 | 13.65 |
| 246 | ARF4 | Non-matrisome | Non-matrisome | GO:CC not extracellular matrix | sp|P18085|ARF4_HUMAN | ADP-ribosylation factor 4 | 18.36 | 17.65 | 17.69 |
| 247 | ARF5 | Non-matrisome | Non-matrisome | GO:CC not extracellular matrix | sp|P84085|ARF5_HUMAN | ADP-ribosylation factor 5 | 18.43 | 18.21 | 18.1 |
| 248 | ARF6 | Non-matrisome | Non-matrisome | GO:CC not extracellular matrix | sp|P62330|ARF6_HUMAN | ADP-ribosylation factor 6 | 18.57 | 19.09 | 19.23 |
| 249 | ARFGAP2 | Non-matrisome | Non-matrisome | GO:CC not extracellular matrix | sp|Q8N6H7|ARFG2_HUMAN | ADP-ribosylation factor GTPase-activating protein 2 | 18.62 | 17.94 | 18.19 |
| 250 | ARFGAP3 | Non-matrisome | Non-matrisome | GO:CC not extracellular matrix | sp|Q9NP61|ARFG3_HUMAN | ADP-ribosylation factor GTPase-activating protein 3 | 18.35 | 17.39 | 17.6 |
| 251 | ARFGEF1 | Non-matrisome | Non-matrisome | GO:CC not extracellular matrix | sp|Q9Y6D6|BIG1_HUMAN | Brefeldin A-inhibited guanine nucleotide-exchange protein 1 | 16.93 | 16.92 | 16.91 |
| 252 | ARFIP1 | Non-matrisome | Non-matrisome | GO:CC not extracellular matrix | sp|P53367|ARFP1_HUMAN | Arfaptin-1 | 18.65 | 18.52 | 18.64 |
| 253 | ARFIP2 | Non-matrisome | Non-matrisome | GO:CC not extracellular matrix | sp|P53365|ARFP2_HUMAN | Arfaptin-2 | 17.78 | 17.58 | 17.55 |
| 254 | ARG1 | Non-matrisome | Non-matrisome | GO:CC extracellular matrix (GO:0005576, GO:0005578 & GO:0031012) & GO:CC not extracellular matrix | sp|P05089|ARGI1_HUMAN | Arginase-1 | 18.9 | 19.12 | 18.83 |
| 255 | ARHGAP1 | Non-matrisome | Non-matrisome | GO:CC not extracellular matrix | sp|Q07960|RHG01_HUMAN | Rho GTPase-activating protein 1 | 18.81 | 18.71 | 18.6 |
| 256 | ARHGAP17 | Non-matrisome | Non-matrisome | GO:CC not extracellular matrix | sp|Q68EM7|RHG17_HUMAN | Rho GTPase-activating protein 17 | 17.35 | 17.31 | 17.35 |
| 257 | ARHGAP23 | Non-matrisome | Non-matrisome | GO:CC not extracellular matrix | sp|Q9P227|RHG23_HUMAN | Rho GTPase-activating protein 23 | 16.1 | 15.44 | 15.52 |
| 258 | ARHGAP26 | Non-matrisome | Non-matrisome | GO:CC not extracellular matrix | sp|Q9UNA1|RHG26_HUMAN | Rho GTPase-activating protein 26 | 15.58 | 16.23 | 16.26 |
| 259 | ARHGAP29 | Non-matrisome | Non-matrisome | GO:CC not extracellular matrix | sp|Q52LW3|RHG29_HUMAN | Rho GTPase-activating protein 29 | 17.82 | 17.28 | 17.46 |
| 260 | ARHGAP32 | Non-matrisome | Non-matrisome | GO:CC not extracellular matrix | sp|A7KAX9|RHG32_HUMAN | Rho GTPase-activating protein 32 | 15.2 | 15.37 | 15.44 |
| 261 | ARHGAP42 | Non-matrisome | Non-matrisome | GO:CC not extracellular matrix | sp|A6NI28|RHG42_HUMAN | Rho GTPase-activating protein 42 | 14.58 | 15.26 | 14.97 |
| 262 | ARHGAP5 | Non-matrisome | Non-matrisome | GO:CC not extracellular matrix | sp|Q13017|RHG05_HUMAN | Rho GTPase-activating protein 5 | 17.81 | 17.44 | 17.41 |
| 263 | ARHGDIA | Non-matrisome | Non-matrisome | GO:CC not extracellular matrix | sp|P52565|GDIR1_HUMAN | Rho GDP-dissociation inhibitor 1 | 19.68 | 19.5 | 19.55 |
| 264 | ARHGDIB | Non-matrisome | Non-matrisome | GO:CC not extracellular matrix | sp|P52566|GDIR2_HUMAN | Rho GDP-dissociation inhibitor 2 | 20.19 | 18.95 | 19.02 |
| 265 | ARHGEF1 | Non-matrisome | Non-matrisome | GO:CC not extracellular matrix | sp|Q92888|ARHG1_HUMAN | Rho guanine nucleotide exchange factor 1 | 18.2 | 17.89 | 17.87 |
| 266 | ARHGEF10L | Non-matrisome | Non-matrisome | GO:CC not extracellular matrix | sp|Q9HCE6|ARGAL_HUMAN | Rho guanine nucleotide exchange factor 10-like protein | 13.28 | 13.76 | 13.75 |
| 267 | ARHGEF12 | Non-matrisome | Non-matrisome | GO:CC not extracellular matrix | sp|Q9NZN5|ARHGC_HUMAN | Rho guanine nucleotide exchange factor 12 | 17.01 | 17.02 | 17.13 |
| 268 | ARHGEF2 | Non-matrisome | Non-matrisome | GO:CC not extracellular matrix | sp|Q92974|ARHG2_HUMAN | Rho guanine nucleotide exchange factor 2 | 17.96 | 17.34 | 17.13 |
| 269 | ARHGEF6 | Non-matrisome | Non-matrisome | GO:CC not extracellular matrix | sp|Q15052|ARHG6_HUMAN | Rho guanine nucleotide exchange factor 6 | 16.98 | 17.29 | 17.19 |
| 270 | ARHGEF7 | Non-matrisome | Non-matrisome | GO:CC not extracellular matrix | sp|Q14155|ARHG7_HUMAN | Rho guanine nucleotide exchange factor 7 | 17.42 | 17.6 | 17.4 |
| 271 | ARIH1 | Non-matrisome | Non-matrisome | GO:CC not extracellular matrix | sp|Q9Y4X5|ARI1_HUMAN | E3 ubiquitin-protein ligase ARIH1 | 18.48 | 18.68 | 18.66 |
| 272 | ARL1 | Non-matrisome | Non-matrisome | GO:CC not extracellular matrix | sp|P40616|ARL1_HUMAN | ADP-ribosylation factor-like protein 1 | 17.05 | 16.82 | 17.06 |
| 273 | ARL2 | Non-matrisome | Non-matrisome | GO:CC not extracellular matrix | sp|P36404|ARL2_HUMAN | ADP-ribosylation factor-like protein 2 | 18.48 | 18.4 | 18.46 |
| 274 | ARL3 | Non-matrisome | Non-matrisome | GO:CC not extracellular matrix | sp|P36405|ARL3_HUMAN | ADP-ribosylation factor-like protein 3 | 20.18 | 20.42 | 20.29 |
| 275 | ARL6IP1 | Non-matrisome | Non-matrisome | GO:CC not extracellular matrix | sp|Q15041|AR6P1_HUMAN | ADP-ribosylation factor-like protein 6-interacting protein 1 | 18.72 | 18.94 | 18.36 |
| 276 | ARL6IP5 | Non-matrisome | Non-matrisome | GO:CC not extracellular matrix | sp|O75915|PRAF3_HUMAN | PRA1 family protein 3 | 17.86 | 18.89 | 18.68 |
| 277 | ARL8A | Non-matrisome | Non-matrisome | GO:CC not extracellular matrix | sp|Q96BM9|ARL8A_HUMAN | ADP-ribosylation factor-like protein 8A | 17.39 | 18.37 | 18.72 |
| 278 | ARL8B | Non-matrisome | Non-matrisome | GO:CC not extracellular matrix | sp|Q9NVJ2|ARL8B_HUMAN | ADP-ribosylation factor-like protein 8B | 18.96 | 19.7 | 19.87 |
| 279 | ARMC10 | Non-matrisome | Non-matrisome | GO:CC not extracellular matrix | sp|Q8N2F6|ARM10_HUMAN | Armadillo repeat-containing protein 10 | 14.43 | 14.62 | 14.5 |
| 280 | ARMC5 | Non-matrisome | Non-matrisome | GO:CC not extracellular matrix | sp|Q96C12|ARMC5_HUMAN | Armadillo repeat-containing protein 5 | 16.64 | 18.03 | 17.78 |
| 281 | ARMC6 | Non-matrisome | Non-matrisome | GO:CC not extracellular matrix | sp|Q6NXE6|ARMC6_HUMAN | Armadillo repeat-containing protein 6 | 18.17 | 17.29 | 17.45 |
| 282 | ARMC8 | Non-matrisome | Non-matrisome | GO:CC extracellular matrix (GO:0005576, GO:0005578 & GO:0031012) & GO:CC not extracellular matrix | sp|Q8IUR7|ARMC8_HUMAN | Armadillo repeat-containing protein 8 | 17.28 | 16.5 | 16.62 |
| 283 | ARMC9 | Non-matrisome | Non-matrisome | GO:CC not extracellular matrix | sp|Q7Z3E5|ARMC9_HUMAN | LisH domain-containing protein ARMC9 | 15.87 | 16.47 | 16.67 |
| 284 | ARMCX1 | Non-matrisome | Non-matrisome | GO:CC not extracellular matrix | sp|Q9P291|ARMX1_HUMAN | Armadillo repeat-containing X-linked protein 1 | 18.1 | 18.56 | 18.75 |
| 285 | ARMH3 | Non-matrisome | Non-matrisome | GO:CC not extracellular matrix | sp|Q5T2E6|ARMD3_HUMAN | Armadillo-like helical domain-containing protein 3 | 17.62 | 17.71 | 17.74 |
| 286 | ARMT1 | Non-matrisome | Non-matrisome | GO:CC not extracellular matrix | sp|Q9H993|ARMT1_HUMAN | Damage-control phosphatase ARMT1 | 18.18 | 18.35 | 18.36 |
| 287 | ARPC1A | Non-matrisome | Non-matrisome | GO:CC not extracellular matrix | sp|Q92747|ARC1A_HUMAN | Actin-related protein 2/3 complex subunit 1A | 20.54 | 20.52 | 20.24 |
| 288 | ARPC1B | Non-matrisome | Non-matrisome | GO:CC not extracellular matrix | sp|O15143|ARC1B_HUMAN | Actin-related protein 2/3 complex subunit 1B | 18.69 | 19.03 | 18.96 |
| 289 | ARPC2 | Non-matrisome | Non-matrisome | GO:CC not extracellular matrix | sp|O15144|ARPC2_HUMAN | Actin-related protein 2/3 complex subunit 2 | 19.82 | 19.91 | 19.85 |
| 290 | ARPC3 | Non-matrisome | Non-matrisome | GO:CC not extracellular matrix | sp|O15145|ARPC3_HUMAN | Actin-related protein 2/3 complex subunit 3 | 21.71 | 21.86 | 21.81 |
| 291 | ARPC4 | Non-matrisome | Non-matrisome | GO:CC not extracellular matrix | sp|P59998|ARPC4_HUMAN | Actin-related protein 2/3 complex subunit 4 | 20.61 | 20.62 | 20.58 |
| 292 | ARPC5 | Non-matrisome | Non-matrisome | GO:CC extracellular matrix (GO:0005576, GO:0005578 & GO:0031012) & GO:CC not extracellular matrix | sp|O15511|ARPC5_HUMAN | Actin-related protein 2/3 complex subunit 5 | 17.45 | 17.76 | 17.65 |
| 293 | ARPC5L | Non-matrisome | Non-matrisome | GO:CC not extracellular matrix | sp|Q9BPX5|ARP5L_HUMAN | Actin-related protein 2/3 complex subunit 5-like protein | 17.27 | 17.36 | 17.4 |
| 294 | ARRB1 | Non-matrisome | Non-matrisome | GO:CC not extracellular matrix | sp|P49407|ARRB1_HUMAN | Beta-arrestin-1 | 15.85 | 17.12 | 17.47 |
| 295 | ARRB2 | Non-matrisome | Non-matrisome | GO:CC not extracellular matrix | sp|P32121|ARRB2_HUMAN | Beta-arrestin-2 | 15.49 | 15.48 | 15.54 |
| 296 | ARSA | Non-matrisome | Non-matrisome | GO:CC extracellular matrix (GO:0005576, GO:0005578 & GO:0031012) & GO:CC not extracellular matrix | sp|P15289|ARSA_HUMAN | Arylsulfatase A | 16.09 | 17.82 | 18.31 |
| 297 | ARSB | Non-matrisome | Non-matrisome | GO:CC extracellular matrix (GO:0005576, GO:0005578 & GO:0031012) & GO:CC not extracellular matrix | sp|P15848|ARSB_HUMAN | Arylsulfatase B | 16.93 | 18.16 | 18.77 |
| 298 | ASAH1 | Non-matrisome | Non-matrisome | GO:CC extracellular matrix (GO:0005576, GO:0005578 & GO:0031012) & GO:CC not extracellular matrix | sp|Q13510|ASAH1_HUMAN | Acid ceramidase | 16.44 | 17.95 | 18.68 |
| 299 | ASCC1 | Non-matrisome | Non-matrisome | GO:CC not extracellular matrix | sp|Q8N9N2|ASCC1_HUMAN | Activating signal cointegrator 1 complex subunit 1 | 16.64 | 16.45 | 16.4 |
| 300 | ASCC3 | Non-matrisome | Non-matrisome | GO:CC not extracellular matrix | sp|Q8N3C0|ASCC3_HUMAN | Activating signal cointegrator 1 complex subunit 3 | 18.04 | 17.66 | 17.62 |
| 301 | ASF1A | Non-matrisome | Non-matrisome | GO:CC not extracellular matrix | sp|Q9Y294|ASF1A_HUMAN | Histone chaperone ASF1A | 18.95 | 18.22 | 18.62 |
| 302 | ASH2L | Non-matrisome | Non-matrisome | GO:CC not extracellular matrix | sp|Q9UBL3|ASH2L_HUMAN | Set1/Ash2 histone methyltransferase complex subunit ASH2 | 17.57 | 17.54 | 17.45 |
| 303 | ASMTL | Non-matrisome | Non-matrisome | GO:CC not extracellular matrix | sp|O95671|ASML_HUMAN | Probable bifunctional dTTP/UTP pyrophosphatase/methyltransferase protein | 18.62 | 18.98 | 18.97 |
| 304 | ASNS | Non-matrisome | Non-matrisome | GO:CC not extracellular matrix | sp|P08243|ASNS_HUMAN | Asparagine synthetase [glutamine-hydrolyzing] | 20.02 | 18.11 | 17.84 |
| 305 | ASPH | Non-matrisome | Non-matrisome | GO:CC not extracellular matrix | sp|Q12797|ASPH_HUMAN | Aspartyl/asparaginyl beta-hydroxylase | 18.7 | 19.19 | 18.94 |
| 306 | ASPM | Non-matrisome | Non-matrisome | GO:CC not extracellular matrix | sp|Q8IZT6|ASPM_HUMAN | Abnormal spindle-like microcephaly-associated protein | 16.79 | 15.78 | 15.97 |
| 307 | ASS1 | Non-matrisome | Non-matrisome | GO:CC not extracellular matrix | sp|P00966|ASSY_HUMAN | Argininosuccinate synthase | 19.89 | 21.38 | 21.76 |
| 308 | ATAD3A | Non-matrisome | Non-matrisome | GO:CC not extracellular matrix | sp|Q9NVI7|ATD3A_HUMAN | ATPase family AAA domain-containing protein 3A | 18.78 | 18.81 | 18.32 |
| 309 | ATF1 | Non-matrisome | Non-matrisome | GO:CC not extracellular matrix | sp|P18846|ATF1_HUMAN | Cyclic AMP-dependent transcription factor ATF-1 | 20.41 | 20.02 | 20.16 |
| 310 | ATG3 | Non-matrisome | Non-matrisome | GO:CC not extracellular matrix | sp|Q9NT62|ATG3_HUMAN | Ubiquitin-like-conjugating enzyme ATG3 | 18.87 | 18.4 | 18.43 |
| 311 | ATG4B | Non-matrisome | Non-matrisome | GO:CC not extracellular matrix | sp|Q9Y4P1|ATG4B_HUMAN | Cysteine protease ATG4B | 17.04 | 16.19 | 16.47 |
| 312 | ATG5 | Non-matrisome | Non-matrisome | GO:CC not extracellular matrix | sp|Q9H1Y0|ATG5_HUMAN | Autophagy protein 5 | 17.88 | 18.08 | 18.2 |
| 313 | ATG7 | Non-matrisome | Non-matrisome | GO:CC extracellular matrix (GO:0005576, GO:0005578 & GO:0031012) & GO:CC not extracellular matrix | sp|O95352|ATG7_HUMAN | Ubiquitin-like modifier-activating enzyme ATG7 | 17.3 | 17.47 | 17.31 |
| 314 | ATG9A | Non-matrisome | Non-matrisome | GO:CC not extracellular matrix | sp|Q7Z3C6|ATG9A_HUMAN | Autophagy-related protein 9A | 15.76 | 15.51 | 15.51 |
| 315 | ATIC | Non-matrisome | Non-matrisome | GO:CC not extracellular matrix | sp|P31939|PUR9_HUMAN | Bifunctional purine biosynthesis protein ATIC | 18.81 | 18.75 | 18.63 |
| 316 | ATL2 | Non-matrisome | Non-matrisome | GO:CC not extracellular matrix | sp|Q8NHH9|ATLA2_HUMAN | Atlastin-2 | 17.96 | 17.69 | 17.57 |
| 317 | ATL3 | Non-matrisome | Non-matrisome | GO:CC not extracellular matrix | sp|Q6DD88|ATLA3_HUMAN | Atlastin-3 | 18.38 | 18.43 | 18.22 |
| 318 | ATM | Non-matrisome | Non-matrisome | GO:CC not extracellular matrix | sp|Q13315|ATM_HUMAN | Serine-protein kinase ATM | 16.39 | 17.08 | 17.2 |
| 319 | ATOX1 | Non-matrisome | Non-matrisome | GO:CC not extracellular matrix | sp|O00244|ATOX1_HUMAN | Copper transport protein ATOX1 | 20.72 | 20.37 | 20.77 |
| 320 | ATP13A1 | Non-matrisome | Non-matrisome | GO:CC not extracellular matrix | sp|Q9HD20|AT131_HUMAN | Endoplasmic reticulum transmembrane helix translocase | 17.66 | 17.7 | 17.69 |
| 321 | ATP13A3 | Non-matrisome | Non-matrisome | GO:CC not extracellular matrix | sp|Q9H7F0|AT133_HUMAN | Polyamine-transporting ATPase 13A3 | 16.47 | 15.4 | 15.37 |
| 322 | ATP1A1 | Non-matrisome | Non-matrisome | GO:CC not extracellular matrix | sp|P05023|AT1A1_HUMAN | Sodium/potassium-transporting ATPase subunit alpha-1 | 18.11 | 18.66 | 18.87 |
| 323 | ATP1B1 | Non-matrisome | Non-matrisome | GO:CC not extracellular matrix | sp|P05026|AT1B1_HUMAN | Sodium/potassium-transporting ATPase subunit beta-1 | 19.2 | 18.96 | 18.52 |
| 324 | ATP1B3 | Non-matrisome | Non-matrisome | GO:CC not extracellular matrix | sp|P54709|AT1B3_HUMAN | Sodium/potassium-transporting ATPase subunit beta-3 | 18.62 | 19.13 | 19.49 |
| 325 | ATP2A2 | Non-matrisome | Non-matrisome | GO:CC not extracellular matrix | sp|P16615|AT2A2_HUMAN | Sarcoplasmic/endoplasmic reticulum calcium ATPase 2 | 19 | 19.15 | 19.07 |
| 326 | ATP2B1 | Non-matrisome | Non-matrisome | GO:CC not extracellular matrix | sp|P20020|AT2B1_HUMAN | Plasma membrane calcium-transporting ATPase 1 | 18.03 | 18.53 | 18.56 |
| 327 | ATP2B4 | Non-matrisome | Non-matrisome | GO:CC not extracellular matrix | sp|P23634|AT2B4_HUMAN | Plasma membrane calcium-transporting ATPase 4 | 17.74 | 18.27 | 17.82 |
| 328 | ATP5F1A | Non-matrisome | Non-matrisome | GO:CC not extracellular matrix | sp|P25705|ATPA_HUMAN | ATP synthase subunit alpha, mitochondrial | 19.34 | 20.13 | 19.98 |
| 329 | ATP5F1B | Non-matrisome | Non-matrisome | GO:CC not extracellular matrix | sp|P06576|ATPB_HUMAN | ATP synthase subunit beta, mitochondrial | 19.15 | 19.89 | 19.82 |
| 330 | ATP5F1C | Non-matrisome | Non-matrisome | GO:CC not extracellular matrix | sp|P36542|ATPG_HUMAN | ATP synthase subunit gamma, mitochondrial | 18.88 | 19.83 | 19.56 |
| 331 | ATP5F1D | Non-matrisome | Non-matrisome | GO:CC not extracellular matrix | sp|P30049|ATPD_HUMAN | ATP synthase subunit delta, mitochondrial | 19.68 | 20.28 | 20.19 |
| 332 | ATP5F1E | Non-matrisome | Non-matrisome | GO:CC not extracellular matrix | sp|P56381|ATP5E_HUMAN | ATP synthase subunit epsilon, mitochondrial | 16.25 | 17.38 | 16.92 |
| 333 | ATP5IF1 | Non-matrisome | Non-matrisome | GO:CC not extracellular matrix | sp|Q9UII2|ATIF1_HUMAN | ATPase inhibitor, mitochondrial | 22.21 | 22.48 | 22.3 |
| 334 | ATP5ME | Non-matrisome | Non-matrisome | GO:CC not extracellular matrix | sp|P56385|ATP5I_HUMAN | ATP synthase subunit e, mitochondrial | 19.43 | 20.14 | 20.02 |
| 335 | ATP5MF | Non-matrisome | Non-matrisome | GO:CC not extracellular matrix | sp|P56134|ATPK_HUMAN | ATP synthase subunit f, mitochondrial | 18.66 | 19.45 | 19.17 |
| 336 | ATP5MG | Non-matrisome | Non-matrisome | GO:CC not extracellular matrix | sp|O75964|ATP5L_HUMAN | ATP synthase subunit g, mitochondrial | 18.5 | 19.31 | 19.1 |
| 337 | ATP5PB | Non-matrisome | Non-matrisome | GO:CC not extracellular matrix | sp|P24539|AT5F1_HUMAN | ATP synthase F(0) complex subunit B1, mitochondrial | 18.71 | 19.34 | 19.35 |
| 338 | ATP5PD | Non-matrisome | Non-matrisome | GO:CC not extracellular matrix | sp|O75947|ATP5H_HUMAN | ATP synthase subunit d, mitochondrial | 18.04 | 18.82 | 18.8 |
| 339 | ATP5PF | Non-matrisome | Non-matrisome | GO:CC not extracellular matrix | sp|P18859|ATP5J_HUMAN | ATP synthase-coupling factor 6, mitochondrial | 16.63 | 17.18 | 17.16 |
| 340 | ATP5PO | Non-matrisome | Non-matrisome | GO:CC not extracellular matrix | sp|P48047|ATPO_HUMAN | ATP synthase subunit O, mitochondrial | 18.57 | 19.37 | 19.26 |
| 341 | ATP6AP1 | Non-matrisome | Non-matrisome | GO:CC not extracellular matrix | sp|Q15904|VAS1_HUMAN | V-type proton ATPase subunit S1 | 17.52 | 17.64 | 17.92 |
| 342 | ATP6V0A1 | Non-matrisome | Non-matrisome | GO:CC not extracellular matrix | sp|Q93050|VPP1_HUMAN | V-type proton ATPase 116 kDa subunit a 1 | 16.97 | 18.05 | 18.29 |
| 343 | ATP6V0C | Non-matrisome | Non-matrisome | GO:CC not extracellular matrix | sp|P27449|VATL_HUMAN | V-type proton ATPase 16 kDa proteolipid subunit c | 16.52 | 17.41 | 17.68 |
| 344 | ATP6V0D1 | Non-matrisome | Non-matrisome | GO:CC not extracellular matrix | sp|P61421|VA0D1_HUMAN | V-type proton ATPase subunit d 1 | 17.62 | 18.44 | 18.66 |
| 345 | ATP6V1A | Non-matrisome | Non-matrisome | GO:CC not extracellular matrix | sp|P38606|VATA_HUMAN | V-type proton ATPase catalytic subunit A | 17.52 | 18.18 | 18.36 |
| 346 | ATP6V1B2 | Non-matrisome | Non-matrisome | GO:CC not extracellular matrix | sp|P21281|VATB2_HUMAN | V-type proton ATPase subunit B, brain isoform | 18.29 | 18.86 | 19.04 |
| 347 | ATP6V1C1 | Non-matrisome | Non-matrisome | GO:CC not extracellular matrix | sp|P21283|VATC1_HUMAN | V-type proton ATPase subunit C 1 | 18.6 | 19.23 | 19.31 |
| 348 | ATP6V1D | Non-matrisome | Non-matrisome | GO:CC not extracellular matrix | sp|Q9Y5K8|VATD_HUMAN | V-type proton ATPase subunit D | 18.81 | 19.15 | 19.33 |
| 349 | ATP6V1E1 | Non-matrisome | Non-matrisome | GO:CC not extracellular matrix | sp|P36543|VATE1_HUMAN | V-type proton ATPase subunit E 1 | 18.98 | 19.61 | 19.75 |
| 350 | ATP6V1F | Non-matrisome | Non-matrisome | GO:CC not extracellular matrix | sp|Q16864|VATF_HUMAN | V-type proton ATPase subunit F | 20.59 | 20.74 | 20.88 |
| 351 | ATP6V1G1 | Non-matrisome | Non-matrisome | GO:CC not extracellular matrix | sp|O75348|VATG1_HUMAN | V-type proton ATPase subunit G 1 | 19.47 | 20.04 | 20.23 |
| 352 | ATP6V1H | Non-matrisome | Non-matrisome | GO:CC not extracellular matrix | sp|Q9UI12|VATH_HUMAN | V-type proton ATPase subunit H | 18.08 | 18.6 | 18.8 |
| 353 | ATP7A | Non-matrisome | Non-matrisome | GO:CC not extracellular matrix | sp|Q04656|ATP7A_HUMAN | Copper-transporting ATPase 1 | 15.76 | 16.48 | 16.5 |
| 354 | ATRN | Non-matrisome | Non-matrisome | GO:CC not extracellular matrix | sp|O75882|ATRN_HUMAN | Attractin | 17.19 | 17.12 | 17.27 |
| 355 | ATRX | Non-matrisome | Non-matrisome | GO:CC not extracellular matrix | sp|P46100|ATRX_HUMAN | Transcriptional regulator ATRX | 16.26 | 16.81 | 16.8 |
| 356 | ATXN10 | Non-matrisome | Non-matrisome | GO:CC not extracellular matrix | sp|Q9UBB4|ATX10_HUMAN | Ataxin-10 | 17.9 | 17.18 | 17.44 |
| 357 | ATXN2L | Non-matrisome | Non-matrisome | GO:CC not extracellular matrix | sp|Q8WWM7|ATX2L_HUMAN | Ataxin-2-like protein | 18.99 | 18.27 | 18.36 |
| 358 | ATXN3 | Non-matrisome | Non-matrisome | GO:CC not extracellular matrix | sp|P54252|ATX3_HUMAN | Ataxin-3 | 16.5 | 16.63 | 16.6 |
| 359 | AUP1 | Non-matrisome | Non-matrisome | GO:CC not extracellular matrix | sp|Q9Y679|AUP1_HUMAN | Lipid droplet-regulating VLDL assembly factor AUP1 | 18.82 | 18.67 | 18.56 |
| 360 | AVEN | Non-matrisome | Non-matrisome | GO:CC not extracellular matrix | sp|Q9NQS1|AVEN_HUMAN | Cell death regulator Aven | 16.4 | 16.16 | 16.16 |
| 361 | B2M | Non-matrisome | Non-matrisome | GO:CC extracellular matrix (GO:0005576, GO:0005578 & GO:0031012) & GO:CC not extracellular matrix | sp|P61769|B2MG_HUMAN | Beta-2-microglobulin | 17.49 | 17.97 | 17.89 |
| 362 | B3GLCT | Non-matrisome | Non-matrisome | GO:CC not extracellular matrix | sp|Q6Y288|B3GLT_HUMAN | Beta-1,3-glucosyltransferase | 17.34 | 17.51 | 17.43 |
| 363 | BABAM2 | Non-matrisome | Non-matrisome | GO:CC not extracellular matrix | sp|Q9NXR7|BABA2_HUMAN | BRISC and BRCA1-A complex member 2 | 17.67 | 18.07 | 17.81 |
| 364 | BAG2 | Non-matrisome | Non-matrisome | GO:CC not extracellular matrix | sp|O95816|BAG2_HUMAN | BAG family molecular chaperone regulator 2 | 17.86 | 17.79 | 17.37 |
| 365 | BAG3 | Non-matrisome | Non-matrisome | GO:CC not extracellular matrix | sp|O95817|BAG3_HUMAN | BAG family molecular chaperone regulator 3 | 18.57 | 17.62 | 18.3 |
| 366 | BAG5 | Non-matrisome | Non-matrisome | GO:CC not extracellular matrix | sp|Q9UL15|BAG5_HUMAN | BAG family molecular chaperone regulator 5 | 16.53 | 16.48 | 16.54 |
| 367 | BAG6 | Non-matrisome | Non-matrisome | GO:CC not extracellular matrix | sp|P46379|BAG6_HUMAN | Large proline-rich protein BAG6 | 17.55 | 17.09 | 17.2 |
| 368 | BAIAP2 | Non-matrisome | Non-matrisome | GO:CC not extracellular matrix | sp|Q9UQB8|BAIP2_HUMAN | Brain-specific angiogenesis inhibitor 1-associated protein 2 | 18.28 | 17.1 | 17.04 |
| 369 | BAIAP2L1 | Non-matrisome | Non-matrisome | GO:CC not extracellular matrix | sp|Q9UHR4|BI2L1_HUMAN | Brain-specific angiogenesis inhibitor 1-associated protein 2-like protein 1 | 17.33 | 16.4 | 16.34 |
| 370 | BANF1 | Non-matrisome | Non-matrisome | GO:CC not extracellular matrix | sp|O75531|BAF_HUMAN | Barrier-to-autointegration factor | 16.76 | 17.32 | 17.36 |
| 371 | BAP18 | Non-matrisome | Non-matrisome | GO:CC not extracellular matrix | sp|Q8IXM2|BAP18_HUMAN | Chromatin complexes subunit BAP18 | 16.46 | 16.97 | 17.07 |
| 372 | BASP1 | Non-matrisome | Non-matrisome | GO:CC not extracellular matrix | sp|P80723|BASP1_HUMAN | Brain acid soluble protein 1 | 18.68 | 19.3 | 19.17 |
| 373 | BAX | Non-matrisome | Non-matrisome | GO:CC not extracellular matrix | sp|Q07812|BAX_HUMAN | Apoptosis regulator BAX | 18.37 | 18.91 | 18.92 |
| 374 | BAZ1B | Non-matrisome | Non-matrisome | GO:CC not extracellular matrix | sp|Q9UIG0|BAZ1B_HUMAN | Tyrosine-protein kinase BAZ1B | 17.33 | 16.93 | 17.02 |
| 375 | BCAM | Non-matrisome | Non-matrisome | GO:CC extracellular matrix (GO:0005576, GO:0005578 & GO:0031012) & GO:CC not extracellular matrix | sp|P50895|BCAM_HUMAN | Basal cell adhesion molecule | 14.68 | 15.17 | 14.95 |
| 376 | BCAP31 | Non-matrisome | Non-matrisome | GO:CC not extracellular matrix | sp|P51572|BAP31_HUMAN | B-cell receptor-associated protein 31 | 19.94 | 19.98 | 19.94 |
| 377 | BCAS2 | Non-matrisome | Non-matrisome | GO:CC extracellular matrix (GO:0005576, GO:0005578 & GO:0031012) & GO:CC not extracellular matrix | sp|O75934|SPF27_HUMAN | Pre-mRNA-splicing factor SPF27 | 18.84 | 18.78 | 18.77 |
| 378 | BCAT2 | Non-matrisome | Non-matrisome | GO:CC not extracellular matrix | sp|O15382|BCAT2_HUMAN | Branched-chain-amino-acid aminotransferase, mitochondrial | 17.38 | 17.63 | 17.12 |
| 379 | BCCIP | Non-matrisome | Non-matrisome | GO:CC not extracellular matrix | sp|Q9P287|BCCIP_HUMAN | BRCA2 and CDKN1A-interacting protein | 19.35 | 18.48 | 18.33 |
| 380 | BCKDHA | Non-matrisome | Non-matrisome | GO:CC not extracellular matrix | sp|P12694|ODBA_HUMAN | 2-oxoisovalerate dehydrogenase subunit alpha, mitochondrial | 17.56 | 18.08 | 17.82 |
| 381 | BCKDHB | Non-matrisome | Non-matrisome | GO:CC not extracellular matrix | sp|P21953|ODBB_HUMAN | 2-oxoisovalerate dehydrogenase subunit beta, mitochondrial | 17.17 | 17.73 | 17.41 |
| 382 | BCKDK | Non-matrisome | Non-matrisome | GO:CC not extracellular matrix | sp|O14874|BCKD_HUMAN | [3-methyl-2-oxobutanoate dehydrogenase [lipoamide]] kinase, mitochondrial | 15.38 | 16.29 | 15.87 |
| 383 | BCL10 | Non-matrisome | Non-matrisome | GO:CC not extracellular matrix | sp|O95999|BCL10_HUMAN | B-cell lymphoma/leukemia 10 | 18.17 | 17.49 | 17.55 |
| 384 | BCL2L13 | Non-matrisome | Non-matrisome | GO:CC not extracellular matrix | sp|Q9BXK5|B2L13_HUMAN | Bcl-2-like protein 13 | 17.07 | 17.59 | 17.5 |
| 385 | BCLAF1 | Non-matrisome | Non-matrisome | GO:CC not extracellular matrix | sp|Q9NYF8|BCLF1_HUMAN | Bcl-2-associated transcription factor 1 | 18.25 | 18.33 | 18.25 |
| 386 | BDH1 | Non-matrisome | Non-matrisome | GO:CC not extracellular matrix | sp|Q02338|BDH_HUMAN | D-beta-hydroxybutyrate dehydrogenase, mitochondrial | 15.58 | 16.7 | 16.33 |
| 387 | BDH2 | Non-matrisome | Non-matrisome | GO:CC not extracellular matrix | sp|Q9BUT1|DHRS6_HUMAN | Dehydrogenase/reductase SDR family member 6 | 17.68 | 19.11 | 19.42 |
| 388 | BGN | Core matrisome | Proteoglycans | GO:CC extracellular matrix (GO:0005576, GO:0005578 & GO:0031012) & GO:CC not extracellular matrix | sp|P21810|PGS1_HUMAN | Biglycan | 17.88 | 19.99 | 19.92 |
| 389 | BICD2 | Non-matrisome | Non-matrisome | GO:CC not extracellular matrix | sp|Q8TD16|BICD2_HUMAN | Protein bicaudal D homolog 2 | 17.81 | 16.81 | 16.96 |
| 390 | BIN1 | Non-matrisome | Non-matrisome | GO:CC not extracellular matrix | sp|O00499|BIN1_HUMAN | Myc box-dependent-interacting protein 1 | 16.78 | 17.03 | 17.16 |
| 391 | BIN3 | Non-matrisome | Non-matrisome | GO:CC not extracellular matrix | sp|Q9NQY0|BIN3_HUMAN | Bridging integrator 3 | 17.59 | 17.09 | 17.01 |
| 392 | BIRC6 | Non-matrisome | Non-matrisome | GO:CC not extracellular matrix | sp|Q9NR09|BIRC6_HUMAN | Baculoviral IAP repeat-containing protein 6 | 16.71 | 16.5 | 16.52 |
| 393 | BLMH | Non-matrisome | Non-matrisome | GO:CC not extracellular matrix | sp|Q13867|BLMH_HUMAN | Bleomycin hydrolase | 18.08 | 18.03 | 17.98 |
| 394 | BLOC1S5 | Non-matrisome | Non-matrisome | GO:CC not extracellular matrix | sp|Q8TDH9|BL1S5_HUMAN | Biogenesis of lysosome-related organelles complex 1 subunit 5 | 17.14 | 17.55 | 17.59 |
| 395 | BLVRA | Non-matrisome | Non-matrisome | GO:CC not extracellular matrix | sp|P53004|BIEA_HUMAN | Biliverdin reductase A | 19.1 | 19.17 | 19.27 |
| 396 | BLVRB | Non-matrisome | Non-matrisome | GO:CC not extracellular matrix | sp|P30043|BLVRB_HUMAN | Flavin reductase (NADPH) | 19.97 | 20.4 | 20.38 |
| 397 | BMI1 | Non-matrisome | Non-matrisome | GO:CC not extracellular matrix | sp|P35226|BMI1_HUMAN | Polycomb complex protein BMI-1 | 18.16 | 17.57 | 17.73 |
| 398 | BMP2K | Non-matrisome | Non-matrisome | GO:CC not extracellular matrix | sp|Q9NSY1|BMP2K_HUMAN | BMP-2-inducible protein kinase | 16.35 | 16.3 | 16.44 |
| 399 | BMS1 | Non-matrisome | Non-matrisome | GO:CC not extracellular matrix | sp|Q14692|BMS1_HUMAN | Ribosome biogenesis protein BMS1 homolog | 18.2 | 17.61 | 17.52 |
| 400 | BNIP1 | Non-matrisome | Non-matrisome | GO:CC not extracellular matrix | sp|Q12981|SEC20_HUMAN | Vesicle transport protein SEC20 | 18.2 | 18.15 | 18.03 |
| 401 | BOLA1 | Non-matrisome | Non-matrisome | GO:CC not extracellular matrix | sp|Q9Y3E2|BOLA1_HUMAN | BolA-like protein 1 | 16.25 | 16.04 | 16.04 |
| 402 | BOP1 | Non-matrisome | Non-matrisome | GO:CC not extracellular matrix | sp|Q14137|BOP1_HUMAN | Ribosome biogenesis protein BOP1 | 18.17 | 17.49 | 17.39 |
| 403 | BPGM | Non-matrisome | Non-matrisome | GO:CC not extracellular matrix | sp|P07738|PMGE_HUMAN | Bisphosphoglycerate mutase | 17.22 | 18.01 | 17.81 |
| 404 | BPHL | Non-matrisome | Non-matrisome | GO:CC not extracellular matrix | sp|Q86WA6|BPHL_HUMAN | Valacyclovir hydrolase | 18.04 | 18.7 | 18.71 |
| 405 | BPNT1 | Non-matrisome | Non-matrisome | GO:CC not extracellular matrix | sp|O95861|BPNT1_HUMAN | 3'(2'),5'-bisphosphate nucleotidase 1 | 16.84 | 17.18 | 17.28 |
| 406 | BPNT2 | Non-matrisome | Non-matrisome | GO:CC not extracellular matrix | sp|Q9NX62|IMPA3_HUMAN | Golgi-resident adenosine 3',5'-bisphosphate 3'-phosphatase | 18.65 | 18.22 | 18.39 |
| 407 | BRD4 | Non-matrisome | Non-matrisome | GO:CC not extracellular matrix | sp|O60885|BRD4_HUMAN | Bromodomain-containing protein 4 | 18.03 | 17.97 | 18.02 |
| 408 | BRI3BP | Non-matrisome | Non-matrisome | GO:CC not extracellular matrix | sp|Q8WY22|BRI3B_HUMAN | BRI3-binding protein | 18.99 | 19.35 | 19.41 |
| 409 | BRIX1 | Non-matrisome | Non-matrisome | GO:CC not extracellular matrix | sp|Q8TDN6|BRX1_HUMAN | Ribosome biogenesis protein BRX1 homolog | 19.11 | 18.91 | 18.73 |
| 410 | BRK1 | Non-matrisome | Non-matrisome | GO:CC not extracellular matrix | sp|Q8WUW1|BRK1_HUMAN | Protein BRICK1 | 19.1 | 18.66 | 18.73 |
| 411 | BROX | Non-matrisome | Non-matrisome | GO:CC not extracellular matrix | sp|Q5VW32|BROX_HUMAN | BRO1 domain-containing protein BROX | 19.11 | 19.29 | 19.23 |
| 412 | BSG | Non-matrisome | Non-matrisome | GO:CC not extracellular matrix | sp|P35613|BASI_HUMAN | Basigin | 19.79 | 20.25 | 20.04 |
| 413 | BST1 | Non-matrisome | Non-matrisome | GO:CC extracellular matrix (GO:0005576, GO:0005578 & GO:0031012) & GO:CC not extracellular matrix | sp|Q10588|BST1_HUMAN | ADP-ribosyl cyclase/cyclic ADP-ribose hydrolase 2 | 17.37 | 18.49 | 18.25 |
| 414 | BST2 | Non-matrisome | Non-matrisome | GO:CC not extracellular matrix | sp|Q10589|BST2_HUMAN | Bone marrow stromal antigen 2 | 16.74 | 16.36 | 16.67 |
| 415 | BTF3 | Non-matrisome | Non-matrisome | GO:CC not extracellular matrix | sp|P20290|BTF3_HUMAN | Transcription factor BTF3 | 18.98 | 18.07 | 18.15 |
| 416 | BTF3L4 | Non-matrisome | Non-matrisome | GO:CC not extracellular matrix | sp|Q96K17|BT3L4_HUMAN | Transcription factor BTF3 homolog 4 | 20.73 | 20.81 | 20.33 |
| 417 | BTN3A3 | Non-matrisome | Non-matrisome | GO:CC not extracellular matrix | sp|O00478|BT3A3_HUMAN | Butyrophilin subfamily 3 member A3 | 16.03 | 17.4 | 17.5 |
| 418 | BUB3 | Non-matrisome | Non-matrisome | GO:CC not extracellular matrix | sp|O43684|BUB3_HUMAN | Mitotic checkpoint protein BUB3 | 19.26 | 18.67 | 18.64 |
| 419 | BUD31 | Non-matrisome | Non-matrisome | GO:CC not extracellular matrix | sp|P41223|BUD31_HUMAN | Protein BUD31 homolog | 18.7 | 17.85 | 17.92 |
| 420 | BYSL | Non-matrisome | Non-matrisome | GO:CC not extracellular matrix | sp|Q13895|BYST_HUMAN | Bystin | 19.01 | 17.91 | 18.15 |
| 421 | BZW1 | Non-matrisome | Non-matrisome | GO:CC not extracellular matrix | sp|Q7L1Q6|5MP2_HUMAN | eIF5-mimic protein 2 | 19.89 | 19.37 | 19.25 |
| 422 | BZW2 | Non-matrisome | Non-matrisome | GO:CC not extracellular matrix | sp|Q9Y6E2|5MP1_HUMAN | eIF5-mimic protein 1 | 19.14 | 18.37 | 18.22 |
| 423 | C11orf54 | Non-matrisome | Non-matrisome | GO:CC not extracellular matrix | sp|Q9H0W9|CK054_HUMAN | Ester hydrolase C11orf54 | 15.4 | 16.3 | 16.51 |
| 424 | C11orf68 | Non-matrisome | Non-matrisome | GO:CC not extracellular matrix | sp|Q9H3H3|CK068_HUMAN | UPF0696 protein C11orf68 | 18.06 | 18.09 | 17.95 |
| 425 | C11orf98 | Non-matrisome | Non-matrisome | gene not present in GO IDs | sp|E9PRG8|CK098_HUMAN | Uncharacterized protein C11orf98 | 18.23 | 17.17 | 17.07 |
| 426 | C19orf12 | Non-matrisome | Non-matrisome | GO:CC not extracellular matrix | sp|Q9NSK7|CS012_HUMAN | Protein C19orf12 | 18.15 | 18.28 | 18.28 |
| 427 | C1orf198 | Non-matrisome | Non-matrisome | GO:CC not extracellular matrix | sp|Q9H425|CA198_HUMAN | Uncharacterized protein C1orf198 | 17.66 | 17.94 | 18.42 |
| 428 | C1QBP | Non-matrisome | Non-matrisome | GO:CC extracellular matrix (GO:0005576, GO:0005578 & GO:0031012) & GO:CC not extracellular matrix | sp|Q07021|C1QBP_HUMAN | Complement component 1 Q subcomponent-binding protein, mitochondrial | 17.68 | 17.85 | 17.43 |
| 429 | C1R | Non-matrisome | Non-matrisome | GO:CC extracellular matrix (GO:0005576, GO:0005578 & GO:0031012) & GO:CC not extracellular matrix | sp|P00736|C1R_HUMAN | Complement C1r subcomponent | 15.54 | 16.77 | 16.13 |
| 430 | C1S | Non-matrisome | Non-matrisome | GO:CC extracellular matrix (GO:0005576, GO:0005578 & GO:0031012) & GO:CC not extracellular matrix | sp|P09871|C1S_HUMAN | Complement C1s subcomponent | 13.25 | 15.49 | 15.09 |
| 431 | C2CD5 | Non-matrisome | Non-matrisome | GO:CC not extracellular matrix | sp|Q86YS7|C2CD5_HUMAN | C2 domain-containing protein 5 | 17.04 | 17.33 | 17.31 |
| 432 | C3 | Non-matrisome | Non-matrisome | GO:CC extracellular matrix (GO:0005576, GO:0005578 & GO:0031012) & GO:CC not extracellular matrix | sp|P01024|CO3_HUMAN | Complement C3 | 17.54 | 16.8 | 17.22 |
| 433 | C4BPA | Non-matrisome | Non-matrisome | GO:CC extracellular matrix (GO:0005576, GO:0005578 & GO:0031012) & GO:CC not extracellular matrix | sp|P04003|C4BPA_HUMAN | C4b-binding protein alpha chain | 17.5 | 19.5 | 19.46 |
| 434 | C4orf3 | Non-matrisome | Non-matrisome | GO:CC not extracellular matrix | sp|Q8WVX3|CD003_HUMAN | Uncharacterized protein C4orf3 | 16.48 | 16.23 | 16.07 |
| 435 | C5 | Non-matrisome | Non-matrisome | GO:CC extracellular matrix (GO:0005576, GO:0005578 & GO:0031012) & GO:CC not extracellular matrix | sp|P01031|CO5_HUMAN | Complement C5 | 18.25 | 17.21 | 18.68 |
| 436 | C6 | Non-matrisome | Non-matrisome | GO:CC extracellular matrix (GO:0005576, GO:0005578 & GO:0031012) & GO:CC not extracellular matrix | sp|P13671|CO6_HUMAN | Complement component C6 | 14.8 | 13.82 | 15.38 |
| 437 | C7orf50 | Non-matrisome | Non-matrisome | GO:CC not extracellular matrix | sp|Q9BRJ6|CG050_HUMAN | Uncharacterized protein C7orf50 | 17.68 | 17.27 | 17.03 |
| 438 | C8B | Non-matrisome | Non-matrisome | GO:CC extracellular matrix (GO:0005576, GO:0005578 & GO:0031012) & GO:CC not extracellular matrix | sp|P07358|CO8B_HUMAN | Complement component C8 beta chain | 15.67 | 15.91 | 16.01 |
| 439 | C8orf33 | Non-matrisome | Non-matrisome | GO:CC not extracellular matrix | sp|Q9H7E9|CH033_HUMAN | UPF0488 protein C8orf33 | 18.87 | 17.92 | 18.02 |
| 440 | C8orf82 | Non-matrisome | Non-matrisome | gene not present in GO IDs | sp|Q6P1X6|CH082_HUMAN | UPF0598 protein C8orf82 | 15.72 | 16.49 | 16.49 |
| 441 | C9 | Non-matrisome | Non-matrisome | GO:CC extracellular matrix (GO:0005576, GO:0005578 & GO:0031012) & GO:CC not extracellular matrix | sp|P02748|CO9_HUMAN | Complement component C9 | 16 | 15.51 | 16.79 |
| 442 | CA12 | Non-matrisome | Non-matrisome | GO:CC not extracellular matrix | sp|O43570|CAH12_HUMAN | Carbonic anhydrase 12 | 16.85 | 18.66 | 17.93 |
| 443 | CAB39 | Non-matrisome | Non-matrisome | GO:CC extracellular matrix (GO:0005576, GO:0005578 & GO:0031012) & GO:CC not extracellular matrix | sp|Q9Y376|CAB39_HUMAN | Calcium-binding protein 39 | 16.62 | 16.39 | 16.49 |
| 444 | CACNA2D1 | Non-matrisome | Non-matrisome | GO:CC not extracellular matrix | sp|P54289|CA2D1_HUMAN | Voltage-dependent calcium channel subunit alpha-2/delta-1 | 17.5 | 17.78 | 17.65 |
| 445 | CACYBP | Non-matrisome | Non-matrisome | GO:CC not extracellular matrix | sp|Q9HB71|CYBP_HUMAN | Calcyclin-binding protein | 18.57 | 18.13 | 18.1 |
| 446 | CAD | Non-matrisome | Non-matrisome | GO:CC not extracellular matrix | sp|P27708|PYR1_HUMAN | CAD protein | 18.4 | 17.86 | 17.7 |
| 447 | CALCOCO1 | Non-matrisome | Non-matrisome | GO:CC not extracellular matrix | sp|Q9P1Z2|CACO1_HUMAN | Calcium-binding and coiled-coil domain-containing protein 1 | 17.47 | 17.04 | 16.92 |
| 448 | CALD1 | Non-matrisome | Non-matrisome | GO:CC not extracellular matrix | sp|Q05682|CALD1_HUMAN | Caldesmon | 19.3 | 18.64 | 18.55 |
| 449 | CALHM2 | Non-matrisome | Non-matrisome | GO:CC not extracellular matrix | sp|Q9HA72|CAHM2_HUMAN | Calcium homeostasis modulator protein 2 | 15.78 | 16.47 | 16.56 |
| 450 | CALHM5 | Non-matrisome | Non-matrisome | GO:CC not extracellular matrix | sp|Q8N5C1|CAHM5_HUMAN | Calcium homeostasis modulator protein 5 | 16.39 | 16.57 | 16.37 |
| 451 | CALML5 | Non-matrisome | Non-matrisome | GO:CC extracellular matrix (GO:0005576, GO:0005578 & GO:0031012) & GO:CC not extracellular matrix | sp|Q9NZT1|CALL5_HUMAN | Calmodulin-like protein 5 | 14.57 | 13.85 | 14.46 |
| 452 | CALR | Non-matrisome | Non-matrisome | GO:CC extracellular matrix (GO:0005576, GO:0005578 & GO:0031012) & GO:CC not extracellular matrix | sp|P27797|CALR_HUMAN | Calreticulin | 18.58 | 18.57 | 18.47 |
| 453 | CALU | Non-matrisome | Non-matrisome | GO:CC extracellular matrix (GO:0005576, GO:0005578 & GO:0031012) & GO:CC not extracellular matrix | sp|O43852|CALU_HUMAN | Calumenin | 19.55 | 19.59 | 19.43 |
| 454 | CAMK1D | Non-matrisome | Non-matrisome | GO:CC not extracellular matrix | sp|Q8IU85|KCC1D_HUMAN | Calcium/calmodulin-dependent protein kinase type 1D | 15.2 | 16 | 15.93 |
| 455 | CAMK2D | Non-matrisome | Non-matrisome | GO:CC not extracellular matrix | sp|Q13557|KCC2D_HUMAN | Calcium/calmodulin-dependent protein kinase type II subunit delta | 17.53 | 17.96 | 17.79 |
| 456 | CAND1 | Non-matrisome | Non-matrisome | GO:CC extracellular matrix (GO:0005576, GO:0005578 & GO:0031012) & GO:CC not extracellular matrix | sp|Q86VP6|CAND1_HUMAN | Cullin-associated NEDD8-dissociated protein 1 | 19.26 | 19.62 | 19.63 |
| 457 | CANX | Non-matrisome | Non-matrisome | GO:CC not extracellular matrix | sp|P27824|CALX_HUMAN | Calnexin | 18.78 | 18.96 | 18.78 |
| 458 | CAP1 | Non-matrisome | Non-matrisome | GO:CC extracellular matrix (GO:0005576, GO:0005578 & GO:0031012) & GO:CC not extracellular matrix | sp|Q01518|CAP1_HUMAN | Adenylyl cyclase-associated protein 1 | 19.41 | 19.18 | 19.03 |
| 459 | CAPG | Non-matrisome | Non-matrisome | GO:CC not extracellular matrix | sp|P40121|CAPG_HUMAN | Macrophage-capping protein | 18.22 | 18.79 | 18.82 |
| 460 | CAPN1 | Non-matrisome | Non-matrisome | GO:CC extracellular matrix (GO:0005576, GO:0005578 & GO:0031012) & GO:CC not extracellular matrix | sp|P07384|CAN1_HUMAN | Calpain-1 catalytic subunit | 19.27 | 19.56 | 19.46 |
| 461 | CAPN2 | Non-matrisome | Non-matrisome | GO:CC not extracellular matrix | sp|P17655|CAN2_HUMAN | Calpain-2 catalytic subunit | 19.02 | 18.95 | 18.75 |
| 462 | CAPNS1 | Non-matrisome | Non-matrisome | GO:CC not extracellular matrix | sp|P04632|CPNS1_HUMAN | Calpain small subunit 1 | 18.95 | 18.77 | 18.86 |
| 463 | CAPRIN1 | Non-matrisome | Non-matrisome | GO:CC not extracellular matrix | sp|Q14444|CAPR1_HUMAN | Caprin-1 | 20.14 | 18.61 | 19.04 |
| 464 | CAPS | Non-matrisome | Non-matrisome | GO:CC not extracellular matrix | sp|Q13938|CAYP1_HUMAN | Calcyphosin | 17.64 | 18.55 | 17.99 |
| 465 | CAPZA1 | Non-matrisome | Non-matrisome | GO:CC extracellular matrix (GO:0005576, GO:0005578 & GO:0031012) & GO:CC not extracellular matrix | sp|P52907|CAZA1_HUMAN | F-actin-capping protein subunit alpha-1 | 18.09 | 18.31 | 18.23 |
| 466 | CAPZA2 | Non-matrisome | Non-matrisome | GO:CC extracellular matrix (GO:0005576, GO:0005578 & GO:0031012) & GO:CC not extracellular matrix | sp|P47755|CAZA2_HUMAN | F-actin-capping protein subunit alpha-2 | 18.43 | 18.72 | 18.72 |
| 467 | CAPZB | Non-matrisome | Non-matrisome | GO:CC not extracellular matrix | sp|P47756|CAPZB_HUMAN | F-actin-capping protein subunit beta | 18.97 | 19.19 | 19.07 |
| 468 | CARHSP1 | Non-matrisome | Non-matrisome | GO:CC not extracellular matrix | sp|Q9Y2V2|CHSP1_HUMAN | Calcium-regulated heat-stable protein 1 | 18.74 | 19.08 | 18.91 |
| 469 | CARM1 | Non-matrisome | Non-matrisome | GO:CC not extracellular matrix | sp|Q86X55|CARM1_HUMAN | Histone-arginine methyltransferase CARM1 | 18.32 | 18.35 | 18.12 |
| 470 | CARMIL1 | Non-matrisome | Non-matrisome | GO:CC not extracellular matrix | sp|Q5VZK9|CARL1_HUMAN | F-actin-uncapping protein LRRC16A | 16.56 | 16.27 | 16.16 |
| 471 | CARS1 | Non-matrisome | Non-matrisome | GO:CC not extracellular matrix | sp|P49589|SYCC_HUMAN | Cysteine--tRNA ligase, cytoplasmic | 18.26 | 18.49 | 18.49 |
| 472 | CASK | Non-matrisome | Non-matrisome | GO:CC extracellular matrix (GO:0005576, GO:0005578 & GO:0031012) & GO:CC not extracellular matrix | sp|O14936|CSKP_HUMAN | Peripheral plasma membrane protein CASK | 16.77 | 18 | 17.91 |
| 473 | CASP1 | Non-matrisome | Non-matrisome | GO:CC not extracellular matrix | sp|P29466|CASP1_HUMAN | Caspase-1 | 16.77 | 17.14 | 17.46 |
| 474 | CASP3 | Non-matrisome | Non-matrisome | GO:CC not extracellular matrix | sp|P42574|CASP3_HUMAN | Caspase-3 | 18.76 | 18.46 | 18.11 |
| 475 | CASP4 | Non-matrisome | Non-matrisome | GO:CC extracellular matrix (GO:0005576, GO:0005578 & GO:0031012) & GO:CC not extracellular matrix | sp|P49662|CASP4_HUMAN | Caspase-4 | 18.06 | 17.33 | 17.56 |
| 476 | CASP6 | Non-matrisome | Non-matrisome | GO:CC not extracellular matrix | sp|P55212|CASP6_HUMAN | Caspase-6 | 17.38 | 17.79 | 17.7 |
| 477 | CASP8 | Non-matrisome | Non-matrisome | GO:CC not extracellular matrix | sp|Q14790|CASP8_HUMAN | Caspase-8 | 17.76 | 17.77 | 17.88 |
| 478 | CAST | Non-matrisome | Non-matrisome | GO:CC not extracellular matrix | sp|P20810|ICAL_HUMAN | Calpastatin | 18.18 | 18.22 | 18.32 |
| 479 | CAT | Non-matrisome | Non-matrisome | GO:CC extracellular matrix (GO:0005576, GO:0005578 & GO:0031012) & GO:CC not extracellular matrix | sp|P04040|CATA_HUMAN | Catalase | 17.66 | 18.42 | 18.78 |
| 480 | CAV1 | Non-matrisome | Non-matrisome | GO:CC not extracellular matrix | sp|Q03135|CAV1_HUMAN | Caveolin-1 | 20.16 | 20.49 | 20.18 |
| 481 | CAV2 | Non-matrisome | Non-matrisome | GO:CC not extracellular matrix | sp|P51636|CAV2_HUMAN | Caveolin-2 | 18.76 | 19.23 | 19.02 |
| 482 | CAVIN1 | Non-matrisome | Non-matrisome | GO:CC not extracellular matrix | sp|Q6NZI2|CAVN1_HUMAN | Caveolae-associated protein 1 | 18.93 | 19.32 | 19.11 |
| 483 | CAVIN2 | Non-matrisome | Non-matrisome | GO:CC not extracellular matrix | sp|O95810|CAVN2_HUMAN | Caveolae-associated protein 2 | 15.1 | 15.99 | 15.76 |
| 484 | CAVIN3 | Non-matrisome | Non-matrisome | GO:CC not extracellular matrix | sp|Q969G5|CAVN3_HUMAN | Caveolae-associated protein 3 | 17.38 | 18.24 | 18.09 |
| 485 | CBFB | Non-matrisome | Non-matrisome | GO:CC not extracellular matrix | sp|Q13951|PEBB_HUMAN | Core-binding factor subunit beta | 17.7 | 17.43 | 17.54 |
| 486 | CBL | Non-matrisome | Non-matrisome | GO:CC not extracellular matrix | sp|P22681|CBL_HUMAN | E3 ubiquitin-protein ligase CBL | 18.38 | 17.91 | 18.02 |
| 487 | CBR1 | Non-matrisome | Non-matrisome | GO:CC not extracellular matrix | sp|P16152|CBR1_HUMAN | Carbonyl reductase [NADPH] 1 | 19.19 | 19.58 | 19.63 |
| 488 | CBR3 | Non-matrisome | Non-matrisome | GO:CC not extracellular matrix | sp|O75828|CBR3_HUMAN | Carbonyl reductase [NADPH] 3 | 17.89 | 18.1 | 18 |
| 489 | CBR4 | Non-matrisome | Non-matrisome | GO:CC not extracellular matrix | sp|Q8N4T8|CBR4_HUMAN | 3-oxoacyl-[acyl-carrier-protein] reductase | 15.62 | 16.87 | 16.76 |
| 490 | CBX1 | Non-matrisome | Non-matrisome | GO:CC not extracellular matrix | sp|P83916|CBX1_HUMAN | Chromobox protein homolog 1 | 19.39 | 19.42 | 19.55 |
| 491 | CBX3 | Non-matrisome | Non-matrisome | GO:CC not extracellular matrix | sp|Q13185|CBX3_HUMAN | Chromobox protein homolog 3 | 18.44 | 18.39 | 18.45 |
| 492 | CBX5 | Non-matrisome | Non-matrisome | GO:CC not extracellular matrix | sp|P45973|CBX5_HUMAN | Chromobox protein homolog 5 | 18.3 | 18.08 | 17.97 |
| 493 | CBX8 | Non-matrisome | Non-matrisome | GO:CC not extracellular matrix | sp|Q9HC52|CBX8_HUMAN | Chromobox protein homolog 8 | 16.62 | 16.85 | 16.8 |
| 494 | CC2D1A | Non-matrisome | Non-matrisome | GO:CC not extracellular matrix | sp|Q6P1N0|C2D1A_HUMAN | Coiled-coil and C2 domain-containing protein 1A | 16.92 | 16.89 | 16.94 |
| 495 | CCAR1 | Non-matrisome | Non-matrisome | GO:CC not extracellular matrix | sp|Q8IX12|CCAR1_HUMAN | Cell division cycle and apoptosis regulator protein 1 | 17.82 | 18.22 | 17.97 |
| 496 | CCAR2 | Non-matrisome | Non-matrisome | GO:CC not extracellular matrix | sp|Q8N163|CCAR2_HUMAN | Cell cycle and apoptosis regulator protein 2 | 17.68 | 17.77 | 17.76 |
| 497 | CCDC124 | Non-matrisome | Non-matrisome | GO:CC not extracellular matrix | sp|Q96CT7|CC124_HUMAN | Coiled-coil domain-containing protein 124 | 19.3 | 18.6 | 18.78 |
| 498 | CCDC22 | Non-matrisome | Non-matrisome | GO:CC not extracellular matrix | sp|O60826|CCD22_HUMAN | Coiled-coil domain-containing protein 22 | 16.77 | 17.11 | 17.01 |
| 499 | CCDC47 | Non-matrisome | Non-matrisome | GO:CC not extracellular matrix | sp|Q96A33|CCD47_HUMAN | PAT complex subunit CCDC47 | 18.35 | 18.25 | 18.25 |
| 500 | CCDC50 | Non-matrisome | Non-matrisome | GO:CC not extracellular matrix | sp|Q8IVM0|CCD50_HUMAN | Coiled-coil domain-containing protein 50 | 19.55 | 18.53 | 18.86 |
| 501 | CCDC6 | Non-matrisome | Non-matrisome | GO:CC not extracellular matrix | sp|Q16204|CCDC6_HUMAN | Coiled-coil domain-containing protein 6 | 17.73 | 17.4 | 17.37 |
| 502 | CCDC80 | Non-matrisome | Non-matrisome | GO:CC extracellular matrix (GO:0005576, GO:0005578 & GO:0031012) & GO:CC not extracellular matrix | sp|Q76M96|CCD80_HUMAN | Coiled-coil domain-containing protein 80 | 17.02 | 16.99 | 16.18 |
| 503 | CCDC9 | Non-matrisome | Non-matrisome | GO:CC not extracellular matrix | sp|Q9Y3X0|CCDC9_HUMAN | Coiled-coil domain-containing protein 9 | 17.94 | 17.68 | 17.58 |
| 504 | CCDC93 | Non-matrisome | Non-matrisome | GO:CC not extracellular matrix | sp|Q567U6|CCD93_HUMAN | Coiled-coil domain-containing protein 93 | 17.61 | 17.94 | 17.92 |
| 505 | CCDC97 | Non-matrisome | Non-matrisome | gene not present in GO IDs | sp|Q96F63|CCD97_HUMAN | Coiled-coil domain-containing protein 97 | 16.51 | 16.12 | 16.25 |
| 506 | CCS | Non-matrisome | Non-matrisome | GO:CC not extracellular matrix | sp|O14618|CCS_HUMAN | Copper chaperone for superoxide dismutase | 19.04 | 19.48 | 19.24 |
| 507 | CCT2 | Non-matrisome | Non-matrisome | GO:CC extracellular matrix (GO:0005576, GO:0005578 & GO:0031012) & GO:CC not extracellular matrix | sp|P78371|TCPB_HUMAN | T-complex protein 1 subunit beta | 20.36 | 20.08 | 19.89 |
| 508 | CCT3 | Non-matrisome | Non-matrisome | GO:CC extracellular matrix (GO:0005576, GO:0005578 & GO:0031012) & GO:CC not extracellular matrix | sp|P49368|TCPG_HUMAN | T-complex protein 1 subunit gamma | 19.09 | 18.75 | 18.59 |
| 509 | CCT4 | Non-matrisome | Non-matrisome | GO:CC extracellular matrix (GO:0005576, GO:0005578 & GO:0031012) & GO:CC not extracellular matrix | sp|P50991|TCPD_HUMAN | T-complex protein 1 subunit delta | 19.75 | 19.46 | 19.25 |
| 510 | CCT5 | Non-matrisome | Non-matrisome | GO:CC extracellular matrix (GO:0005576, GO:0005578 & GO:0031012) & GO:CC not extracellular matrix | sp|P48643|TCPE_HUMAN | T-complex protein 1 subunit epsilon | 20.29 | 19.87 | 19.69 |
| 511 | CCT6A | Non-matrisome | Non-matrisome | GO:CC not extracellular matrix | sp|P40227|TCPZ_HUMAN | T-complex protein 1 subunit zeta | 20.49 | 20.22 | 19.98 |
| 512 | CCT7 | Non-matrisome | Non-matrisome | GO:CC extracellular matrix (GO:0005576, GO:0005578 & GO:0031012) & GO:CC not extracellular matrix | sp|Q99832|TCPH_HUMAN | T-complex protein 1 subunit eta | 19.24 | 18.92 | 18.67 |
| 513 | CCT8 | Non-matrisome | Non-matrisome | GO:CC extracellular matrix (GO:0005576, GO:0005578 & GO:0031012) & GO:CC not extracellular matrix | sp|P50990|TCPQ_HUMAN | T-complex protein 1 subunit theta | 20.42 | 20.14 | 19.89 |
| 514 | CD109 | Matrisome-associated | ECM Regulators | GO:CC extracellular matrix (GO:0005576, GO:0005578 & GO:0031012) & GO:CC not extracellular matrix | sp|Q6YHK3|CD109_HUMAN | CD109 antigen | 17.58 | 19.66 | 18.94 |
| 515 | CD14 | Non-matrisome | Non-matrisome | GO:CC extracellular matrix (GO:0005576, GO:0005578 & GO:0031012) & GO:CC not extracellular matrix | sp|P08571|CD14_HUMAN | Monocyte differentiation antigen CD14 | 15.13 | 14.69 | 14.81 |
| 516 | CD151 | Non-matrisome | Non-matrisome | GO:CC not extracellular matrix | sp|P48509|CD151_HUMAN | CD151 antigen | 20.12 | 19.33 | 19.19 |
| 517 | CD248 | Non-matrisome | Non-matrisome | GO:CC extracellular matrix (GO:0005576, GO:0005578 & GO:0031012) & GO:CC not extracellular matrix | sp|Q9HCU0|CD248_HUMAN | Endosialin | 16.73 | 18.33 | 18.58 |
| 518 | CD276 | Non-matrisome | Non-matrisome | GO:CC not extracellular matrix | sp|Q5ZPR3|CD276_HUMAN | CD276 antigen | 16.21 | 16.39 | 16.65 |
| 519 | CD2AP | Non-matrisome | Non-matrisome | GO:CC not extracellular matrix | sp|Q9Y5K6|CD2AP_HUMAN | CD2-associated protein | 18.49 | 18.15 | 18.11 |
| 520 | CD2BP2 | Non-matrisome | Non-matrisome | GO:CC not extracellular matrix | sp|O95400|CD2B2_HUMAN | CD2 antigen cytoplasmic tail-binding protein 2 | 18.04 | 17.52 | 17.51 |
| 521 | CD36 | Non-matrisome | Non-matrisome | GO:CC not extracellular matrix | sp|P16671|CD36_HUMAN | Platelet glycoprotein 4 | 15.52 | 16.37 | 16.82 |
| 522 | CD40 | Non-matrisome | Non-matrisome | GO:CC not extracellular matrix | sp|P25942|TNR5_HUMAN | Tumor necrosis factor receptor superfamily member 5 | 15.37 | 17.1 | 17.23 |
| 523 | CD44 | Non-matrisome | Non-matrisome | GO:CC not extracellular matrix | sp|P16070|CD44_HUMAN | CD44 antigen | 17.57 | 17.43 | 17.24 |
| 524 | CD46 | Non-matrisome | Non-matrisome | GO:CC not extracellular matrix | sp|P15529|MCP_HUMAN | Membrane cofactor protein | 20.09 | 20.09 | 20.02 |
| 525 | CD47 | Non-matrisome | Non-matrisome | GO:CC not extracellular matrix | sp|Q08722|CD47_HUMAN | Leukocyte surface antigen CD47 | 17.97 | 19.45 | 19.35 |
| 526 | CD55 | Non-matrisome | Non-matrisome | GO:CC extracellular matrix (GO:0005576, GO:0005578 & GO:0031012) & GO:CC not extracellular matrix | sp|P08174|DAF_HUMAN | Complement decay-accelerating factor | 18.07 | 18.5 | 17.77 |
| 527 | CD59 | Non-matrisome | Non-matrisome | GO:CC not extracellular matrix | sp|P13987|CD59_HUMAN | CD59 glycoprotein | 19.39 | 19.2 | 18.99 |
| 528 | CD63 | Non-matrisome | Non-matrisome | GO:CC not extracellular matrix | sp|P08962|CD63_HUMAN | CD63 antigen | 18.61 | 19.78 | 20.23 |
| 529 | CD74 | Non-matrisome | Non-matrisome | GO:CC not extracellular matrix | sp|P04233|HG2A_HUMAN | HLA class II histocompatibility antigen gamma chain | 11.67 | 12.01 | 11.97 |
| 530 | CD81 | Non-matrisome | Non-matrisome | GO:CC not extracellular matrix | sp|P60033|CD81_HUMAN | CD81 antigen | 17.97 | 18.55 | 18.77 |
| 531 | CD82 | Non-matrisome | Non-matrisome | GO:CC not extracellular matrix | sp|P27701|CD82_HUMAN | CD82 antigen | 16.74 | 17.43 | 17.7 |
| 532 | CD9 | Non-matrisome | Non-matrisome | GO:CC not extracellular matrix | sp|P21926|CD9_HUMAN | CD9 antigen | 18.2 | 18.89 | 18.82 |
| 533 | CD99 | Non-matrisome | Non-matrisome | GO:CC not extracellular matrix | sp|P14209|CD99_HUMAN | CD99 antigen | 16.44 | 15.75 | 15.77 |
| 534 | CDA | Non-matrisome | Non-matrisome | GO:CC extracellular matrix (GO:0005576, GO:0005578 & GO:0031012) & GO:CC not extracellular matrix | sp|P32320|CDD_HUMAN | Cytidine deaminase | 15.99 | 16.07 | 15.77 |
| 535 | CDC23 | Non-matrisome | Non-matrisome | GO:CC not extracellular matrix | sp|Q9UJX2|CDC23_HUMAN | Cell division cycle protein 23 homolog | 17.52 | 17.36 | 17.31 |
| 536 | CDC37 | Non-matrisome | Non-matrisome | GO:CC not extracellular matrix | sp|Q16543|CDC37_HUMAN | Hsp90 co-chaperone Cdc37 | 19.95 | 19.25 | 19.2 |
| 537 | CDC42 | Non-matrisome | Non-matrisome | GO:CC not extracellular matrix | sp|P60953|CDC42_HUMAN | Cell division control protein 42 homolog | 20.54 | 20.47 | 20.39 |
| 538 | CDC42BPB | Non-matrisome | Non-matrisome | GO:CC not extracellular matrix | sp|Q9Y5S2|MRCKB_HUMAN | Serine/threonine-protein kinase MRCK beta | 17.74 | 18.17 | 18.26 |
| 539 | CDC42EP4 | Non-matrisome | Non-matrisome | GO:CC not extracellular matrix | sp|Q9H3Q1|BORG4_HUMAN | Cdc42 effector protein 4 | 17.26 | 17.36 | 17.27 |
| 540 | CDC5L | Non-matrisome | Non-matrisome | GO:CC not extracellular matrix | sp|Q99459|CDC5L_HUMAN | Cell division cycle 5-like protein | 18 | 17.66 | 17.76 |
| 541 | CDC73 | Non-matrisome | Non-matrisome | GO:CC not extracellular matrix | sp|Q6P1J9|CDC73_HUMAN | Parafibromin | 18.25 | 17.43 | 17.6 |
| 542 | CDCP1 | Non-matrisome | Non-matrisome | GO:CC extracellular matrix (GO:0005576, GO:0005578 & GO:0031012) & GO:CC not extracellular matrix | sp|Q9H5V8|CDCP1_HUMAN | CUB domain-containing protein 1 | 17.63 | 18.33 | 18.18 |
| 543 | CDH1 | Non-matrisome | Non-matrisome | GO:CC extracellular matrix (GO:0005576, GO:0005578 & GO:0031012) & GO:CC not extracellular matrix | sp|P12830|CADH1_HUMAN | Cadherin-1 | 17.76 | 17.34 | 17.36 |
| 544 | CDH11 | Non-matrisome | Non-matrisome | GO:CC not extracellular matrix | sp|P55287|CAD11_HUMAN | Cadherin-11 | 15.59 | 18.42 | 18.48 |
| 545 | CDH13 | Non-matrisome | Non-matrisome | GO:CC extracellular matrix (GO:0005576, GO:0005578 & GO:0031012) & GO:CC not extracellular matrix | sp|P55290|CAD13_HUMAN | Cadherin-13 | 15.56 | 18.15 | 17.64 |
| 546 | CDIPT | Non-matrisome | Non-matrisome | GO:CC not extracellular matrix | sp|O14735|CDIPT_HUMAN | CDP-diacylglycerol--inositol 3-phosphatidyltransferase | 18.1 | 18.72 | 18.61 |
| 547 | CDK1 | Non-matrisome | Non-matrisome | GO:CC not extracellular matrix | sp|P06493|CDK1_HUMAN | Cyclin-dependent kinase 1 | 20.32 | 18.02 | 18.44 |
| 548 | CDK11B | Non-matrisome | Non-matrisome | GO:CC not extracellular matrix | sp|P21127|CD11B_HUMAN | Cyclin-dependent kinase 11B | 17.91 | 17.83 | 17.87 |
| 549 | CDK2 | Non-matrisome | Non-matrisome | GO:CC not extracellular matrix | sp|P24941|CDK2_HUMAN | Cyclin-dependent kinase 2 | 18.09 | 16.96 | 16.91 |
| 550 | CDK2AP1 | Non-matrisome | Non-matrisome | GO:CC extracellular matrix (GO:0005576, GO:0005578 & GO:0031012) & GO:CC not extracellular matrix | sp|O14519|CDKA1_HUMAN | Cyclin-dependent kinase 2-associated protein 1 | 16.17 | 15.27 | 15.83 |
| 551 | CDK5RAP3 | Non-matrisome | Non-matrisome | GO:CC not extracellular matrix | sp|Q96JB5|CK5P3_HUMAN | CDK5 regulatory subunit-associated protein 3 | 18.09 | 18 | 17.93 |
| 552 | CDK6 | Non-matrisome | Non-matrisome | GO:CC not extracellular matrix | sp|Q00534|CDK6_HUMAN | Cyclin-dependent kinase 6 | 18.78 | 16.94 | 16.5 |
| 553 | CDK9 | Non-matrisome | Non-matrisome | GO:CC not extracellular matrix | sp|P50750|CDK9_HUMAN | Cyclin-dependent kinase 9 | 16.75 | 16.8 | 16.92 |
| 554 | CDKN2AIP | Non-matrisome | Non-matrisome | GO:CC not extracellular matrix | sp|Q9NXV6|CARF_HUMAN | CDKN2A-interacting protein | 16.72 | 16.81 | 17 |
| 555 | CDS2 | Non-matrisome | Non-matrisome | GO:CC not extracellular matrix | sp|O95674|CDS2_HUMAN | Phosphatidate cytidylyltransferase 2 | 17.29 | 17.57 | 17.42 |
| 556 | CDV3 | Non-matrisome | Non-matrisome | GO:CC not extracellular matrix | sp|Q9UKY7|CDV3_HUMAN | Protein CDV3 homolog | 17.7 | 16.54 | 16.61 |
| 557 | CEBPB | Non-matrisome | Non-matrisome | GO:CC not extracellular matrix | sp|P17676|CEBPB_HUMAN | CCAAT/enhancer-binding protein beta | 17 | 18.59 | 18.12 |
| 558 | CEBPD | Non-matrisome | Non-matrisome | GO:CC not extracellular matrix | sp|P49716|CEBPD_HUMAN | CCAAT/enhancer-binding protein delta | 14.05 | 13.69 | 13.71 |
| 559 | CELF2 | Non-matrisome | Non-matrisome | GO:CC not extracellular matrix | sp|O95319|CELF2_HUMAN | CUGBP Elav-like family member 2 | 18.61 | 18.67 | 18.4 |
| 560 | CENPA | Non-matrisome | Non-matrisome | GO:CC not extracellular matrix | sp|P49450|CENPA_HUMAN | Histone H3-like centromeric protein A | 14.14 | 15.41 | 15.08 |
| 561 | CENPB | Non-matrisome | Non-matrisome | GO:CC not extracellular matrix | sp|P07199|CENPB_HUMAN | Major centromere autoantigen B | 17.68 | 17.71 | 17.56 |
| 562 | CEP104 | Non-matrisome | Non-matrisome | GO:CC not extracellular matrix | sp|O60308|CE104_HUMAN | Centrosomal protein of 104 kDa | 15.12 | 14.94 | 15 |
| 563 | CEP170B | Non-matrisome | Non-matrisome | GO:CC not extracellular matrix | sp|Q9Y4F5|C170B_HUMAN | Centrosomal protein of 170 kDa protein B | 16.45 | 16.09 | 16.16 |
| 564 | CERS2 | Non-matrisome | Non-matrisome | GO:CC not extracellular matrix | sp|Q96G23|CERS2_HUMAN | Ceramide synthase 2 | 20.46 | 19.98 | 20.02 |
| 565 | CERT1 | Non-matrisome | Non-matrisome | GO:CC not extracellular matrix | sp|Q9Y5P4|CERT_HUMAN | Ceramide transfer protein | 17.16 | 17.74 | 17.65 |
| 566 | CES2 | Non-matrisome | Non-matrisome | GO:CC not extracellular matrix | sp|O00748|EST2_HUMAN | Cocaine esterase | 16.35 | 16.99 | 16.73 |
| 567 | CETN2 | Non-matrisome | Non-matrisome | GO:CC not extracellular matrix | sp|P41208|CETN2_HUMAN | Centrin-2 | 18.37 | 18.13 | 18.3 |
| 568 | CFAP44 | Non-matrisome | Non-matrisome | GO:CC not extracellular matrix | sp|Q96MT7|CFA44_HUMAN | Cilia- and flagella-associated protein 44 | 18.71 | 16.34 | 16.57 |
| 569 | CFH | Non-matrisome | Non-matrisome | GO:CC extracellular matrix (GO:0005576, GO:0005578 & GO:0031012) & GO:CC not extracellular matrix | sp|P08603|CFAH_HUMAN | Complement factor H | 16.32 | 17.15 | 17.21 |
| 570 | CFI | Non-matrisome | Non-matrisome | GO:CC extracellular matrix (GO:0005576, GO:0005578 & GO:0031012) & GO:CC not extracellular matrix | sp|P05156|CFAI_HUMAN | Complement factor I | 13.51 | 13.48 | 13.24 |
| 571 | CFL1 | Non-matrisome | Non-matrisome | GO:CC not extracellular matrix | sp|P23528|COF1_HUMAN | Cofilin-1 | 19.93 | 19.78 | 19.77 |
| 572 | CFL2 | Non-matrisome | Non-matrisome | GO:CC not extracellular matrix | sp|Q9Y281|COF2_HUMAN | Cofilin-2 | 19.3 | 19.18 | 19.03 |
| 573 | CHCHD2 | Non-matrisome | Non-matrisome | GO:CC not extracellular matrix | sp|Q9Y6H1|CHCH2_HUMAN | Coiled-coil-helix-coiled-coil-helix domain-containing protein 2 | 17.58 | 17.37 | 17.22 |
| 574 | CHCHD3 | Non-matrisome | Non-matrisome | GO:CC not extracellular matrix | sp|Q9NX63|MIC19_HUMAN | MICOS complex subunit MIC19 | 17.18 | 17.85 | 17.65 |
| 575 | CHCHD5 | Non-matrisome | Non-matrisome | GO:CC not extracellular matrix | sp|Q9BSY4|CHCH5_HUMAN | Coiled-coil-helix-coiled-coil-helix domain-containing protein 5 | 16.64 | 15.69 | 16.18 |
| 576 | CHCHD6 | Non-matrisome | Non-matrisome | GO:CC not extracellular matrix | sp|Q9BRQ6|MIC25_HUMAN | MICOS complex subunit MIC25 | 16.86 | 17.5 | 17.68 |
| 577 | CHD4 | Non-matrisome | Non-matrisome | GO:CC not extracellular matrix | sp|Q14839|CHD4_HUMAN | Chromodomain-helicase-DNA-binding protein 4 | 17.9 | 17.45 | 17.6 |
| 578 | CHD8 | Non-matrisome | Non-matrisome | GO:CC not extracellular matrix | sp|Q9HCK8|CHD8_HUMAN | Chromodomain-helicase-DNA-binding protein 8 | 16.66 | 16.19 | 16.36 |
| 579 | CHERP | Non-matrisome | Non-matrisome | GO:CC not extracellular matrix | sp|Q8IWX8|CHERP_HUMAN | Calcium homeostasis endoplasmic reticulum protein | 18.39 | 18.11 | 18.15 |
| 580 | CHI3L1 | Non-matrisome | Non-matrisome | GO:CC extracellular matrix (GO:0005576, GO:0005578 & GO:0031012) & GO:CC not extracellular matrix | sp|P36222|CH3L1_HUMAN | Chitinase-3-like protein 1 | 10.92 | 15.24 | 15.28 |
| 581 | CHIC2 | Non-matrisome | Non-matrisome | GO:CC not extracellular matrix | sp|Q9UKJ5|CHIC2_HUMAN | Cysteine-rich hydrophobic domain-containing protein 2 | 18.87 | 18.28 | 18.32 |
| 582 | CHID1 | Non-matrisome | Non-matrisome | GO:CC extracellular matrix (GO:0005576, GO:0005578 & GO:0031012) & GO:CC not extracellular matrix | sp|Q9BWS9|CHID1_HUMAN | Chitinase domain-containing protein 1 | 19.02 | 19.56 | 19.41 |
| 583 | CHMP1A | Non-matrisome | Non-matrisome | GO:CC not extracellular matrix | sp|Q9HD42|CHM1A_HUMAN | Charged multivesicular body protein 1a | 19.83 | 19.47 | 19.52 |
| 584 | CHMP1B | Non-matrisome | Non-matrisome | GO:CC not extracellular matrix | sp|Q7LBR1|CHM1B_HUMAN | Charged multivesicular body protein 1b | 18.48 | 19.2 | 19.5 |
| 585 | CHMP2A | Non-matrisome | Non-matrisome | GO:CC not extracellular matrix | sp|O43633|CHM2A_HUMAN | Charged multivesicular body protein 2a | 18.84 | 18.35 | 18.52 |
| 586 | CHMP3 | Non-matrisome | Non-matrisome | GO:CC not extracellular matrix | sp|Q9Y3E7|CHMP3_HUMAN | Charged multivesicular body protein 3 | 19.23 | 19.17 | 19.16 |
| 587 | CHMP4A | Non-matrisome | Non-matrisome | GO:CC not extracellular matrix | sp|Q9BY43|CHM4A_HUMAN | Charged multivesicular body protein 4a | 18.74 | 18.02 | 17.99 |
| 588 | CHMP4B | Non-matrisome | Non-matrisome | GO:CC not extracellular matrix | sp|Q9H444|CHM4B_HUMAN | Charged multivesicular body protein 4b | 19.56 | 19.46 | 19.59 |
| 589 | CHMP5 | Non-matrisome | Non-matrisome | GO:CC not extracellular matrix | sp|Q9NZZ3|CHMP5_HUMAN | Charged multivesicular body protein 5 | 18.73 | 18.57 | 18.8 |
| 590 | CHMP6 | Non-matrisome | Non-matrisome | GO:CC not extracellular matrix | sp|Q96FZ7|CHMP6_HUMAN | Charged multivesicular body protein 6 | 18.71 | 18.65 | 18.53 |
| 591 | CHP1 | Non-matrisome | Non-matrisome | GO:CC not extracellular matrix | sp|Q99653|CHP1_HUMAN | Calcineurin B homologous protein 1 | 17.85 | 17.82 | 18.06 |
| 592 | CHTOP | Non-matrisome | Non-matrisome | GO:CC not extracellular matrix | sp|Q9Y3Y2|CHTOP_HUMAN | Chromatin target of PRMT1 protein | 16.72 | 16.68 | 16.85 |
| 593 | CIAO1 | Non-matrisome | Non-matrisome | GO:CC not extracellular matrix | sp|O76071|CIAO1_HUMAN | Probable cytosolic iron-sulfur protein assembly protein CIAO1 | 17.45 | 17.39 | 17.27 |
| 594 | CIAO2A | Non-matrisome | Non-matrisome | GO:CC not extracellular matrix | sp|Q9H5X1|CIA2A_HUMAN | Cytosolic iron-sulfur assembly component 2A | 16.52 | 16.32 | 16.29 |
| 595 | CIAO2B | Non-matrisome | Non-matrisome | GO:CC not extracellular matrix | sp|Q9Y3D0|CIA2B_HUMAN | Cytosolic iron-sulfur assembly component 2B | 16.85 | 16.42 | 16.48 |
| 596 | CIAPIN1 | Non-matrisome | Non-matrisome | GO:CC extracellular matrix (GO:0005576, GO:0005578 & GO:0031012) & GO:CC not extracellular matrix | sp|Q6FI81|CPIN1_HUMAN | Anamorsin | 17.61 | 16.82 | 16.91 |
| 597 | CILP | Core matrisome | ECM Glycoproteins | GO:CC not extracellular matrix | sp|O75339|CILP1_HUMAN | Cartilage intermediate layer protein 1 | 15.15 | 14.04 | 15.26 |
| 598 | CIRBP | Non-matrisome | Non-matrisome | GO:CC not extracellular matrix | sp|Q14011|CIRBP_HUMAN | Cold-inducible RNA-binding protein | 18.11 | 17.83 | 18.19 |
| 599 | CISD1 | Non-matrisome | Non-matrisome | GO:CC not extracellular matrix | sp|Q9NZ45|CISD1_HUMAN | CDGSH iron-sulfur domain-containing protein 1 | 20.46 | 20.99 | 20.31 |
| 600 | CISD2 | Non-matrisome | Non-matrisome | GO:CC not extracellular matrix | sp|Q8N5K1|CISD2_HUMAN | CDGSH iron-sulfur domain-containing protein 2 | 17.53 | 17.81 | 17.75 |
| 601 | CIT | Non-matrisome | Non-matrisome | GO:CC not extracellular matrix | sp|O14578|CTRO_HUMAN | Citron Rho-interacting kinase | 17.16 | 15.98 | 16.14 |
| 602 | CKAP4 | Non-matrisome | Non-matrisome | GO:CC not extracellular matrix | sp|Q07065|CKAP4_HUMAN | Cytoskeleton-associated protein 4 | 18.86 | 19.43 | 19.07 |
| 603 | CKAP5 | Non-matrisome | Non-matrisome | GO:CC not extracellular matrix | sp|Q14008|CKAP5_HUMAN | Cytoskeleton-associated protein 5 | 18.58 | 17.88 | 18 |
| 604 | CKB | Non-matrisome | Non-matrisome | GO:CC not extracellular matrix | sp|P12277|KCRB_HUMAN | Creatine kinase B-type | 15.85 | 16.74 | 16.02 |
| 605 | CLASP2 | Non-matrisome | Non-matrisome | GO:CC not extracellular matrix | sp|O75122|CLAP2_HUMAN | CLIP-associating protein 2 | 17.18 | 16.74 | 16.88 |
| 606 | CLASRP | Non-matrisome | Non-matrisome | GO:CC not extracellular matrix | sp|Q8N2M8|CLASR_HUMAN | CLK4-associating serine/arginine rich protein | 17.65 | 17.57 | 17.53 |
| 607 | CLCA2 | Non-matrisome | Non-matrisome | GO:CC extracellular matrix (GO:0005576, GO:0005578 & GO:0031012) & GO:CC not extracellular matrix | sp|Q9UQC9|CLCA2_HUMAN | Calcium-activated chloride channel regulator 2 | 13.5 | 17.19 | 18.28 |
| 608 | CLDND1 | Non-matrisome | Non-matrisome | GO:CC not extracellular matrix | sp|Q9NY35|CLDN1_HUMAN | Claudin domain-containing protein 1 | 18.2 | 18.1 | 18.11 |
| 609 | CLEC16A | Non-matrisome | Non-matrisome | GO:CC not extracellular matrix | sp|Q2KHT3|CL16A_HUMAN | Protein CLEC16A | 16.37 | 16.37 | 16.44 |
| 610 | CLEC2B | Matrisome-associated | ECM-affiliated Proteins | GO:CC not extracellular matrix | sp|Q92478|CLC2B_HUMAN | C-type lectin domain family 2 member B | 14.56 | 16.44 | 15.98 |
| 611 | CLEC3B | Matrisome-associated | ECM-affiliated Proteins | GO:CC extracellular matrix (GO:0005576, GO:0005578 & GO:0031012) & GO:CC not extracellular matrix | sp|P05452|TETN_HUMAN | Tetranectin | 16.32 | 16.43 | 15.94 |
| 612 | CLIC1 | Non-matrisome | Non-matrisome | GO:CC not extracellular matrix | sp|O00299|CLIC1_HUMAN | Chloride intracellular channel protein 1 | 20.94 | 20.93 | 20.77 |
| 613 | CLIC2 | Non-matrisome | Non-matrisome | GO:CC not extracellular matrix | sp|O15247|CLIC2_HUMAN | Chloride intracellular channel protein 2 | 13.62 | 16.51 | 16.59 |
| 614 | CLIC4 | Non-matrisome | Non-matrisome | GO:CC not extracellular matrix | sp|Q9Y696|CLIC4_HUMAN | Chloride intracellular channel protein 4 | 18.32 | 18.06 | 18.05 |
| 615 | CLINT1 | Non-matrisome | Non-matrisome | GO:CC not extracellular matrix | sp|Q14677|EPN4_HUMAN | Clathrin interactor 1 | 18.47 | 17.94 | 18.07 |
| 616 | CLIP1 | Non-matrisome | Non-matrisome | GO:CC not extracellular matrix | sp|P30622|CLIP1_HUMAN | CAP-Gly domain-containing linker protein 1 | 18.3 | 17.48 | 17.57 |
| 617 | CLIP2 | Non-matrisome | Non-matrisome | GO:CC not extracellular matrix | sp|Q9UDT6|CLIP2_HUMAN | CAP-Gly domain-containing linker protein 2 | 18.29 | 18.57 | 18.6 |
| 618 | CLNS1A | Non-matrisome | Non-matrisome | GO:CC not extracellular matrix | sp|P54105|ICLN_HUMAN | Methylosome subunit pICln | 16.52 | 15.73 | 15.74 |
| 619 | CLPP | Non-matrisome | Non-matrisome | GO:CC not extracellular matrix | sp|Q16740|CLPP_HUMAN | ATP-dependent Clp protease proteolytic subunit, mitochondrial | 17.48 | 17.61 | 17.24 |
| 620 | CLPTM1 | Non-matrisome | Non-matrisome | GO:CC not extracellular matrix | sp|O96005|CLPT1_HUMAN | Putative lipid scramblase CLPTM1 | 16.84 | 16.01 | 16.38 |
| 621 | CLSTN1 | Non-matrisome | Non-matrisome | GO:CC extracellular matrix (GO:0005576, GO:0005578 & GO:0031012) & GO:CC not extracellular matrix | sp|O94985|CSTN1_HUMAN | Calsyntenin-1 | 17.92 | 17.61 | 17.45 |
| 622 | CLTA | Non-matrisome | Non-matrisome | GO:CC not extracellular matrix | sp|P09496|CLCA_HUMAN | Clathrin light chain A | 22.17 | 22.3 | 22.17 |
| 623 | CLTB | Non-matrisome | Non-matrisome | GO:CC not extracellular matrix | sp|P09497|CLCB_HUMAN | Clathrin light chain B | 20.42 | 20.56 | 20.18 |
| 624 | CLTC | Non-matrisome | Non-matrisome | GO:CC not extracellular matrix | sp|Q00610|CLH1_HUMAN | Clathrin heavy chain 1 | 19.62 | 19.94 | 19.7 |
| 625 | CLU | Non-matrisome | Non-matrisome | GO:CC extracellular matrix (GO:0005576, GO:0005578 & GO:0031012) & GO:CC not extracellular matrix | sp|P10909|CLUS_HUMAN | Clusterin | 14.55 | 17.11 | 18.48 |
| 626 | CLUH | Non-matrisome | Non-matrisome | GO:CC not extracellular matrix | sp|O75153|CLU_HUMAN | Clustered mitochondria protein homolog | 17.97 | 16.17 | 16.4 |
| 627 | CMAS | Non-matrisome | Non-matrisome | GO:CC not extracellular matrix | sp|Q8NFW8|NEUA_HUMAN | N-acylneuraminate cytidylyltransferase | 17.39 | 18.03 | 17.97 |
| 628 | CMPK1 | Non-matrisome | Non-matrisome | GO:CC not extracellular matrix | sp|P30085|KCY_HUMAN | UMP-CMP kinase | 18.62 | 18.67 | 18.76 |
| 629 | CMPK2 | Non-matrisome | Non-matrisome | GO:CC not extracellular matrix | sp|Q5EBM0|CMPK2_HUMAN | UMP-CMP kinase 2, mitochondrial | 12.12 | 12.94 | 12.86 |
| 630 | CMTM4 | Non-matrisome | Non-matrisome | GO:CC not extracellular matrix | sp|Q8IZR5|CKLF4_HUMAN | CKLF-like MARVEL transmembrane domain-containing protein 4 | 15.13 | 15.64 | 15.74 |
| 631 | CMTR1 | Non-matrisome | Non-matrisome | GO:CC not extracellular matrix | sp|Q8N1G2|CMTR1_HUMAN | Cap-specific mRNA (nucleoside-2'-O-)-methyltransferase 1 | 17.73 | 17.71 | 17.66 |
| 632 | CNBP | Non-matrisome | Non-matrisome | GO:CC extracellular matrix (GO:0005576, GO:0005578 & GO:0031012) & GO:CC not extracellular matrix | sp|P62633|CNBP_HUMAN | CCHC-type zinc finger nucleic acid binding protein | 17.83 | 17.09 | 17.51 |
| 633 | CNDP2 | Non-matrisome | Non-matrisome | GO:CC not extracellular matrix | sp|Q96KP4|CNDP2_HUMAN | Cytosolic non-specific dipeptidase | 19.04 | 19.35 | 19.23 |
| 634 | CNN1 | Non-matrisome | Non-matrisome | GO:CC not extracellular matrix | sp|P51911|CNN1_HUMAN | Calponin-1 | 16.99 | 17.86 | 17.55 |
| 635 | CNN2 | Non-matrisome | Non-matrisome | GO:CC extracellular matrix (GO:0005576, GO:0005578 & GO:0031012) & GO:CC not extracellular matrix | sp|Q99439|CNN2_HUMAN | Calponin-2 | 19.04 | 18.07 | 18.19 |
| 636 | CNN3 | Non-matrisome | Non-matrisome | GO:CC not extracellular matrix | sp|Q15417|CNN3_HUMAN | Calponin-3 | 18.86 | 18.23 | 18.43 |
| 637 | CNNM2 | Non-matrisome | Non-matrisome | GO:CC not extracellular matrix | sp|Q9H8M5|CNNM2_HUMAN | Metal transporter CNNM2 | 15.18 | 16.2 | 16.04 |
| 638 | CNOT1 | Non-matrisome | Non-matrisome | GO:CC not extracellular matrix | sp|A5YKK6|CNOT1_HUMAN | CCR4-NOT transcription complex subunit 1 | 17.8 | 17.76 | 17.75 |
| 639 | CNOT11 | Non-matrisome | Non-matrisome | GO:CC not extracellular matrix | sp|Q9UKZ1|CNO11_HUMAN | CCR4-NOT transcription complex subunit 11 | 17.11 | 17.01 | 17 |
| 640 | CNOT2 | Non-matrisome | Non-matrisome | GO:CC not extracellular matrix | sp|Q9NZN8|CNOT2_HUMAN | CCR4-NOT transcription complex subunit 2 | 17.6 | 17.38 | 17.41 |
| 641 | CNOT6L | Non-matrisome | Non-matrisome | GO:CC not extracellular matrix | sp|Q96LI5|CNO6L_HUMAN | CCR4-NOT transcription complex subunit 6-like | 17.34 | 17.22 | 17.21 |
| 642 | CNOT7 | Non-matrisome | Non-matrisome | GO:CC not extracellular matrix | sp|Q9UIV1|CNOT7_HUMAN | CCR4-NOT transcription complex subunit 7 | 15.17 | 15.14 | 14.93 |
| 643 | CNOT9 | Non-matrisome | Non-matrisome | GO:CC not extracellular matrix | sp|Q92600|CNOT9_HUMAN | CCR4-NOT transcription complex subunit 9 | 17.21 | 17.14 | 17.23 |
| 644 | CNP | Non-matrisome | Non-matrisome | GO:CC not extracellular matrix | sp|P09543|CN37_HUMAN | 2',3'-cyclic-nucleotide 3'-phosphodiesterase | 18.87 | 19.49 | 19.49 |
| 645 | CNPY2 | Non-matrisome | Non-matrisome | GO:CC not extracellular matrix | sp|Q9Y2B0|CNPY2_HUMAN | Protein canopy homolog 2 | 17.96 | 17.99 | 18.04 |
| 646 | CNPY3 | Non-matrisome | Non-matrisome | GO:CC not extracellular matrix | sp|Q9BT09|CNPY3_HUMAN | Protein canopy homolog 3 | 19.03 | 19.08 | 19.06 |
| 647 | CNPY4 | Non-matrisome | Non-matrisome | GO:CC extracellular matrix (GO:0005576, GO:0005578 & GO:0031012) & GO:CC not extracellular matrix | sp|Q8N129|CNPY4_HUMAN | Protein canopy homolog 4 | 18.29 | 18.4 | 18.31 |
| 648 | CNTNAP1 | Non-matrisome | Non-matrisome | GO:CC not extracellular matrix | sp|P78357|CNTP1_HUMAN | Contactin-associated protein 1 | 16.6 | 17.4 | 16.89 |
| 649 | COA6 | Non-matrisome | Non-matrisome | GO:CC not extracellular matrix | sp|Q5JTJ3|COA6_HUMAN | Cytochrome c oxidase assembly factor 6 homolog | 19.05 | 19.26 | 19.34 |
| 650 | COASY | Non-matrisome | Non-matrisome | GO:CC not extracellular matrix | sp|Q13057|COASY_HUMAN | Bifunctional coenzyme A synthase | 17.58 | 17.67 | 17.53 |
| 651 | COBLL1 | Non-matrisome | Non-matrisome | GO:CC not extracellular matrix | sp|Q53SF7|COBL1_HUMAN | Cordon-bleu protein-like 1 | 17.17 | 16.62 | 16.39 |
| 652 | COCH | Core matrisome | ECM Glycoproteins | GO:CC extracellular matrix (GO:0005576, GO:0005578 & GO:0031012) & GO:CC not extracellular matrix | sp|O43405|COCH_HUMAN | Cochlin | 16.04 | 16.93 | 18.15 |
| 653 | COG2 | Non-matrisome | Non-matrisome | GO:CC not extracellular matrix | sp|Q14746|COG2_HUMAN | Conserved oligomeric Golgi complex subunit 2 | 17.75 | 17.61 | 17.78 |
| 654 | COG3 | Non-matrisome | Non-matrisome | GO:CC not extracellular matrix | sp|Q96JB2|COG3_HUMAN | Conserved oligomeric Golgi complex subunit 3 | 17.45 | 17.3 | 17.42 |
| 655 | COG5 | Non-matrisome | Non-matrisome | GO:CC not extracellular matrix | sp|Q9UP83|COG5_HUMAN | Conserved oligomeric Golgi complex subunit 5 | 17.35 | 17.18 | 17.31 |
| 656 | COG7 | Non-matrisome | Non-matrisome | GO:CC not extracellular matrix | sp|P83436|COG7_HUMAN | Conserved oligomeric Golgi complex subunit 7 | 18.03 | 18 | 18.08 |
| 657 | COIL | Non-matrisome | Non-matrisome | GO:CC not extracellular matrix | sp|P38432|COIL_HUMAN | Coilin | 16.35 | 16.61 | 16.66 |
| 658 | COL12A1 | Core matrisome | Collagens | GO:CC extracellular matrix (GO:0005576, GO:0005578 & GO:0031012) & GO:CC not extracellular matrix | sp|Q99715|COCA1_HUMAN | Collagen alpha-1(XII) chain | 17.02 | 19.06 | 18.26 |
| 659 | COL14A1 | Core matrisome | Collagens | GO:CC extracellular matrix (GO:0005576, GO:0005578 & GO:0031012) & GO:CC not extracellular matrix | sp|Q05707|COEA1_HUMAN | Collagen alpha-1(XIV) chain | 14.8 | 16 | 16.44 |
| 660 | COL15A1 | Core matrisome | Collagens | GO:CC extracellular matrix (GO:0005576, GO:0005578 & GO:0031012) & GO:CC not extracellular matrix | sp|P39059|COFA1_HUMAN | Collagen alpha-1(XV) chain | 14.23 | 16.57 | 17.09 |
| 661 | COL16A1 | Core matrisome | Collagens | GO:CC extracellular matrix (GO:0005576, GO:0005578 & GO:0031012) & GO:CC not extracellular matrix | sp|Q07092|COGA1_HUMAN | Collagen alpha-1(XVI) chain | 16.3 | 17.27 | 18.1 |
| 662 | COL18A1 | Core matrisome | Collagens | GO:CC extracellular matrix (GO:0005576, GO:0005578 & GO:0031012) & GO:CC not extracellular matrix | sp|P39060|COIA1_HUMAN | Collagen alpha-1(XVIII) chain | 15.24 | 16.6 | 17.17 |
| 663 | COL1A1 | Core matrisome | Collagens | GO:CC extracellular matrix (GO:0005576, GO:0005578 & GO:0031012) & GO:CC not extracellular matrix | sp|P02452|CO1A1_HUMAN | Collagen alpha-1(I) chain | 18.8 | 16.04 | 17.17 |
| 664 | COL1A2 | Core matrisome | Collagens | GO:CC extracellular matrix (GO:0005576, GO:0005578 & GO:0031012) & GO:CC not extracellular matrix | sp|P08123|CO1A2_HUMAN | Collagen alpha-2(I) chain | 18.19 | 15.74 | 16.92 |
| 665 | COL21A1 | Core matrisome | Collagens | GO:CC extracellular matrix (GO:0005576, GO:0005578 & GO:0031012) & GO:CC not extracellular matrix | sp|Q96P44|COLA1_HUMAN | Collagen alpha-1(XXI) chain | 13.58 | 13.81 | 13.69 |
| 666 | COL2A1 | Core matrisome | Collagens | GO:CC extracellular matrix (GO:0005576, GO:0005578 & GO:0031012) & GO:CC not extracellular matrix | sp|P02458|CO2A1_HUMAN | Collagen alpha-1(II) chain | 17.34 | 16.22 | 17.51 |
| 667 | COL3A1 | Core matrisome | Collagens | GO:CC extracellular matrix (GO:0005576, GO:0005578 & GO:0031012) & GO:CC not extracellular matrix | sp|P02461|CO3A1_HUMAN | Collagen alpha-1(III) chain | 16.67 | 16.13 | 16.98 |
| 668 | COL4A1 | Core matrisome | Collagens | GO:CC extracellular matrix (GO:0005576, GO:0005578 & GO:0031012) & GO:CC not extracellular matrix | sp|P02462|CO4A1_HUMAN | Collagen alpha-1(IV) chain | 15.9 | 14.7 | 14.41 |
| 669 | COL4A2 | Core matrisome | Collagens | GO:CC extracellular matrix (GO:0005576, GO:0005578 & GO:0031012) & GO:CC not extracellular matrix | sp|P08572|CO4A2_HUMAN | Collagen alpha-2(IV) chain | 16.95 | 16.81 | 16.38 |
| 670 | COL5A1 | Core matrisome | Collagens | GO:CC extracellular matrix (GO:0005576, GO:0005578 & GO:0031012) & GO:CC not extracellular matrix | sp|P20908|CO5A1_HUMAN | Collagen alpha-1(V) chain | 18.23 | 17.42 | 17.37 |
| 671 | COL5A2 | Core matrisome | Collagens | GO:CC extracellular matrix (GO:0005576, GO:0005578 & GO:0031012) & GO:CC not extracellular matrix | sp|P05997|CO5A2_HUMAN | Collagen alpha-2(V) chain | 17.04 | 16.48 | 16.42 |
| 672 | COL5A3 | Core matrisome | Collagens | GO:CC extracellular matrix (GO:0005576, GO:0005578 & GO:0031012) & GO:CC not extracellular matrix | sp|P25940|CO5A3_HUMAN | Collagen alpha-3(V) chain | 15.57 | 16.88 | 16.51 |
| 673 | COL6A1 | Core matrisome | Collagens | GO:CC extracellular matrix (GO:0005576, GO:0005578 & GO:0031012) & GO:CC not extracellular matrix | sp|P12109|CO6A1_HUMAN | Collagen alpha-1(VI) chain | 17 | 18.88 | 18.8 |
| 674 | COL6A2 | Core matrisome | Collagens | GO:CC extracellular matrix (GO:0005576, GO:0005578 & GO:0031012) & GO:CC not extracellular matrix | sp|P12110|CO6A2_HUMAN | Collagen alpha-2(VI) chain | 17.69 | 19.48 | 19.35 |
| 675 | COL6A3 | Core matrisome | Collagens | GO:CC extracellular matrix (GO:0005576, GO:0005578 & GO:0031012) & GO:CC not extracellular matrix | sp|P12111|CO6A3_HUMAN | Collagen alpha-3(VI) chain | 17.97 | 19.98 | 19.57 |
| 676 | COL7A1 | Core matrisome | Collagens | GO:CC extracellular matrix (GO:0005576, GO:0005578 & GO:0031012) & GO:CC not extracellular matrix | sp|Q02388|CO7A1_HUMAN | Collagen alpha-1(VII) chain | 16.87 | 16.76 | 16.77 |
| 677 | COL8A1 | Core matrisome | Collagens | GO:CC extracellular matrix (GO:0005576, GO:0005578 & GO:0031012) & GO:CC not extracellular matrix | sp|P27658|CO8A1_HUMAN | Collagen alpha-1(VIII) chain | 17.54 | 17.33 | 17.04 |
| 678 | COLEC12 | Matrisome-associated | ECM-affiliated Proteins | GO:CC not extracellular matrix | sp|Q5KU26|COL12_HUMAN | Collectin-12 | 17.7 | 17.5 | 18.11 |
| 679 | COLGALT1 | Non-matrisome | Non-matrisome | GO:CC not extracellular matrix | sp|Q8NBJ5|GT251_HUMAN | Procollagen galactosyltransferase 1 | 19.38 | 19.4 | 18.97 |
| 680 | COMMD10 | Non-matrisome | Non-matrisome | GO:CC not extracellular matrix | sp|Q9Y6G5|COMDA_HUMAN | COMM domain-containing protein 10 | 17.39 | 17.67 | 17.75 |
| 681 | COMMD2 | Non-matrisome | Non-matrisome | GO:CC not extracellular matrix | sp|Q86X83|COMD2_HUMAN | COMM domain-containing protein 2 | 19.24 | 19.44 | 19.44 |
| 682 | COMMD3 | Non-matrisome | Non-matrisome | GO:CC extracellular matrix (GO:0005576, GO:0005578 & GO:0031012) & GO:CC not extracellular matrix | sp|Q9UBI1|COMD3_HUMAN | COMM domain-containing protein 3 | 17.7 | 17.83 | 17.99 |
| 683 | COMMD4 | Non-matrisome | Non-matrisome | GO:CC not extracellular matrix | sp|Q9H0A8|COMD4_HUMAN | COMM domain-containing protein 4 | 16.65 | 16.7 | 16.86 |
| 684 | COMMD5 | Non-matrisome | Non-matrisome | GO:CC not extracellular matrix | sp|Q9GZQ3|COMD5_HUMAN | COMM domain-containing protein 5 | 18.39 | 18.87 | 18.82 |
| 685 | COMMD6 | Non-matrisome | Non-matrisome | GO:CC not extracellular matrix | sp|Q7Z4G1|COMD6_HUMAN | COMM domain-containing protein 6 | 17.86 | 17.96 | 17.94 |
| 686 | COMMD9 | Non-matrisome | Non-matrisome | GO:CC extracellular matrix (GO:0005576, GO:0005578 & GO:0031012) & GO:CC not extracellular matrix | sp|Q9P000|COMD9_HUMAN | COMM domain-containing protein 9 | 18.36 | 18.6 | 18.64 |
| 687 | COMP | Core matrisome | ECM Glycoproteins | GO:CC extracellular matrix (GO:0005576, GO:0005578 & GO:0031012) & GO:CC not extracellular matrix | sp|P49747|COMP_HUMAN | Cartilage oligomeric matrix protein | 15.73 | 15.29 | 15.32 |
| 688 | COMT | Non-matrisome | Non-matrisome | GO:CC not extracellular matrix | sp|P21964|COMT_HUMAN | Catechol O-methyltransferase | 18.96 | 19.23 | 19.01 |
| 689 | COPA | Non-matrisome | Non-matrisome | GO:CC not extracellular matrix | sp|P53621|COPA_HUMAN | Coatomer subunit alpha | 19.4 | 19.03 | 19.08 |
| 690 | COPB1 | Non-matrisome | Non-matrisome | GO:CC not extracellular matrix | sp|P53618|COPB_HUMAN | Coatomer subunit beta | 18.94 | 18.54 | 18.64 |
| 691 | COPB2 | Non-matrisome | Non-matrisome | GO:CC not extracellular matrix | sp|P35606|COPB2_HUMAN | Coatomer subunit beta' | 18.86 | 18.45 | 18.54 |
| 692 | COPE | Non-matrisome | Non-matrisome | GO:CC not extracellular matrix | sp|O14579|COPE_HUMAN | Coatomer subunit epsilon | 18.71 | 18.2 | 18.21 |
| 693 | COPG1 | Non-matrisome | Non-matrisome | GO:CC not extracellular matrix | sp|Q9Y678|COPG1_HUMAN | Coatomer subunit gamma-1 | 18.81 | 18.23 | 18.38 |
| 694 | COPG2 | Non-matrisome | Non-matrisome | GO:CC not extracellular matrix | sp|Q9UBF2|COPG2_HUMAN | Coatomer subunit gamma-2 | 17.42 | 17.62 | 17.54 |
| 695 | COPS2 | Non-matrisome | Non-matrisome | GO:CC not extracellular matrix | sp|P61201|CSN2_HUMAN | COP9 signalosome complex subunit 2 | 18.13 | 18.32 | 18.24 |
| 696 | COPS3 | Non-matrisome | Non-matrisome | GO:CC not extracellular matrix | sp|Q9UNS2|CSN3_HUMAN | COP9 signalosome complex subunit 3 | 18.22 | 18.23 | 18.1 |
| 697 | COPS4 | Non-matrisome | Non-matrisome | GO:CC not extracellular matrix | sp|Q9BT78|CSN4_HUMAN | COP9 signalosome complex subunit 4 | 18.87 | 19.01 | 18.93 |
| 698 | COPS5 | Non-matrisome | Non-matrisome | GO:CC not extracellular matrix | sp|Q92905|CSN5_HUMAN | COP9 signalosome complex subunit 5 | 19.09 | 19.1 | 19.07 |
| 699 | COPS6 | Non-matrisome | Non-matrisome | GO:CC not extracellular matrix | sp|Q7L5N1|CSN6_HUMAN | COP9 signalosome complex subunit 6 | 18.34 | 18.42 | 18.3 |
| 700 | COPS7A | Non-matrisome | Non-matrisome | GO:CC not extracellular matrix | sp|Q9UBW8|CSN7A_HUMAN | COP9 signalosome complex subunit 7a | 18.2 | 18.19 | 18.16 |
| 701 | COPS7B | Non-matrisome | Non-matrisome | GO:CC not extracellular matrix | sp|Q9H9Q2|CSN7B_HUMAN | COP9 signalosome complex subunit 7b | 17.31 | 17.46 | 17.37 |
| 702 | COPS8 | Non-matrisome | Non-matrisome | GO:CC not extracellular matrix | sp|Q99627|CSN8_HUMAN | COP9 signalosome complex subunit 8 | 17.9 | 18.01 | 17.91 |
| 703 | COPZ1 | Non-matrisome | Non-matrisome | GO:CC not extracellular matrix | sp|P61923|COPZ1_HUMAN | Coatomer subunit zeta-1 | 18.88 | 18.19 | 18.41 |
| 704 | COQ3 | Non-matrisome | Non-matrisome | GO:CC not extracellular matrix | sp|Q9NZJ6|COQ3_HUMAN | Ubiquinone biosynthesis O-methyltransferase, mitochondrial | 18.1 | 18.15 | 18.04 |
| 705 | COQ6 | Non-matrisome | Non-matrisome | GO:CC not extracellular matrix | sp|Q9Y2Z9|COQ6_HUMAN | Ubiquinone biosynthesis monooxygenase COQ6, mitochondrial | 16.17 | 16.41 | 16.21 |
| 706 | COQ9 | Non-matrisome | Non-matrisome | GO:CC not extracellular matrix | sp|O75208|COQ9_HUMAN | Ubiquinone biosynthesis protein COQ9, mitochondrial | 17.9 | 18.39 | 18.29 |
| 707 | CORO1A | Non-matrisome | Non-matrisome | GO:CC not extracellular matrix | sp|P31146|COR1A_HUMAN | Coronin-1A | 17.25 | 17.57 | 17.51 |
| 708 | CORO1B | Non-matrisome | Non-matrisome | GO:CC not extracellular matrix | sp|Q9BR76|COR1B_HUMAN | Coronin-1B | 18.93 | 18.94 | 18.96 |
| 709 | CORO1C | Non-matrisome | Non-matrisome | GO:CC not extracellular matrix | sp|Q9ULV4|COR1C_HUMAN | Coronin-1C | 19.19 | 19.05 | 18.86 |
| 710 | CORO7 | Non-matrisome | Non-matrisome | GO:CC not extracellular matrix | sp|P57737|CORO7_HUMAN | Coronin-7 | 17.55 | 17.81 | 18.05 |
| 711 | COTL1 | Non-matrisome | Non-matrisome | GO:CC extracellular matrix (GO:0005576, GO:0005578 & GO:0031012) & GO:CC not extracellular matrix | sp|Q14019|COTL1_HUMAN | Coactosin-like protein | 21.27 | 21.18 | 21.24 |
| 712 | COX17 | Non-matrisome | Non-matrisome | GO:CC not extracellular matrix | sp|Q14061|COX17_HUMAN | Cytochrome c oxidase copper chaperone | 20.12 | 19.49 | 20.01 |
| 713 | COX4I1 | Non-matrisome | Non-matrisome | GO:CC not extracellular matrix | sp|P13073|COX41_HUMAN | Cytochrome c oxidase subunit 4 isoform 1, mitochondrial | 19.15 | 19.49 | 19.29 |
| 714 | COX5A | Non-matrisome | Non-matrisome | GO:CC extracellular matrix (GO:0005576, GO:0005578 & GO:0031012) & GO:CC not extracellular matrix | sp|P20674|COX5A_HUMAN | Cytochrome c oxidase subunit 5A, mitochondrial | 19.81 | 20.02 | 19.89 |
| 715 | COX5B | Non-matrisome | Non-matrisome | GO:CC extracellular matrix (GO:0005576, GO:0005578 & GO:0031012) & GO:CC not extracellular matrix | sp|P10606|COX5B_HUMAN | Cytochrome c oxidase subunit 5B, mitochondrial | 18.06 | 18.39 | 18.28 |
| 716 | COX6B1 | Non-matrisome | Non-matrisome | GO:CC not extracellular matrix | sp|P14854|CX6B1_HUMAN | Cytochrome c oxidase subunit 6B1 | 18.72 | 18.9 | 18.83 |
| 717 | COX6C | Non-matrisome | Non-matrisome | GO:CC not extracellular matrix | sp|P09669|COX6C_HUMAN | Cytochrome c oxidase subunit 6C | 20.83 | 21.32 | 21.12 |
| 718 | COX7A2 | Non-matrisome | Non-matrisome | GO:CC not extracellular matrix | sp|P14406|CX7A2_HUMAN | Cytochrome c oxidase subunit 7A2, mitochondrial | 20.46 | 20.74 | 20.6 |
| 719 | COX7A2L | Non-matrisome | Non-matrisome | GO:CC not extracellular matrix | sp|O14548|COX7R_HUMAN | Cytochrome c oxidase subunit 7A-related protein, mitochondrial | 19.25 | 18.93 | 18.76 |
| 720 | CPB2 | Non-matrisome | Non-matrisome | GO:CC extracellular matrix (GO:0005576, GO:0005578 & GO:0031012) & GO:CC not extracellular matrix | sp|Q96IY4|CBPB2_HUMAN | Carboxypeptidase B2 | 16.67 | 16.77 | 18.97 |
| 721 | CPE | Non-matrisome | Non-matrisome | GO:CC not extracellular matrix | sp|P16870|CBPE_HUMAN | Carboxypeptidase E | 16.19 | 19.44 | 18.39 |
| 722 | CPEB2 | Non-matrisome | Non-matrisome | GO:CC not extracellular matrix | sp|Q7Z5Q1|CPEB2_HUMAN | Cytoplasmic polyadenylation element-binding protein 2 | 19.24 | 17.7 | 17.62 |
| 723 | CPM | Non-matrisome | Non-matrisome | GO:CC extracellular matrix (GO:0005576, GO:0005578 & GO:0031012) & GO:CC not extracellular matrix | sp|P14384|CBPM_HUMAN | Carboxypeptidase M | 18.35 | 16.2 | 15.81 |
| 724 | CPNE1 | Non-matrisome | Non-matrisome | GO:CC not extracellular matrix | sp|Q99829|CPNE1_HUMAN | Copine-1 | 18.65 | 19.35 | 19.21 |
| 725 | CPNE2 | Non-matrisome | Non-matrisome | GO:CC not extracellular matrix | sp|Q96FN4|CPNE2_HUMAN | Copine-2 | 17 | 17.06 | 16.95 |
| 726 | CPNE3 | Non-matrisome | Non-matrisome | GO:CC not extracellular matrix | sp|O75131|CPNE3_HUMAN | Copine-3 | 19.63 | 20.45 | 20.37 |
| 727 | CPNE8 | Non-matrisome | Non-matrisome | GO:CC not extracellular matrix | sp|Q86YQ8|CPNE8_HUMAN | Copine-8 | 16.96 | 17.2 | 17.43 |
| 728 | CPOX | Non-matrisome | Non-matrisome | GO:CC not extracellular matrix | sp|P36551|HEM6_HUMAN | Oxygen-dependent coproporphyrinogen-III oxidase, mitochondrial | 17.51 | 18 | 17.89 |
| 729 | CPPED1 | Non-matrisome | Non-matrisome | GO:CC extracellular matrix (GO:0005576, GO:0005578 & GO:0031012) & GO:CC not extracellular matrix | sp|Q9BRF8|CPPED_HUMAN | Serine/threonine-protein phosphatase CPPED1 | 18.97 | 18.29 | 18.07 |
| 730 | CPQ | Non-matrisome | Non-matrisome | GO:CC not extracellular matrix | sp|Q9Y646|CBPQ_HUMAN | Carboxypeptidase Q | 16.38 | 18.32 | 18.67 |
| 731 | CPSF1 | Non-matrisome | Non-matrisome | GO:CC not extracellular matrix | sp|Q10570|CPSF1_HUMAN | Cleavage and polyadenylation specificity factor subunit 1 | 17.34 | 17.43 | 17.33 |
| 732 | CPSF2 | Non-matrisome | Non-matrisome | GO:CC not extracellular matrix | sp|Q9P2I0|CPSF2_HUMAN | Cleavage and polyadenylation specificity factor subunit 2 | 17.28 | 17.08 | 17.09 |
| 733 | CPSF3 | Non-matrisome | Non-matrisome | GO:CC not extracellular matrix | sp|Q9UKF6|CPSF3_HUMAN | Cleavage and polyadenylation specificity factor subunit 3 | 17.36 | 17.11 | 17.23 |
| 734 | CPSF6 | Non-matrisome | Non-matrisome | GO:CC not extracellular matrix | sp|Q16630|CPSF6_HUMAN | Cleavage and polyadenylation specificity factor subunit 6 | 17.21 | 17.57 | 17.45 |
| 735 | CPSF7 | Non-matrisome | Non-matrisome | GO:CC not extracellular matrix | sp|Q8N684|CPSF7_HUMAN | Cleavage and polyadenylation specificity factor subunit 7 | 17.62 | 17.58 | 17.69 |
| 736 | CPT1A | Non-matrisome | Non-matrisome | GO:CC not extracellular matrix | sp|P50416|CPT1A_HUMAN | Carnitine O-palmitoyltransferase 1, liver isoform | 17.25 | 18.43 | 18.51 |
| 737 | CPT2 | Non-matrisome | Non-matrisome | GO:CC extracellular matrix (GO:0005576, GO:0005578 & GO:0031012) & GO:CC not extracellular matrix | sp|P23786|CPT2_HUMAN | Carnitine O-palmitoyltransferase 2, mitochondrial | 17.1 | 17.59 | 17.5 |
| 738 | CPVL | Non-matrisome | Non-matrisome | GO:CC not extracellular matrix | sp|Q9H3G5|CPVL_HUMAN | Probable serine carboxypeptidase CPVL | 16.36 | 15.68 | 15.59 |
| 739 | CPZ | Non-matrisome | Non-matrisome | GO:CC not extracellular matrix | sp|Q66K79|CBPZ_HUMAN | Carboxypeptidase Z | 13.84 | 16.22 | 16.03 |
| 740 | CRABP1 | Non-matrisome | Non-matrisome | GO:CC not extracellular matrix | sp|P29762|RABP1_HUMAN | Cellular retinoic acid-binding protein 1 | 14.06 | 18.59 | 19.8 |
| 741 | CRABP2 | Non-matrisome | Non-matrisome | GO:CC not extracellular matrix | sp|P29373|RABP2_HUMAN | Cellular retinoic acid-binding protein 2 | 18.31 | 18.81 | 18.75 |
| 742 | CRADD | Non-matrisome | Non-matrisome | GO:CC not extracellular matrix | sp|P78560|CRADD_HUMAN | Death domain-containing protein CRADD | 16.32 | 16.59 | 16.7 |
| 743 | CRBN | Non-matrisome | Non-matrisome | GO:CC not extracellular matrix | sp|Q96SW2|CRBN_HUMAN | Protein cereblon | 15.26 | 16.05 | 16.11 |
| 744 | CREB1 | Non-matrisome | Non-matrisome | GO:CC not extracellular matrix | sp|P16220|CREB1_HUMAN | Cyclic AMP-responsive element-binding protein 1 | 17.14 | 17.04 | 17.24 |
| 745 | CREBRF | Non-matrisome | Non-matrisome | GO:CC not extracellular matrix | sp|Q8IUR6|CRERF_HUMAN | CREB3 regulatory factor | 12.82 | 13.61 | 13.31 |
| 746 | CRELD1 | Core matrisome | ECM Glycoproteins | GO:CC not extracellular matrix | sp|Q96HD1|CREL1_HUMAN | Protein disulfide isomerase CRELD1 | 17.55 | 17.68 | 17.73 |
| 747 | CRELD2 | Core matrisome | ECM Glycoproteins | GO:CC not extracellular matrix | sp|Q6UXH1|CREL2_HUMAN | Protein disulfide isomerase CRELD2 | 19.06 | 18.66 | 18.79 |
| 748 | CRIP1 | Non-matrisome | Non-matrisome | GO:CC not extracellular matrix | sp|P50238|CRIP1_HUMAN | Cysteine-rich protein 1 | 21.66 | 21.04 | 20.95 |
| 749 | CRIP2 | Non-matrisome | Non-matrisome | GO:CC not extracellular matrix | sp|P52943|CRIP2_HUMAN | Cysteine-rich protein 2 | 18.82 | 18.69 | 18.64 |
| 750 | CRISPLD2 | Core matrisome | ECM Glycoproteins | GO:CC extracellular matrix (GO:0005576, GO:0005578 & GO:0031012) & GO:CC not extracellular matrix | sp|Q9H0B8|CRLD2_HUMAN | Cysteine-rich secretory protein LCCL domain-containing 2 | 16.01 | 18.22 | 17.67 |
| 751 | CRK | Non-matrisome | Non-matrisome | GO:CC not extracellular matrix | sp|P46108|CRK_HUMAN | Adapter molecule crk | 17.76 | 17.7 | 17.79 |
| 752 | CRKL | Non-matrisome | Non-matrisome | GO:CC not extracellular matrix | sp|P46109|CRKL_HUMAN | Crk-like protein | 17.73 | 17.9 | 17.85 |
| 753 | CRLF3 | Matrisome-associated | Secreted Factors | GO:CC not extracellular matrix | sp|Q8IUI8|CRLF3_HUMAN | Cytokine receptor-like factor 3 | 17.57 | 17.21 | 17.09 |
| 754 | CRNKL1 | Non-matrisome | Non-matrisome | GO:CC not extracellular matrix | sp|Q9BZJ0|CRNL1_HUMAN | Crooked neck-like protein 1 | 17.9 | 17.7 | 17.66 |
| 755 | CRTAP | Non-matrisome | Non-matrisome | GO:CC not extracellular matrix | sp|O75718|CRTAP_HUMAN | Cartilage-associated protein | 18.13 | 17.79 | 17.75 |
| 756 | CRYAB | Non-matrisome | Non-matrisome | GO:CC not extracellular matrix | sp|P02511|CRYAB_HUMAN | Alpha-crystallin B chain | 19.11 | 18.16 | 18.21 |
| 757 | CRYBG1 | Non-matrisome | Non-matrisome | GO:CC not extracellular matrix | sp|Q9Y4K1|CRBG1_HUMAN | Beta/gamma crystallin domain-containing protein 1 | 17.44 | 17.1 | 17.14 |
| 758 | CRYBG3 | Non-matrisome | Non-matrisome | GO:CC not extracellular matrix | sp|Q68DQ2|CRBG3_HUMAN | Very large A-kinase anchor protein | 16.33 | 15.79 | 15.75 |
| 759 | CRYL1 | Non-matrisome | Non-matrisome | GO:CC not extracellular matrix | sp|Q9Y2S2|CRYL1_HUMAN | Lambda-crystallin homolog | 16.68 | 17.94 | 18.38 |
| 760 | CRYZ | Non-matrisome | Non-matrisome | GO:CC not extracellular matrix | sp|Q08257|QOR_HUMAN | Quinone oxidoreductase | 18.49 | 19.25 | 19.17 |
| 761 | CRYZL1 | Non-matrisome | Non-matrisome | GO:CC not extracellular matrix | sp|O95825|QORL1_HUMAN | Quinone oxidoreductase-like protein 1 | 17.63 | 17.69 | 17.62 |
| 762 | CS | Non-matrisome | Non-matrisome | GO:CC not extracellular matrix | sp|O75390|CISY_HUMAN | Citrate synthase, mitochondrial | 18.96 | 19.07 | 18.73 |
| 763 | CSDE1 | Non-matrisome | Non-matrisome | GO:CC not extracellular matrix | sp|O75534|CSDE1_HUMAN | Cold shock domain-containing protein E1 | 19.44 | 18.31 | 18.57 |
| 764 | CSE1L | Non-matrisome | Non-matrisome | GO:CC not extracellular matrix | sp|P55060|XPO2_HUMAN | Exportin-2 | 19.56 | 19.27 | 19.01 |
| 765 | CSK | Non-matrisome | Non-matrisome | GO:CC not extracellular matrix | sp|P41240|CSK_HUMAN | Tyrosine-protein kinase CSK | 18.48 | 18.39 | 18.27 |
| 766 | CSNK1A1 | Non-matrisome | Non-matrisome | GO:CC not extracellular matrix | sp|P48729|KC1A_HUMAN | Casein kinase I isoform alpha | 17.69 | 17.19 | 17.23 |
| 767 | CSNK1E | Non-matrisome | Non-matrisome | GO:CC not extracellular matrix | sp|P49674|KC1E_HUMAN | Casein kinase I isoform epsilon | 16.75 | 16.52 | 16.59 |
| 768 | CSNK2A1 | Non-matrisome | Non-matrisome | GO:CC not extracellular matrix | sp|P68400|CSK21_HUMAN | Casein kinase II subunit alpha | 16.08 | 15.99 | 16.16 |
| 769 | CSNK2A2 | Non-matrisome | Non-matrisome | GO:CC not extracellular matrix | sp|P19784|CSK22_HUMAN | Casein kinase II subunit alpha' | 17.75 | 17.73 | 17.71 |
| 770 | CSNK2B | Non-matrisome | Non-matrisome | GO:CC extracellular matrix (GO:0005576, GO:0005578 & GO:0031012) & GO:CC not extracellular matrix | sp|P67870|CSK2B_HUMAN | Casein kinase II subunit beta | 17.47 | 17.28 | 17.35 |
| 771 | CSPG4 | Matrisome-associated | ECM-affiliated Proteins | GO:CC extracellular matrix (GO:0005576, GO:0005578 & GO:0031012) & GO:CC not extracellular matrix | sp|Q6UVK1|CSPG4_HUMAN | Chondroitin sulfate proteoglycan 4 | 17.59 | 18.02 | 18.37 |
| 772 | CSRP1 | Non-matrisome | Non-matrisome | GO:CC not extracellular matrix | sp|P21291|CSRP1_HUMAN | Cysteine and glycine-rich protein 1 | 18.57 | 18.18 | 18.2 |
| 773 | CST3 | Matrisome-associated | ECM Regulators | GO:CC extracellular matrix (GO:0005576, GO:0005578 & GO:0031012) & GO:CC not extracellular matrix | sp|P01034|CYTC_HUMAN | Cystatin-C | 18.27 | 17.8 | 18 |
| 774 | CSTA | Matrisome-associated | ECM Regulators | GO:CC not extracellular matrix | sp|P01040|CYTA_HUMAN | Cystatin-A | 17.41 | 17.13 | 17.78 |
| 775 | CSTB | Matrisome-associated | ECM Regulators | GO:CC extracellular matrix (GO:0005576, GO:0005578 & GO:0031012) & GO:CC not extracellular matrix | sp|P04080|CYTB_HUMAN | Cystatin-B | 18.64 | 18.81 | 18.66 |
| 776 | CSTF2 | Non-matrisome | Non-matrisome | GO:CC not extracellular matrix | sp|P33240|CSTF2_HUMAN | Cleavage stimulation factor subunit 2 | 16.9 | 16.47 | 16.56 |
| 777 | CSTF3 | Non-matrisome | Non-matrisome | GO:CC not extracellular matrix | sp|Q12996|CSTF3_HUMAN | Cleavage stimulation factor subunit 3 | 17.89 | 17.78 | 17.74 |
| 778 | CTBP1 | Non-matrisome | Non-matrisome | GO:CC not extracellular matrix | sp|Q13363|CTBP1_HUMAN | C-terminal-binding protein 1 | 18.39 | 18.8 | 18.82 |
| 779 | CTBP2 | Non-matrisome | Non-matrisome | GO:CC not extracellular matrix | sp|P56545|CTBP2_HUMAN | C-terminal-binding protein 2 | 18.99 | 18.82 | 18.83 |
| 780 | CTBS | Non-matrisome | Non-matrisome | GO:CC not extracellular matrix | sp|Q01459|DIAC_HUMAN | Di-N-acetylchitobiase | 16.05 | 17.73 | 18.35 |
| 781 | CTDNEP1 | Non-matrisome | Non-matrisome | GO:CC not extracellular matrix | sp|O95476|CNEP1_HUMAN | CTD nuclear envelope phosphatase 1 | 17.97 | 17.86 | 17.77 |
| 782 | CTDP1 | Non-matrisome | Non-matrisome | GO:CC not extracellular matrix | sp|Q9Y5B0|CTDP1_HUMAN | RNA polymerase II subunit A C-terminal domain phosphatase | 17.26 | 17.31 | 17.41 |
| 783 | CTHRC1 | Core matrisome | ECM Glycoproteins | GO:CC extracellular matrix (GO:0005576, GO:0005578 & GO:0031012) & GO:CC not extracellular matrix | sp|Q96CG8|CTHR1_HUMAN | Collagen triple helix repeat-containing protein 1 | 18.71 | 18.25 | 18 |
| 784 | CTNNA1 | Non-matrisome | Non-matrisome | GO:CC not extracellular matrix | sp|P35221|CTNA1_HUMAN | Catenin alpha-1 | 19.05 | 18.39 | 18.44 |
| 785 | CTNNAL1 | Non-matrisome | Non-matrisome | GO:CC not extracellular matrix | sp|Q9UBT7|CTNL1_HUMAN | Alpha-catulin | 17.79 | 17.07 | 17.27 |
| 786 | CTNNB1 | Non-matrisome | Non-matrisome | GO:CC not extracellular matrix | sp|P35222|CTNB1_HUMAN | Catenin beta-1 | 18.13 | 17.69 | 17.64 |
| 787 | CTNNBIP1 | Non-matrisome | Non-matrisome | GO:CC not extracellular matrix | sp|Q9NSA3|CNBP1_HUMAN | Beta-catenin-interacting protein 1 | 14.99 | 14.41 | 15.54 |
| 788 | CTNNBL1 | Non-matrisome | Non-matrisome | GO:CC not extracellular matrix | sp|Q8WYA6|CTBL1_HUMAN | Beta-catenin-like protein 1 | 17.54 | 17.59 | 17.47 |
| 789 | CTNND1 | Non-matrisome | Non-matrisome | GO:CC not extracellular matrix | sp|O60716|CTND1_HUMAN | Catenin delta-1 | 18.79 | 18.36 | 18.48 |
| 790 | CTPS1 | Non-matrisome | Non-matrisome | GO:CC not extracellular matrix | sp|P17812|PYRG1_HUMAN | CTP synthase 1 | 19.32 | 18.53 | 18.12 |
| 791 | CTPS2 | Non-matrisome | Non-matrisome | GO:CC not extracellular matrix | sp|Q9NRF8|PYRG2_HUMAN | CTP synthase 2 | 16.88 | 17.14 | 16.95 |
| 792 | CTSA | Matrisome-associated | ECM Regulators | GO:CC extracellular matrix (GO:0005576, GO:0005578 & GO:0031012) & GO:CC not extracellular matrix | sp|P10619|PPGB_HUMAN | Lysosomal protective protein | 17.41 | 18.65 | 19.05 |
| 793 | CTSB | Matrisome-associated | ECM Regulators | GO:CC extracellular matrix (GO:0005576, GO:0005578 & GO:0031012) & GO:CC not extracellular matrix | sp|P07858|CATB_HUMAN | Cathepsin B | 17.66 | 18.92 | 19.4 |
| 794 | CTSC | Matrisome-associated | ECM Regulators | GO:CC extracellular matrix (GO:0005576, GO:0005578 & GO:0031012) & GO:CC not extracellular matrix | sp|P53634|CATC_HUMAN | Dipeptidyl peptidase 1 | 16.97 | 17.63 | 17.78 |
| 795 | CTSD | Matrisome-associated | ECM Regulators | GO:CC extracellular matrix (GO:0005576, GO:0005578 & GO:0031012) & GO:CC not extracellular matrix | sp|P07339|CATD_HUMAN | Cathepsin D | 17.31 | 18.97 | 18.87 |
| 796 | CTSL | Matrisome-associated | ECM Regulators | GO:CC extracellular matrix (GO:0005576, GO:0005578 & GO:0031012) & GO:CC not extracellular matrix | sp|P07711|CATL1_HUMAN | Procathepsin L | 15.89 | 17.23 | 17.29 |
| 797 | CTSS | Matrisome-associated | ECM Regulators | GO:CC extracellular matrix (GO:0005576, GO:0005578 & GO:0031012) & GO:CC not extracellular matrix | sp|P25774|CATS_HUMAN | Cathepsin S | 15.93 | 17.24 | 18 |
| 798 | CTSZ | Matrisome-associated | ECM Regulators | GO:CC extracellular matrix (GO:0005576, GO:0005578 & GO:0031012) & GO:CC not extracellular matrix | sp|Q9UBR2|CATZ_HUMAN | Cathepsin Z | 17.4 | 18.59 | 18.99 |
| 799 | CTTN | Non-matrisome | Non-matrisome | GO:CC not extracellular matrix | sp|Q14247|SRC8_HUMAN | Src substrate cortactin | 19.65 | 19.64 | 19.84 |
| 800 | CUL1 | Non-matrisome | Non-matrisome | GO:CC not extracellular matrix | sp|Q13616|CUL1_HUMAN | Cullin-1 | 18.23 | 18.37 | 18.23 |
| 801 | CUL2 | Non-matrisome | Non-matrisome | GO:CC not extracellular matrix | sp|Q13617|CUL2_HUMAN | Cullin-2 | 18.07 | 18.19 | 18.19 |
| 802 | CUL3 | Non-matrisome | Non-matrisome | GO:CC not extracellular matrix | sp|Q13618|CUL3_HUMAN | Cullin-3 | 17.66 | 17.87 | 17.8 |
| 803 | CUL4B | Non-matrisome | Non-matrisome | GO:CC not extracellular matrix | sp|Q13620|CUL4B_HUMAN | Cullin-4B | 18.11 | 17.98 | 17.85 |
| 804 | CUL5 | Non-matrisome | Non-matrisome | GO:CC not extracellular matrix | sp|Q93034|CUL5_HUMAN | Cullin-5 | 17.94 | 18.5 | 18.62 |
| 805 | CUTA | Non-matrisome | Non-matrisome | GO:CC not extracellular matrix | sp|O60888|CUTA_HUMAN | Protein CutA | 20.34 | 20.47 | 20.33 |
| 806 | CUTC | Non-matrisome | Non-matrisome | GO:CC not extracellular matrix | sp|Q9NTM9|CUTC_HUMAN | Copper homeostasis protein cutC homolog | 15.56 | 15.85 | 15.79 |
| 807 | CUX1 | Non-matrisome | Non-matrisome | GO:CC not extracellular matrix | sp|P39880|CUX1_HUMAN | Homeobox protein cut-like 1 | 17.04 | 16.46 | 16.6 |
| 808 | CWF19L1 | Non-matrisome | Non-matrisome | GO:CC not extracellular matrix | sp|Q69YN2|C19L1_HUMAN | CWF19-like protein 1 | 16.98 | 16.46 | 16.34 |
| 809 | CYB561D2 | Non-matrisome | Non-matrisome | GO:CC not extracellular matrix | sp|O14569|C56D2_HUMAN | Transmembrane reductase CYB561D2 | 13.64 | 12.8 | 12.73 |
| 810 | CYB5A | Non-matrisome | Non-matrisome | GO:CC not extracellular matrix | sp|P00167|CYB5_HUMAN | Cytochrome b5 | 20.62 | 20.7 | 20.61 |
| 811 | CYB5B | Non-matrisome | Non-matrisome | GO:CC not extracellular matrix | sp|O43169|CYB5B_HUMAN | Cytochrome b5 type B | 17.94 | 18.03 | 18.13 |
| 812 | CYB5R1 | Non-matrisome | Non-matrisome | GO:CC not extracellular matrix | sp|Q9UHQ9|NB5R1_HUMAN | NADH-cytochrome b5 reductase 1 | 18.88 | 19.97 | 19.92 |
| 813 | CYB5R2 | Non-matrisome | Non-matrisome | GO:CC not extracellular matrix | sp|Q6BCY4|NB5R2_HUMAN | NADH-cytochrome b5 reductase 2 | 17.17 | 17.72 | 17.81 |
| 814 | CYB5R3 | Non-matrisome | Non-matrisome | GO:CC extracellular matrix (GO:0005576, GO:0005578 & GO:0031012) & GO:CC not extracellular matrix | sp|P00387|NB5R3_HUMAN | NADH-cytochrome b5 reductase 3 | 17.84 | 18.26 | 18.02 |
| 815 | CYBA | Non-matrisome | Non-matrisome | GO:CC not extracellular matrix | sp|P13498|CY24A_HUMAN | Cytochrome b-245 light chain | 17.58 | 16.18 | 16.32 |
| 816 | CYBC1 | Non-matrisome | Non-matrisome | GO:CC not extracellular matrix | sp|Q9BQA9|CYBC1_HUMAN | Cytochrome b-245 chaperone 1 | 17.52 | 18.17 | 18.15 |
| 817 | CYBRD1 | Non-matrisome | Non-matrisome | GO:CC not extracellular matrix | sp|Q53TN4|CYBR1_HUMAN | Plasma membrane ascorbate-dependent reductase CYBRD1 | 15.87 | 17.21 | 17.62 |
| 818 | CYC1 | Non-matrisome | Non-matrisome | GO:CC not extracellular matrix | sp|P08574|CY1_HUMAN | Cytochrome c1, heme protein, mitochondrial | 16.85 | 17.43 | 17.1 |
| 819 | CYCS | Non-matrisome | Non-matrisome | GO:CC not extracellular matrix | sp|P99999|CYC_HUMAN | Cytochrome c | 19.27 | 19.04 | 18.81 |
| 820 | CYFIP1 | Non-matrisome | Non-matrisome | GO:CC extracellular matrix (GO:0005576, GO:0005578 & GO:0031012) & GO:CC not extracellular matrix | sp|Q7L576|CYFP1_HUMAN | Cytoplasmic FMR1-interacting protein 1 | 16.84 | 17.03 | 16.81 |
| 821 | CYFIP2 | Non-matrisome | Non-matrisome | GO:CC not extracellular matrix | sp|Q96F07|CYFP2_HUMAN | Cytoplasmic FMR1-interacting protein 2 | 15.61 | 16.89 | 17.05 |
| 822 | CYGB | Non-matrisome | Non-matrisome | GO:CC not extracellular matrix | sp|Q8WWM9|CYGB_HUMAN | Cytoglobin | 15.92 | 17.86 | 18.13 |
| 823 | CYP1B1 | Non-matrisome | Non-matrisome | GO:CC not extracellular matrix | sp|Q16678|CP1B1_HUMAN | Cytochrome P450 1B1 | 15.08 | 17.59 | 17.32 |
| 824 | CYP51A1 | Non-matrisome | Non-matrisome | GO:CC not extracellular matrix | sp|Q16850|CP51A_HUMAN | Lanosterol 14-alpha demethylase | 18.21 | 17.24 | 17.15 |
| 825 | CYRIB | Non-matrisome | Non-matrisome | GO:CC extracellular matrix (GO:0005576, GO:0005578 & GO:0031012) & GO:CC not extracellular matrix | sp|Q9NUQ9|CYRIB_HUMAN | CYFIP-related Rac1 interactor B | 15.97 | 16.14 | 16.06 |
| 826 | CZIB | Non-matrisome | Non-matrisome | GO:CC not extracellular matrix | sp|Q9NWV4|CZIB_HUMAN | CXXC motif containing zinc binding protein | 19.22 | 19.33 | 19.4 |
| 827 | DAAM1 | Non-matrisome | Non-matrisome | GO:CC not extracellular matrix | sp|Q9Y4D1|DAAM1_HUMAN | Disheveled-associated activator of morphogenesis 1 | 14.22 | 14.54 | 14.21 |
| 828 | DAB2 | Non-matrisome | Non-matrisome | GO:CC not extracellular matrix | sp|P98082|DAB2_HUMAN | Disabled homolog 2 | 18.96 | 17.61 | 17.55 |
| 829 | DAD1 | Non-matrisome | Non-matrisome | GO:CC not extracellular matrix | sp|P61803|DAD1_HUMAN | Dolichyl-diphosphooligosaccharide--protein glycosyltransferase subunit DAD1 | 19.68 | 19.94 | 19.98 |
| 830 | DAG1 | Non-matrisome | Non-matrisome | GO:CC extracellular matrix (GO:0005576, GO:0005578 & GO:0031012) & GO:CC not extracellular matrix | sp|Q14118|DAG1_HUMAN | Dystroglycan 1 | 18.29 | 18.83 | 18.82 |
| 831 | DAP3 | Non-matrisome | Non-matrisome | GO:CC not extracellular matrix | sp|P51398|RT29_HUMAN | Small ribosomal subunit protein mS29 | 18.9 | 18.49 | 18.33 |
| 832 | DARS1 | Non-matrisome | Non-matrisome | GO:CC not extracellular matrix | sp|P14868|SYDC_HUMAN | Aspartate--tRNA ligase, cytoplasmic | 18.93 | 19.06 | 18.66 |
| 833 | DARS2 | Non-matrisome | Non-matrisome | GO:CC not extracellular matrix | sp|Q6PI48|SYDM_HUMAN | Aspartate--tRNA ligase, mitochondrial | 18.43 | 18.45 | 18.07 |
| 834 | DAZAP1 | Non-matrisome | Non-matrisome | GO:CC not extracellular matrix | sp|Q96EP5|DAZP1_HUMAN | DAZ-associated protein 1 | 19.99 | 20.13 | 19.97 |
| 835 | DBI | Non-matrisome | Non-matrisome | GO:CC not extracellular matrix | sp|P07108|ACBP_HUMAN | Acyl-CoA-binding protein | 18.58 | 18.63 | 18.73 |
| 836 | DBN1 | Non-matrisome | Non-matrisome | GO:CC not extracellular matrix | sp|Q16643|DREB_HUMAN | Drebrin | 18.41 | 17.63 | 17.74 |
| 837 | DBNL | Non-matrisome | Non-matrisome | GO:CC extracellular matrix (GO:0005576, GO:0005578 & GO:0031012) & GO:CC not extracellular matrix | sp|Q9UJU6|DBNL_HUMAN | Drebrin-like protein | 18.91 | 18.68 | 18.85 |
| 838 | DBR1 | Non-matrisome | Non-matrisome | GO:CC not extracellular matrix | sp|Q9UK59|DBR1_HUMAN | Lariat debranching enzyme | 17.19 | 17.21 | 17.19 |
| 839 | DBT | Non-matrisome | Non-matrisome | GO:CC not extracellular matrix | sp|P11182|ODB2_HUMAN | Lipoamide acyltransferase component of branched-chain alpha-keto acid dehydrogenase complex, mitochondrial | 17.82 | 18.56 | 18.34 |
| 840 | DCAF8 | Non-matrisome | Non-matrisome | GO:CC not extracellular matrix | sp|Q5TAQ9|DCAF8_HUMAN | DDB1- and CUL4-associated factor 8 | 15.81 | 16.3 | 16.33 |
| 841 | DCD | Non-matrisome | Non-matrisome | GO:CC extracellular matrix (GO:0005576, GO:0005578 & GO:0031012) & GO:CC not extracellular matrix | sp|P81605|DCD_HUMAN | Dermcidin | 18.76 | 18.36 | 18.78 |
| 842 | DCLK1 | Non-matrisome | Non-matrisome | GO:CC not extracellular matrix | sp|O15075|DCLK1_HUMAN | Serine/threonine-protein kinase DCLK1 | 16.07 | 16.92 | 17.03 |
| 843 | DCN | Core matrisome | Proteoglycans | GO:CC extracellular matrix (GO:0005576, GO:0005578 & GO:0031012) & GO:CC not extracellular matrix | sp|P07585|PGS2_HUMAN | Decorin | 16.84 | 20.06 | 19.55 |
| 844 | DCPS | Non-matrisome | Non-matrisome | GO:CC not extracellular matrix | sp|Q96C86|DCPS_HUMAN | m7GpppX diphosphatase | 19.5 | 20.05 | 19.82 |
| 845 | DCTD | Non-matrisome | Non-matrisome | GO:CC not extracellular matrix | sp|P32321|DCTD_HUMAN | Deoxycytidylate deaminase | 18.35 | 18.06 | 17.99 |
| 846 | DCTN1 | Non-matrisome | Non-matrisome | GO:CC not extracellular matrix | sp|Q14203|DCTN1_HUMAN | Dynactin subunit 1 | 18.54 | 18.45 | 18.41 |
| 847 | DCTN2 | Non-matrisome | Non-matrisome | GO:CC not extracellular matrix | sp|Q13561|DCTN2_HUMAN | Dynactin subunit 2 | 20.36 | 20.04 | 20.09 |
| 848 | DCTN3 | Non-matrisome | Non-matrisome | GO:CC not extracellular matrix | sp|O75935|DCTN3_HUMAN | Dynactin subunit 3 | 20.37 | 20.22 | 20.15 |
| 849 | DCTN4 | Non-matrisome | Non-matrisome | GO:CC not extracellular matrix | sp|Q9UJW0|DCTN4_HUMAN | Dynactin subunit 4 | 18.11 | 18.03 | 17.94 |
| 850 | DCTN5 | Non-matrisome | Non-matrisome | GO:CC not extracellular matrix | sp|Q9BTE1|DCTN5_HUMAN | Dynactin subunit 5 | 18.52 | 18.43 | 18.3 |
| 851 | DCTN6 | Non-matrisome | Non-matrisome | GO:CC not extracellular matrix | sp|O00399|DCTN6_HUMAN | Dynactin subunit 6 | 18.43 | 18.37 | 18.21 |
| 852 | DCUN1D1 | Non-matrisome | Non-matrisome | GO:CC not extracellular matrix | sp|Q96GG9|DCNL1_HUMAN | DCN1-like protein 1 | 18.29 | 18.27 | 18.46 |
| 853 | DCUN1D5 | Non-matrisome | Non-matrisome | GO:CC not extracellular matrix | sp|Q9BTE7|DCNL5_HUMAN | DCN1-like protein 5 | 19.71 | 18.61 | 18.75 |
| 854 | DCXR | Non-matrisome | Non-matrisome | GO:CC not extracellular matrix | sp|Q7Z4W1|DCXR_HUMAN | L-xylulose reductase | 18.41 | 19.1 | 18.77 |
| 855 | DDAH1 | Non-matrisome | Non-matrisome | GO:CC not extracellular matrix | sp|O94760|DDAH1_HUMAN | N(G),N(G)-dimethylarginine dimethylaminohydrolase 1 | 18.09 | 18.44 | 18.35 |
| 856 | DDAH2 | Non-matrisome | Non-matrisome | GO:CC not extracellular matrix | sp|O95865|DDAH2_HUMAN | N(G),N(G)-dimethylarginine dimethylaminohydrolase 2 | 18.36 | 19.36 | 19.4 |
| 857 | DDB1 | Non-matrisome | Non-matrisome | GO:CC not extracellular matrix | sp|Q16531|DDB1_HUMAN | DNA damage-binding protein 1 | 18.16 | 18.43 | 18.41 |
| 858 | DDB2 | Non-matrisome | Non-matrisome | GO:CC not extracellular matrix | sp|Q92466|DDB2_HUMAN | DNA damage-binding protein 2 | 17.31 | 18.6 | 18.42 |
| 859 | DDI2 | Non-matrisome | Non-matrisome | GO:CC not extracellular matrix | sp|Q5TDH0|DDI2_HUMAN | Protein DDI1 homolog 2 | 17.4 | 17.5 | 17.62 |
| 860 | DDOST | Non-matrisome | Non-matrisome | GO:CC not extracellular matrix | sp|P39656|OST48_HUMAN | Dolichyl-diphosphooligosaccharide--protein glycosyltransferase 48 kDa subunit | 18.48 | 18.83 | 18.7 |
| 861 | DDRGK1 | Non-matrisome | Non-matrisome | GO:CC not extracellular matrix | sp|Q96HY6|DDRGK_HUMAN | DDRGK domain-containing protein 1 | 17.92 | 17.69 | 17.72 |
| 862 | DDT | Non-matrisome | Non-matrisome | GO:CC not extracellular matrix | sp|P30046|DOPD_HUMAN | D-dopachrome decarboxylase | 20.04 | 20.28 | 20.3 |
| 863 | DDX1 | Non-matrisome | Non-matrisome | GO:CC not extracellular matrix | sp|Q92499|DDX1_HUMAN | ATP-dependent RNA helicase DDX1 | 18.56 | 18.74 | 18.69 |
| 864 | DDX10 | Non-matrisome | Non-matrisome | GO:CC not extracellular matrix | sp|Q13206|DDX10_HUMAN | Probable ATP-dependent RNA helicase DDX10 | 16.89 | 16.11 | 16.12 |
| 865 | DDX17 | Non-matrisome | Non-matrisome | GO:CC not extracellular matrix | sp|Q92841|DDX17_HUMAN | Probable ATP-dependent RNA helicase DDX17 | 17.91 | 18.43 | 18.31 |
| 866 | DDX18 | Non-matrisome | Non-matrisome | GO:CC not extracellular matrix | sp|Q9NVP1|DDX18_HUMAN | ATP-dependent RNA helicase DDX18 | 17.89 | 17.13 | 16.94 |
| 867 | DDX19A | Non-matrisome | Non-matrisome | GO:CC not extracellular matrix | sp|Q9NUU7|DD19A_HUMAN | ATP-dependent RNA helicase DDX19A | 17.4 | 16.72 | 17 |
| 868 | DDX19B | Non-matrisome | Non-matrisome | GO:CC not extracellular matrix | sp|Q9UMR2|DD19B_HUMAN | ATP-dependent RNA helicase DDX19B | 15.25 | 15.98 | 15.76 |
| 869 | DDX20 | Non-matrisome | Non-matrisome | GO:CC not extracellular matrix | sp|Q9UHI6|DDX20_HUMAN | Probable ATP-dependent RNA helicase DDX20 | 17.49 | 16.79 | 16.79 |
| 870 | DDX21 | Non-matrisome | Non-matrisome | GO:CC not extracellular matrix | sp|Q9NR30|DDX21_HUMAN | Nucleolar RNA helicase 2 | 19.11 | 16.69 | 16.86 |
| 871 | DDX23 | Non-matrisome | Non-matrisome | GO:CC not extracellular matrix | sp|Q9BUQ8|DDX23_HUMAN | Probable ATP-dependent RNA helicase DDX23 | 18.29 | 18.58 | 18.37 |
| 872 | DDX24 | Non-matrisome | Non-matrisome | GO:CC not extracellular matrix | sp|Q9GZR7|DDX24_HUMAN | ATP-dependent RNA helicase DDX24 | 18.36 | 18.39 | 18.41 |
| 873 | DDX27 | Non-matrisome | Non-matrisome | GO:CC not extracellular matrix | sp|Q96GQ7|DDX27_HUMAN | Probable ATP-dependent RNA helicase DDX27 | 18.82 | 18.45 | 18.38 |
| 874 | DDX31 | Non-matrisome | Non-matrisome | GO:CC not extracellular matrix | sp|Q9H8H2|DDX31_HUMAN | Probable ATP-dependent RNA helicase DDX31 | 16.96 | 16.56 | 16.48 |
| 875 | DDX39A | Non-matrisome | Non-matrisome | GO:CC not extracellular matrix | sp|O00148|DX39A_HUMAN | ATP-dependent RNA helicase DDX39A | 19.41 | 18.9 | 18.49 |
| 876 | DDX39B | Non-matrisome | Non-matrisome | GO:CC not extracellular matrix | sp|Q13838|DX39B_HUMAN | Spliceosome RNA helicase DDX39B | 17.28 | 17.49 | 17.49 |
| 877 | DDX3X | Non-matrisome | Non-matrisome | GO:CC extracellular matrix (GO:0005576, GO:0005578 & GO:0031012) & GO:CC not extracellular matrix | sp|O00571|DDX3X_HUMAN | ATP-dependent RNA helicase DDX3X | 18.3 | 17.84 | 17.93 |
| 878 | DDX41 | Non-matrisome | Non-matrisome | GO:CC not extracellular matrix | sp|Q9UJV9|DDX41_HUMAN | Probable ATP-dependent RNA helicase DDX41 | 17.71 | 17.75 | 17.67 |
| 879 | DDX42 | Non-matrisome | Non-matrisome | GO:CC not extracellular matrix | sp|Q86XP3|DDX42_HUMAN | ATP-dependent RNA helicase DDX42 | 18.91 | 18.28 | 18.44 |
| 880 | DDX46 | Non-matrisome | Non-matrisome | GO:CC not extracellular matrix | sp|Q7L014|DDX46_HUMAN | Probable ATP-dependent RNA helicase DDX46 | 18.25 | 18.38 | 18.28 |
| 881 | DDX5 | Non-matrisome | Non-matrisome | GO:CC not extracellular matrix | sp|P17844|DDX5_HUMAN | Probable ATP-dependent RNA helicase DDX5 | 19.03 | 17.88 | 18.33 |
| 882 | DDX50 | Non-matrisome | Non-matrisome | GO:CC not extracellular matrix | sp|Q9BQ39|DDX50_HUMAN | ATP-dependent RNA helicase DDX50 | 17.3 | 16.44 | 16.59 |
| 883 | DDX52 | Non-matrisome | Non-matrisome | GO:CC not extracellular matrix | sp|Q9Y2R4|DDX52_HUMAN | Probable ATP-dependent RNA helicase DDX52 | 18.51 | 17.73 | 17.7 |
| 884 | DDX54 | Non-matrisome | Non-matrisome | GO:CC not extracellular matrix | sp|Q8TDD1|DDX54_HUMAN | ATP-dependent RNA helicase DDX54 | 17.44 | 16.9 | 16.94 |
| 885 | DDX59 | Non-matrisome | Non-matrisome | GO:CC not extracellular matrix | sp|Q5T1V6|DDX59_HUMAN | Probable ATP-dependent RNA helicase DDX59 | 15.4 | 15.85 | 16.05 |
| 886 | DDX6 | Non-matrisome | Non-matrisome | GO:CC not extracellular matrix | sp|P26196|DDX6_HUMAN | Probable ATP-dependent RNA helicase DDX6 | 18.64 | 18.27 | 18.37 |
| 887 | DDX60 | Non-matrisome | Non-matrisome | GO:CC not extracellular matrix | sp|Q8IY21|DDX60_HUMAN | Probable ATP-dependent RNA helicase DDX60 | 15.4 | 15.99 | 15.83 |
| 888 | DECR1 | Non-matrisome | Non-matrisome | GO:CC not extracellular matrix | sp|Q16698|DECR_HUMAN | 2,4-dienoyl-CoA reductase [(3E)-enoyl-CoA-producing], mitochondrial | 17.93 | 18.93 | 18.66 |
| 889 | DEGS1 | Non-matrisome | Non-matrisome | GO:CC not extracellular matrix | sp|O15121|DEGS1_HUMAN | Sphingolipid delta(4)-desaturase DES1 | 17.86 | 17.65 | 17.91 |
| 890 | DEK | Non-matrisome | Non-matrisome | GO:CC not extracellular matrix | sp|P35659|DEK_HUMAN | Protein DEK | 18.09 | 18.41 | 18.15 |
| 891 | DENND4C | Non-matrisome | Non-matrisome | GO:CC not extracellular matrix | sp|Q5VZ89|DEN4C_HUMAN | DENN domain-containing protein 4C | 16.49 | 16.96 | 16.99 |
| 892 | DENR | Non-matrisome | Non-matrisome | GO:CC extracellular matrix (GO:0005576, GO:0005578 & GO:0031012) & GO:CC not extracellular matrix | sp|O43583|DENR_HUMAN | Density-regulated protein | 19.28 | 19.01 | 18.98 |
| 893 | DERA | Non-matrisome | Non-matrisome | GO:CC extracellular matrix (GO:0005576, GO:0005578 & GO:0031012) & GO:CC not extracellular matrix | sp|Q9Y315|DEOC_HUMAN | Deoxyribose-phosphate aldolase | 18.96 | 18.95 | 18.74 |
| 894 | DERL1 | Non-matrisome | Non-matrisome | GO:CC not extracellular matrix | sp|Q9BUN8|DERL1_HUMAN | Derlin-1 | 17.58 | 17.51 | 17.37 |
| 895 | DERPC | Non-matrisome | Non-matrisome | GO:CC not extracellular matrix | sp|P0CG12|DERPC_HUMAN | Decreased expression in renal and prostate cancer protein | 17.64 | 17.49 | 17.59 |
| 896 | DES | Non-matrisome | Non-matrisome | GO:CC not extracellular matrix | sp|P17661|DESM_HUMAN | Desmin | 15.18 | 17.54 | 17.15 |
| 897 | DGKA | Non-matrisome | Non-matrisome | GO:CC not extracellular matrix | sp|P23743|DGKA_HUMAN | Diacylglycerol kinase alpha | 18.18 | 18.88 | 18.78 |
| 898 | DGKQ | Non-matrisome | Non-matrisome | GO:CC not extracellular matrix | sp|P52824|DGKQ_HUMAN | Diacylglycerol kinase theta | 15.54 | 15.69 | 15.9 |
| 899 | DGLUCY | Non-matrisome | Non-matrisome | GO:CC not extracellular matrix | sp|Q7Z3D6|GLUCM_HUMAN | D-glutamate cyclase, mitochondrial | 16.03 | 17.48 | 17.19 |
| 900 | DHCR24 | Non-matrisome | Non-matrisome | GO:CC not extracellular matrix | sp|Q15392|DHC24_HUMAN | Delta(24)-sterol reductase | 19.39 | 17.8 | 17.98 |
| 901 | DHCR7 | Non-matrisome | Non-matrisome | GO:CC not extracellular matrix | sp|Q9UBM7|DHCR7_HUMAN | 7-dehydrocholesterol reductase | 17.37 | 17.15 | 16.98 |
| 902 | DHRS1 | Non-matrisome | Non-matrisome | GO:CC not extracellular matrix | sp|Q96LJ7|DHRS1_HUMAN | Dehydrogenase/reductase SDR family member 1 | 18.03 | 18.77 | 18.94 |
| 903 | DHRS11 | Non-matrisome | Non-matrisome | GO:CC extracellular matrix (GO:0005576, GO:0005578 & GO:0031012) & GO:CC not extracellular matrix | sp|Q6UWP2|DHR11_HUMAN | Dehydrogenase/reductase SDR family member 11 | 16.07 | 16.21 | 16.28 |
| 904 | DHRS4 | Non-matrisome | Non-matrisome | GO:CC not extracellular matrix | sp|Q9BTZ2|DHRS4_HUMAN | Dehydrogenase/reductase SDR family member 4 | 19.28 | 19.4 | 18.98 |
| 905 | DHRS7 | Non-matrisome | Non-matrisome | GO:CC not extracellular matrix | sp|Q9Y394|DHRS7_HUMAN | Dehydrogenase/reductase SDR family member 7 | 18.41 | 19.09 | 19.04 |
| 906 | DHRS7B | Non-matrisome | Non-matrisome | GO:CC not extracellular matrix | sp|Q6IAN0|DRS7B_HUMAN | Dehydrogenase/reductase SDR family member 7B | 18.49 | 18.72 | 18.65 |
| 907 | DHTKD1 | Non-matrisome | Non-matrisome | GO:CC not extracellular matrix | sp|Q96HY7|DHTK1_HUMAN | 2-oxoadipate dehydrogenase complex component E1 | 18 | 18.52 | 18 |
| 908 | DHX15 | Non-matrisome | Non-matrisome | GO:CC not extracellular matrix | sp|O43143|DHX15_HUMAN | ATP-dependent RNA helicase DHX15 | 18.52 | 18.39 | 18.37 |
| 909 | DHX16 | Non-matrisome | Non-matrisome | GO:CC not extracellular matrix | sp|O60231|DHX16_HUMAN | Pre-mRNA-splicing factor ATP-dependent RNA helicase DHX16 | 17.4 | 16.87 | 16.93 |
| 910 | DHX29 | Non-matrisome | Non-matrisome | GO:CC not extracellular matrix | sp|Q7Z478|DHX29_HUMAN | ATP-dependent RNA helicase DHX29 | 17.96 | 17.48 | 17.63 |
| 911 | DHX30 | Non-matrisome | Non-matrisome | GO:CC not extracellular matrix | sp|Q7L2E3|DHX30_HUMAN | ATP-dependent RNA helicase DHX30 | 18.02 | 17.74 | 17.83 |
| 912 | DHX32 | Non-matrisome | Non-matrisome | GO:CC not extracellular matrix | sp|Q7L7V1|DHX32_HUMAN | Putative pre-mRNA-splicing factor ATP-dependent RNA helicase DHX32 | 15.43 | 15.2 | 15.3 |
| 913 | DHX36 | Non-matrisome | Non-matrisome | GO:CC not extracellular matrix | sp|Q9H2U1|DHX36_HUMAN | ATP-dependent DNA/RNA helicase DHX36 | 17.35 | 17.06 | 17.25 |
| 914 | DHX38 | Non-matrisome | Non-matrisome | GO:CC not extracellular matrix | sp|Q92620|PRP16_HUMAN | Pre-mRNA-splicing factor ATP-dependent RNA helicase PRP16 | 17.39 | 17.32 | 17.34 |
| 915 | DHX57 | Non-matrisome | Non-matrisome | GO:CC not extracellular matrix | sp|Q6P158|DHX57_HUMAN | Putative ATP-dependent RNA helicase DHX57 | 17.02 | 16.53 | 16.53 |
| 916 | DHX8 | Non-matrisome | Non-matrisome | GO:CC not extracellular matrix | sp|Q14562|DHX8_HUMAN | ATP-dependent RNA helicase DHX8 | 16.97 | 16.65 | 16.83 |
| 917 | DHX9 | Non-matrisome | Non-matrisome | GO:CC not extracellular matrix | sp|Q08211|DHX9_HUMAN | ATP-dependent RNA helicase A | 18.82 | 18.95 | 18.9 |
| 918 | DIABLO | Non-matrisome | Non-matrisome | GO:CC not extracellular matrix | sp|Q9NR28|DBLOH_HUMAN | Diablo IAP-binding mitochondrial protein | 17.68 | 17.97 | 18.07 |
| 919 | DIAPH1 | Non-matrisome | Non-matrisome | GO:CC not extracellular matrix | sp|O60610|DIAP1_HUMAN | Protein diaphanous homolog 1 | 18.58 | 18.15 | 18.05 |
| 920 | DIAPH2 | Non-matrisome | Non-matrisome | GO:CC not extracellular matrix | sp|O60879|DIAP2_HUMAN | Protein diaphanous homolog 2 | 17.4 | 17.3 | 17.27 |
| 921 | DICER1 | Non-matrisome | Non-matrisome | GO:CC not extracellular matrix | sp|Q9UPY3|DICER_HUMAN | Endoribonuclease Dicer | 17.01 | 16.48 | 16.29 |
| 922 | DIMT1 | Non-matrisome | Non-matrisome | GO:CC not extracellular matrix | sp|Q9UNQ2|DIM1_HUMAN | Probable dimethyladenosine transferase | 18.15 | 17.03 | 16.99 |
| 923 | DIP2A | Non-matrisome | Non-matrisome | GO:CC not extracellular matrix | sp|Q14689|DIP2A_HUMAN | Disco-interacting protein 2 homolog A | 16.08 | 16.34 | 16.36 |
| 924 | DIP2B | Non-matrisome | Non-matrisome | GO:CC not extracellular matrix | sp|Q9P265|DIP2B_HUMAN | Disco-interacting protein 2 homolog B | 17.26 | 17.45 | 17.52 |
| 925 | DIS3 | Non-matrisome | Non-matrisome | GO:CC not extracellular matrix | sp|Q9Y2L1|RRP44_HUMAN | Exosome complex exonuclease RRP44 | 17.49 | 17.28 | 17.15 |
| 926 | DKC1 | Non-matrisome | Non-matrisome | GO:CC not extracellular matrix | sp|O60832|DKC1_HUMAN | H/ACA ribonucleoprotein complex subunit DKC1 | 18.85 | 19.04 | 18.98 |
| 927 | DLAT | Non-matrisome | Non-matrisome | GO:CC not extracellular matrix | sp|P10515|ODP2_HUMAN | Dihydrolipoyllysine-residue acetyltransferase component of pyruvate dehydrogenase complex, mitochondrial | 18.14 | 18.58 | 18.27 |
| 928 | DLD | Non-matrisome | Non-matrisome | GO:CC not extracellular matrix | sp|P09622|DLDH_HUMAN | Dihydrolipoyl dehydrogenase, mitochondrial | 18.26 | 18.56 | 18.36 |
| 929 | DLG1 | Non-matrisome | Non-matrisome | GO:CC not extracellular matrix | sp|Q12959|DLG1_HUMAN | Disks large homolog 1 | 17.25 | 17.93 | 17.88 |
| 930 | DLST | Non-matrisome | Non-matrisome | GO:CC not extracellular matrix | sp|P36957|ODO2_HUMAN | Dihydrolipoyllysine-residue succinyltransferase component of 2-oxoglutarate dehydrogenase complex, mitochondrial | 17.44 | 18.07 | 17.65 |
| 931 | DMAC2L | Non-matrisome | Non-matrisome | GO:CC not extracellular matrix | sp|Q99766|ATP5S_HUMAN | ATP synthase subunit s, mitochondrial | 17.71 | 18.4 | 18.23 |
| 932 | DMBT1 | Core matrisome | ECM Glycoproteins | GO:CC extracellular matrix (GO:0005576, GO:0005578 & GO:0031012) & GO:CC not extracellular matrix | sp|Q9UGM3|DMBT1_HUMAN | Deleted in malignant brain tumors 1 protein | 16.04 | 16.28 | 15.88 |
| 933 | DMD | Non-matrisome | Non-matrisome | GO:CC not extracellular matrix | sp|P11532|DMD_HUMAN | Dystrophin | 16.68 | 15.72 | 16.08 |
| 934 | DNAAF5 | Non-matrisome | Non-matrisome | GO:CC not extracellular matrix | sp|Q86Y56|DAAF5_HUMAN | Dynein axonemal assembly factor 5 | 18.28 | 17.72 | 17.75 |
| 935 | DNAJA1 | Non-matrisome | Non-matrisome | GO:CC not extracellular matrix | sp|P31689|DNJA1_HUMAN | DnaJ homolog subfamily A member 1 | 17.88 | 17.39 | 17.63 |
| 936 | DNAJA2 | Non-matrisome | Non-matrisome | GO:CC not extracellular matrix | sp|O60884|DNJA2_HUMAN | DnaJ homolog subfamily A member 2 | 19.15 | 18.97 | 19.09 |
| 937 | DNAJA3 | Non-matrisome | Non-matrisome | GO:CC not extracellular matrix | sp|Q96EY1|DNJA3_HUMAN | DnaJ homolog subfamily A member 3, mitochondrial | 16.81 | 16.76 | 16.6 |
| 938 | DNAJB1 | Non-matrisome | Non-matrisome | GO:CC not extracellular matrix | sp|P25685|DNJB1_HUMAN | DnaJ homolog subfamily B member 1 | 18.34 | 18.16 | 18.31 |
| 939 | DNAJB11 | Non-matrisome | Non-matrisome | GO:CC not extracellular matrix | sp|Q9UBS4|DJB11_HUMAN | DnaJ homolog subfamily B member 11 | 19.56 | 19.34 | 19.34 |
| 940 | DNAJB2 | Non-matrisome | Non-matrisome | GO:CC not extracellular matrix | sp|P25686|DNJB2_HUMAN | DnaJ homolog subfamily B member 2 | 15.87 | 16.66 | 16.34 |
| 941 | DNAJB6 | Non-matrisome | Non-matrisome | GO:CC not extracellular matrix | sp|O75190|DNJB6_HUMAN | DnaJ homolog subfamily B member 6 | 16.22 | 15.68 | 15.78 |
| 942 | DNAJB9 | Non-matrisome | Non-matrisome | GO:CC not extracellular matrix | sp|Q9UBS3|DNJB9_HUMAN | DnaJ homolog subfamily B member 9 | 14.43 | 14.83 | 14.78 |
| 943 | DNAJC1 | Non-matrisome | Non-matrisome | GO:CC not extracellular matrix | sp|Q96KC8|DNJC1_HUMAN | DnaJ homolog subfamily C member 1 | 16.57 | 16.2 | 16.27 |
| 944 | DNAJC10 | Non-matrisome | Non-matrisome | GO:CC not extracellular matrix | sp|Q8IXB1|DJC10_HUMAN | DnaJ homolog subfamily C member 10 | 17.68 | 17.72 | 17.71 |
| 945 | DNAJC11 | Non-matrisome | Non-matrisome | GO:CC not extracellular matrix | sp|Q9NVH1|DJC11_HUMAN | DnaJ homolog subfamily C member 11 | 17.36 | 17.97 | 17.7 |
| 946 | DNAJC13 | Non-matrisome | Non-matrisome | GO:CC not extracellular matrix | sp|O75165|DJC13_HUMAN | DnaJ homolog subfamily C member 13 | 17.85 | 18.42 | 18.43 |
| 947 | DNAJC17 | Non-matrisome | Non-matrisome | GO:CC not extracellular matrix | sp|Q9NVM6|DJC17_HUMAN | DnaJ homolog subfamily C member 17 | 17.03 | 16.43 | 16.59 |
| 948 | DNAJC2 | Non-matrisome | Non-matrisome | GO:CC not extracellular matrix | sp|Q99543|DNJC2_HUMAN | DnaJ homolog subfamily C member 2 | 18.05 | 17.43 | 17.49 |
| 949 | DNAJC25 | Non-matrisome | Non-matrisome | GO:CC not extracellular matrix | sp|Q9H1X3|DJC25_HUMAN | DnaJ homolog subfamily C member 25 | 17.18 | 17.16 | 17.17 |
| 950 | DNAJC30 | Non-matrisome | Non-matrisome | GO:CC not extracellular matrix | sp|Q96LL9|DJC30_HUMAN | DnaJ homolog subfamily C member 30, mitochondrial | 15.13 | 15.41 | 15.28 |
| 951 | DNAJC7 | Non-matrisome | Non-matrisome | GO:CC not extracellular matrix | sp|Q99615|DNJC7_HUMAN | DnaJ homolog subfamily C member 7 | 18.49 | 17.41 | 17.54 |
| 952 | DNAJC8 | Non-matrisome | Non-matrisome | GO:CC not extracellular matrix | sp|O75937|DNJC8_HUMAN | DnaJ homolog subfamily C member 8 | 20.36 | 19.95 | 20.12 |
| 953 | DNAJC9 | Non-matrisome | Non-matrisome | GO:CC not extracellular matrix | sp|Q8WXX5|DNJC9_HUMAN | DnaJ homolog subfamily C member 9 | 19.1 | 17.99 | 17.57 |
| 954 | DNASE2 | Non-matrisome | Non-matrisome | GO:CC not extracellular matrix | sp|O00115|DNS2A_HUMAN | Deoxyribonuclease-2-alpha | 16.6 | 18.25 | 18.45 |
| 955 | DNM1 | Non-matrisome | Non-matrisome | GO:CC not extracellular matrix | sp|Q05193|DYN1_HUMAN | Dynamin-1 | 17.03 | 17.9 | 18.21 |
| 956 | DNM1L | Non-matrisome | Non-matrisome | GO:CC not extracellular matrix | sp|O00429|DNM1L_HUMAN | Dynamin-1-like protein | 17.84 | 17.76 | 17.73 |
| 957 | DNM2 | Non-matrisome | Non-matrisome | GO:CC not extracellular matrix | sp|P50570|DYN2_HUMAN | Dynamin-2 | 17.8 | 18.31 | 18.39 |
| 958 | DNM3 | Non-matrisome | Non-matrisome | GO:CC not extracellular matrix | sp|Q9UQ16|DYN3_HUMAN | Dynamin-3 | 16.19 | 17.11 | 16.83 |
| 959 | DNPEP | Non-matrisome | Non-matrisome | GO:CC not extracellular matrix | sp|Q9ULA0|DNPEP_HUMAN | Aspartyl aminopeptidase | 17.5 | 18.16 | 18.05 |
| 960 | DNPH1 | Non-matrisome | Non-matrisome | GO:CC not extracellular matrix | sp|O43598|DNPH1_HUMAN | 2'-deoxynucleoside 5'-phosphate N-hydrolase 1 | 18.25 | 18.34 | 18.38 |
| 961 | DOCK1 | Non-matrisome | Non-matrisome | GO:CC not extracellular matrix | sp|Q14185|DOCK1_HUMAN | Dedicator of cytokinesis protein 1 | 17.19 | 17.38 | 17.62 |
| 962 | DOCK11 | Non-matrisome | Non-matrisome | GO:CC not extracellular matrix | sp|Q5JSL3|DOC11_HUMAN | Dedicator of cytokinesis protein 11 | 16.65 | 17.4 | 17.29 |
| 963 | DOCK2 | Non-matrisome | Non-matrisome | GO:CC extracellular matrix (GO:0005576, GO:0005578 & GO:0031012) & GO:CC not extracellular matrix | sp|Q92608|DOCK2_HUMAN | Dedicator of cytokinesis protein 2 | 19.41 | 19.79 | 19.66 |
| 964 | DOCK6 | Non-matrisome | Non-matrisome | GO:CC not extracellular matrix | sp|Q96HP0|DOCK6_HUMAN | Dedicator of cytokinesis protein 6 | 15.8 | 16.72 | 16.93 |
| 965 | DOCK9 | Non-matrisome | Non-matrisome | GO:CC not extracellular matrix | sp|Q9BZ29|DOCK9_HUMAN | Dedicator of cytokinesis protein 9 | 16.01 | 15.82 | 15.82 |
| 966 | DPH1 | Non-matrisome | Non-matrisome | GO:CC not extracellular matrix | sp|Q9BZG8|DPH1_HUMAN | 2-(3-amino-3-carboxypropyl)histidine synthase subunit 1 | 16.02 | 16.07 | 15.78 |
| 967 | DPM1 | Non-matrisome | Non-matrisome | GO:CC not extracellular matrix | sp|O60762|DPM1_HUMAN | Dolichol-phosphate mannosyltransferase subunit 1 | 17.79 | 18.04 | 17.8 |
| 968 | DPM3 | Non-matrisome | Non-matrisome | GO:CC not extracellular matrix | sp|Q9P2X0|DPM3_HUMAN | Dolichol-phosphate mannosyltransferase subunit 3 | 18.22 | 18.4 | 18.31 |
| 969 | DPP3 | Non-matrisome | Non-matrisome | GO:CC not extracellular matrix | sp|Q9NY33|DPP3_HUMAN | Dipeptidyl peptidase 3 | 19.19 | 19.46 | 19.4 |
| 970 | DPP4 | Non-matrisome | Non-matrisome | GO:CC extracellular matrix (GO:0005576, GO:0005578 & GO:0031012) & GO:CC not extracellular matrix | sp|P27487|DPP4_HUMAN | Dipeptidyl peptidase 4 | 15.2 | 17.92 | 18.16 |
| 971 | DPP7 | Non-matrisome | Non-matrisome | GO:CC extracellular matrix (GO:0005576, GO:0005578 & GO:0031012) & GO:CC not extracellular matrix | sp|Q9UHL4|DPP2_HUMAN | Dipeptidyl peptidase 2 | 17.59 | 19.56 | 20.03 |
| 972 | DPY30 | Non-matrisome | Non-matrisome | GO:CC not extracellular matrix | sp|Q9C005|DPY30_HUMAN | Protein dpy-30 homolog | 20.74 | 20.84 | 20.71 |
| 973 | DPYD | Non-matrisome | Non-matrisome | GO:CC not extracellular matrix | sp|Q12882|DPYD_HUMAN | Dihydropyrimidine dehydrogenase [NADP(+)] | 16.46 | 17.83 | 17.07 |
| 974 | DPYSL2 | Non-matrisome | Non-matrisome | GO:CC not extracellular matrix | sp|Q16555|DPYL2_HUMAN | Dihydropyrimidinase-related protein 2 | 18.66 | 19.38 | 19.54 |
| 975 | DPYSL3 | Non-matrisome | Non-matrisome | GO:CC not extracellular matrix | sp|Q14195|DPYL3_HUMAN | Dihydropyrimidinase-related protein 3 | 18.06 | 18.85 | 18.77 |
| 976 | DR1 | Non-matrisome | Non-matrisome | GO:CC not extracellular matrix | sp|Q01658|NC2B_HUMAN | Protein Dr1 | 19.09 | 18.56 | 18.62 |
| 977 | DRAP1 | Non-matrisome | Non-matrisome | GO:CC not extracellular matrix | sp|Q14919|NC2A_HUMAN | Dr1-associated corepressor | 16.75 | 16.24 | 16.28 |
| 978 | DRG1 | Non-matrisome | Non-matrisome | GO:CC not extracellular matrix | sp|Q9Y295|DRG1_HUMAN | Developmentally-regulated GTP-binding protein 1 | 18.66 | 17.92 | 17.92 |
| 979 | DRG2 | Non-matrisome | Non-matrisome | GO:CC not extracellular matrix | sp|P55039|DRG2_HUMAN | Developmentally-regulated GTP-binding protein 2 | 18.23 | 18.06 | 18.02 |
| 980 | DSP | Non-matrisome | Non-matrisome | GO:CC not extracellular matrix | sp|P15924|DESP_HUMAN | Desmoplakin | 18.06 | 17.03 | 17.11 |
| 981 | DST | Non-matrisome | Non-matrisome | GO:CC not extracellular matrix | sp|Q03001|DYST_HUMAN | Dystonin | 17.71 | 17.46 | 17.38 |
| 982 | DSTN | Non-matrisome | Non-matrisome | GO:CC not extracellular matrix | sp|P60981|DEST_HUMAN | Destrin | 19.24 | 19.2 | 19.22 |
| 983 | DTD1 | Non-matrisome | Non-matrisome | GO:CC not extracellular matrix | sp|Q8TEA8|DTD1_HUMAN | D-aminoacyl-tRNA deacylase 1 | 17.18 | 16.71 | 16.57 |
| 984 | DTNA | Non-matrisome | Non-matrisome | GO:CC not extracellular matrix | sp|Q9Y4J8|DTNA_HUMAN | Dystrobrevin alpha | 15.46 | 16.36 | 16.17 |
| 985 | DTX3L | Non-matrisome | Non-matrisome | GO:CC not extracellular matrix | sp|Q8TDB6|DTX3L_HUMAN | E3 ubiquitin-protein ligase DTX3L | 16.21 | 16.49 | 16.64 |
| 986 | DTYMK | Non-matrisome | Non-matrisome | GO:CC not extracellular matrix | sp|P23919|KTHY_HUMAN | Thymidylate kinase | 19.41 | 19.48 | 19.14 |
| 987 | DUSP14 | Non-matrisome | Non-matrisome | GO:CC not extracellular matrix | sp|O95147|DUS14_HUMAN | Dual specificity protein phosphatase 14 | 12.79 | 12.72 | 12.49 |
| 988 | DUSP23 | Non-matrisome | Non-matrisome | GO:CC not extracellular matrix | sp|Q9BVJ7|DUS23_HUMAN | Dual specificity protein phosphatase 23 | 18.39 | 18.33 | 18.54 |
| 989 | DUSP3 | Non-matrisome | Non-matrisome | GO:CC not extracellular matrix | sp|P51452|DUS3_HUMAN | Dual specificity protein phosphatase 3 | 17.48 | 17.61 | 17.66 |
| 990 | DUT | Non-matrisome | Non-matrisome | GO:CC not extracellular matrix | sp|P33316|DUT_HUMAN | Deoxyuridine 5'-triphosphate nucleotidohydrolase, mitochondrial | 20.36 | 18.64 | 18.78 |
| 991 | DYNC1H1 | Non-matrisome | Non-matrisome | GO:CC extracellular matrix (GO:0005576, GO:0005578 & GO:0031012) & GO:CC not extracellular matrix | sp|Q14204|DYHC1_HUMAN | Cytoplasmic dynein 1 heavy chain 1 | 18.91 | 19.08 | 18.88 |
| 992 | DYNC1I2 | Non-matrisome | Non-matrisome | GO:CC not extracellular matrix | sp|Q13409|DC1I2_HUMAN | Cytoplasmic dynein 1 intermediate chain 2 | 17.09 | 16.92 | 16.92 |
| 993 | DYNC1LI1 | Non-matrisome | Non-matrisome | GO:CC not extracellular matrix | sp|Q9Y6G9|DC1L1_HUMAN | Cytoplasmic dynein 1 light intermediate chain 1 | 19.05 | 19.08 | 18.97 |
| 994 | DYNC1LI2 | Non-matrisome | Non-matrisome | GO:CC not extracellular matrix | sp|O43237|DC1L2_HUMAN | Cytoplasmic dynein 1 light intermediate chain 2 | 18 | 17.87 | 17.9 |
| 995 | DYNLL1 | Non-matrisome | Non-matrisome | GO:CC not extracellular matrix | sp|P63167|DYL1_HUMAN | Dynein light chain 1, cytoplasmic | 18.47 | 18.14 | 18.19 |
| 996 | DYNLL2 | Non-matrisome | Non-matrisome | GO:CC not extracellular matrix | sp|Q96FJ2|DYL2_HUMAN | Dynein light chain 2, cytoplasmic | 19.35 | 19.16 | 19 |
| 997 | DYNLRB1 | Non-matrisome | Non-matrisome | GO:CC not extracellular matrix | sp|Q9NP97|DLRB1_HUMAN | Dynein light chain roadblock-type 1 | 17.7 | 17.4 | 17.98 |
| 998 | DYNLT1 | Non-matrisome | Non-matrisome | GO:CC extracellular matrix (GO:0005576, GO:0005578 & GO:0031012) & GO:CC not extracellular matrix | sp|P63172|DYLT1_HUMAN | Dynein light chain Tctex-type 1 | 18.5 | 18.03 | 17.55 |
| 999 | EBAG9 | Non-matrisome | Non-matrisome | GO:CC not extracellular matrix | sp|O00559|RCAS1_HUMAN | Receptor-binding cancer antigen expressed on SiSo cells | 15.1 | 14.96 | 15.41 |
| 1000 | EBNA1BP2 | Non-matrisome | Non-matrisome | GO:CC not extracellular matrix | sp|Q99848|EBP2_HUMAN | Probable rRNA-processing protein EBP2 | 19.49 | 19.21 | 19.1 |
| 1001 | EBP | Non-matrisome | Non-matrisome | GO:CC not extracellular matrix | sp|Q15125|EBP_HUMAN | 3-beta-hydroxysteroid-Delta(8),Delta(7)-isomerase | 19.71 | 18.96 | 18.77 |
| 1002 | ECH1 | Non-matrisome | Non-matrisome | GO:CC not extracellular matrix | sp|Q13011|ECH1_HUMAN | Delta(3,5)-Delta(2,4)-dienoyl-CoA isomerase, mitochondrial | 17.83 | 18.95 | 18.75 |
| 1003 | ECHDC1 | Non-matrisome | Non-matrisome | GO:CC not extracellular matrix | sp|Q9NTX5|ECHD1_HUMAN | Ethylmalonyl-CoA decarboxylase | 17.5 | 17.61 | 17.5 |
| 1004 | ECHDC3 | Non-matrisome | Non-matrisome | GO:CC not extracellular matrix | sp|Q96DC8|ECHD3_HUMAN | Enoyl-CoA hydratase domain-containing protein 3, mitochondrial | 14.84 | 16.27 | 16.23 |
| 1005 | ECHS1 | Non-matrisome | Non-matrisome | GO:CC not extracellular matrix | sp|P30084|ECHM_HUMAN | Enoyl-CoA hydratase, mitochondrial | 18.17 | 18.74 | 18.44 |
| 1006 | ECI1 | Non-matrisome | Non-matrisome | GO:CC not extracellular matrix | sp|P42126|ECI1_HUMAN | Enoyl-CoA delta isomerase 1, mitochondrial | 18.52 | 18.82 | 18.42 |
| 1007 | ECI2 | Non-matrisome | Non-matrisome | GO:CC not extracellular matrix | sp|O75521|ECI2_HUMAN | Enoyl-CoA delta isomerase 2 | 17.23 | 17.82 | 17.57 |
| 1008 | ECM1 | Core matrisome | ECM Glycoproteins | GO:CC extracellular matrix (GO:0005576, GO:0005578 & GO:0031012) & GO:CC not extracellular matrix | sp|Q16610|ECM1_HUMAN | Extracellular matrix protein 1 | 17.08 | 17.16 | 17.34 |
| 1009 | ECPAS | Non-matrisome | Non-matrisome | GO:CC not extracellular matrix | sp|Q5VYK3|ECM29_HUMAN | Proteasome adapter and scaffold protein ECM29 | 18.28 | 18.18 | 17.98 |
| 1010 | EDC4 | Non-matrisome | Non-matrisome | GO:CC not extracellular matrix | sp|Q6P2E9|EDC4_HUMAN | Enhancer of mRNA-decapping protein 4 | 17.93 | 17.98 | 17.81 |
| 1011 | EDF1 | Non-matrisome | Non-matrisome | GO:CC not extracellular matrix | sp|O60869|EDF1_HUMAN | Endothelial differentiation-related factor 1 | 20.34 | 19.05 | 19.44 |
| 1012 | EEA1 | Non-matrisome | Non-matrisome | GO:CC not extracellular matrix | sp|Q15075|EEA1_HUMAN | Early endosome antigen 1 | 18.34 | 18.99 | 18.83 |
| 1013 | EEF1A1 | Non-matrisome | Non-matrisome | GO:CC extracellular matrix (GO:0005576, GO:0005578 & GO:0031012) & GO:CC not extracellular matrix | sp|P68104|EF1A1_HUMAN | Elongation factor 1-alpha 1 | 18.37 | 16.99 | 17.35 |
| 1014 | EEF1A2 | Non-matrisome | Non-matrisome | GO:CC not extracellular matrix | sp|Q05639|EF1A2_HUMAN | Elongation factor 1-alpha 2 | 16.08 | 15.59 | 15.6 |
| 1015 | EEF1B2 | Non-matrisome | Non-matrisome | GO:CC not extracellular matrix | sp|P24534|EF1B_HUMAN | Elongation factor 1-beta | 18.76 | 18.45 | 18.32 |
| 1016 | EEF1D | Non-matrisome | Non-matrisome | GO:CC not extracellular matrix | sp|P29692|EF1D_HUMAN | Elongation factor 1-delta | 18.14 | 18.02 | 17.97 |
| 1017 | EEF1E1 | Non-matrisome | Non-matrisome | GO:CC not extracellular matrix | sp|O43324|MCA3_HUMAN | Eukaryotic translation elongation factor 1 epsilon-1 | 18.91 | 18.93 | 18.8 |
| 1018 | EEF1G | Non-matrisome | Non-matrisome | GO:CC not extracellular matrix | sp|P26641|EF1G_HUMAN | Elongation factor 1-gamma | 19.52 | 19.2 | 19.06 |
| 1019 | EEF2 | Non-matrisome | Non-matrisome | GO:CC extracellular matrix (GO:0005576, GO:0005578 & GO:0031012) & GO:CC not extracellular matrix | sp|P13639|EF2_HUMAN | Elongation factor 2 | 19.8 | 18.83 | 18.76 |
| 1020 | EEF2K | Non-matrisome | Non-matrisome | GO:CC not extracellular matrix | sp|O00418|EF2K_HUMAN | Eukaryotic elongation factor 2 kinase | 17.64 | 16.98 | 17.45 |
| 1021 | EEFSEC | Non-matrisome | Non-matrisome | GO:CC not extracellular matrix | sp|P57772|SELB_HUMAN | Selenocysteine-specific elongation factor | 18 | 18.31 | 18.52 |
| 1022 | EFCAB7 | Non-matrisome | Non-matrisome | GO:CC not extracellular matrix | sp|A8K855|EFCB7_HUMAN | EF-hand calcium-binding domain-containing protein 7 | 14.64 | 15.12 | 15.38 |
| 1023 | EFEMP1 | Core matrisome | ECM Glycoproteins | GO:CC extracellular matrix (GO:0005576, GO:0005578 & GO:0031012) & GO:CC not extracellular matrix | sp|Q12805|FBLN3_HUMAN | EGF-containing fibulin-like extracellular matrix protein 1 | 16.21 | 17.13 | 16.75 |
| 1024 | EFEMP2 | Core matrisome | ECM Glycoproteins | GO:CC extracellular matrix (GO:0005576, GO:0005578 & GO:0031012) & GO:CC not extracellular matrix | sp|O95967|FBLN4_HUMAN | EGF-containing fibulin-like extracellular matrix protein 2 | 14.66 | 17.83 | 17.4 |
| 1025 | EFHD1 | Non-matrisome | Non-matrisome | GO:CC not extracellular matrix | sp|Q9BUP0|EFHD1_HUMAN | EF-hand domain-containing protein D1 | 17.53 | 16.5 | 16.13 |
| 1026 | EFHD2 | Non-matrisome | Non-matrisome | GO:CC not extracellular matrix | sp|Q96C19|EFHD2_HUMAN | EF-hand domain-containing protein D2 | 19.48 | 18.59 | 18.41 |
| 1027 | EFL1 | Non-matrisome | Non-matrisome | GO:CC not extracellular matrix | sp|Q7Z2Z2|EFL1_HUMAN | Elongation factor-like GTPase 1 | 17.96 | 18.33 | 18.2 |
| 1028 | EFR3A | Non-matrisome | Non-matrisome | GO:CC not extracellular matrix | sp|Q14156|EFR3A_HUMAN | Protein EFR3 homolog A | 16.06 | 16.42 | 16.43 |
| 1029 | EFTUD2 | Non-matrisome | Non-matrisome | GO:CC not extracellular matrix | sp|Q15029|U5S1_HUMAN | 116 kDa U5 small nuclear ribonucleoprotein component | 18.83 | 18.89 | 18.78 |
| 1030 | EGFR | Non-matrisome | Non-matrisome | GO:CC not extracellular matrix | sp|P00533|EGFR_HUMAN | Epidermal growth factor receptor | 17.91 | 17.37 | 16.97 |
| 1031 | EGLN1 | Matrisome-associated | ECM Regulators | GO:CC not extracellular matrix | sp|Q9GZT9|EGLN1_HUMAN | Egl nine homolog 1 | 18.26 | 18.63 | 18.18 |
| 1032 | EHBP1 | Non-matrisome | Non-matrisome | GO:CC not extracellular matrix | sp|Q8NDI1|EHBP1_HUMAN | EH domain-binding protein 1 | 17.74 | 17.21 | 17.08 |
| 1033 | EHBP1L1 | Non-matrisome | Non-matrisome | GO:CC not extracellular matrix | sp|Q8N3D4|EH1L1_HUMAN | EH domain-binding protein 1-like protein 1 | 16.55 | 16.44 | 16.15 |
| 1034 | EHD1 | Non-matrisome | Non-matrisome | GO:CC not extracellular matrix | sp|Q9H4M9|EHD1_HUMAN | EH domain-containing protein 1 | 19.02 | 19.48 | 19.46 |
| 1035 | EHD2 | Non-matrisome | Non-matrisome | GO:CC not extracellular matrix | sp|Q9NZN4|EHD2_HUMAN | EH domain-containing protein 2 | 18.34 | 18.44 | 18.16 |
| 1036 | EHD3 | Non-matrisome | Non-matrisome | GO:CC not extracellular matrix | sp|Q9NZN3|EHD3_HUMAN | EH domain-containing protein 3 | 18.44 | 19.27 | 19.3 |
| 1037 | EHD4 | Non-matrisome | Non-matrisome | GO:CC not extracellular matrix | sp|Q9H223|EHD4_HUMAN | EH domain-containing protein 4 | 18.63 | 18.68 | 18.54 |
| 1038 | EHHADH | Non-matrisome | Non-matrisome | GO:CC not extracellular matrix | sp|Q08426|ECHP_HUMAN | Peroxisomal bifunctional enzyme | 17.73 | 17.83 | 17.69 |
| 1039 | EIF1 | Non-matrisome | Non-matrisome | GO:CC not extracellular matrix | sp|P41567|EIF1_HUMAN | Eukaryotic translation initiation factor 1 | 18.45 | 16.92 | 17.52 |
| 1040 | EIF1AX | Non-matrisome | Non-matrisome | GO:CC not extracellular matrix | sp|P47813|IF1AX_HUMAN | Eukaryotic translation initiation factor 1A, X-chromosomal | 20.04 | 20.08 | 19.79 |
| 1041 | EIF2A | Non-matrisome | Non-matrisome | GO:CC not extracellular matrix | sp|Q9BY44|EIF2A_HUMAN | Eukaryotic translation initiation factor 2A | 18.92 | 18.09 | 18.21 |
| 1042 | EIF2AK2 | Non-matrisome | Non-matrisome | GO:CC not extracellular matrix | sp|P19525|E2AK2_HUMAN | Interferon-induced, double-stranded RNA-activated protein kinase | 19.46 | 19.2 | 19.33 |
| 1043 | EIF2B1 | Non-matrisome | Non-matrisome | GO:CC not extracellular matrix | sp|Q14232|EI2BA_HUMAN | Translation initiation factor eIF-2B subunit alpha | 18.06 | 17.79 | 17.71 |
| 1044 | EIF2B2 | Non-matrisome | Non-matrisome | GO:CC not extracellular matrix | sp|P49770|EI2BB_HUMAN | Translation initiation factor eIF-2B subunit beta | 18.67 | 18.48 | 18.51 |
| 1045 | EIF2B3 | Non-matrisome | Non-matrisome | GO:CC not extracellular matrix | sp|Q9NR50|EI2BG_HUMAN | Translation initiation factor eIF-2B subunit gamma | 18.15 | 17.73 | 17.72 |
| 1046 | EIF2B4 | Non-matrisome | Non-matrisome | GO:CC not extracellular matrix | sp|Q9UI10|EI2BD_HUMAN | Translation initiation factor eIF-2B subunit delta | 17.42 | 17.26 | 17.23 |
| 1047 | EIF2B5 | Non-matrisome | Non-matrisome | GO:CC not extracellular matrix | sp|Q13144|EI2BE_HUMAN | Translation initiation factor eIF-2B subunit epsilon | 17.52 | 17.45 | 17.44 |
| 1048 | EIF2S1 | Non-matrisome | Non-matrisome | GO:CC not extracellular matrix | sp|P05198|IF2A_HUMAN | Eukaryotic translation initiation factor 2 subunit 1 | 19.46 | 18.96 | 18.84 |
| 1049 | EIF2S2 | Non-matrisome | Non-matrisome | GO:CC not extracellular matrix | sp|P20042|IF2B_HUMAN | Eukaryotic translation initiation factor 2 subunit 2 | 17.98 | 17.54 | 17.57 |
| 1050 | EIF2S3 | Non-matrisome | Non-matrisome | GO:CC not extracellular matrix | sp|P41091|IF2G_HUMAN | Eukaryotic translation initiation factor 2 subunit 3 | 20.55 | 20.2 | 19.99 |
| 1051 | EIF3A | Non-matrisome | Non-matrisome | GO:CC not extracellular matrix | sp|Q14152|EIF3A_HUMAN | Eukaryotic translation initiation factor 3 subunit A | 19.15 | 18.52 | 18.51 |
| 1052 | EIF3B | Non-matrisome | Non-matrisome | GO:CC not extracellular matrix | sp|P55884|EIF3B_HUMAN | Eukaryotic translation initiation factor 3 subunit B | 17.61 | 17.13 | 17.06 |
| 1053 | EIF3C | Non-matrisome | Non-matrisome | GO:CC not extracellular matrix | sp|Q99613|EIF3C_HUMAN | Eukaryotic translation initiation factor 3 subunit C | 15.77 | 14.92 | 14.83 |
| 1054 | EIF3D | Non-matrisome | Non-matrisome | GO:CC not extracellular matrix | sp|O15371|EIF3D_HUMAN | Eukaryotic translation initiation factor 3 subunit D | 18.21 | 17.59 | 17.58 |
| 1055 | EIF3E | Non-matrisome | Non-matrisome | GO:CC not extracellular matrix | sp|P60228|EIF3E_HUMAN | Eukaryotic translation initiation factor 3 subunit E | 17.99 | 17.39 | 17.38 |
| 1056 | EIF3F | Non-matrisome | Non-matrisome | GO:CC not extracellular matrix | sp|O00303|EIF3F_HUMAN | Eukaryotic translation initiation factor 3 subunit F | 17.81 | 17.28 | 17.27 |
| 1057 | EIF3G | Non-matrisome | Non-matrisome | GO:CC not extracellular matrix | sp|O75821|EIF3G_HUMAN | Eukaryotic translation initiation factor 3 subunit G | 18.15 | 17.32 | 17.44 |
| 1058 | EIF3H | Non-matrisome | Non-matrisome | GO:CC not extracellular matrix | sp|O15372|EIF3H_HUMAN | Eukaryotic translation initiation factor 3 subunit H | 18.96 | 18.39 | 18.31 |
| 1059 | EIF3I | Non-matrisome | Non-matrisome | GO:CC not extracellular matrix | sp|Q13347|EIF3I_HUMAN | Eukaryotic translation initiation factor 3 subunit I | 18.61 | 18.09 | 18.04 |
| 1060 | EIF3J | Non-matrisome | Non-matrisome | GO:CC not extracellular matrix | sp|O75822|EIF3J_HUMAN | Eukaryotic translation initiation factor 3 subunit J | 17.87 | 17.08 | 17.03 |
| 1061 | EIF3K | Non-matrisome | Non-matrisome | GO:CC not extracellular matrix | sp|Q9UBQ5|EIF3K_HUMAN | Eukaryotic translation initiation factor 3 subunit K | 17.82 | 17.15 | 17.06 |
| 1062 | EIF3L | Non-matrisome | Non-matrisome | GO:CC not extracellular matrix | sp|Q9Y262|EIF3L_HUMAN | Eukaryotic translation initiation factor 3 subunit L | 20.58 | 20 | 19.95 |
| 1063 | EIF3M | Non-matrisome | Non-matrisome | GO:CC not extracellular matrix | sp|Q7L2H7|EIF3M_HUMAN | Eukaryotic translation initiation factor 3 subunit M | 17.61 | 17.15 | 17.16 |
| 1064 | EIF4A1 | Non-matrisome | Non-matrisome | GO:CC not extracellular matrix | sp|P60842|IF4A1_HUMAN | Eukaryotic initiation factor 4A-I | 21.4 | 20.27 | 20.6 |
| 1065 | EIF4A2 | Non-matrisome | Non-matrisome | GO:CC not extracellular matrix | sp|Q14240|IF4A2_HUMAN | Eukaryotic initiation factor 4A-II | 17.37 | 17.49 | 17.98 |
| 1066 | EIF4A3 | Non-matrisome | Non-matrisome | GO:CC not extracellular matrix | sp|P38919|IF4A3_HUMAN | Eukaryotic initiation factor 4A-III | 18.94 | 18.87 | 18.78 |
| 1067 | EIF4B | Non-matrisome | Non-matrisome | GO:CC not extracellular matrix | sp|P23588|IF4B_HUMAN | Eukaryotic translation initiation factor 4B | 19.07 | 18.17 | 18.2 |
| 1068 | EIF4E | Non-matrisome | Non-matrisome | GO:CC not extracellular matrix | sp|P06730|IF4E_HUMAN | Eukaryotic translation initiation factor 4E | 17.38 | 17.26 | 17.4 |
| 1069 | EIF4G1 | Non-matrisome | Non-matrisome | GO:CC not extracellular matrix | sp|Q04637|IF4G1_HUMAN | Eukaryotic translation initiation factor 4 gamma 1 | 19.09 | 18.06 | 18.12 |
| 1070 | EIF4G2 | Non-matrisome | Non-matrisome | GO:CC not extracellular matrix | sp|P78344|IF4G2_HUMAN | Eukaryotic translation initiation factor 4 gamma 2 | 18.48 | 17.95 | 17.82 |
| 1071 | EIF4G3 | Non-matrisome | Non-matrisome | GO:CC not extracellular matrix | sp|O43432|IF4G3_HUMAN | Eukaryotic translation initiation factor 4 gamma 3 | 17.75 | 17.91 | 18.19 |
| 1072 | EIF4H | Non-matrisome | Non-matrisome | GO:CC not extracellular matrix | sp|Q15056|IF4H_HUMAN | Eukaryotic translation initiation factor 4H | 19.43 | 18.69 | 18.92 |
| 1073 | EIF5 | Non-matrisome | Non-matrisome | GO:CC not extracellular matrix | sp|P55010|IF5_HUMAN | Eukaryotic translation initiation factor 5 | 19.11 | 18.72 | 18.51 |
| 1074 | EIF5A | Non-matrisome | Non-matrisome | GO:CC not extracellular matrix | sp|P63241|IF5A1_HUMAN | Eukaryotic translation initiation factor 5A-1 | 16.34 | 15.91 | 15.74 |
| 1075 | EIF5B | Non-matrisome | Non-matrisome | GO:CC not extracellular matrix | sp|O60841|IF2P_HUMAN | Eukaryotic translation initiation factor 5B | 18.75 | 18.15 | 18.09 |
| 1076 | EIF6 | Non-matrisome | Non-matrisome | GO:CC not extracellular matrix | sp|P56537|IF6_HUMAN | Eukaryotic translation initiation factor 6 | 19.16 | 18.46 | 18.49 |
| 1077 | ELAC2 | Non-matrisome | Non-matrisome | GO:CC not extracellular matrix | sp|Q9BQ52|RNZ2_HUMAN | Zinc phosphodiesterase ELAC protein 2 | 16.96 | 16.02 | 16.25 |
| 1078 | ELAVL1 | Non-matrisome | Non-matrisome | GO:CC not extracellular matrix | sp|Q15717|ELAV1_HUMAN | ELAV-like protein 1 | 18.29 | 18.11 | 18.23 |
| 1079 | ELMO2 | Non-matrisome | Non-matrisome | GO:CC not extracellular matrix | sp|Q96JJ3|ELMO2_HUMAN | Engulfment and cell motility protein 2 | 17.55 | 18.03 | 18.05 |
| 1080 | ELMOD2 | Non-matrisome | Non-matrisome | GO:CC not extracellular matrix | sp|Q8IZ81|ELMD2_HUMAN | ELMO domain-containing protein 2 | 17.79 | 18 | 17.94 |
| 1081 | ELOB | Non-matrisome | Non-matrisome | GO:CC not extracellular matrix | sp|Q15370|ELOB_HUMAN | Elongin-B | 19.1 | 18.61 | 18.63 |
| 1082 | ELOC | Non-matrisome | Non-matrisome | GO:CC not extracellular matrix | sp|Q15369|ELOC_HUMAN | Elongin-C | 18.15 | 17.92 | 17.96 |
| 1083 | ELOVL1 | Non-matrisome | Non-matrisome | GO:CC not extracellular matrix | sp|Q9BW60|ELOV1_HUMAN | Elongation of very long chain fatty acids protein 1 | 16.33 | 15.4 | 15.52 |
| 1084 | ELP1 | Non-matrisome | Non-matrisome | GO:CC not extracellular matrix | sp|O95163|ELP1_HUMAN | Elongator complex protein 1 | 17.37 | 17.03 | 16.92 |
| 1085 | ELP3 | Non-matrisome | Non-matrisome | GO:CC not extracellular matrix | sp|Q9H9T3|ELP3_HUMAN | Elongator complex protein 3 | 17.59 | 17.45 | 17.16 |
| 1086 | EMC1 | Non-matrisome | Non-matrisome | GO:CC extracellular matrix (GO:0005576, GO:0005578 & GO:0031012) & GO:CC not extracellular matrix | sp|Q8N766|EMC1_HUMAN | ER membrane protein complex subunit 1 | 18.61 | 18.39 | 18.33 |
| 1087 | EMC2 | Non-matrisome | Non-matrisome | GO:CC not extracellular matrix | sp|Q15006|EMC2_HUMAN | ER membrane protein complex subunit 2 | 17.76 | 17.81 | 17.75 |
| 1088 | EMC3 | Non-matrisome | Non-matrisome | GO:CC extracellular matrix (GO:0005576, GO:0005578 & GO:0031012) & GO:CC not extracellular matrix | sp|Q9P0I2|EMC3_HUMAN | ER membrane protein complex subunit 3 | 16.91 | 16.96 | 16.87 |
| 1089 | EMC7 | Non-matrisome | Non-matrisome | GO:CC extracellular matrix (GO:0005576, GO:0005578 & GO:0031012) & GO:CC not extracellular matrix | sp|Q9NPA0|EMC7_HUMAN | ER membrane protein complex subunit 7 | 19.29 | 19.09 | 19.18 |
| 1090 | EMD | Non-matrisome | Non-matrisome | GO:CC not extracellular matrix | sp|P50402|EMD_HUMAN | Emerin | 17.42 | 17.91 | 18.03 |
| 1091 | EMG1 | Non-matrisome | Non-matrisome | GO:CC not extracellular matrix | sp|Q92979|NEP1_HUMAN | Ribosomal RNA small subunit methyltransferase NEP1 | 17.49 | 16.56 | 16.46 |
| 1092 | EMILIN1 | Core matrisome | ECM Glycoproteins | GO:CC extracellular matrix (GO:0005576, GO:0005578 & GO:0031012) & GO:CC not extracellular matrix | sp|Q9Y6C2|EMIL1_HUMAN | EMILIN-1 | 14.32 | 18.38 | 18.83 |
| 1093 | EMILIN2 | Core matrisome | ECM Glycoproteins | GO:CC extracellular matrix (GO:0005576, GO:0005578 & GO:0031012) & GO:CC not extracellular matrix | sp|Q9BXX0|EMIL2_HUMAN | EMILIN-2 | 15.46 | 18.33 | 18.37 |
| 1094 | EML2 | Non-matrisome | Non-matrisome | GO:CC not extracellular matrix | sp|O95834|EMAL2_HUMAN | Echinoderm microtubule-associated protein-like 2 | 18.35 | 18.99 | 19.14 |
| 1095 | EML3 | Non-matrisome | Non-matrisome | GO:CC not extracellular matrix | sp|Q32P44|EMAL3_HUMAN | Echinoderm microtubule-associated protein-like 3 | 17.26 | 17.96 | 18.02 |
| 1096 | EML4 | Non-matrisome | Non-matrisome | GO:CC not extracellular matrix | sp|Q9HC35|EMAL4_HUMAN | Echinoderm microtubule-associated protein-like 4 | 18 | 18.43 | 18.11 |
| 1097 | ENDOD1 | Non-matrisome | Non-matrisome | GO:CC extracellular matrix (GO:0005576, GO:0005578 & GO:0031012) & GO:CC not extracellular matrix | sp|O94919|ENDD1_HUMAN | Endonuclease domain-containing 1 protein | 17.2 | 17.54 | 17.32 |
| 1098 | ENG | Non-matrisome | Non-matrisome | GO:CC not extracellular matrix | sp|P17813|EGLN_HUMAN | Endoglin | 17.36 | 17.94 | 18.05 |
| 1099 | ENO1 | Non-matrisome | Non-matrisome | GO:CC not extracellular matrix | sp|P06733|ENOA_HUMAN | Alpha-enolase | 18.7 | 18.97 | 18.63 |
| 1100 | ENO2 | Non-matrisome | Non-matrisome | GO:CC not extracellular matrix | sp|P09104|ENOG_HUMAN | Gamma-enolase | 16.34 | 18.14 | 17.34 |
| 1101 | ENO3 | Non-matrisome | Non-matrisome | GO:CC not extracellular matrix | sp|P13929|ENOB_HUMAN | Beta-enolase | 18.38 | 19.28 | 19.41 |
| 1102 | ENOPH1 | Non-matrisome | Non-matrisome | GO:CC not extracellular matrix | sp|Q9UHY7|ENOPH_HUMAN | Enolase-phosphatase E1 | 17.25 | 17.12 | 17.33 |
| 1103 | ENSA | Non-matrisome | Non-matrisome | GO:CC not extracellular matrix | sp|O43768|ENSA_HUMAN | Alpha-endosulfine | 17.99 | 17.44 | 17.72 |
| 1104 | ENY2 | Non-matrisome | Non-matrisome | GO:CC not extracellular matrix | sp|Q9NPA8|ENY2_HUMAN | Transcription and mRNA export factor ENY2 | 19.29 | 19.03 | 19.37 |
| 1105 | EOGT | Non-matrisome | Non-matrisome | GO:CC not extracellular matrix | sp|Q5NDL2|EOGT_HUMAN | EGF domain-specific O-linked N-acetylglucosamine transferase | 17.31 | 17.29 | 17.18 |
| 1106 | EPB41 | Non-matrisome | Non-matrisome | GO:CC not extracellular matrix | sp|P11171|EPB41_HUMAN | Protein 4.1 | 15.64 | 16.69 | 16.67 |
| 1107 | EPB41L1 | Non-matrisome | Non-matrisome | GO:CC not extracellular matrix | sp|Q9H4G0|E41L1_HUMAN | Band 4.1-like protein 1 | 14.85 | 16.15 | 16.49 |
| 1108 | EPB41L2 | Non-matrisome | Non-matrisome | GO:CC not extracellular matrix | sp|O43491|E41L2_HUMAN | Band 4.1-like protein 2 | 17.66 | 17.74 | 17.64 |
| 1109 | EPB41L3 | Non-matrisome | Non-matrisome | GO:CC not extracellular matrix | sp|Q9Y2J2|E41L3_HUMAN | Band 4.1-like protein 3 | 16.19 | 16.2 | 16.28 |
| 1110 | EPDR1 | Non-matrisome | Non-matrisome | GO:CC extracellular matrix (GO:0005576, GO:0005578 & GO:0031012) & GO:CC not extracellular matrix | sp|Q9UM22|EPDR1_HUMAN | Mammalian ependymin-related protein 1 | 15.51 | 16.57 | 16.9 |
| 1111 | EPHX1 | Non-matrisome | Non-matrisome | GO:CC not extracellular matrix | sp|P07099|HYEP_HUMAN | Epoxide hydrolase 1 | 17.2 | 17.96 | 18.51 |
| 1112 | EPHX2 | Non-matrisome | Non-matrisome | GO:CC not extracellular matrix | sp|P34913|HYES_HUMAN | Bifunctional epoxide hydrolase 2 | 17.28 | 17.08 | 16.98 |
| 1113 | EPM2AIP1 | Non-matrisome | Non-matrisome | GO:CC not extracellular matrix | sp|Q7L775|EPMIP_HUMAN | EPM2A-interacting protein 1 | 17.21 | 17.73 | 17.82 |
| 1114 | EPN1 | Non-matrisome | Non-matrisome | GO:CC not extracellular matrix | sp|Q9Y6I3|EPN1_HUMAN | Epsin-1 | 17.37 | 17.18 | 17.25 |
| 1115 | EPPK1 | Non-matrisome | Non-matrisome | GO:CC not extracellular matrix | sp|P58107|EPIPL_HUMAN | Epiplakin | 14.81 | 15.08 | 15.01 |
| 1116 | EPRS1 | Non-matrisome | Non-matrisome | GO:CC not extracellular matrix | sp|P07814|SYEP_HUMAN | Bifunctional glutamate/proline--tRNA ligase | 18.58 | 18.18 | 17.98 |
| 1117 | EPS15 | Non-matrisome | Non-matrisome | GO:CC not extracellular matrix | sp|P42566|EPS15_HUMAN | Epidermal growth factor receptor substrate 15 | 18.09 | 18.12 | 18.2 |
| 1118 | EPS15L1 | Non-matrisome | Non-matrisome | GO:CC not extracellular matrix | sp|Q9UBC2|EP15R_HUMAN | Epidermal growth factor receptor substrate 15-like 1 | 17.21 | 17.32 | 17.35 |
| 1119 | EPS8L2 | Non-matrisome | Non-matrisome | GO:CC not extracellular matrix | sp|Q9H6S3|ES8L2_HUMAN | Epidermal growth factor receptor kinase substrate 8-like protein 2 | 16.86 | 17.25 | 17.06 |
| 1120 | ERAP1 | Non-matrisome | Non-matrisome | GO:CC extracellular matrix (GO:0005576, GO:0005578 & GO:0031012) & GO:CC not extracellular matrix | sp|Q9NZ08|ERAP1_HUMAN | Endoplasmic reticulum aminopeptidase 1 | 17.15 | 17.59 | 17.52 |
| 1121 | ERAP2 | Non-matrisome | Non-matrisome | GO:CC not extracellular matrix | sp|Q6P179|ERAP2_HUMAN | Endoplasmic reticulum aminopeptidase 2 | 18.63 | 18.35 | 18.43 |
| 1122 | ERBB2 | Non-matrisome | Non-matrisome | GO:CC not extracellular matrix | sp|P04626|ERBB2_HUMAN | Receptor tyrosine-protein kinase erbB-2 | 17.22 | 16.81 | 16.78 |
| 1123 | ERBIN | Non-matrisome | Non-matrisome | GO:CC not extracellular matrix | sp|Q96RT1|ERBIN_HUMAN | Erbin | 17.43 | 16.77 | 16.79 |
| 1124 | ERCC2 | Non-matrisome | Non-matrisome | GO:CC not extracellular matrix | sp|P18074|ERCC2_HUMAN | General transcription and DNA repair factor IIH helicase subunit XPD | 17.56 | 17.49 | 17.55 |
| 1125 | ERGIC1 | Non-matrisome | Non-matrisome | GO:CC not extracellular matrix | sp|Q969X5|ERGI1_HUMAN | Endoplasmic reticulum-Golgi intermediate compartment protein 1 | 17.79 | 18.33 | 18.12 |
| 1126 | ERH | Non-matrisome | Non-matrisome | GO:CC not extracellular matrix | sp|P84090|ERH_HUMAN | Enhancer of rudimentary homolog | 17.94 | 17.94 | 17.93 |
| 1127 | ERI3 | Non-matrisome | Non-matrisome | GO:CC not extracellular matrix | sp|O43414|ERI3_HUMAN | ERI1 exoribonuclease 3 | 18.61 | 18.8 | 18.75 |
| 1128 | ERLEC1 | Non-matrisome | Non-matrisome | GO:CC not extracellular matrix | sp|Q96DZ1|ERLEC_HUMAN | Endoplasmic reticulum lectin 1 | 17.08 | 17.12 | 17.3 |
| 1129 | ERLIN1 | Non-matrisome | Non-matrisome | GO:CC not extracellular matrix | sp|O75477|ERLN1_HUMAN | Erlin-1 | 19.33 | 19.52 | 19.25 |
| 1130 | ERLIN2 | Non-matrisome | Non-matrisome | GO:CC not extracellular matrix | sp|O94905|ERLN2_HUMAN | Erlin-2 | 19.21 | 19.52 | 19.3 |
| 1131 | ERMP1 | Non-matrisome | Non-matrisome | GO:CC not extracellular matrix | sp|Q7Z2K6|ERMP1_HUMAN | Endoplasmic reticulum metallopeptidase 1 | 16.46 | 18.29 | 19.09 |
| 1132 | ERN1 | Non-matrisome | Non-matrisome | GO:CC not extracellular matrix | sp|O75460|ERN1_HUMAN | Serine/threonine-protein kinase/endoribonuclease IRE1 | 15.88 | 16.21 | 16.01 |
| 1133 | ERO1A | Non-matrisome | Non-matrisome | GO:CC not extracellular matrix | sp|Q96HE7|ERO1A_HUMAN | ERO1-like protein alpha | 17.82 | 19.23 | 17.88 |
| 1134 | ERP29 | Non-matrisome | Non-matrisome | GO:CC not extracellular matrix | sp|P30040|ERP29_HUMAN | Endoplasmic reticulum resident protein 29 | 19.04 | 18.96 | 18.81 |
| 1135 | ERP44 | Non-matrisome | Non-matrisome | GO:CC extracellular matrix (GO:0005576, GO:0005578 & GO:0031012) & GO:CC not extracellular matrix | sp|Q9BS26|ERP44_HUMAN | Endoplasmic reticulum resident protein 44 | 19.51 | 19.64 | 19.71 |
| 1136 | ESD | Non-matrisome | Non-matrisome | GO:CC not extracellular matrix | sp|P10768|ESTD_HUMAN | S-formylglutathione hydrolase | 19.29 | 19.51 | 19.37 |
| 1137 | ESRRA | Non-matrisome | Non-matrisome | GO:CC not extracellular matrix | sp|P11474|ERR1_HUMAN | Steroid hormone receptor ERR1 | 14.59 | 14.29 | 14.27 |
| 1138 | ESYT1 | Non-matrisome | Non-matrisome | GO:CC not extracellular matrix | sp|Q9BSJ8|ESYT1_HUMAN | Extended synaptotagmin-1 | 18.26 | 18.25 | 18.01 |
| 1139 | ESYT2 | Non-matrisome | Non-matrisome | GO:CC not extracellular matrix | sp|A0FGR8|ESYT2_HUMAN | Extended synaptotagmin-2 | 18.66 | 19.16 | 18.91 |
| 1140 | ETF1 | Non-matrisome | Non-matrisome | GO:CC not extracellular matrix | sp|P62495|ERF1_HUMAN | Eukaryotic peptide chain release factor subunit 1 | 19.68 | 19.02 | 19.07 |
| 1141 | ETFA | Non-matrisome | Non-matrisome | GO:CC not extracellular matrix | sp|P13804|ETFA_HUMAN | Electron transfer flavoprotein subunit alpha, mitochondrial | 19.89 | 20.48 | 20.28 |
| 1142 | ETFB | Non-matrisome | Non-matrisome | GO:CC not extracellular matrix | sp|P38117|ETFB_HUMAN | Electron transfer flavoprotein subunit beta | 19.42 | 19.96 | 19.73 |
| 1143 | ETFDH | Non-matrisome | Non-matrisome | GO:CC not extracellular matrix | sp|Q16134|ETFD_HUMAN | Electron transfer flavoprotein-ubiquinone oxidoreductase, mitochondrial | 17.18 | 18.46 | 18.23 |
| 1144 | ETHE1 | Non-matrisome | Non-matrisome | GO:CC not extracellular matrix | sp|O95571|ETHE1_HUMAN | Persulfide dioxygenase ETHE1, mitochondrial | 18.06 | 18.57 | 18.5 |
| 1145 | EVI2B | Non-matrisome | Non-matrisome | GO:CC not extracellular matrix | sp|P34910|EVI2B_HUMAN | Protein EVI2B | 15.57 | 15.57 | 15.79 |
| 1146 | EWSR1 | Non-matrisome | Non-matrisome | GO:CC not extracellular matrix | sp|Q01844|EWS_HUMAN | RNA-binding protein EWS | 19.06 | 19.09 | 19.19 |
| 1147 | EXOC1 | Non-matrisome | Non-matrisome | GO:CC not extracellular matrix | sp|Q9NV70|EXOC1_HUMAN | Exocyst complex component 1 | 18.18 | 18.33 | 18.29 |
| 1148 | EXOC2 | Non-matrisome | Non-matrisome | GO:CC not extracellular matrix | sp|Q96KP1|EXOC2_HUMAN | Exocyst complex component 2 | 17.81 | 18.15 | 18.14 |
| 1149 | EXOC3 | Non-matrisome | Non-matrisome | GO:CC not extracellular matrix | sp|O60645|EXOC3_HUMAN | Exocyst complex component 3 | 18.2 | 18.49 | 18.52 |
| 1150 | EXOC4 | Non-matrisome | Non-matrisome | GO:CC not extracellular matrix | sp|Q96A65|EXOC4_HUMAN | Exocyst complex component 4 | 17.7 | 17.97 | 17.96 |
| 1151 | EXOC5 | Non-matrisome | Non-matrisome | GO:CC not extracellular matrix | sp|O00471|EXOC5_HUMAN | Exocyst complex component 5 | 18.43 | 18.71 | 18.69 |
| 1152 | EXOC6B | Non-matrisome | Non-matrisome | GO:CC not extracellular matrix | sp|Q9Y2D4|EXC6B_HUMAN | Exocyst complex component 6B | 16.81 | 17.16 | 17.16 |
| 1153 | EXOC7 | Non-matrisome | Non-matrisome | GO:CC not extracellular matrix | sp|Q9UPT5|EXOC7_HUMAN | Exocyst complex component 7 | 17.82 | 18.01 | 18.06 |
| 1154 | EXOC8 | Non-matrisome | Non-matrisome | GO:CC not extracellular matrix | sp|Q8IYI6|EXOC8_HUMAN | Exocyst complex component 8 | 17.77 | 18.03 | 17.98 |
| 1155 | EXOSC1 | Non-matrisome | Non-matrisome | GO:CC not extracellular matrix | sp|Q9Y3B2|EXOS1_HUMAN | Exosome complex component CSL4 | 19.35 | 18.8 | 18.69 |
| 1156 | EXOSC10 | Non-matrisome | Non-matrisome | GO:CC not extracellular matrix | sp|Q01780|EXOSX_HUMAN | Exosome complex component 10 | 18.13 | 17.36 | 17.35 |
| 1157 | EXOSC2 | Non-matrisome | Non-matrisome | GO:CC not extracellular matrix | sp|Q13868|EXOS2_HUMAN | Exosome complex component RRP4 | 16.25 | 15.99 | 15.85 |
| 1158 | EXOSC3 | Non-matrisome | Non-matrisome | GO:CC not extracellular matrix | sp|Q9NQT5|EXOS3_HUMAN | Exosome complex component RRP40 | 18.44 | 17.79 | 17.86 |
| 1159 | EXOSC4 | Non-matrisome | Non-matrisome | GO:CC not extracellular matrix | sp|Q9NPD3|EXOS4_HUMAN | Exosome complex component RRP41 | 17.96 | 17.63 | 17.43 |
| 1160 | EXOSC5 | Non-matrisome | Non-matrisome | GO:CC not extracellular matrix | sp|Q9NQT4|EXOS5_HUMAN | Exosome complex component RRP46 | 17.68 | 17.24 | 17.11 |
| 1161 | EXOSC6 | Non-matrisome | Non-matrisome | GO:CC not extracellular matrix | sp|Q5RKV6|EXOS6_HUMAN | Exosome complex component MTR3 | 17.45 | 16.97 | 16.83 |
| 1162 | EXOSC7 | Non-matrisome | Non-matrisome | GO:CC not extracellular matrix | sp|Q15024|EXOS7_HUMAN | Exosome complex component RRP42 | 17.95 | 17.57 | 17.45 |
| 1163 | EXOSC9 | Non-matrisome | Non-matrisome | GO:CC not extracellular matrix | sp|Q06265|EXOS9_HUMAN | Exosome complex component RRP45 | 17.2 | 16.58 | 16.42 |
| 1164 | EZR | Non-matrisome | Non-matrisome | GO:CC not extracellular matrix | sp|P15311|EZRI_HUMAN | Ezrin | 19.47 | 18.35 | 17.89 |
| 1165 | F11R | Non-matrisome | Non-matrisome | GO:CC not extracellular matrix | sp|Q9Y624|JAM1_HUMAN | Junctional adhesion molecule A | 15.95 | 17.7 | 16.98 |
| 1166 | F13A1 | Matrisome-associated | ECM Regulators | GO:CC extracellular matrix (GO:0005576, GO:0005578 & GO:0031012) & GO:CC not extracellular matrix | sp|P00488|F13A_HUMAN | Coagulation factor XIII A chain | 18.21 | 16.39 | 18.77 |
| 1167 | F2 | Matrisome-associated | ECM Regulators | GO:CC extracellular matrix (GO:0005576, GO:0005578 & GO:0031012) & GO:CC not extracellular matrix | sp|P00734|THRB_HUMAN | Prothrombin | 17.67 | 18.3 | 19.85 |
| 1168 | F5 | Non-matrisome | Non-matrisome | GO:CC extracellular matrix (GO:0005576, GO:0005578 & GO:0031012) & GO:CC not extracellular matrix | sp|P12259|FA5_HUMAN | Coagulation factor V | 17.26 | 19.73 | 19.89 |
| 1169 | F9 | Matrisome-associated | ECM Regulators | GO:CC extracellular matrix (GO:0005576, GO:0005578 & GO:0031012) & GO:CC not extracellular matrix | sp|P00740|FA9_HUMAN | Coagulation factor IX | 13.42 | 15.67 | 15.55 |
| 1170 | FABP3 | Non-matrisome | Non-matrisome | GO:CC not extracellular matrix | sp|P05413|FABPH_HUMAN | Fatty acid-binding protein, heart | 17.32 | 17.79 | 18.4 |
| 1171 | FABP5 | Non-matrisome | Non-matrisome | GO:CC extracellular matrix (GO:0005576, GO:0005578 & GO:0031012) & GO:CC not extracellular matrix | sp|Q01469|FABP5_HUMAN | Fatty acid-binding protein 5 | 19.65 | 18.97 | 19.01 |
| 1172 | FAF1 | Non-matrisome | Non-matrisome | GO:CC not extracellular matrix | sp|Q9UNN5|FAF1_HUMAN | FAS-associated factor 1 | 17.85 | 17.09 | 17.13 |
| 1173 | FAF2 | Non-matrisome | Non-matrisome | GO:CC extracellular matrix (GO:0005576, GO:0005578 & GO:0031012) & GO:CC not extracellular matrix | sp|Q96CS3|FAF2_HUMAN | FAS-associated factor 2 | 17.52 | 17.62 | 17.53 |
| 1174 | FAH | Non-matrisome | Non-matrisome | GO:CC not extracellular matrix | sp|P16930|FAAA_HUMAN | Fumarylacetoacetase | 19.39 | 19.53 | 19.7 |
| 1175 | FAHD1 | Non-matrisome | Non-matrisome | GO:CC not extracellular matrix | sp|Q6P587|FAHD1_HUMAN | Acylpyruvase FAHD1, mitochondrial | 18.16 | 18.57 | 18.38 |
| 1176 | FAHD2A | Non-matrisome | Non-matrisome | GO:CC not extracellular matrix | sp|Q96GK7|FAH2A_HUMAN | Fumarylacetoacetate hydrolase domain-containing protein 2A | 18.63 | 18.83 | 18.67 |
| 1177 | FAM107B | Non-matrisome | Non-matrisome | gene not present in GO IDs | sp|Q9H098|F107B_HUMAN | Protein FAM107B | 15.83 | 15.49 | 16.02 |
| 1178 | FAM114A1 | Non-matrisome | Non-matrisome | GO:CC not extracellular matrix | sp|Q8IWE2|NXP20_HUMAN | Protein NOXP20 | 18.12 | 17.88 | 17.83 |
| 1179 | FAM114A2 | Non-matrisome | Non-matrisome | GO:CC not extracellular matrix | sp|Q9NRY5|F1142_HUMAN | Protein FAM114A2 | 17.89 | 16.87 | 16.96 |
| 1180 | FAM120A | Non-matrisome | Non-matrisome | GO:CC not extracellular matrix | sp|Q9NZB2|F120A_HUMAN | Constitutive coactivator of PPAR-gamma-like protein 1 | 18.4 | 18.5 | 18.41 |
| 1181 | FAM120B | Non-matrisome | Non-matrisome | GO:CC not extracellular matrix | sp|Q96EK7|F120B_HUMAN | Constitutive coactivator of peroxisome proliferator-activated receptor gamma | 16 | 16.05 | 16.02 |
| 1182 | FAM162A | Non-matrisome | Non-matrisome | GO:CC not extracellular matrix | sp|Q96A26|F162A_HUMAN | Protein FAM162A | 19.23 | 20.91 | 20.35 |
| 1183 | FAM180A | Non-matrisome | Non-matrisome | GO:CC extracellular matrix (GO:0005576, GO:0005578 & GO:0031012) | sp|Q6UWF9|F180A_HUMAN | Protein FAM180A | 17.95 | 17.07 | 17.09 |
| 1184 | FAM3C | Non-matrisome | Non-matrisome | GO:CC extracellular matrix (GO:0005576, GO:0005578 & GO:0031012) & GO:CC not extracellular matrix | sp|Q92520|FAM3C_HUMAN | Protein FAM3C | 19.76 | 18.88 | 19.23 |
| 1185 | FAM83G | Non-matrisome | Non-matrisome | GO:CC not extracellular matrix | sp|A6ND36|FA83G_HUMAN | Protein FAM83G | 16.15 | 15.14 | 15.31 |
| 1186 | FAM91A1 | Non-matrisome | Non-matrisome | GO:CC not extracellular matrix | sp|Q658Y4|F91A1_HUMAN | Protein FAM91A1 | 17.17 | 17.27 | 17.14 |
| 1187 | FAM98A | Non-matrisome | Non-matrisome | GO:CC not extracellular matrix | sp|Q8NCA5|FA98A_HUMAN | Protein FAM98A | 18.53 | 18.36 | 18.22 |
| 1188 | FAM98B | Non-matrisome | Non-matrisome | GO:CC not extracellular matrix | sp|Q52LJ0|FA98B_HUMAN | Protein FAM98B | 16.71 | 17.18 | 17.15 |
| 1189 | FAM98C | Non-matrisome | Non-matrisome | GO:CC not extracellular matrix | sp|Q17RN3|FA98C_HUMAN | Protein FAM98C | 15.57 | 16.16 | 16.42 |
| 1190 | FAP | Non-matrisome | Non-matrisome | GO:CC not extracellular matrix | sp|Q12884|SEPR_HUMAN | Prolyl endopeptidase FAP | 17.06 | 18.3 | 18.53 |
| 1191 | FARP1 | Non-matrisome | Non-matrisome | GO:CC not extracellular matrix | sp|Q9Y4F1|FARP1_HUMAN | FERM, ARHGEF and pleckstrin domain-containing protein 1 | 17.76 | 18.27 | 18.27 |
| 1192 | FARS2 | Non-matrisome | Non-matrisome | GO:CC not extracellular matrix | sp|O95363|SYFM_HUMAN | Phenylalanine--tRNA ligase, mitochondrial | 16.79 | 16.76 | 16.54 |
| 1193 | FARSA | Non-matrisome | Non-matrisome | GO:CC not extracellular matrix | sp|Q9Y285|SYFA_HUMAN | Phenylalanine--tRNA ligase alpha subunit | 17.97 | 17.83 | 17.76 |
| 1194 | FARSB | Non-matrisome | Non-matrisome | GO:CC not extracellular matrix | sp|Q9NSD9|SYFB_HUMAN | Phenylalanine--tRNA ligase beta subunit | 18.61 | 18.54 | 18.35 |
| 1195 | FASN | Non-matrisome | Non-matrisome | GO:CC not extracellular matrix | sp|P49327|FAS_HUMAN | Fatty acid synthase | 18.93 | 18.14 | 18.13 |
| 1196 | FAU | Non-matrisome | Non-matrisome | GO:CC not extracellular matrix | sp|P62861|RS30_HUMAN | Ubiquitin-like FUBI-ribosomal protein eS30 fusion protein | 22.09 | 21.4 | 21.31 |
| 1197 | FBL | Non-matrisome | Non-matrisome | GO:CC not extracellular matrix | sp|P22087|FBRL_HUMAN | rRNA 2'-O-methyltransferase fibrillarin | 17.83 | 17.81 | 17.89 |
| 1198 | FBLN1 | Core matrisome | ECM Glycoproteins | GO:CC extracellular matrix (GO:0005576, GO:0005578 & GO:0031012) & GO:CC not extracellular matrix | sp|P23142|FBLN1_HUMAN | Fibulin-1 | 15.92 | 19.58 | 19.46 |
| 1199 | FBLN2 | Core matrisome | ECM Glycoproteins | GO:CC extracellular matrix (GO:0005576, GO:0005578 & GO:0031012) & GO:CC not extracellular matrix | sp|P98095|FBLN2_HUMAN | Fibulin-2 | 13.8 | 17.93 | 18.47 |
| 1200 | FBLN5 | Core matrisome | ECM Glycoproteins | GO:CC extracellular matrix (GO:0005576, GO:0005578 & GO:0031012) & GO:CC not extracellular matrix | sp|Q9UBX5|FBLN5_HUMAN | Fibulin-5 | 16.02 | 16.28 | 15.78 |
| 1201 | FBN1 | Core matrisome | ECM Glycoproteins | GO:CC extracellular matrix (GO:0005576, GO:0005578 & GO:0031012) & GO:CC not extracellular matrix | sp|P35555|FBN1_HUMAN | Fibrillin-1 | 15.3 | 17.23 | 16.88 |
| 1202 | FBN2 | Core matrisome | ECM Glycoproteins | GO:CC extracellular matrix (GO:0005576, GO:0005578 & GO:0031012) & GO:CC not extracellular matrix | sp|P35556|FBN2_HUMAN | Fibrillin-2 | 17.58 | 16.83 | 16.68 |
| 1203 | FBXL8 | Non-matrisome | Non-matrisome | GO:CC not extracellular matrix | sp|Q96CD0|FBXL8_HUMAN | F-box/LRR-repeat protein 8 | 15.28 | 16.58 | 16.54 |
| 1204 | FBXO22 | Non-matrisome | Non-matrisome | GO:CC not extracellular matrix | sp|Q8NEZ5|FBX22_HUMAN | F-box only protein 22 | 18.17 | 18.37 | 18.21 |
| 1205 | FBXO3 | Non-matrisome | Non-matrisome | GO:CC not extracellular matrix | sp|Q9UK99|FBX3_HUMAN | F-box only protein 3 | 16.13 | 16.52 | 16.63 |
| 1206 | FBXO6 | Non-matrisome | Non-matrisome | GO:CC not extracellular matrix | sp|Q9NRD1|FBX6_HUMAN | F-box only protein 6 | 15.02 | 15.36 | 15.33 |
| 1207 | FBXO7 | Non-matrisome | Non-matrisome | GO:CC not extracellular matrix | sp|Q9Y3I1|FBX7_HUMAN | F-box only protein 7 | 17.77 | 17.82 | 17.68 |
| 1208 | FCGRT | Non-matrisome | Non-matrisome | GO:CC not extracellular matrix | sp|P55899|FCGRN_HUMAN | IgG receptor FcRn large subunit p51 | 14.33 | 15.92 | 16.19 |
| 1209 | FCHO2 | Non-matrisome | Non-matrisome | GO:CC not extracellular matrix | sp|Q0JRZ9|FCHO2_HUMAN | F-BAR domain only protein 2 | 17.32 | 17.37 | 17.47 |
| 1210 | FCSK | Non-matrisome | Non-matrisome | GO:CC not extracellular matrix | sp|Q8N0W3|FCSK_HUMAN | L-fucose kinase | 14.59 | 15.24 | 15.24 |
| 1211 | FDFT1 | Non-matrisome | Non-matrisome | GO:CC not extracellular matrix | sp|P37268|FDFT_HUMAN | Squalene synthase | 17.97 | 16.17 | 16.88 |
| 1212 | FDPS | Non-matrisome | Non-matrisome | GO:CC not extracellular matrix | sp|P14324|FPPS_HUMAN | Farnesyl pyrophosphate synthase | 17.42 | 17.14 | 17.05 |
| 1213 | FDXR | Non-matrisome | Non-matrisome | GO:CC not extracellular matrix | sp|P22570|ADRO_HUMAN | NADPH:adrenodoxin oxidoreductase, mitochondrial | 17.23 | 17.95 | 17.8 |
| 1214 | FECH | Non-matrisome | Non-matrisome | GO:CC not extracellular matrix | sp|P22830|HEMH_HUMAN | Ferrochelatase, mitochondrial | 17.73 | 18.81 | 18.76 |
| 1215 | FEN1 | Non-matrisome | Non-matrisome | GO:CC not extracellular matrix | sp|P39748|FEN1_HUMAN | Flap endonuclease 1 | 19.78 | 19.18 | 18.87 |
| 1216 | FER | Non-matrisome | Non-matrisome | GO:CC extracellular matrix (GO:0005576, GO:0005578 & GO:0031012) & GO:CC not extracellular matrix | sp|P16591|FER_HUMAN | Tyrosine-protein kinase Fer | 16.65 | 17.31 | 17.45 |
| 1217 | FERMT1 | Non-matrisome | Non-matrisome | GO:CC not extracellular matrix | sp|Q9BQL6|FERM1_HUMAN | Fermitin family homolog 1 | 17.21 | 17.14 | 17.19 |
| 1218 | FERMT2 | Non-matrisome | Non-matrisome | GO:CC not extracellular matrix | sp|Q96AC1|FERM2_HUMAN | Fermitin family homolog 2 | 18.84 | 18.42 | 18.39 |
| 1219 | FERMT3 | Non-matrisome | Non-matrisome | GO:CC extracellular matrix (GO:0005576, GO:0005578 & GO:0031012) & GO:CC not extracellular matrix | sp|Q86UX7|URP2_HUMAN | Fermitin family homolog 3 | 12.78 | 14.04 | 15.17 |
| 1220 | FGA | Core matrisome | ECM Glycoproteins | GO:CC extracellular matrix (GO:0005576, GO:0005578 & GO:0031012) & GO:CC not extracellular matrix | sp|P02671|FIBA_HUMAN | Fibrinogen alpha chain | 17.62 | 16.53 | 18.23 |
| 1221 | FGB | Core matrisome | ECM Glycoproteins | GO:CC extracellular matrix (GO:0005576, GO:0005578 & GO:0031012) & GO:CC not extracellular matrix | sp|P02675|FIBB_HUMAN | Fibrinogen beta chain | 17.95 | 16.6 | 18.55 |
| 1222 | FGG | Core matrisome | ECM Glycoproteins | GO:CC extracellular matrix (GO:0005576, GO:0005578 & GO:0031012) & GO:CC not extracellular matrix | sp|P02679|FIBG_HUMAN | Fibrinogen gamma chain | 14.69 | 12.98 | 15.43 |
| 1223 | FH | Non-matrisome | Non-matrisome | GO:CC not extracellular matrix | sp|P07954|FUMH_HUMAN | Fumarate hydratase, mitochondrial | 19.83 | 19.47 | 19.21 |
| 1224 | FHL1 | Non-matrisome | Non-matrisome | GO:CC not extracellular matrix | sp|Q13642|FHL1_HUMAN | Four and a half LIM domains protein 1 | 17.69 | 18.03 | 17.93 |
| 1225 | FHL2 | Non-matrisome | Non-matrisome | GO:CC not extracellular matrix | sp|Q14192|FHL2_HUMAN | Four and a half LIM domains protein 2 | 18.8 | 17.96 | 18.09 |
| 1226 | FHL3 | Non-matrisome | Non-matrisome | GO:CC not extracellular matrix | sp|Q13643|FHL3_HUMAN | Four and a half LIM domains protein 3 | 19.84 | 19.28 | 19.1 |
| 1227 | FIG4 | Non-matrisome | Non-matrisome | GO:CC extracellular matrix (GO:0005576, GO:0005578 & GO:0031012) & GO:CC not extracellular matrix | sp|Q92562|FIG4_HUMAN | Polyphosphoinositide phosphatase | 15.79 | 16.44 | 16.44 |
| 1228 | FILIP1L | Non-matrisome | Non-matrisome | GO:CC not extracellular matrix | sp|Q4L180|FIL1L_HUMAN | Filamin A-interacting protein 1-like | 17.32 | 15.48 | 15.69 |
| 1229 | FIS1 | Non-matrisome | Non-matrisome | GO:CC not extracellular matrix | sp|Q9Y3D6|FIS1_HUMAN | Mitochondrial fission 1 protein | 17.47 | 17.8 | 17.62 |
| 1230 | FKBP10 | Non-matrisome | Non-matrisome | GO:CC not extracellular matrix | sp|Q96AY3|FKB10_HUMAN | Peptidyl-prolyl cis-trans isomerase FKBP10 | 18.49 | 18.19 | 18.25 |
| 1231 | FKBP11 | Non-matrisome | Non-matrisome | GO:CC not extracellular matrix | sp|Q9NYL4|FKB11_HUMAN | Peptidyl-prolyl cis-trans isomerase FKBP11 | 19.31 | 19.32 | 19.38 |
| 1232 | FKBP15 | Non-matrisome | Non-matrisome | GO:CC not extracellular matrix | sp|Q5T1M5|FKB15_HUMAN | FK506-binding protein 15 | 17.79 | 17.85 | 17.87 |
| 1233 | FKBP1A | Non-matrisome | Non-matrisome | GO:CC not extracellular matrix | sp|P62942|FKB1A_HUMAN | Peptidyl-prolyl cis-trans isomerase FKBP1A | 18.5 | 18.57 | 18.79 |
| 1234 | FKBP2 | Non-matrisome | Non-matrisome | GO:CC not extracellular matrix | sp|P26885|FKBP2_HUMAN | Peptidyl-prolyl cis-trans isomerase FKBP2 | 19.43 | 19.81 | 19.85 |
| 1235 | FKBP3 | Non-matrisome | Non-matrisome | GO:CC not extracellular matrix | sp|Q00688|FKBP3_HUMAN | Peptidyl-prolyl cis-trans isomerase FKBP3 | 18.22 | 18.23 | 18.38 |
| 1236 | FKBP4 | Non-matrisome | Non-matrisome | GO:CC not extracellular matrix | sp|Q02790|FKBP4_HUMAN | Peptidyl-prolyl cis-trans isomerase FKBP4 | 19.05 | 18.77 | 18.92 |
| 1237 | FKBP5 | Non-matrisome | Non-matrisome | GO:CC not extracellular matrix | sp|Q13451|FKBP5_HUMAN | Peptidyl-prolyl cis-trans isomerase FKBP5 | 18.74 | 16.45 | 16.23 |
| 1238 | FKBP8 | Non-matrisome | Non-matrisome | GO:CC not extracellular matrix | sp|Q14318|FKBP8_HUMAN | Peptidyl-prolyl cis-trans isomerase FKBP8 | 18.87 | 18.43 | 18.52 |
| 1239 | FKBP9 | Non-matrisome | Non-matrisome | GO:CC not extracellular matrix | sp|O95302|FKBP9_HUMAN | Peptidyl-prolyl cis-trans isomerase FKBP9 | 19.6 | 19.44 | 19.56 |
| 1240 | FLAD1 | Non-matrisome | Non-matrisome | GO:CC not extracellular matrix | sp|Q8NFF5|FAD1_HUMAN | FAD synthase | 17.18 | 17.34 | 17.29 |
| 1241 | FLII | Non-matrisome | Non-matrisome | GO:CC not extracellular matrix | sp|Q13045|FLII_HUMAN | Protein flightless-1 homolog | 18.61 | 17.89 | 17.71 |
| 1242 | FLNA | Non-matrisome | Non-matrisome | GO:CC extracellular matrix (GO:0005576, GO:0005578 & GO:0031012) & GO:CC not extracellular matrix | sp|P21333|FLNA_HUMAN | Filamin-A | 19.32 | 19.06 | 18.73 |
| 1243 | FLNB | Non-matrisome | Non-matrisome | GO:CC not extracellular matrix | sp|O75369|FLNB_HUMAN | Filamin-B | 19.8 | 19.64 | 18.83 |
| 1244 | FLNC | Non-matrisome | Non-matrisome | GO:CC not extracellular matrix | sp|Q14315|FLNC_HUMAN | Filamin-C | 19.08 | 18.98 | 18.34 |
| 1245 | FLOT1 | Non-matrisome | Non-matrisome | GO:CC not extracellular matrix | sp|O75955|FLOT1_HUMAN | Flotillin-1 | 18.15 | 18.71 | 18.65 |
| 1246 | FLOT2 | Non-matrisome | Non-matrisome | GO:CC not extracellular matrix | sp|Q14254|FLOT2_HUMAN | Flotillin-2 | 17.81 | 18.72 | 18.63 |
| 1247 | FMNL1 | Non-matrisome | Non-matrisome | GO:CC not extracellular matrix | sp|O95466|FMNL1_HUMAN | Formin-like protein 1 | 16.85 | 18.04 | 18.26 |
| 1248 | FMNL2 | Non-matrisome | Non-matrisome | GO:CC not extracellular matrix | sp|Q96PY5|FMNL2_HUMAN | Formin-like protein 2 | 16.91 | 18.48 | 18.74 |
| 1249 | FMNL3 | Non-matrisome | Non-matrisome | GO:CC not extracellular matrix | sp|Q8IVF7|FMNL3_HUMAN | Formin-like protein 3 | 16.65 | 17.28 | 17.23 |
| 1250 | FMR1 | Non-matrisome | Non-matrisome | GO:CC not extracellular matrix | sp|Q06787|FMR1_HUMAN | Fragile X messenger ribonucleoprotein 1 | 16.85 | 16.98 | 17.02 |
| 1251 | FN1 | Core matrisome | ECM Glycoproteins | GO:CC extracellular matrix (GO:0005576, GO:0005578 & GO:0031012) & GO:CC not extracellular matrix | sp|P02751|FINC_HUMAN | Fibronectin | 17.03 | 18.32 | 18.6 |
| 1252 | FN3KRP | Non-matrisome | Non-matrisome | GO:CC not extracellular matrix | sp|Q9HA64|KT3K_HUMAN | Ketosamine-3-kinase | 17.34 | 17.54 | 17.44 |
| 1253 | FNBP1 | Non-matrisome | Non-matrisome | GO:CC not extracellular matrix | sp|Q96RU3|FNBP1_HUMAN | Formin-binding protein 1 | 18.38 | 18.57 | 18.51 |
| 1254 | FNDC3A | Non-matrisome | Non-matrisome | GO:CC not extracellular matrix | sp|Q9Y2H6|FND3A_HUMAN | Fibronectin type-III domain-containing protein 3A | 17.7 | 17.75 | 17.75 |
| 1255 | FNDC3B | Non-matrisome | Non-matrisome | GO:CC not extracellular matrix | sp|Q53EP0|FND3B_HUMAN | Fibronectin type III domain-containing protein 3B | 18.31 | 17.55 | 17.44 |
| 1256 | FNTA | Non-matrisome | Non-matrisome | GO:CC not extracellular matrix | sp|P49354|FNTA_HUMAN | Protein farnesyltransferase/geranylgeranyltransferase type-1 subunit alpha | 16.1 | 16.2 | 16.38 |
| 1257 | FRMD8 | Non-matrisome | Non-matrisome | GO:CC not extracellular matrix | sp|Q9BZ67|FRMD8_HUMAN | FERM domain-containing protein 8 | 15.72 | 16.27 | 16.67 |
| 1258 | FRMPD1 | Non-matrisome | Non-matrisome | GO:CC not extracellular matrix | sp|Q5SYB0|FRPD1_HUMAN | FERM and PDZ domain-containing protein 1 | 19.67 | 21.54 | 21.92 |
| 1259 | FRRS1 | Non-matrisome | Non-matrisome | GO:CC not extracellular matrix | sp|Q6ZNA5|FRRS1_HUMAN | Ferric-chelate reductase 1 | 17.41 | 19.01 | 19.24 |
| 1260 | FRYL | Non-matrisome | Non-matrisome | GO:CC not extracellular matrix | sp|O94915|FRYL_HUMAN | Protein furry homolog-like | 16.92 | 16.46 | 16.33 |
| 1261 | FSCN1 | Non-matrisome | Non-matrisome | GO:CC not extracellular matrix | sp|Q16658|FSCN1_HUMAN | Fascin | 18.31 | 18.01 | 17.82 |
| 1262 | FTH1 | Non-matrisome | Non-matrisome | GO:CC extracellular matrix (GO:0005576, GO:0005578 & GO:0031012) & GO:CC not extracellular matrix | sp|P02794|FRIH_HUMAN | Ferritin heavy chain | 17.84 | 18.6 | 19.43 |
| 1263 | FTL | Non-matrisome | Non-matrisome | GO:CC extracellular matrix (GO:0005576, GO:0005578 & GO:0031012) & GO:CC not extracellular matrix | sp|P02792|FRIL_HUMAN | Ferritin light chain | 15.65 | 16.88 | 17.66 |
| 1264 | FTO | Non-matrisome | Non-matrisome | GO:CC not extracellular matrix | sp|Q9C0B1|FTO_HUMAN | Alpha-ketoglutarate-dependent dioxygenase FTO | 17.49 | 17.49 | 17.65 |
| 1265 | FTSJ3 | Non-matrisome | Non-matrisome | GO:CC not extracellular matrix | sp|Q8IY81|SPB1_HUMAN | pre-rRNA 2'-O-ribose RNA methyltransferase FTSJ3 | 18.57 | 18.21 | 18.14 |
| 1266 | FUBP1 | Non-matrisome | Non-matrisome | GO:CC not extracellular matrix | sp|Q96AE4|FUBP1_HUMAN | Far upstream element-binding protein 1 | 18.51 | 17.79 | 17.8 |
| 1267 | FUBP3 | Non-matrisome | Non-matrisome | GO:CC not extracellular matrix | sp|Q96I24|FUBP3_HUMAN | Far upstream element-binding protein 3 | 17.66 | 17.56 | 17.69 |
| 1268 | FUCA1 | Non-matrisome | Non-matrisome | GO:CC extracellular matrix (GO:0005576, GO:0005578 & GO:0031012) & GO:CC not extracellular matrix | sp|P04066|FUCO_HUMAN | Tissue alpha-L-fucosidase | 14.7 | 17.43 | 18.67 |
| 1269 | FUCA2 | Non-matrisome | Non-matrisome | GO:CC extracellular matrix (GO:0005576, GO:0005578 & GO:0031012) & GO:CC not extracellular matrix | sp|Q9BTY2|FUCO2_HUMAN | Plasma alpha-L-fucosidase | 16.2 | 17.61 | 18.32 |
| 1270 | FUNDC2 | Non-matrisome | Non-matrisome | GO:CC not extracellular matrix | sp|Q9BWH2|FUND2_HUMAN | FUN14 domain-containing protein 2 | 18.65 | 18.68 | 18.81 |
| 1271 | FUS | Non-matrisome | Non-matrisome | GO:CC not extracellular matrix | sp|P35637|FUS_HUMAN | RNA-binding protein FUS | 16.73 | 15.93 | 15.97 |
| 1272 | FXR1 | Non-matrisome | Non-matrisome | GO:CC not extracellular matrix | sp|P51114|FXR1_HUMAN | RNA-binding protein FXR1 | 17.97 | 17.83 | 17.77 |
| 1273 | FYCO1 | Non-matrisome | Non-matrisome | GO:CC not extracellular matrix | sp|Q9BQS8|FYCO1_HUMAN | FYVE and coiled-coil domain-containing protein 1 | 16.59 | 17.59 | 17.75 |
| 1274 | FYTTD1 | Non-matrisome | Non-matrisome | GO:CC not extracellular matrix | sp|Q96QD9|UIF_HUMAN | UAP56-interacting factor | 18.94 | 18.68 | 18.98 |
| 1275 | G3BP1 | Non-matrisome | Non-matrisome | GO:CC not extracellular matrix | sp|Q13283|G3BP1_HUMAN | Ras GTPase-activating protein-binding protein 1 | 18.85 | 17.79 | 18.15 |
| 1276 | G3BP2 | Non-matrisome | Non-matrisome | GO:CC not extracellular matrix | sp|Q9UN86|G3BP2_HUMAN | Ras GTPase-activating protein-binding protein 2 | 19.15 | 18.69 | 18.74 |
| 1277 | G6PD | Non-matrisome | Non-matrisome | GO:CC not extracellular matrix | sp|P11413|G6PD_HUMAN | Glucose-6-phosphate 1-dehydrogenase | 18.21 | 18.17 | 18.36 |
| 1278 | GAA | Non-matrisome | Non-matrisome | GO:CC not extracellular matrix | sp|P10253|LYAG_HUMAN | Lysosomal alpha-glucosidase | 16.01 | 17.2 | 17.66 |
| 1279 | GABARAPL2 | Non-matrisome | Non-matrisome | GO:CC not extracellular matrix | sp|P60520|GBRL2_HUMAN | Gamma-aminobutyric acid receptor-associated protein-like 2 | 18.97 | 17.56 | 17.78 |
| 1280 | GAK | Non-matrisome | Non-matrisome | GO:CC not extracellular matrix | sp|O14976|GAK_HUMAN | Cyclin-G-associated kinase | 17.59 | 17.34 | 17.42 |
| 1281 | GALC | Non-matrisome | Non-matrisome | GO:CC not extracellular matrix | sp|P54803|GALC_HUMAN | Galactocerebrosidase | 15.44 | 17.41 | 17.55 |
| 1282 | GALE | Non-matrisome | Non-matrisome | GO:CC not extracellular matrix | sp|Q14376|GALE_HUMAN | UDP-glucose 4-epimerase | 18.52 | 18.79 | 18.67 |
| 1283 | GALK1 | Non-matrisome | Non-matrisome | GO:CC not extracellular matrix | sp|P51570|GALK1_HUMAN | Galactokinase | 17.13 | 17.55 | 17.51 |
| 1284 | GALM | Non-matrisome | Non-matrisome | GO:CC not extracellular matrix | sp|Q96C23|GALM_HUMAN | Galactose mutarotase | 16.87 | 17.73 | 17.69 |
| 1285 | GALNT1 | Non-matrisome | Non-matrisome | GO:CC extracellular matrix (GO:0005576, GO:0005578 & GO:0031012) & GO:CC not extracellular matrix | sp|Q10472|GALT1_HUMAN | Polypeptide N-acetylgalactosaminyltransferase 1 | 16.29 | 15.28 | 15.71 |
| 1286 | GALNT2 | Non-matrisome | Non-matrisome | GO:CC extracellular matrix (GO:0005576, GO:0005578 & GO:0031012) & GO:CC not extracellular matrix | sp|Q10471|GALT2_HUMAN | Polypeptide N-acetylgalactosaminyltransferase 2 | 19.21 | 18.36 | 18.31 |
| 1287 | GALT | Non-matrisome | Non-matrisome | GO:CC not extracellular matrix | sp|P07902|GALT_HUMAN | Galactose-1-phosphate uridylyltransferase | 16.56 | 17.1 | 17.21 |
| 1288 | GAN | Non-matrisome | Non-matrisome | GO:CC not extracellular matrix | sp|Q9H2C0|GAN_HUMAN | Gigaxonin | 17.05 | 16.69 | 16.41 |
| 1289 | GANAB | Non-matrisome | Non-matrisome | GO:CC not extracellular matrix | sp|Q14697|GANAB_HUMAN | Neutral alpha-glucosidase AB | 18.73 | 19.27 | 19.02 |
| 1290 | GANC | Non-matrisome | Non-matrisome | GO:CC not extracellular matrix | sp|Q8TET4|GANC_HUMAN | Neutral alpha-glucosidase C | 15.9 | 16.67 | 16.55 |
| 1291 | GAPDH | Non-matrisome | Non-matrisome | GO:CC not extracellular matrix | sp|P04406|G3P_HUMAN | Glyceraldehyde-3-phosphate dehydrogenase | 18.79 | 19.34 | 19.12 |
| 1292 | GAPVD1 | Non-matrisome | Non-matrisome | GO:CC not extracellular matrix | sp|Q14C86|GAPD1_HUMAN | GTPase-activating protein and VPS9 domain-containing protein 1 | 18.16 | 18.07 | 17.92 |
| 1293 | GAR1 | Non-matrisome | Non-matrisome | GO:CC not extracellular matrix | sp|Q9NY12|GAR1_HUMAN | H/ACA ribonucleoprotein complex subunit 1 | 17.31 | 17.44 | 17.44 |
| 1294 | GARS1 | Non-matrisome | Non-matrisome | GO:CC not extracellular matrix | sp|P41250|GARS_HUMAN | Glycine--tRNA ligase | 19.61 | 19.44 | 19.14 |
| 1295 | GART | Non-matrisome | Non-matrisome | GO:CC not extracellular matrix | sp|P22102|PUR2_HUMAN | Trifunctional purine biosynthetic protein adenosine-3 | 19.59 | 19.03 | 18.81 |
| 1296 | GATD1 | Non-matrisome | Non-matrisome | GO:CC not extracellular matrix | sp|Q8NB37|GALD1_HUMAN | Glutamine amidotransferase-like class 1 domain-containing protein 1 | 16.59 | 17.04 | 17.06 |
| 1297 | GBE1 | Non-matrisome | Non-matrisome | GO:CC not extracellular matrix | sp|Q04446|GLGB_HUMAN | 1,4-alpha-glucan-branching enzyme | 19.82 | 20.23 | 19.85 |
| 1298 | GBF1 | Non-matrisome | Non-matrisome | GO:CC not extracellular matrix | sp|Q92538|GBF1_HUMAN | Golgi-specific brefeldin A-resistance guanine nucleotide exchange factor 1 | 16.94 | 16.3 | 16.49 |
| 1299 | GBP1 | Non-matrisome | Non-matrisome | GO:CC extracellular matrix (GO:0005576, GO:0005578 & GO:0031012) & GO:CC not extracellular matrix | sp|P32455|GBP1_HUMAN | Guanylate-binding protein 1 | 19.46 | 19.66 | 19.59 |
| 1300 | GBP2 | Non-matrisome | Non-matrisome | GO:CC not extracellular matrix | sp|P32456|GBP2_HUMAN | Guanylate-binding protein 2 | 17.06 | 17.89 | 18.18 |
| 1301 | GBP5 | Non-matrisome | Non-matrisome | GO:CC not extracellular matrix | sp|Q96PP8|GBP5_HUMAN | Guanylate-binding protein 5 | 16.08 | 16.21 | 16.33 |
| 1302 | GC | Non-matrisome | Non-matrisome | GO:CC extracellular matrix (GO:0005576, GO:0005578 & GO:0031012) & GO:CC not extracellular matrix | sp|P02774|VTDB_HUMAN | Vitamin D-binding protein | 18.53 | 18.25 | 18.11 |
| 1303 | GCA | Non-matrisome | Non-matrisome | GO:CC extracellular matrix (GO:0005576, GO:0005578 & GO:0031012) & GO:CC not extracellular matrix | sp|P28676|GRAN_HUMAN | Grancalcin | 16.56 | 16.85 | 16.87 |
| 1304 | GCC1 | Non-matrisome | Non-matrisome | GO:CC not extracellular matrix | sp|Q96CN9|GCC1_HUMAN | GRIP and coiled-coil domain-containing protein 1 | 16.97 | 16.68 | 16.83 |
| 1305 | GCC2 | Non-matrisome | Non-matrisome | GO:CC not extracellular matrix | sp|Q8IWJ2|GCC2_HUMAN | GRIP and coiled-coil domain-containing protein 2 | 17.07 | 16.68 | 16.82 |
| 1306 | GCDH | Non-matrisome | Non-matrisome | GO:CC not extracellular matrix | sp|Q92947|GCDH_HUMAN | Glutaryl-CoA dehydrogenase, mitochondrial | 16.78 | 17.12 | 16.81 |
| 1307 | GCLC | Non-matrisome | Non-matrisome | GO:CC not extracellular matrix | sp|P48506|GSH1_HUMAN | Glutamate--cysteine ligase catalytic subunit | 16.67 | 16.96 | 17.05 |
| 1308 | GCLM | Non-matrisome | Non-matrisome | GO:CC not extracellular matrix | sp|P48507|GSH0_HUMAN | Glutamate--cysteine ligase regulatory subunit | 17.36 | 17 | 17.47 |
| 1309 | GCN1 | Non-matrisome | Non-matrisome | GO:CC not extracellular matrix | sp|Q92616|GCN1_HUMAN | Stalled ribosome sensor GCN1 | 18.77 | 18.57 | 18.37 |
| 1310 | GDE1 | Non-matrisome | Non-matrisome | GO:CC not extracellular matrix | sp|Q9NZC3|GDE1_HUMAN | Glycerophosphodiester phosphodiesterase 1 | 16.8 | 17.56 | 17.74 |
| 1311 | GDI1 | Non-matrisome | Non-matrisome | GO:CC not extracellular matrix | sp|P31150|GDIA_HUMAN | Rab GDP dissociation inhibitor alpha | 18.29 | 18.67 | 18.66 |
| 1312 | GDI2 | Non-matrisome | Non-matrisome | GO:CC extracellular matrix (GO:0005576, GO:0005578 & GO:0031012) & GO:CC not extracellular matrix | sp|P50395|GDIB_HUMAN | Rab GDP dissociation inhibitor beta | 18.97 | 18.66 | 18.76 |
| 1313 | GEMIN5 | Non-matrisome | Non-matrisome | GO:CC not extracellular matrix | sp|Q8TEQ6|GEMI5_HUMAN | Gem-associated protein 5 | 17.9 | 17.26 | 17.09 |
| 1314 | GET3 | Non-matrisome | Non-matrisome | GO:CC not extracellular matrix | sp|O43681|GET3_HUMAN | ATPase GET3 | 20.98 | 21.1 | 20.94 |
| 1315 | GET4 | Non-matrisome | Non-matrisome | GO:CC not extracellular matrix | sp|Q7L5D6|GET4_HUMAN | Golgi to ER traffic protein 4 homolog | 17.79 | 17.54 | 17.47 |
| 1316 | GFM1 | Non-matrisome | Non-matrisome | GO:CC not extracellular matrix | sp|Q96RP9|EFGM_HUMAN | Elongation factor G, mitochondrial | 18.94 | 18.75 | 18.73 |
| 1317 | GFOD2 | Non-matrisome | Non-matrisome | GO:CC extracellular matrix (GO:0005576, GO:0005578 & GO:0031012) & GO:CC not extracellular matrix | sp|Q3B7J2|GFOD2_HUMAN | Glucose-fructose oxidoreductase domain-containing protein 2 | 13.86 | 14.48 | 14.67 |
| 1318 | GFPT1 | Non-matrisome | Non-matrisome | GO:CC not extracellular matrix | sp|Q06210|GFPT1_HUMAN | Glutamine--fructose-6-phosphate aminotransferase [isomerizing] 1 | 18.53 | 18.14 | 17.96 |
| 1319 | GFUS | Non-matrisome | Non-matrisome | GO:CC not extracellular matrix | sp|Q13630|FCL_HUMAN | GDP-L-fucose synthase | 17.81 | 17.99 | 18.02 |
| 1320 | GGACT | Non-matrisome | Non-matrisome | GO:CC not extracellular matrix | sp|Q9BVM4|GGACT_HUMAN | Gamma-glutamylaminecyclotransferase | 15.35 | 15.47 | 15.53 |
| 1321 | GGCT | Non-matrisome | Non-matrisome | GO:CC not extracellular matrix | sp|O75223|GGCT_HUMAN | Gamma-glutamylcyclotransferase | 18.53 | 17.82 | 17.84 |
| 1322 | GGH | Non-matrisome | Non-matrisome | GO:CC extracellular matrix (GO:0005576, GO:0005578 & GO:0031012) & GO:CC not extracellular matrix | sp|Q92820|GGH_HUMAN | Gamma-glutamyl hydrolase | 17.66 | 18.79 | 19.2 |
| 1323 | GGPS1 | Non-matrisome | Non-matrisome | GO:CC not extracellular matrix | sp|O95749|GGPPS_HUMAN | Geranylgeranyl pyrophosphate synthase | 17.12 | 17.69 | 17.61 |
| 1324 | GGT5 | Non-matrisome | Non-matrisome | GO:CC not extracellular matrix | sp|P36269|GGT5_HUMAN | Glutathione hydrolase 5 proenzyme | 16.34 | 17.35 | 17.56 |
| 1325 | GHDC | Non-matrisome | Non-matrisome | GO:CC extracellular matrix (GO:0005576, GO:0005578 & GO:0031012) & GO:CC not extracellular matrix | sp|Q8N2G8|GHDC_HUMAN | GH3 domain-containing protein | 17.68 | 18.1 | 18 |
| 1326 | GHITM | Non-matrisome | Non-matrisome | GO:CC not extracellular matrix | sp|Q9H3K2|GHITM_HUMAN | Growth hormone-inducible transmembrane protein | 17.62 | 17.94 | 17.84 |
| 1327 | GID8 | Non-matrisome | Non-matrisome | GO:CC not extracellular matrix | sp|Q9NWU2|GID8_HUMAN | Glucose-induced degradation protein 8 homolog | 17.79 | 17.4 | 17.52 |
| 1328 | GIGYF2 | Non-matrisome | Non-matrisome | GO:CC not extracellular matrix | sp|Q6Y7W6|GGYF2_HUMAN | GRB10-interacting GYF protein 2 | 17.17 | 16.31 | 16.62 |
| 1329 | GIPC1 | Non-matrisome | Non-matrisome | GO:CC not extracellular matrix | sp|O14908|GIPC1_HUMAN | PDZ domain-containing protein GIPC1 | 18.34 | 17.28 | 17.54 |
| 1330 | GIT1 | Non-matrisome | Non-matrisome | GO:CC not extracellular matrix | sp|Q9Y2X7|GIT1_HUMAN | ARF GTPase-activating protein GIT1 | 16.66 | 16.95 | 17.05 |
| 1331 | GIT2 | Non-matrisome | Non-matrisome | GO:CC not extracellular matrix | sp|Q14161|GIT2_HUMAN | ARF GTPase-activating protein GIT2 | 17.74 | 17.74 | 17.72 |
| 1332 | GJA1 | Non-matrisome | Non-matrisome | GO:CC not extracellular matrix | sp|P17302|CXA1_HUMAN | Gap junction alpha-1 protein | 16.83 | 18.2 | 17.55 |
| 1333 | GLA | Non-matrisome | Non-matrisome | GO:CC extracellular matrix (GO:0005576, GO:0005578 & GO:0031012) & GO:CC not extracellular matrix | sp|P06280|AGAL_HUMAN | Alpha-galactosidase A | 16.67 | 16.82 | 17.42 |
| 1334 | GLB1 | Non-matrisome | Non-matrisome | GO:CC extracellular matrix (GO:0005576, GO:0005578 & GO:0031012) & GO:CC not extracellular matrix | sp|P16278|BGAL_HUMAN | Beta-galactosidase | 17.8 | 19.43 | 19.88 |
| 1335 | GLG1 | Non-matrisome | Non-matrisome | GO:CC extracellular matrix (GO:0005576, GO:0005578 & GO:0031012) & GO:CC not extracellular matrix | sp|Q92896|GSLG1_HUMAN | Golgi apparatus protein 1 | 18.52 | 18.86 | 19.07 |
| 1336 | GLIPR2 | Non-matrisome | Non-matrisome | GO:CC not extracellular matrix | sp|Q9H4G4|GAPR1_HUMAN | Golgi-associated plant pathogenesis-related protein 1 | 17.25 | 17.65 | 17.65 |
| 1337 | GLMN | Non-matrisome | Non-matrisome | GO:CC not extracellular matrix | sp|Q92990|GLMN_HUMAN | Glomulin | 16.97 | 16.81 | 16.72 |
| 1338 | GLO1 | Non-matrisome | Non-matrisome | GO:CC not extracellular matrix | sp|Q04760|LGUL_HUMAN | Lactoylglutathione lyase | 19.64 | 19.69 | 19.62 |
| 1339 | GLOD4 | Non-matrisome | Non-matrisome | GO:CC not extracellular matrix | sp|Q9HC38|GLOD4_HUMAN | Glyoxalase domain-containing protein 4 | 18.32 | 18.64 | 18.69 |
| 1340 | GLRX | Non-matrisome | Non-matrisome | GO:CC not extracellular matrix | sp|P35754|GLRX1_HUMAN | Glutaredoxin-1 | 19.95 | 20.54 | 20.74 |
| 1341 | GLRX3 | Non-matrisome | Non-matrisome | GO:CC not extracellular matrix | sp|O76003|GLRX3_HUMAN | Glutaredoxin-3 | 18.84 | 18.22 | 18.19 |
| 1342 | GLS | Non-matrisome | Non-matrisome | GO:CC extracellular matrix (GO:0005576, GO:0005578 & GO:0031012) & GO:CC not extracellular matrix | sp|O94925|GLSK_HUMAN | Glutaminase kidney isoform, mitochondrial | 17.69 | 17.64 | 17.37 |
| 1343 | GLTP | Non-matrisome | Non-matrisome | GO:CC not extracellular matrix | sp|Q9NZD2|GLTP_HUMAN | Glycolipid transfer protein | 19.97 | 19.61 | 19.6 |
| 1344 | GLUD1 | Non-matrisome | Non-matrisome | GO:CC not extracellular matrix | sp|P00367|DHE3_HUMAN | Glutamate dehydrogenase 1, mitochondrial | 18.77 | 19.55 | 19.24 |
| 1345 | GLUL | Non-matrisome | Non-matrisome | GO:CC not extracellular matrix | sp|P15104|GLNA_HUMAN | Glutamine synthetase | 19.52 | 16.77 | 16.49 |
| 1346 | GLYR1 | Non-matrisome | Non-matrisome | GO:CC not extracellular matrix | sp|Q49A26|GLYR1_HUMAN | Cytokine-like nuclear factor N-PAC | 17.61 | 17.92 | 17.85 |
| 1347 | GM2A | Non-matrisome | Non-matrisome | GO:CC extracellular matrix (GO:0005576, GO:0005578 & GO:0031012) & GO:CC not extracellular matrix | sp|P17900|SAP3_HUMAN | Ganglioside GM2 activator | 19.52 | 21.01 | 21.44 |
| 1348 | GMDS | Non-matrisome | Non-matrisome | GO:CC not extracellular matrix | sp|O60547|GMDS_HUMAN | GDP-mannose 4,6 dehydratase | 18.09 | 18.23 | 18.31 |
| 1349 | GMFB | Non-matrisome | Non-matrisome | GO:CC not extracellular matrix | sp|P60983|GMFB_HUMAN | Glia maturation factor beta | 19.16 | 18.97 | 19.02 |
| 1350 | GMFG | Non-matrisome | Non-matrisome | GO:CC extracellular matrix (GO:0005576, GO:0005578 & GO:0031012) & GO:CC not extracellular matrix | sp|O60234|GMFG_HUMAN | Glia maturation factor gamma | 17.29 | 17.14 | 17.24 |
| 1351 | GMPPA | Non-matrisome | Non-matrisome | GO:CC not extracellular matrix | sp|Q96IJ6|GMPPA_HUMAN | Mannose-1-phosphate guanyltransferase alpha | 18.82 | 18.43 | 18.38 |
| 1352 | GMPPB | Non-matrisome | Non-matrisome | GO:CC not extracellular matrix | sp|Q9Y5P6|GMPPB_HUMAN | Mannose-1-phosphate guanyltransferase beta | 19.06 | 18.74 | 18.53 |
| 1353 | GMPR2 | Non-matrisome | Non-matrisome | GO:CC not extracellular matrix | sp|Q9P2T1|GMPR2_HUMAN | GMP reductase 2 | 18.53 | 18.7 | 18.63 |
| 1354 | GMPS | Non-matrisome | Non-matrisome | GO:CC not extracellular matrix | sp|P49915|GUAA_HUMAN | GMP synthase [glutamine-hydrolyzing] | 18.74 | 18.41 | 18.47 |
| 1355 | GNA11 | Non-matrisome | Non-matrisome | GO:CC not extracellular matrix | sp|P29992|GNA11_HUMAN | Guanine nucleotide-binding protein subunit alpha-11 | 19.16 | 19.51 | 19.53 |
| 1356 | GNA13 | Non-matrisome | Non-matrisome | GO:CC not extracellular matrix | sp|Q14344|GNA13_HUMAN | Guanine nucleotide-binding protein subunit alpha-13 | 18.42 | 18.89 | 19 |
| 1357 | GNAI1 | Non-matrisome | Non-matrisome | GO:CC not extracellular matrix | sp|P63096|GNAI1_HUMAN | Guanine nucleotide-binding protein G(i) subunit alpha-1 | 16.47 | 17.11 | 16.87 |
| 1358 | GNAI2 | Non-matrisome | Non-matrisome | GO:CC not extracellular matrix | sp|P04899|GNAI2_HUMAN | Guanine nucleotide-binding protein G(i) subunit alpha-2 | 18.5 | 18.72 | 18.75 |
| 1359 | GNAI3 | Non-matrisome | Non-matrisome | GO:CC not extracellular matrix | sp|P08754|GNAI3_HUMAN | Guanine nucleotide-binding protein G(i) subunit alpha-3 | 17.93 | 17.85 | 17.6 |
| 1360 | GNAQ | Non-matrisome | Non-matrisome | GO:CC not extracellular matrix | sp|P50148|GNAQ_HUMAN | Guanine nucleotide-binding protein G(q) subunit alpha | 16.84 | 17.38 | 17.43 |
| 1361 | GNAS | Non-matrisome | Non-matrisome | GO:CC not extracellular matrix | sp|Q5JWF2|GNAS1_HUMAN | Guanine nucleotide-binding protein G(s) subunit alpha isoforms XLas | 18.15 | 18.86 | 18.92 |
| 1362 | GNB1 | Non-matrisome | Non-matrisome | GO:CC not extracellular matrix | sp|P62873|GBB1_HUMAN | Guanine nucleotide-binding protein G(I)/G(S)/G(T) subunit beta-1 | 18.74 | 19.23 | 19.08 |
| 1363 | GNB2 | Non-matrisome | Non-matrisome | GO:CC not extracellular matrix | sp|P62879|GBB2_HUMAN | Guanine nucleotide-binding protein G(I)/G(S)/G(T) subunit beta-2 | 18.77 | 19.17 | 19.02 |
| 1364 | GNB4 | Non-matrisome | Non-matrisome | GO:CC not extracellular matrix | sp|Q9HAV0|GBB4_HUMAN | Guanine nucleotide-binding protein subunit beta-4 | 17.28 | 17.12 | 16.89 |
| 1365 | GNG10 | Non-matrisome | Non-matrisome | GO:CC not extracellular matrix | sp|P50151|GBG10_HUMAN | Guanine nucleotide-binding protein G(I)/G(S)/G(O) subunit gamma-10 | 19.09 | 18.71 | 18.45 |
| 1366 | GNG12 | Non-matrisome | Non-matrisome | GO:CC not extracellular matrix | sp|Q9UBI6|GBG12_HUMAN | Guanine nucleotide-binding protein G(I)/G(S)/G(O) subunit gamma-12 | 17.67 | 18.07 | 18.04 |
| 1367 | GNG2 | Non-matrisome | Non-matrisome | GO:CC not extracellular matrix | sp|P59768|GBG2_HUMAN | Guanine nucleotide-binding protein G(I)/G(S)/G(O) subunit gamma-2 | 17.62 | 18.27 | 18.36 |
| 1368 | GNG5 | Non-matrisome | Non-matrisome | GO:CC not extracellular matrix | sp|P63218|GBG5_HUMAN | Guanine nucleotide-binding protein G(I)/G(S)/G(O) subunit gamma-5 | 22.02 | 22.13 | 21.68 |
| 1369 | GNL1 | Non-matrisome | Non-matrisome | GO:CC not extracellular matrix | sp|P36915|GNL1_HUMAN | Guanine nucleotide-binding protein-like 1 | 17.38 | 17.83 | 17.78 |
| 1370 | GNL3 | Non-matrisome | Non-matrisome | GO:CC not extracellular matrix | sp|Q9BVP2|GNL3_HUMAN | Guanine nucleotide-binding protein-like 3 | 18.19 | 17.86 | 17.78 |
| 1371 | GNPDA1 | Non-matrisome | Non-matrisome | GO:CC not extracellular matrix | sp|P46926|GNPI1_HUMAN | Glucosamine-6-phosphate isomerase 1 | 17.82 | 18.46 | 18.41 |
| 1372 | GNPDA2 | Non-matrisome | Non-matrisome | GO:CC not extracellular matrix | sp|Q8TDQ7|GNPI2_HUMAN | Glucosamine-6-phosphate isomerase 2 | 19.17 | 19.39 | 19.46 |
| 1373 | GNPNAT1 | Non-matrisome | Non-matrisome | GO:CC not extracellular matrix | sp|Q96EK6|GNA1_HUMAN | Glucosamine 6-phosphate N-acetyltransferase | 19.11 | 17.55 | 17.37 |
| 1374 | GNS | Non-matrisome | Non-matrisome | GO:CC extracellular matrix (GO:0005576, GO:0005578 & GO:0031012) & GO:CC not extracellular matrix | sp|P15586|GNS_HUMAN | N-acetylglucosamine-6-sulfatase | 17.21 | 18.44 | 18.65 |
| 1375 | GOLGA2 | Non-matrisome | Non-matrisome | GO:CC not extracellular matrix | sp|Q08379|GOGA2_HUMAN | Golgin subfamily A member 2 | 17.47 | 17.25 | 17.48 |
| 1376 | GOLGA3 | Non-matrisome | Non-matrisome | GO:CC not extracellular matrix | sp|Q08378|GOGA3_HUMAN | Golgin subfamily A member 3 | 17.86 | 17.45 | 17.46 |
| 1377 | GOLGA4 | Non-matrisome | Non-matrisome | GO:CC not extracellular matrix | sp|Q13439|GOGA4_HUMAN | Golgin subfamily A member 4 | 17.58 | 17.19 | 17.45 |
| 1378 | GOLGA5 | Non-matrisome | Non-matrisome | GO:CC not extracellular matrix | sp|Q8TBA6|GOGA5_HUMAN | Golgin subfamily A member 5 | 17.6 | 16.74 | 16.98 |
| 1379 | GOLGA7 | Non-matrisome | Non-matrisome | GO:CC extracellular matrix (GO:0005576, GO:0005578 & GO:0031012) & GO:CC not extracellular matrix | sp|Q7Z5G4|GOGA7_HUMAN | Golgin subfamily A member 7 | 17.48 | 17.51 | 17.6 |
| 1380 | GOLGB1 | Non-matrisome | Non-matrisome | GO:CC not extracellular matrix | sp|Q14789|GOGB1_HUMAN | Golgin subfamily B member 1 | 17.92 | 17.4 | 17.61 |
| 1381 | GOLIM4 | Non-matrisome | Non-matrisome | GO:CC not extracellular matrix | sp|O00461|GOLI4_HUMAN | Golgi integral membrane protein 4 | 18.82 | 18.43 | 18.83 |
| 1382 | GOLPH3 | Non-matrisome | Non-matrisome | GO:CC not extracellular matrix | sp|Q9H4A6|GOLP3_HUMAN | Golgi phosphoprotein 3 | 17.93 | 17.25 | 17.38 |
| 1383 | GOLT1B | Non-matrisome | Non-matrisome | GO:CC not extracellular matrix | sp|Q9Y3E0|GOT1B_HUMAN | Vesicle transport protein GOT1B | 18.83 | 18.84 | 18.79 |
| 1384 | GON7 | Non-matrisome | Non-matrisome | GO:CC not extracellular matrix | sp|Q9BXV9|GON7_HUMAN | EKC/KEOPS complex subunit GON7 | 15.37 | 15.65 | 15.89 |
| 1385 | GOPC | Non-matrisome | Non-matrisome | GO:CC not extracellular matrix | sp|Q9HD26|GOPC_HUMAN | Golgi-associated PDZ and coiled-coil motif-containing protein | 18.46 | 18.15 | 18.17 |
| 1386 | GORASP2 | Non-matrisome | Non-matrisome | GO:CC not extracellular matrix | sp|Q9H8Y8|GORS2_HUMAN | Golgi reassembly-stacking protein 2 | 18.03 | 17.85 | 18 |
| 1387 | GOSR1 | Non-matrisome | Non-matrisome | GO:CC not extracellular matrix | sp|O95249|GOSR1_HUMAN | Golgi SNAP receptor complex member 1 | 18.31 | 17.52 | 17.75 |
| 1388 | GOT1 | Non-matrisome | Non-matrisome | GO:CC not extracellular matrix | sp|P17174|AATC_HUMAN | Aspartate aminotransferase, cytoplasmic | 18.76 | 18.73 | 18.62 |
| 1389 | GOT2 | Non-matrisome | Non-matrisome | GO:CC not extracellular matrix | sp|P00505|AATM_HUMAN | Aspartate aminotransferase, mitochondrial | 19.53 | 19.77 | 19.52 |
| 1390 | GPC1 | Matrisome-associated | ECM-affiliated Proteins | GO:CC extracellular matrix (GO:0005576, GO:0005578 & GO:0031012) & GO:CC not extracellular matrix | sp|P35052|GPC1_HUMAN | Glypican-1 | 17.33 | 18.36 | 18.41 |
| 1391 | GPD1L | Non-matrisome | Non-matrisome | GO:CC not extracellular matrix | sp|Q8N335|GPD1L_HUMAN | Glycerol-3-phosphate dehydrogenase 1-like protein | 17.62 | 18.48 | 18.73 |
| 1392 | GPD2 | Non-matrisome | Non-matrisome | GO:CC not extracellular matrix | sp|P43304|GPDM_HUMAN | Glycerol-3-phosphate dehydrogenase, mitochondrial | 17.89 | 18.77 | 18.43 |
| 1393 | GPHN | Non-matrisome | Non-matrisome | GO:CC not extracellular matrix | sp|Q9NQX3|GEPH_HUMAN | Gephyrin | 17.57 | 17 | 16.96 |
| 1394 | GPI | Non-matrisome | Non-matrisome | GO:CC extracellular matrix (GO:0005576, GO:0005578 & GO:0031012) & GO:CC not extracellular matrix | sp|P06744|G6PI_HUMAN | Glucose-6-phosphate isomerase | 18.97 | 19.29 | 19.05 |
| 1395 | GPN1 | Non-matrisome | Non-matrisome | GO:CC not extracellular matrix | sp|Q9HCN4|GPN1_HUMAN | GPN-loop GTPase 1 | 16.65 | 16.34 | 16.18 |
| 1396 | GPNMB | Non-matrisome | Non-matrisome | GO:CC not extracellular matrix | sp|Q14956|GPNMB_HUMAN | Transmembrane glycoprotein NMB | 13.9 | 15.71 | 15.55 |
| 1397 | GPS1 | Non-matrisome | Non-matrisome | GO:CC not extracellular matrix | sp|Q13098|CSN1_HUMAN | COP9 signalosome complex subunit 1 | 18.61 | 18.61 | 18.55 |
| 1398 | GPX1 | Non-matrisome | Non-matrisome | GO:CC not extracellular matrix | sp|P07203|GPX1_HUMAN | Glutathione peroxidase 1 | 18.73 | 19.45 | 19.29 |
| 1399 | GPX3 | Non-matrisome | Non-matrisome | GO:CC extracellular matrix (GO:0005576, GO:0005578 & GO:0031012) & GO:CC not extracellular matrix | sp|P22352|GPX3_HUMAN | Glutathione peroxidase 3 | 14.67 | 16.84 | 17.42 |
| 1400 | GPX4 | Non-matrisome | Non-matrisome | GO:CC not extracellular matrix | sp|P36969|GPX4_HUMAN | Phospholipid hydroperoxide glutathione peroxidase | 19.35 | 19.77 | 19.7 |
| 1401 | GPX8 | Non-matrisome | Non-matrisome | GO:CC not extracellular matrix | sp|Q8TED1|GPX8_HUMAN | Probable glutathione peroxidase 8 | 19.03 | 18.99 | 18.95 |
| 1402 | GRB2 | Non-matrisome | Non-matrisome | GO:CC not extracellular matrix | sp|P62993|GRB2_HUMAN | Growth factor receptor-bound protein 2 | 19.63 | 19.04 | 19.1 |
| 1403 | GREM1 | Matrisome-associated | ECM-affiliated Proteins | GO:CC not extracellular matrix | sp|O60565|GREM1_HUMAN | Gremlin-1 | 16.65 | 17.73 | 18.17 |
| 1404 | GRHPR | Non-matrisome | Non-matrisome | GO:CC not extracellular matrix | sp|Q9UBQ7|GRHPR_HUMAN | Glyoxylate reductase/hydroxypyruvate reductase | 17.95 | 18.32 | 18.33 |
| 1405 | GRIPAP1 | Non-matrisome | Non-matrisome | GO:CC not extracellular matrix | sp|Q4V328|GRAP1_HUMAN | GRIP1-associated protein 1 | 16.83 | 16.97 | 17.03 |
| 1406 | GRK2 | Non-matrisome | Non-matrisome | GO:CC not extracellular matrix | sp|P25098|ARBK1_HUMAN | Beta-adrenergic receptor kinase 1 | 17.61 | 17.39 | 17.5 |
| 1407 | GRN | Non-matrisome | Non-matrisome | GO:CC extracellular matrix (GO:0005576, GO:0005578 & GO:0031012) & GO:CC not extracellular matrix | sp|P28799|GRN_HUMAN | Progranulin | 16.86 | 17.7 | 17.98 |
| 1408 | GRPEL1 | Non-matrisome | Non-matrisome | GO:CC not extracellular matrix | sp|Q9HAV7|GRPE1_HUMAN | GrpE protein homolog 1, mitochondrial | 18.94 | 18.82 | 18.46 |
| 1409 | GRSF1 | Non-matrisome | Non-matrisome | GO:CC not extracellular matrix | sp|Q12849|GRSF1_HUMAN | G-rich sequence factor 1 | 17.82 | 16.54 | 16.69 |
| 1410 | GRWD1 | Non-matrisome | Non-matrisome | GO:CC not extracellular matrix | sp|Q9BQ67|GRWD1_HUMAN | Glutamate-rich WD repeat-containing protein 1 | 18.05 | 17.2 | 17.18 |
| 1411 | GSDMD | Non-matrisome | Non-matrisome | GO:CC extracellular matrix (GO:0005576, GO:0005578 & GO:0031012) & GO:CC not extracellular matrix | sp|P57764|GSDMD_HUMAN | Gasdermin-D | 18.59 | 19.45 | 19.48 |
| 1412 | GSK3A | Non-matrisome | Non-matrisome | GO:CC not extracellular matrix | sp|P49840|GSK3A_HUMAN | Glycogen synthase kinase-3 alpha | 17.41 | 17.28 | 17.32 |
| 1413 | GSK3B | Non-matrisome | Non-matrisome | GO:CC not extracellular matrix | sp|P49841|GSK3B_HUMAN | Glycogen synthase kinase-3 beta | 17.72 | 17.48 | 17.57 |
| 1414 | GSN | Non-matrisome | Non-matrisome | GO:CC extracellular matrix (GO:0005576, GO:0005578 & GO:0031012) & GO:CC not extracellular matrix | sp|P06396|GELS_HUMAN | Gelsolin | 18.55 | 19.41 | 19.51 |
| 1415 | GSPT1 | Non-matrisome | Non-matrisome | GO:CC not extracellular matrix | sp|P15170|ERF3A_HUMAN | Eukaryotic peptide chain release factor GTP-binding subunit ERF3A | 19.51 | 18.99 | 18.85 |
| 1416 | GSPT2 | Non-matrisome | Non-matrisome | GO:CC not extracellular matrix | sp|Q8IYD1|ERF3B_HUMAN | Eukaryotic peptide chain release factor GTP-binding subunit ERF3B | 17.74 | 17.82 | 17.91 |
| 1417 | GSR | Non-matrisome | Non-matrisome | GO:CC not extracellular matrix | sp|P00390|GSHR_HUMAN | Glutathione reductase, mitochondrial | 18.85 | 18.67 | 18.66 |
| 1418 | GSS | Non-matrisome | Non-matrisome | GO:CC not extracellular matrix | sp|P48637|GSHB_HUMAN | Glutathione synthetase | 19.91 | 19.97 | 19.91 |
| 1419 | GSTA4 | Non-matrisome | Non-matrisome | GO:CC not extracellular matrix | sp|O15217|GSTA4_HUMAN | Glutathione S-transferase A4 | 18.1 | 18.34 | 18.59 |
| 1420 | GSTK1 | Non-matrisome | Non-matrisome | GO:CC not extracellular matrix | sp|Q9Y2Q3|GSTK1_HUMAN | Glutathione S-transferase kappa 1 | 18.59 | 18.9 | 18.87 |
| 1421 | GSTM1 | Non-matrisome | Non-matrisome | GO:CC not extracellular matrix | sp|P09488|GSTM1_HUMAN | Glutathione S-transferase Mu 1 | 16.13 | 17.02 | 17.43 |
| 1422 | GSTM2 | Non-matrisome | Non-matrisome | GO:CC not extracellular matrix | sp|P28161|GSTM2_HUMAN | Glutathione S-transferase Mu 2 | 16.79 | 17.52 | 17.8 |
| 1423 | GSTM3 | Non-matrisome | Non-matrisome | GO:CC not extracellular matrix | sp|P21266|GSTM3_HUMAN | Glutathione S-transferase Mu 3 | 19.01 | 19.06 | 19.55 |
| 1424 | GSTM4 | Non-matrisome | Non-matrisome | GO:CC not extracellular matrix | sp|Q03013|GSTM4_HUMAN | Glutathione S-transferase Mu 4 | 17.28 | 17.3 | 17.7 |
| 1425 | GSTM5 | Non-matrisome | Non-matrisome | GO:CC not extracellular matrix | sp|P46439|GSTM5_HUMAN | Glutathione S-transferase Mu 5 | 16.03 | 17.55 | 18.12 |
| 1426 | GSTO1 | Non-matrisome | Non-matrisome | GO:CC not extracellular matrix | sp|P78417|GSTO1_HUMAN | Glutathione S-transferase omega-1 | 19.56 | 19.53 | 19.62 |
| 1427 | GSTP1 | Non-matrisome | Non-matrisome | GO:CC extracellular matrix (GO:0005576, GO:0005578 & GO:0031012) & GO:CC not extracellular matrix | sp|P09211|GSTP1_HUMAN | Glutathione S-transferase P | 19.45 | 19.48 | 19.67 |
| 1428 | GSTT1 | Non-matrisome | Non-matrisome | GO:CC not extracellular matrix | sp|P30711|GSTT1_HUMAN | Glutathione S-transferase theta-1 | 14.68 | 15.85 | 15.91 |
| 1429 | GSTZ1 | Non-matrisome | Non-matrisome | GO:CC not extracellular matrix | sp|O43708|MAAI_HUMAN | Maleylacetoacetate isomerase | 18.56 | 18.66 | 18.63 |
| 1430 | GTF2A1 | Non-matrisome | Non-matrisome | GO:CC not extracellular matrix | sp|P52655|TF2AA_HUMAN | Transcription initiation factor IIA subunit 1 | 19.18 | 19.21 | 19.19 |
| 1431 | GTF2A2 | Non-matrisome | Non-matrisome | GO:CC not extracellular matrix | sp|P52657|T2AG_HUMAN | Transcription initiation factor IIA subunit 2 | 19.48 | 19.47 | 19.39 |
| 1432 | GTF2E1 | Non-matrisome | Non-matrisome | GO:CC not extracellular matrix | sp|P29083|T2EA_HUMAN | General transcription factor IIE subunit 1 | 17.31 | 16.94 | 17.24 |
| 1433 | GTF2H4 | Non-matrisome | Non-matrisome | GO:CC not extracellular matrix | sp|Q92759|TF2H4_HUMAN | General transcription factor IIH subunit 4 | 17.15 | 17.52 | 17.54 |
| 1434 | GTF2I | Non-matrisome | Non-matrisome | GO:CC not extracellular matrix | sp|P78347|GTF2I_HUMAN | General transcription factor II-I | 18.49 | 18.37 | 18.57 |
| 1435 | GTF3C1 | Non-matrisome | Non-matrisome | GO:CC not extracellular matrix | sp|Q12789|TF3C1_HUMAN | General transcription factor 3C polypeptide 1 | 17.37 | 17.35 | 17.3 |
| 1436 | GTF3C3 | Non-matrisome | Non-matrisome | GO:CC not extracellular matrix | sp|Q9Y5Q9|TF3C3_HUMAN | General transcription factor 3C polypeptide 3 | 17.03 | 16.94 | 16.94 |
| 1437 | GTF3C4 | Non-matrisome | Non-matrisome | GO:CC not extracellular matrix | sp|Q9UKN8|TF3C4_HUMAN | General transcription factor 3C polypeptide 4 | 16.04 | 15.99 | 15.93 |
| 1438 | GTPBP1 | Non-matrisome | Non-matrisome | GO:CC not extracellular matrix | sp|O00178|GTPB1_HUMAN | GTP-binding protein 1 | 18.24 | 17.95 | 18.02 |
| 1439 | GTPBP4 | Non-matrisome | Non-matrisome | GO:CC not extracellular matrix | sp|Q9BZE4|GTPB4_HUMAN | GTP-binding protein 4 | 18.08 | 17.07 | 16.92 |
| 1440 | GUK1 | Non-matrisome | Non-matrisome | GO:CC not extracellular matrix | sp|Q16774|KGUA_HUMAN | Guanylate kinase | 17.71 | 18.16 | 18.24 |
| 1441 | GUSB | Non-matrisome | Non-matrisome | GO:CC extracellular matrix (GO:0005576, GO:0005578 & GO:0031012) & GO:CC not extracellular matrix | sp|P08236|BGLR_HUMAN | Beta-glucuronidase | 17.06 | 18.16 | 18.44 |
| 1442 | GYG1 | Non-matrisome | Non-matrisome | GO:CC extracellular matrix (GO:0005576, GO:0005578 & GO:0031012) & GO:CC not extracellular matrix | sp|P46976|GLYG_HUMAN | Glycogenin-1 | 17.4 | 17.44 | 17.38 |
| 1443 | GYS1 | Non-matrisome | Non-matrisome | GO:CC not extracellular matrix | sp|P13807|GYS1_HUMAN | Glycogen [starch] synthase, muscle | 18.53 | 19.06 | 18.27 |
| 1444 | H1-0 | Non-matrisome | Non-matrisome | GO:CC not extracellular matrix | sp|P07305|H10_HUMAN | Histone H1.0 | 18.61 | 20.5 | 20.49 |
| 1445 | H1-1 | Non-matrisome | Non-matrisome | GO:CC not extracellular matrix | sp|Q02539|H11_HUMAN | Histone H1.1 | 20.03 | 19.61 | 19.51 |
| 1446 | H1-10 | Non-matrisome | Non-matrisome | GO:CC not extracellular matrix | sp|Q92522|H1X_HUMAN | Histone H1.10 | 17.78 | 19.45 | 19.08 |
| 1447 | H1-2 | Non-matrisome | Non-matrisome | GO:CC not extracellular matrix | sp|P16403|H12_HUMAN | Histone H1.2 | 19.21 | 20.24 | 19.61 |
| 1448 | H1-3 | Non-matrisome | Non-matrisome | GO:CC not extracellular matrix | sp|P16402|H13_HUMAN | Histone H1.3 | 19.4 | 20.54 | 19.99 |
| 1449 | H1-4 | Non-matrisome | Non-matrisome | GO:CC not extracellular matrix | sp|P10412|H14_HUMAN | Histone H1.4 | 20.32 | 21.58 | 20.89 |
| 1450 | H1-5 | Non-matrisome | Non-matrisome | GO:CC not extracellular matrix | sp|P16401|H15_HUMAN | Histone H1.5 | 20.03 | 20.69 | 20.38 |
| 1451 | H2AC21 | Non-matrisome | Non-matrisome | GO:CC not extracellular matrix | sp|Q8IUE6|H2A2B_HUMAN | Histone H2A type 2-B | 18.02 | 18.36 | 18.08 |
| 1452 | H2AX | Non-matrisome | Non-matrisome | GO:CC not extracellular matrix | sp|P16104|H2AX_HUMAN | Histone H2AX | 21.64 | 22.63 | 22.4 |
| 1453 | H2BC1 | Non-matrisome | Non-matrisome | GO:CC not extracellular matrix | sp|Q96A08|H2B1A_HUMAN | Histone H2B type 1-A | 19.34 | 19.58 | 19.44 |
| 1454 | H6PD | Non-matrisome | Non-matrisome | GO:CC not extracellular matrix | sp|O95479|G6PE_HUMAN | GDH/6PGL endoplasmic bifunctional protein | 16.95 | 17.95 | 17.83 |
| 1455 | HAAO | Non-matrisome | Non-matrisome | GO:CC not extracellular matrix | sp|P46952|3HAO_HUMAN | 3-hydroxyanthranilate 3,4-dioxygenase | 16.45 | 16.95 | 16.93 |
| 1456 | HABP2 | Matrisome-associated | ECM Regulators | GO:CC extracellular matrix (GO:0005576, GO:0005578 & GO:0031012) & GO:CC not extracellular matrix | sp|Q14520|HABP2_HUMAN | Hyaluronan-binding protein 2 | 18.42 | 20.73 | 21.04 |
| 1457 | HACD2 | Non-matrisome | Non-matrisome | GO:CC not extracellular matrix | sp|Q6Y1H2|HACD2_HUMAN | Very-long-chain (3R)-3-hydroxyacyl-CoA dehydratase 2 | 20.02 | 20.25 | 19.43 |
| 1458 | HACD3 | Non-matrisome | Non-matrisome | GO:CC not extracellular matrix | sp|Q9P035|HACD3_HUMAN | Very-long-chain (3R)-3-hydroxyacyl-CoA dehydratase 3 | 18.59 | 18.84 | 18.69 |
| 1459 | HADH | Non-matrisome | Non-matrisome | GO:CC not extracellular matrix | sp|Q16836|HCDH_HUMAN | Hydroxyacyl-coenzyme A dehydrogenase, mitochondrial | 19.6 | 20.42 | 19.97 |
| 1460 | HADHA | Non-matrisome | Non-matrisome | GO:CC not extracellular matrix | sp|P40939|ECHA_HUMAN | Trifunctional enzyme subunit alpha, mitochondrial | 18.69 | 19.74 | 19.57 |
| 1461 | HADHB | Non-matrisome | Non-matrisome | GO:CC not extracellular matrix | sp|P55084|ECHB_HUMAN | Trifunctional enzyme subunit beta, mitochondrial | 17.5 | 18.49 | 18.44 |
| 1462 | HAGH | Non-matrisome | Non-matrisome | GO:CC not extracellular matrix | sp|Q16775|GLO2_HUMAN | Hydroxyacylglutathione hydrolase, mitochondrial | 18.19 | 18.77 | 19.07 |
| 1463 | HAPLN1 | Core matrisome | Proteoglycans | GO:CC extracellular matrix (GO:0005576, GO:0005578 & GO:0031012) & GO:CC not extracellular matrix | sp|P10915|HPLN1_HUMAN | Hyaluronan and proteoglycan link protein 1 | 18.75 | 18.19 | 17.6 |
| 1464 | HARS1 | Non-matrisome | Non-matrisome | GO:CC not extracellular matrix | sp|P12081|HARS1_HUMAN | Histidine--tRNA ligase, cytoplasmic | 19.78 | 19.4 | 19.26 |
| 1465 | HAT1 | Non-matrisome | Non-matrisome | GO:CC not extracellular matrix | sp|O14929|HAT1_HUMAN | Histone acetyltransferase type B catalytic subunit | 18.37 | 16.88 | 17.21 |
| 1466 | HCFC1 | Matrisome-associated | Secreted Factors | GO:CC not extracellular matrix | sp|P51610|HCFC1_HUMAN | Host cell factor 1 | 17.64 | 17.71 | 17.69 |
| 1467 | HDAC1 | Non-matrisome | Non-matrisome | GO:CC extracellular matrix (GO:0005576, GO:0005578 & GO:0031012) & GO:CC not extracellular matrix | sp|Q13547|HDAC1_HUMAN | Histone deacetylase 1 | 17.57 | 17.8 | 17.91 |
| 1468 | HDAC2 | Non-matrisome | Non-matrisome | GO:CC not extracellular matrix | sp|Q92769|HDAC2_HUMAN | Histone deacetylase 2 | 17.78 | 17.41 | 17.37 |
| 1469 | HDAC6 | Non-matrisome | Non-matrisome | GO:CC extracellular matrix (GO:0005576, GO:0005578 & GO:0031012) & GO:CC not extracellular matrix | sp|Q9UBN7|HDAC6_HUMAN | Histone deacetylase 6 | 17.57 | 17.98 | 18.02 |
| 1470 | HDDC2 | Non-matrisome | Non-matrisome | GO:CC not extracellular matrix | sp|Q7Z4H3|HDDC2_HUMAN | 5'-deoxynucleotidase HDDC2 | 16.87 | 17.2 | 17.22 |
| 1471 | HDDC3 | Non-matrisome | Non-matrisome | GO:CC not extracellular matrix | sp|Q8N4P3|MESH1_HUMAN | Guanosine-3',5'-bis(diphosphate) 3'-pyrophosphohydrolase MESH1 | 16.94 | 17.81 | 17.69 |
| 1472 | HDGF | Non-matrisome | Non-matrisome | GO:CC extracellular matrix (GO:0005576, GO:0005578 & GO:0031012) & GO:CC not extracellular matrix | sp|P51858|HDGF_HUMAN | Hepatoma-derived growth factor | 17.43 | 17.7 | 17.86 |
| 1473 | HDGFL2 | Non-matrisome | Non-matrisome | GO:CC not extracellular matrix | sp|Q7Z4V5|HDGR2_HUMAN | Hepatoma-derived growth factor-related protein 2 | 17.25 | 17.47 | 17.52 |
| 1474 | HDGFL3 | Non-matrisome | Non-matrisome | GO:CC extracellular matrix (GO:0005576, GO:0005578 & GO:0031012) & GO:CC not extracellular matrix | sp|Q9Y3E1|HDGR3_HUMAN | Hepatoma-derived growth factor-related protein 3 | 17.99 | 18.88 | 19.03 |
| 1475 | HDHD2 | Non-matrisome | Non-matrisome | GO:CC not extracellular matrix | sp|Q9H0R4|HDHD2_HUMAN | Haloacid dehalogenase-like hydrolase domain-containing protein 2 | 17.94 | 17.88 | 17.89 |
| 1476 | HDHD3 | Non-matrisome | Non-matrisome | GO:CC not extracellular matrix | sp|Q9BSH5|HDHD3_HUMAN | Haloacid dehalogenase-like hydrolase domain-containing protein 3 | 16.09 | 17.14 | 17.15 |
| 1477 | HDHD5 | Non-matrisome | Non-matrisome | GO:CC not extracellular matrix | sp|Q9BXW7|HDHD5_HUMAN | Haloacid dehalogenase-like hydrolase domain-containing 5 | 16.73 | 17.37 | 16.92 |
| 1478 | HDLBP | Non-matrisome | Non-matrisome | GO:CC not extracellular matrix | sp|Q00341|VIGLN_HUMAN | Vigilin | 19.65 | 18.86 | 19.05 |
| 1479 | HEATR1 | Non-matrisome | Non-matrisome | GO:CC not extracellular matrix | sp|Q9H583|HEAT1_HUMAN | HEAT repeat-containing protein 1 | 17.39 | 16.87 | 16.72 |
| 1480 | HEATR3 | Non-matrisome | Non-matrisome | GO:CC not extracellular matrix | sp|Q7Z4Q2|HEAT3_HUMAN | HEAT repeat-containing protein 3 | 17.66 | 16.94 | 16.99 |
| 1481 | HEBP1 | Non-matrisome | Non-matrisome | GO:CC extracellular matrix (GO:0005576, GO:0005578 & GO:0031012) & GO:CC not extracellular matrix | sp|Q9NRV9|HEBP1_HUMAN | Heme-binding protein 1 | 17.79 | 18.15 | 18.33 |
| 1482 | HEBP2 | Non-matrisome | Non-matrisome | GO:CC extracellular matrix (GO:0005576, GO:0005578 & GO:0031012) & GO:CC not extracellular matrix | sp|Q9Y5Z4|HEBP2_HUMAN | Heme-binding protein 2 | 16.69 | 18.13 | 18.24 |
| 1483 | HECTD1 | Non-matrisome | Non-matrisome | GO:CC not extracellular matrix | sp|Q9ULT8|HECD1_HUMAN | E3 ubiquitin-protein ligase HECTD1 | 17.79 | 17.37 | 17.52 |
| 1484 | HECTD3 | Non-matrisome | Non-matrisome | GO:CC not extracellular matrix | sp|Q5T447|HECD3_HUMAN | E3 ubiquitin-protein ligase HECTD3 | 16.85 | 16.63 | 16.78 |
| 1485 | HERC1 | Non-matrisome | Non-matrisome | GO:CC not extracellular matrix | sp|Q15751|HERC1_HUMAN | Probable E3 ubiquitin-protein ligase HERC1 | 16.59 | 16.27 | 16.38 |
| 1486 | HERC4 | Non-matrisome | Non-matrisome | GO:CC not extracellular matrix | sp|Q5GLZ8|HERC4_HUMAN | Probable E3 ubiquitin-protein ligase HERC4 | 17.64 | 17.51 | 17.61 |
| 1487 | HERPUD1 | Non-matrisome | Non-matrisome | GO:CC not extracellular matrix | sp|Q15011|HERP1_HUMAN | Homocysteine-responsive endoplasmic reticulum-resident ubiquitin-like domain member 1 protein | 16.77 | 15.39 | 15.77 |
| 1488 | HEXA | Non-matrisome | Non-matrisome | GO:CC not extracellular matrix | sp|P06865|HEXA_HUMAN | Beta-hexosaminidase subunit alpha | 18.15 | 19.53 | 20.08 |
| 1489 | HEXB | Non-matrisome | Non-matrisome | GO:CC extracellular matrix (GO:0005576, GO:0005578 & GO:0031012) & GO:CC not extracellular matrix | sp|P07686|HEXB_HUMAN | Beta-hexosaminidase subunit beta | 17.47 | 18.67 | 19.34 |
| 1490 | HGH1 | Non-matrisome | Non-matrisome | GO:CC not extracellular matrix | sp|Q9BTY7|HGH1_HUMAN | Protein HGH1 homolog | 17.92 | 17.1 | 17.3 |
| 1491 | HGS | Non-matrisome | Non-matrisome | GO:CC not extracellular matrix | sp|O14964|HGS_HUMAN | Hepatocyte growth factor-regulated tyrosine kinase substrate | 19.18 | 18.98 | 19.01 |
| 1492 | HIBADH | Non-matrisome | Non-matrisome | GO:CC not extracellular matrix | sp|P31937|3HIDH_HUMAN | 3-hydroxyisobutyrate dehydrogenase, mitochondrial | 16.46 | 17.84 | 17.66 |
| 1493 | HIBCH | Non-matrisome | Non-matrisome | GO:CC not extracellular matrix | sp|Q6NVY1|HIBCH_HUMAN | 3-hydroxyisobutyryl-CoA hydrolase, mitochondrial | 17.67 | 18.48 | 18.41 |
| 1494 | HIF1AN | Non-matrisome | Non-matrisome | GO:CC not extracellular matrix | sp|Q9NWT6|HIF1N_HUMAN | Hypoxia-inducible factor 1-alpha inhibitor | 17.89 | 17.46 | 17.48 |
| 1495 | HIGD1A | Non-matrisome | Non-matrisome | GO:CC not extracellular matrix | sp|Q9Y241|HIG1A_HUMAN | HIG1 domain family member 1A, mitochondrial | 18.17 | 17.55 | 17.86 |
| 1496 | HIKESHI | Non-matrisome | Non-matrisome | GO:CC not extracellular matrix | sp|Q53FT3|HIKES_HUMAN | Protein Hikeshi | 16.39 | 16.48 | 16.45 |
| 1497 | HINT1 | Non-matrisome | Non-matrisome | GO:CC extracellular matrix (GO:0005576, GO:0005578 & GO:0031012) & GO:CC not extracellular matrix | sp|P49773|HINT1_HUMAN | Adenosine 5'-monophosphoramidase HINT1 | 19.98 | 19.93 | 19.96 |
| 1498 | HINT2 | Non-matrisome | Non-matrisome | GO:CC not extracellular matrix | sp|Q9BX68|HINT2_HUMAN | Adenosine 5'-monophosphoramidase HINT2 | 19.89 | 19.93 | 19.84 |
| 1499 | HINT3 | Non-matrisome | Non-matrisome | GO:CC not extracellular matrix | sp|Q9NQE9|HINT3_HUMAN | Adenosine 5'-monophosphoramidase HINT3 | 16.54 | 17.66 | 17.65 |
| 1500 | HIP1 | Non-matrisome | Non-matrisome | GO:CC not extracellular matrix | sp|O00291|HIP1_HUMAN | Huntingtin-interacting protein 1 | 18.39 | 18.04 | 17.87 |
| 1501 | HIP1R | Non-matrisome | Non-matrisome | GO:CC not extracellular matrix | sp|O75146|HIP1R_HUMAN | Huntingtin-interacting protein 1-related protein | 15.93 | 15.24 | 15.53 |
| 1502 | HK1 | Non-matrisome | Non-matrisome | GO:CC not extracellular matrix | sp|P19367|HXK1_HUMAN | Hexokinase-1 | 19.22 | 19.92 | 19.69 |
| 1503 | HK2 | Non-matrisome | Non-matrisome | GO:CC not extracellular matrix | sp|P52789|HXK2_HUMAN | Hexokinase-2 | 18.52 | 19.58 | 18.32 |
| 1504 | HLA-A | Non-matrisome | Non-matrisome | GO:CC not extracellular matrix | sp|P04439|HLAA_HUMAN | HLA class I histocompatibility antigen, A alpha chain | 16.4 | 18.34 | 18.05 |
| 1505 | HLA-B | Non-matrisome | Non-matrisome | GO:CC not extracellular matrix | sp|P01889|HLAB_HUMAN | HLA class I histocompatibility antigen, B alpha chain | 16.34 | 16.43 | 16.24 |
| 1506 | HLA-C | Non-matrisome | Non-matrisome | GO:CC not extracellular matrix | sp|P10321|HLAC_HUMAN | HLA class I histocompatibility antigen, C alpha chain | 17.12 | 17.2 | 17.15 |
| 1507 | HLA-F | Non-matrisome | Non-matrisome | GO:CC not extracellular matrix | sp|P30511|HLAF_HUMAN | HLA class I histocompatibility antigen, alpha chain F | 14.81 | 16 | 15.64 |
| 1508 | HLA-H | Non-matrisome | Non-matrisome | GO:CC not extracellular matrix | sp|P01893|HLAH_HUMAN | Putative HLA class I histocompatibility antigen, alpha chain H | 17.8 | 18.73 | 18.63 |
| 1509 | HM13 | Non-matrisome | Non-matrisome | GO:CC not extracellular matrix | sp|Q8TCT9|HM13_HUMAN | Minor histocompatibility antigen H13 | 18.47 | 18.03 | 18.12 |
| 1510 | HMCN1 | Core matrisome | ECM Glycoproteins | GO:CC not extracellular matrix | sp|Q96RW7|HMCN1_HUMAN | Hemicentin-1 | 13.74 | 17.41 | 17.67 |
| 1511 | HMGA1 | Non-matrisome | Non-matrisome | GO:CC not extracellular matrix | sp|P17096|HMGA1_HUMAN | High mobility group protein HMG-I/HMG-Y | 16.81 | 17.89 | 17.9 |
| 1512 | HMGB1 | Non-matrisome | Non-matrisome | GO:CC extracellular matrix (GO:0005576, GO:0005578 & GO:0031012) & GO:CC not extracellular matrix | sp|P09429|HMGB1_HUMAN | High mobility group protein B1 | 17.75 | 17.95 | 18.13 |
| 1513 | HMGB2 | Non-matrisome | Non-matrisome | GO:CC not extracellular matrix | sp|P26583|HMGB2_HUMAN | High mobility group protein B2 | 20.46 | 20.77 | 20.23 |
| 1514 | HMGB3 | Non-matrisome | Non-matrisome | GO:CC not extracellular matrix | sp|O15347|HMGB3_HUMAN | High mobility group protein B3 | 17.71 | 17.6 | 17.3 |
| 1515 | HMGCL | Non-matrisome | Non-matrisome | GO:CC not extracellular matrix | sp|P35914|HMGCL_HUMAN | Hydroxymethylglutaryl-CoA lyase, mitochondrial | 18.13 | 19.2 | 18.84 |
| 1516 | HMGCS1 | Non-matrisome | Non-matrisome | GO:CC not extracellular matrix | sp|Q01581|HMCS1_HUMAN | Hydroxymethylglutaryl-CoA synthase, cytoplasmic | 19.46 | 16.77 | 17.08 |
| 1517 | HMGN1 | Non-matrisome | Non-matrisome | GO:CC not extracellular matrix | sp|P05114|HMGN1_HUMAN | Non-histone chromosomal protein HMG-14 | 17.88 | 17.74 | 18.22 |
| 1518 | HMGN2 | Non-matrisome | Non-matrisome | GO:CC not extracellular matrix | sp|P05204|HMGN2_HUMAN | Non-histone chromosomal protein HMG-17 | 19.33 | 19.37 | 18.74 |
| 1519 | HMGN4 | Non-matrisome | Non-matrisome | GO:CC not extracellular matrix | sp|O00479|HMGN4_HUMAN | High mobility group nucleosome-binding domain-containing protein 4 | 16.42 | 17.52 | 17.41 |
| 1520 | HMGN5 | Non-matrisome | Non-matrisome | GO:CC not extracellular matrix | sp|P82970|HMGN5_HUMAN | High mobility group nucleosome-binding domain-containing protein 5 | 15.94 | 16.45 | 16.7 |
| 1521 | HMOX1 | Non-matrisome | Non-matrisome | GO:CC not extracellular matrix | sp|P09601|HMOX1_HUMAN | Heme oxygenase 1 | 18.92 | 17.7 | 18.58 |
| 1522 | HMOX2 | Non-matrisome | Non-matrisome | GO:CC not extracellular matrix | sp|P30519|HMOX2_HUMAN | Heme oxygenase 2 | 18.04 | 17.64 | 17.55 |
| 1523 | HNRNPA0 | Non-matrisome | Non-matrisome | GO:CC not extracellular matrix | sp|Q13151|ROA0_HUMAN | Heterogeneous nuclear ribonucleoprotein A0 | 19.62 | 19.39 | 19.54 |
| 1524 | HNRNPA1 | Non-matrisome | Non-matrisome | GO:CC not extracellular matrix | sp|P09651|ROA1_HUMAN | Heterogeneous nuclear ribonucleoprotein A1 | 16.14 | 15.74 | 15.68 |
| 1525 | HNRNPA2B1 | Non-matrisome | Non-matrisome | GO:CC not extracellular matrix | sp|P22626|ROA2_HUMAN | Heterogeneous nuclear ribonucleoproteins A2/B1 | 20.02 | 20.2 | 20.35 |
| 1526 | HNRNPA3 | Non-matrisome | Non-matrisome | GO:CC not extracellular matrix | sp|P51991|ROA3_HUMAN | Heterogeneous nuclear ribonucleoprotein A3 | 18.11 | 18.21 | 18.47 |
| 1527 | HNRNPAB | Non-matrisome | Non-matrisome | GO:CC not extracellular matrix | sp|Q99729|ROAA_HUMAN | Heterogeneous nuclear ribonucleoprotein A/B | 20.4 | 19.97 | 19.82 |
| 1528 | HNRNPC | Non-matrisome | Non-matrisome | GO:CC extracellular matrix (GO:0005576, GO:0005578 & GO:0031012) & GO:CC not extracellular matrix | sp|P07910|HNRPC_HUMAN | Heterogeneous nuclear ribonucleoproteins C1/C2 | 16.53 | 16.89 | 16.51 |
| 1529 | HNRNPD | Non-matrisome | Non-matrisome | GO:CC not extracellular matrix | sp|Q14103|HNRPD_HUMAN | Heterogeneous nuclear ribonucleoprotein D0 | 19.33 | 19.63 | 19.64 |
| 1530 | HNRNPDL | Non-matrisome | Non-matrisome | GO:CC not extracellular matrix | sp|O14979|HNRDL_HUMAN | Heterogeneous nuclear ribonucleoprotein D-like | 18.12 | 17.87 | 17.85 |
| 1531 | HNRNPF | Non-matrisome | Non-matrisome | GO:CC not extracellular matrix | sp|P52597|HNRPF_HUMAN | Heterogeneous nuclear ribonucleoprotein F | 18.45 | 18.24 | 18.29 |
| 1532 | HNRNPH1 | Non-matrisome | Non-matrisome | GO:CC not extracellular matrix | sp|P31943|HNRH1_HUMAN | Heterogeneous nuclear ribonucleoprotein H | 18.27 | 18.1 | 18.09 |
| 1533 | HNRNPH2 | Non-matrisome | Non-matrisome | GO:CC not extracellular matrix | sp|P55795|HNRH2_HUMAN | Heterogeneous nuclear ribonucleoprotein H2 | 16.5 | 17.38 | 17.63 |
| 1534 | HNRNPH3 | Non-matrisome | Non-matrisome | GO:CC not extracellular matrix | sp|P31942|HNRH3_HUMAN | Heterogeneous nuclear ribonucleoprotein H3 | 15.94 | 16.01 | 15.94 |
| 1535 | HNRNPK | Non-matrisome | Non-matrisome | GO:CC not extracellular matrix | sp|P61978|HNRPK_HUMAN | Heterogeneous nuclear ribonucleoprotein K | 18.98 | 18.95 | 18.93 |
| 1536 | HNRNPL | Non-matrisome | Non-matrisome | GO:CC not extracellular matrix | sp|P14866|HNRPL_HUMAN | Heterogeneous nuclear ribonucleoprotein L | 18.87 | 19.13 | 19.05 |
| 1537 | HNRNPM | Non-matrisome | Non-matrisome | GO:CC not extracellular matrix | sp|P52272|HNRPM_HUMAN | Heterogeneous nuclear ribonucleoprotein M | 19.71 | 19.9 | 19.94 |
| 1538 | HNRNPR | Non-matrisome | Non-matrisome | GO:CC not extracellular matrix | sp|O43390|HNRPR_HUMAN | Heterogeneous nuclear ribonucleoprotein R | 19.49 | 19.53 | 19.56 |
| 1539 | HNRNPU | Non-matrisome | Non-matrisome | GO:CC not extracellular matrix | sp|Q00839|HNRPU_HUMAN | Heterogeneous nuclear ribonucleoprotein U | 18.87 | 18.88 | 18.78 |
| 1540 | HNRNPUL1 | Non-matrisome | Non-matrisome | GO:CC not extracellular matrix | sp|Q9BUJ2|HNRL1_HUMAN | Heterogeneous nuclear ribonucleoprotein U-like protein 1 | 18.07 | 18.6 | 18.6 |
| 1541 | HNRNPUL2 | Non-matrisome | Non-matrisome | GO:CC not extracellular matrix | sp|Q1KMD3|HNRL2_HUMAN | Heterogeneous nuclear ribonucleoprotein U-like protein 2 | 17.75 | 18.39 | 18.38 |
| 1542 | HOOK3 | Non-matrisome | Non-matrisome | GO:CC not extracellular matrix | sp|Q86VS8|HOOK3_HUMAN | Protein Hook homolog 3 | 18.12 | 17.68 | 17.72 |
| 1543 | HP1BP3 | Non-matrisome | Non-matrisome | GO:CC not extracellular matrix | sp|Q5SSJ5|HP1B3_HUMAN | Heterochromatin protein 1-binding protein 3 | 19.02 | 20.21 | 20.24 |
| 1544 | HPCAL1 | Non-matrisome | Non-matrisome | GO:CC not extracellular matrix | sp|P37235|HPCL1_HUMAN | Hippocalcin-like protein 1 | 18.08 | 18.41 | 19.1 |
| 1545 | HPF1 | Non-matrisome | Non-matrisome | GO:CC not extracellular matrix | sp|Q9NWY4|HPF1_HUMAN | Histone PARylation factor 1 | 18.29 | 18.04 | 18.18 |
| 1546 | HPGD | Non-matrisome | Non-matrisome | GO:CC not extracellular matrix | sp|P15428|PGDH_HUMAN | 15-hydroxyprostaglandin dehydrogenase [NAD(+)] | 14.57 | 15.99 | 16.91 |
| 1547 | HPRT1 | Non-matrisome | Non-matrisome | GO:CC not extracellular matrix | sp|P00492|HPRT_HUMAN | Hypoxanthine-guanine phosphoribosyltransferase | 19.3 | 18.98 | 18.73 |
| 1548 | HPS3 | Non-matrisome | Non-matrisome | GO:CC not extracellular matrix | sp|Q969F9|HPS3_HUMAN | BLOC-2 complex member HPS3 | 16.45 | 16.55 | 16.49 |
| 1549 | HRAS | Non-matrisome | Non-matrisome | GO:CC not extracellular matrix | sp|P01112|RASH_HUMAN | GTPase HRas | 19.26 | 19.52 | 19.41 |
| 1550 | HRNR | Matrisome-associated | Secreted Factors | GO:CC extracellular matrix (GO:0005576, GO:0005578 & GO:0031012) & GO:CC not extracellular matrix | sp|Q86YZ3|HORN_HUMAN | Hornerin | 13.59 | 12.46 | 14.05 |
| 1551 | HS1BP3 | Non-matrisome | Non-matrisome | GO:CC not extracellular matrix | sp|Q53T59|H1BP3_HUMAN | HCLS1-binding protein 3 | 18.14 | 18.8 | 19.08 |
| 1552 | HSD17B10 | Non-matrisome | Non-matrisome | GO:CC not extracellular matrix | sp|Q99714|HCD2_HUMAN | 3-hydroxyacyl-CoA dehydrogenase type-2 | 18.19 | 18.27 | 18.18 |
| 1553 | HSD17B11 | Non-matrisome | Non-matrisome | GO:CC not extracellular matrix | sp|Q8NBQ5|DHB11_HUMAN | Estradiol 17-beta-dehydrogenase 11 | 17.38 | 18.11 | 17.95 |
| 1554 | HSD17B12 | Non-matrisome | Non-matrisome | GO:CC extracellular matrix (GO:0005576, GO:0005578 & GO:0031012) & GO:CC not extracellular matrix | sp|Q53GQ0|DHB12_HUMAN | Very-long-chain 3-oxoacyl-CoA reductase | 18.96 | 19.01 | 18.98 |
| 1555 | HSD17B4 | Non-matrisome | Non-matrisome | GO:CC not extracellular matrix | sp|P51659|DHB4_HUMAN | Peroxisomal multifunctional enzyme type 2 | 18.21 | 18.09 | 18.11 |
| 1556 | HSD17B8 | Non-matrisome | Non-matrisome | GO:CC not extracellular matrix | sp|Q92506|DHB8_HUMAN | (3R)-3-hydroxyacyl-CoA dehydrogenase | 14.27 | 15.78 | 15.57 |
| 1557 | HSDL1 | Non-matrisome | Non-matrisome | GO:CC not extracellular matrix | sp|Q3SXM5|HSDL1_HUMAN | Inactive hydroxysteroid dehydrogenase-like protein 1 | 17 | 17.67 | 17.67 |
| 1558 | HSDL2 | Non-matrisome | Non-matrisome | GO:CC not extracellular matrix | sp|Q6YN16|HSDL2_HUMAN | Hydroxysteroid dehydrogenase-like protein 2 | 18.16 | 18.26 | 18.18 |
| 1559 | HSP90AA1 | Non-matrisome | Non-matrisome | GO:CC extracellular matrix (GO:0005576, GO:0005578 & GO:0031012) & GO:CC not extracellular matrix | sp|P07900|HS90A_HUMAN | Heat shock protein HSP 90-alpha | 19.49 | 19.27 | 19.14 |
| 1560 | HSP90AB1 | Non-matrisome | Non-matrisome | GO:CC extracellular matrix (GO:0005576, GO:0005578 & GO:0031012) & GO:CC not extracellular matrix | sp|P08238|HS90B_HUMAN | Heat shock protein HSP 90-beta | 17.24 | 16.75 | 16.99 |
| 1561 | HSP90B1 | Non-matrisome | Non-matrisome | GO:CC extracellular matrix (GO:0005576, GO:0005578 & GO:0031012) & GO:CC not extracellular matrix | sp|P14625|ENPL_HUMAN | Endoplasmin | 17.84 | 18.06 | 18.01 |
| 1562 | HSPA12A | Non-matrisome | Non-matrisome | GO:CC not extracellular matrix | sp|O43301|HS12A_HUMAN | Heat shock 70 kDa protein 12A | 16.56 | 18.13 | 17.93 |
| 1563 | HSPA13 | Non-matrisome | Non-matrisome | GO:CC not extracellular matrix | sp|P48723|HSP13_HUMAN | Heat shock 70 kDa protein 13 | 17.33 | 17.3 | 17.54 |
| 1564 | HSPA14 | Non-matrisome | Non-matrisome | GO:CC not extracellular matrix | sp|Q0VDF9|HSP7E_HUMAN | Heat shock 70 kDa protein 14 | 17.5 | 17.19 | 17.24 |
| 1565 | HSPA1L | Non-matrisome | Non-matrisome | GO:CC not extracellular matrix | sp|P34931|HS71L_HUMAN | Heat shock 70 kDa protein 1-like | 16.09 | 16.46 | 16.36 |
| 1566 | HSPA2 | Non-matrisome | Non-matrisome | GO:CC not extracellular matrix | sp|P54652|HSP72_HUMAN | Heat shock-related 70 kDa protein 2 | 17.87 | 17.38 | 17.68 |
| 1567 | HSPA4 | Non-matrisome | Non-matrisome | GO:CC not extracellular matrix | sp|P34932|HSP74_HUMAN | Heat shock 70 kDa protein 4 | 18 | 17.8 | 17.86 |
| 1568 | HSPA4L | Non-matrisome | Non-matrisome | GO:CC not extracellular matrix | sp|O95757|HS74L_HUMAN | Heat shock 70 kDa protein 4L | 16.69 | 17.77 | 18.01 |
| 1569 | HSPA5 | Non-matrisome | Non-matrisome | GO:CC not extracellular matrix | sp|P11021|BIP_HUMAN | Endoplasmic reticulum chaperone BiP | 18.92 | 18.84 | 18.67 |
| 1570 | HSPA8 | Non-matrisome | Non-matrisome | GO:CC extracellular matrix (GO:0005576, GO:0005578 & GO:0031012) & GO:CC not extracellular matrix | sp|P11142|HSP7C_HUMAN | Heat shock cognate 71 kDa protein | 19.21 | 18.53 | 18.75 |
| 1571 | HSPA9 | Non-matrisome | Non-matrisome | GO:CC not extracellular matrix | sp|P38646|GRP75_HUMAN | Stress-70 protein, mitochondrial | 19.59 | 19.72 | 19.38 |
| 1572 | HSPB1 | Non-matrisome | Non-matrisome | GO:CC not extracellular matrix | sp|P04792|HSPB1_HUMAN | Heat shock protein beta-1 | 18.31 | 18.21 | 18.34 |
| 1573 | HSPB6 | Non-matrisome | Non-matrisome | GO:CC extracellular matrix (GO:0005576, GO:0005578 & GO:0031012) & GO:CC not extracellular matrix | sp|O14558|HSPB6_HUMAN | Heat shock protein beta-6 | 14.28 | 14.67 | 14.75 |
| 1574 | HSPB7 | Non-matrisome | Non-matrisome | GO:CC not extracellular matrix | sp|Q9UBY9|HSPB7_HUMAN | Heat shock protein beta-7 | 16.34 | 17.98 | 17.73 |
| 1575 | HSPB8 | Non-matrisome | Non-matrisome | GO:CC not extracellular matrix | sp|Q9UJY1|HSPB8_HUMAN | Heat shock protein beta-8 | 17.87 | 17.2 | 17.12 |
| 1576 | HSPBP1 | Non-matrisome | Non-matrisome | GO:CC not extracellular matrix | sp|Q9NZL4|HPBP1_HUMAN | Hsp70-binding protein 1 | 18.38 | 17.9 | 17.52 |
| 1577 | HSPD1 | Non-matrisome | Non-matrisome | GO:CC not extracellular matrix | sp|P10809|CH60_HUMAN | 60 kDa heat shock protein, mitochondrial | 19.46 | 19.62 | 19.23 |
| 1578 | HSPE1 | Non-matrisome | Non-matrisome | GO:CC not extracellular matrix | sp|P61604|CH10_HUMAN | 10 kDa heat shock protein, mitochondrial | 20.14 | 20.23 | 20.03 |
| 1579 | HSPG2 | Core matrisome | Proteoglycans | GO:CC extracellular matrix (GO:0005576, GO:0005578 & GO:0031012) & GO:CC not extracellular matrix | sp|P98160|PGBM_HUMAN | Basement membrane-specific heparan sulfate proteoglycan core protein | 15.45 | 18.05 | 17.77 |
| 1580 | HSPH1 | Non-matrisome | Non-matrisome | GO:CC extracellular matrix (GO:0005576, GO:0005578 & GO:0031012) & GO:CC not extracellular matrix | sp|Q92598|HS105_HUMAN | Heat shock protein 105 kDa | 19.45 | 18.54 | 18.82 |
| 1581 | HTATIP2 | Non-matrisome | Non-matrisome | GO:CC not extracellular matrix | sp|Q9BUP3|HTAI2_HUMAN | Oxidoreductase HTATIP2 | 17.88 | 18.17 | 18.1 |
| 1582 | HTATSF1 | Non-matrisome | Non-matrisome | GO:CC not extracellular matrix | sp|O43719|HTSF1_HUMAN | HIV Tat-specific factor 1 | 17 | 17.04 | 16.96 |
| 1583 | HTRA1 | Matrisome-associated | ECM Regulators | GO:CC extracellular matrix (GO:0005576, GO:0005578 & GO:0031012) & GO:CC not extracellular matrix | sp|Q92743|HTRA1_HUMAN | Serine protease HTRA1 | 14.73 | 18.69 | 19.66 |
| 1584 | HTRA2 | Non-matrisome | Non-matrisome | GO:CC not extracellular matrix | sp|O43464|HTRA2_HUMAN | Serine protease HTRA2, mitochondrial | 14.95 | 15.65 | 15.53 |
| 1585 | HTT | Non-matrisome | Non-matrisome | GO:CC not extracellular matrix | sp|P42858|HD_HUMAN | Huntingtin | 16.36 | 16.54 | 16.61 |
| 1586 | HUWE1 | Non-matrisome | Non-matrisome | GO:CC extracellular matrix (GO:0005576, GO:0005578 & GO:0031012) & GO:CC not extracellular matrix | sp|Q7Z6Z7|HUWE1_HUMAN | E3 ubiquitin-protein ligase HUWE1 | 17.54 | 17.82 | 17.69 |
| 1587 | HVCN1 | Non-matrisome | Non-matrisome | GO:CC not extracellular matrix | sp|Q96D96|HVCN1_HUMAN | Voltage-gated hydrogen channel 1 | 16.13 | 17.15 | 17.82 |
| 1588 | HYI | Non-matrisome | Non-matrisome | GO:CC not extracellular matrix | sp|Q5T013|HYI_HUMAN | Putative hydroxypyruvate isomerase | 17.25 | 18.01 | 17.87 |
| 1589 | HYOU1 | Non-matrisome | Non-matrisome | GO:CC extracellular matrix (GO:0005576, GO:0005578 & GO:0031012) & GO:CC not extracellular matrix | sp|Q9Y4L1|HYOU1_HUMAN | Hypoxia up-regulated protein 1 | 18.99 | 18.78 | 18.75 |
| 1590 | HYPK | Non-matrisome | Non-matrisome | GO:CC not extracellular matrix | sp|Q9NX55|HYPK_HUMAN | Huntingtin-interacting protein K | 17.3 | 16.15 | 16.36 |
| 1591 | IAH1 | Non-matrisome | Non-matrisome | GO:CC not extracellular matrix | sp|Q2TAA2|IAH1_HUMAN | Isoamyl acetate-hydrolyzing esterase 1 homolog | 18.88 | 19.14 | 19.16 |
| 1592 | IARS1 | Non-matrisome | Non-matrisome | GO:CC not extracellular matrix | sp|P41252|SYIC_HUMAN | Isoleucine--tRNA ligase, cytoplasmic | 18.74 | 18.54 | 18.37 |
| 1593 | IARS2 | Non-matrisome | Non-matrisome | GO:CC not extracellular matrix | sp|Q9NSE4|SYIM_HUMAN | Isoleucine--tRNA ligase, mitochondrial | 18.38 | 19.16 | 18.91 |
| 1594 | IBA57 | Non-matrisome | Non-matrisome | GO:CC not extracellular matrix | sp|Q5T440|CAF17_HUMAN | Putative transferase CAF17, mitochondrial | 17.61 | 17.92 | 17.57 |
| 1595 | ICAM1 | Non-matrisome | Non-matrisome | GO:CC not extracellular matrix | sp|P05362|ICAM1_HUMAN | Intercellular adhesion molecule 1 | 15.11 | 18.16 | 16.87 |
| 1596 | ICMT | Non-matrisome | Non-matrisome | GO:CC not extracellular matrix | sp|O60725|ICMT_HUMAN | Protein-S-isoprenylcysteine O-methyltransferase | 20.28 | 19.8 | 19.86 |
| 1597 | ICOSLG | Non-matrisome | Non-matrisome | GO:CC not extracellular matrix | sp|O75144|ICOSL_HUMAN | ICOS ligand | 15.58 | 16.91 | 17.2 |
| 1598 | IDE | Non-matrisome | Non-matrisome | GO:CC not extracellular matrix | sp|P14735|IDE_HUMAN | Insulin-degrading enzyme | 17.53 | 17.68 | 17.68 |
| 1599 | IDH1 | Non-matrisome | Non-matrisome | GO:CC extracellular matrix (GO:0005576, GO:0005578 & GO:0031012) & GO:CC not extracellular matrix | sp|O75874|IDHC_HUMAN | Isocitrate dehydrogenase [NADP] cytoplasmic | 20.33 | 20.81 | 20.96 |
| 1600 | IDH2 | Non-matrisome | Non-matrisome | GO:CC not extracellular matrix | sp|P48735|IDHP_HUMAN | Isocitrate dehydrogenase [NADP], mitochondrial | 18.21 | 18.78 | 18.29 |
| 1601 | IDH3A | Non-matrisome | Non-matrisome | GO:CC not extracellular matrix | sp|P50213|IDH3A_HUMAN | Isocitrate dehydrogenase [NAD] subunit alpha, mitochondrial | 18.61 | 19.01 | 18.77 |
| 1602 | IDH3B | Non-matrisome | Non-matrisome | GO:CC not extracellular matrix | sp|O43837|IDH3B_HUMAN | Isocitrate dehydrogenase [NAD] subunit beta, mitochondrial | 17.87 | 18.04 | 17.88 |
| 1603 | IDH3G | Non-matrisome | Non-matrisome | GO:CC not extracellular matrix | sp|P51553|IDH3G_HUMAN | Isocitrate dehydrogenase [NAD] subunit gamma, mitochondrial | 17.27 | 17.7 | 17.59 |
| 1604 | IDI1 | Non-matrisome | Non-matrisome | GO:CC not extracellular matrix | sp|Q13907|IDI1_HUMAN | Isopentenyl-diphosphate Delta-isomerase 1 | 19.01 | 17.99 | 18.44 |
| 1605 | IDNK | Non-matrisome | Non-matrisome | GO:CC not extracellular matrix | sp|Q5T6J7|GNTK_HUMAN | Probable gluconokinase | 13.55 | 14.22 | 14.32 |
| 1606 | IDS | Non-matrisome | Non-matrisome | GO:CC not extracellular matrix | sp|P22304|IDS_HUMAN | Iduronate 2-sulfatase | 14.7 | 15.67 | 16.03 |
| 1607 | IDUA | Non-matrisome | Non-matrisome | GO:CC not extracellular matrix | sp|P35475|IDUA_HUMAN | Alpha-L-iduronidase | 15.74 | 17.36 | 17.98 |
| 1608 | IFI16 | Non-matrisome | Non-matrisome | GO:CC not extracellular matrix | sp|Q16666|IF16_HUMAN | Gamma-interferon-inducible protein 16 | 17.25 | 18.27 | 18.19 |
| 1609 | IFI30 | Non-matrisome | Non-matrisome | GO:CC extracellular matrix (GO:0005576, GO:0005578 & GO:0031012) & GO:CC not extracellular matrix | sp|P13284|GILT_HUMAN | Gamma-interferon-inducible lysosomal thiol reductase | 15.77 | 17.11 | 16.96 |
| 1610 | IFI35 | Non-matrisome | Non-matrisome | GO:CC not extracellular matrix | sp|P80217|IN35_HUMAN | Interferon-induced 35 kDa protein | 17.58 | 17.28 | 17.18 |
| 1611 | IFIT1 | Non-matrisome | Non-matrisome | GO:CC not extracellular matrix | sp|P09914|IFIT1_HUMAN | Interferon-induced protein with tetratricopeptide repeats 1 | 17.31 | 17.5 | 16.97 |
| 1612 | IFIT3 | Non-matrisome | Non-matrisome | GO:CC not extracellular matrix | sp|O14879|IFIT3_HUMAN | Interferon-induced protein with tetratricopeptide repeats 3 | 17.79 | 18.06 | 17.72 |
| 1613 | IFIT5 | Non-matrisome | Non-matrisome | GO:CC not extracellular matrix | sp|Q13325|IFIT5_HUMAN | Interferon-induced protein with tetratricopeptide repeats 5 | 18.87 | 18.56 | 18.71 |
| 1614 | IFT140 | Non-matrisome | Non-matrisome | GO:CC not extracellular matrix | sp|Q96RY7|IF140_HUMAN | Intraflagellar transport protein 140 homolog | 16.37 | 16.99 | 17.08 |
| 1615 | IGBP1 | Non-matrisome | Non-matrisome | GO:CC not extracellular matrix | sp|P78318|IGBP1_HUMAN | Immunoglobulin-binding protein 1 | 17.87 | 17.41 | 17.48 |
| 1616 | IGF2R | Non-matrisome | Non-matrisome | GO:CC not extracellular matrix | sp|P11717|MPRI_HUMAN | Cation-independent mannose-6-phosphate receptor | 17.36 | 17.5 | 17.57 |
| 1617 | IGFBP5 | Core matrisome | ECM Glycoproteins | GO:CC extracellular matrix (GO:0005576, GO:0005578 & GO:0031012) & GO:CC not extracellular matrix | sp|P24593|IBP5_HUMAN | Insulin-like growth factor-binding protein 5 | 16.12 | 19.16 | 18.19 |
| 1618 | IGFBP7 | Core matrisome | ECM Glycoproteins | GO:CC extracellular matrix (GO:0005576, GO:0005578 & GO:0031012) & GO:CC not extracellular matrix | sp|Q16270|IBP7_HUMAN | Insulin-like growth factor-binding protein 7 | 17.53 | 17.43 | 17.48 |
| 1619 | IGLL5 | Non-matrisome | Non-matrisome | GO:CC not extracellular matrix | sp|B9A064|IGLL5_HUMAN | Immunoglobulin lambda-like polypeptide 5 | 19.37 | 18.62 | 18.91 |
| 1620 | IGSF8 | Non-matrisome | Non-matrisome | GO:CC not extracellular matrix | sp|Q969P0|IGSF8_HUMAN | Immunoglobulin superfamily member 8 | 15.07 | 16.31 | 16.37 |
| 1621 | IK | Non-matrisome | Non-matrisome | GO:CC not extracellular matrix | sp|Q13123|RED_HUMAN | Protein Red | 17.72 | 17.35 | 17.49 |
| 1622 | IKBIP | Non-matrisome | Non-matrisome | GO:CC not extracellular matrix | sp|Q70UQ0|IKIP_HUMAN | Inhibitor of nuclear factor kappa-B kinase-interacting protein | 20.14 | 20.62 | 20.53 |
| 1623 | IKBKB | Non-matrisome | Non-matrisome | GO:CC not extracellular matrix | sp|O14920|IKKB_HUMAN | Inhibitor of nuclear factor kappa-B kinase subunit beta | 16.82 | 17.42 | 17.39 |
| 1624 | IL4I1 | Non-matrisome | Non-matrisome | GO:CC extracellular matrix (GO:0005576, GO:0005578 & GO:0031012) & GO:CC not extracellular matrix | sp|Q96RQ9|OXLA_HUMAN | L-amino-acid oxidase | 14.37 | 14.44 | 14.61 |
| 1625 | ILF2 | Non-matrisome | Non-matrisome | GO:CC extracellular matrix (GO:0005576, GO:0005578 & GO:0031012) & GO:CC not extracellular matrix | sp|Q12905|ILF2_HUMAN | Interleukin enhancer-binding factor 2 | 20.14 | 20.19 | 20.21 |
| 1626 | ILF3 | Non-matrisome | Non-matrisome | GO:CC extracellular matrix (GO:0005576, GO:0005578 & GO:0031012) & GO:CC not extracellular matrix | sp|Q12906|ILF3_HUMAN | Interleukin enhancer-binding factor 3 | 18.19 | 18.26 | 18.33 |
| 1627 | ILK | Non-matrisome | Non-matrisome | GO:CC not extracellular matrix | sp|Q13418|ILK_HUMAN | Integrin-linked protein kinase | 19.67 | 19.68 | 19.44 |
| 1628 | ILKAP | Non-matrisome | Non-matrisome | GO:CC not extracellular matrix | sp|Q9H0C8|ILKAP_HUMAN | Integrin-linked kinase-associated serine/threonine phosphatase 2C | 18.28 | 17.79 | 17.68 |
| 1629 | ILVBL | Non-matrisome | Non-matrisome | GO:CC not extracellular matrix | sp|A1L0T0|HACL2_HUMAN | 2-hydroxyacyl-CoA lyase 2 | 18.92 | 19.21 | 18.99 |
| 1630 | IMMT | Non-matrisome | Non-matrisome | GO:CC not extracellular matrix | sp|Q16891|MIC60_HUMAN | MICOS complex subunit MIC60 | 18.39 | 19.12 | 18.97 |
| 1631 | IMP3 | Non-matrisome | Non-matrisome | GO:CC not extracellular matrix | sp|Q9NV31|IMP3_HUMAN | U3 small nucleolar ribonucleoprotein protein IMP3 | 18.64 | 17.93 | 17.92 |
| 1632 | IMPA1 | Non-matrisome | Non-matrisome | GO:CC not extracellular matrix | sp|P29218|IMPA1_HUMAN | Inositol monophosphatase 1 | 19.05 | 18.98 | 18.97 |
| 1633 | IMPA2 | Non-matrisome | Non-matrisome | GO:CC not extracellular matrix | sp|O14732|IMPA2_HUMAN | Inositol monophosphatase 2 | 17.14 | 17.02 | 16.92 |
| 1634 | IMPDH1 | Non-matrisome | Non-matrisome | GO:CC extracellular matrix (GO:0005576, GO:0005578 & GO:0031012) & GO:CC not extracellular matrix | sp|P20839|IMDH1_HUMAN | Inosine-5'-monophosphate dehydrogenase 1 | 18.5 | 17.54 | 17.1 |
| 1635 | IMPDH2 | Non-matrisome | Non-matrisome | GO:CC extracellular matrix (GO:0005576, GO:0005578 & GO:0031012) & GO:CC not extracellular matrix | sp|P12268|IMDH2_HUMAN | Inosine-5'-monophosphate dehydrogenase 2 | 19.18 | 18.39 | 18.14 |
| 1636 | IMUP | Non-matrisome | Non-matrisome | GO:CC not extracellular matrix | sp|Q9GZP8|IMUP_HUMAN | Immortalization up-regulated protein | 14.65 | 14.21 | 13.63 |
| 1637 | INA | Non-matrisome | Non-matrisome | GO:CC not extracellular matrix | sp|Q16352|AINX_HUMAN | Alpha-internexin | 17.13 | 18.37 | 18.44 |
| 1638 | INF2 | Non-matrisome | Non-matrisome | GO:CC not extracellular matrix | sp|Q27J81|INF2_HUMAN | Inverted formin-2 | 18.05 | 17.45 | 17.27 |
| 1639 | ING5 | Non-matrisome | Non-matrisome | GO:CC not extracellular matrix | sp|Q8WYH8|ING5_HUMAN | Inhibitor of growth protein 5 | 15.63 | 15.86 | 15.61 |
| 1640 | INPP1 | Non-matrisome | Non-matrisome | GO:CC not extracellular matrix | sp|P49441|INPP_HUMAN | Inositol polyphosphate 1-phosphatase | 17.7 | 17.89 | 18.05 |
| 1641 | INPP5D | Non-matrisome | Non-matrisome | GO:CC not extracellular matrix | sp|Q92835|SHIP1_HUMAN | Phosphatidylinositol 3,4,5-trisphosphate 5-phosphatase 1 | 14.86 | 16.27 | 16.55 |
| 1642 | INTS10 | Non-matrisome | Non-matrisome | GO:CC not extracellular matrix | sp|Q9NVR2|INT10_HUMAN | Integrator complex subunit 10 | 16.48 | 16.51 | 16.5 |
| 1643 | INTS14 | Non-matrisome | Non-matrisome | GO:CC not extracellular matrix | sp|Q96SY0|INT14_HUMAN | Integrator complex subunit 14 | 15.85 | 15.83 | 15.84 |
| 1644 | INTS2 | Non-matrisome | Non-matrisome | GO:CC not extracellular matrix | sp|Q9H0H0|INT2_HUMAN | Integrator complex subunit 2 | 16.69 | 16.72 | 16.79 |
| 1645 | INTS3 | Non-matrisome | Non-matrisome | GO:CC not extracellular matrix | sp|Q68E01|INT3_HUMAN | Integrator complex subunit 3 | 17.03 | 16.85 | 17.09 |
| 1646 | INTS6 | Non-matrisome | Non-matrisome | GO:CC not extracellular matrix | sp|Q9UL03|INT6_HUMAN | Integrator complex subunit 6 | 17.12 | 17.13 | 17.23 |
| 1647 | INTS8 | Non-matrisome | Non-matrisome | GO:CC not extracellular matrix | sp|Q75QN2|INT8_HUMAN | Integrator complex subunit 8 | 16.86 | 16.94 | 16.97 |
| 1648 | IPO4 | Non-matrisome | Non-matrisome | GO:CC not extracellular matrix | sp|Q8TEX9|IPO4_HUMAN | Importin-4 | 18.86 | 18.36 | 18.13 |
| 1649 | IPO5 | Non-matrisome | Non-matrisome | GO:CC not extracellular matrix | sp|O00410|IPO5_HUMAN | Importin-5 | 19.61 | 18.84 | 18.49 |
| 1650 | IPO7 | Non-matrisome | Non-matrisome | GO:CC not extracellular matrix | sp|O95373|IPO7_HUMAN | Importin-7 | 18.96 | 18.17 | 17.92 |
| 1651 | IPO8 | Non-matrisome | Non-matrisome | GO:CC not extracellular matrix | sp|O15397|IPO8_HUMAN | Importin-8 | 16.98 | 16.16 | 16.44 |
| 1652 | IPO9 | Non-matrisome | Non-matrisome | GO:CC not extracellular matrix | sp|Q96P70|IPO9_HUMAN | Importin-9 | 19.51 | 19.2 | 19.04 |
| 1653 | IQGAP1 | Non-matrisome | Non-matrisome | GO:CC not extracellular matrix | sp|P46940|IQGA1_HUMAN | Ras GTPase-activating-like protein IQGAP1 | 18.18 | 18.63 | 18.48 |
| 1654 | IQGAP2 | Non-matrisome | Non-matrisome | GO:CC not extracellular matrix | sp|Q13576|IQGA2_HUMAN | Ras GTPase-activating-like protein IQGAP2 | 15.65 | 15.83 | 15.9 |
| 1655 | IQGAP3 | Non-matrisome | Non-matrisome | GO:CC not extracellular matrix | sp|Q86VI3|IQGA3_HUMAN | Ras GTPase-activating-like protein IQGAP3 | 17.87 | 16.55 | 16.18 |
| 1656 | IRF2BP2 | Non-matrisome | Non-matrisome | GO:CC not extracellular matrix | sp|Q7Z5L9|I2BP2_HUMAN | Interferon regulatory factor 2-binding protein 2 | 16.99 | 17.19 | 17.26 |
| 1657 | IRF2BPL | Non-matrisome | Non-matrisome | GO:CC not extracellular matrix | sp|Q9H1B7|I2BPL_HUMAN | Probable E3 ubiquitin-protein ligase IRF2BPL | 15.95 | 17.18 | 17.57 |
| 1658 | IRF3 | Non-matrisome | Non-matrisome | GO:CC not extracellular matrix | sp|Q14653|IRF3_HUMAN | Interferon regulatory factor 3 | 18.44 | 18.59 | 18.63 |
| 1659 | IRF9 | Non-matrisome | Non-matrisome | GO:CC not extracellular matrix | sp|Q00978|IRF9_HUMAN | Interferon regulatory factor 9 | 16.11 | 17.26 | 17.24 |
| 1660 | IRGQ | Non-matrisome | Non-matrisome | GO:CC not extracellular matrix | sp|Q8WZA9|IRGQ_HUMAN | Immunity-related GTPase family Q protein | 17.96 | 17.41 | 17.72 |
| 1661 | ISG15 | Non-matrisome | Non-matrisome | GO:CC extracellular matrix (GO:0005576, GO:0005578 & GO:0031012) & GO:CC not extracellular matrix | sp|P05161|ISG15_HUMAN | Ubiquitin-like protein ISG15 | 21.1 | 20.76 | 20.45 |
| 1662 | ISLR | Non-matrisome | Non-matrisome | GO:CC extracellular matrix (GO:0005576, GO:0005578 & GO:0031012) & GO:CC not extracellular matrix | sp|O14498|ISLR_HUMAN | Immunoglobulin superfamily containing leucine-rich repeat protein | 15.3 | 17.42 | 18.01 |
| 1663 | ISOC1 | Non-matrisome | Non-matrisome | GO:CC not extracellular matrix | sp|Q96CN7|ISOC1_HUMAN | Isochorismatase domain-containing protein 1 | 19.22 | 19.18 | 19.04 |
| 1664 | ISOC2 | Non-matrisome | Non-matrisome | GO:CC not extracellular matrix | sp|Q96AB3|ISOC2_HUMAN | Isochorismatase domain-containing protein 2 | 17.24 | 17.73 | 17.82 |
| 1665 | IST1 | Non-matrisome | Non-matrisome | GO:CC extracellular matrix (GO:0005576, GO:0005578 & GO:0031012) & GO:CC not extracellular matrix | sp|P53990|IST1_HUMAN | IST1 homolog | 17.89 | 18.07 | 18.25 |
| 1666 | ISY1 | Non-matrisome | Non-matrisome | GO:CC not extracellular matrix | sp|Q9ULR0|ISY1_HUMAN | Pre-mRNA-splicing factor ISY1 homolog | 18.1 | 17.45 | 17.47 |
| 1667 | ISYNA1 | Non-matrisome | Non-matrisome | GO:CC not extracellular matrix | sp|Q9NPH2|INO1_HUMAN | Inositol-3-phosphate synthase 1 | 17.65 | 19.09 | 19.49 |
| 1668 | ITGA1 | Non-matrisome | Non-matrisome | GO:CC not extracellular matrix | sp|P56199|ITA1_HUMAN | Integrin alpha-1 | 18.26 | 18.77 | 19.63 |
| 1669 | ITGA2 | Non-matrisome | Non-matrisome | GO:CC not extracellular matrix | sp|P17301|ITA2_HUMAN | Integrin alpha-2 | 16.84 | 18.3 | 18.62 |
| 1670 | ITGA3 | Non-matrisome | Non-matrisome | GO:CC not extracellular matrix | sp|P26006|ITA3_HUMAN | Integrin alpha-3 | 18.64 | 18.27 | 18.16 |
| 1671 | ITGA5 | Non-matrisome | Non-matrisome | GO:CC not extracellular matrix | sp|P08648|ITA5_HUMAN | Integrin alpha-5 | 18.98 | 16.75 | 16.68 |
| 1672 | ITGA6 | Non-matrisome | Non-matrisome | GO:CC not extracellular matrix | sp|P23229|ITA6_HUMAN | Integrin alpha-6 | 18.29 | 16.57 | 16.8 |
| 1673 | ITGA7 | Non-matrisome | Non-matrisome | GO:CC not extracellular matrix | sp|Q13683|ITA7_HUMAN | Integrin alpha-7 | 13.71 | 15.63 | 15.99 |
| 1674 | ITGA8 | Non-matrisome | Non-matrisome | GO:CC not extracellular matrix | sp|P53708|ITA8_HUMAN | Integrin alpha-8 | 17.45 | 17.4 | 18.13 |
| 1675 | ITGAL | Non-matrisome | Non-matrisome | GO:CC not extracellular matrix | sp|P20701|ITAL_HUMAN | Integrin alpha-L | 14.58 | 14.67 | 14.53 |
| 1676 | ITGAV | Non-matrisome | Non-matrisome | GO:CC not extracellular matrix | sp|P06756|ITAV_HUMAN | Integrin alpha-V | 18.43 | 18.83 | 19.21 |
| 1677 | ITGB1 | Non-matrisome | Non-matrisome | GO:CC not extracellular matrix | sp|P05556|ITB1_HUMAN | Integrin beta-1 | 20.04 | 19.81 | 20 |
| 1678 | ITGB3 | Non-matrisome | Non-matrisome | GO:CC not extracellular matrix | sp|P05106|ITB3_HUMAN | Integrin beta-3 | 16.96 | 17.71 | 18.02 |
| 1679 | ITGB4 | Non-matrisome | Non-matrisome | GO:CC not extracellular matrix | sp|P16144|ITB4_HUMAN | Integrin beta-4 | 14.54 | 14.08 | 14.16 |
| 1680 | ITGB5 | Non-matrisome | Non-matrisome | GO:CC not extracellular matrix | sp|P18084|ITB5_HUMAN | Integrin beta-5 | 17.18 | 17.52 | 18.04 |
| 1681 | ITIH2 | Matrisome-associated | ECM Regulators | GO:CC extracellular matrix (GO:0005576, GO:0005578 & GO:0031012) & GO:CC not extracellular matrix | sp|P19823|ITIH2_HUMAN | Inter-alpha-trypsin inhibitor heavy chain H2 | 17.68 | 17.84 | 17.88 |
| 1682 | ITIH3 | Matrisome-associated | ECM Regulators | GO:CC extracellular matrix (GO:0005576, GO:0005578 & GO:0031012) & GO:CC not extracellular matrix | sp|Q06033|ITIH3_HUMAN | Inter-alpha-trypsin inhibitor heavy chain H3 | 20.06 | 19.16 | 20.39 |
| 1683 | ITIH4 | Matrisome-associated | ECM Regulators | GO:CC extracellular matrix (GO:0005576, GO:0005578 & GO:0031012) & GO:CC not extracellular matrix | sp|Q14624|ITIH4_HUMAN | Inter-alpha-trypsin inhibitor heavy chain H4 | 20.36 | 19.28 | 20.09 |
| 1684 | ITM2B | Non-matrisome | Non-matrisome | GO:CC extracellular matrix (GO:0005576, GO:0005578 & GO:0031012) & GO:CC not extracellular matrix | sp|Q9Y287|ITM2B_HUMAN | Integral membrane protein 2B | 15.75 | 16.94 | 16.84 |
| 1685 | ITPA | Non-matrisome | Non-matrisome | GO:CC not extracellular matrix | sp|Q9BY32|ITPA_HUMAN | Inosine triphosphate pyrophosphatase | 17.43 | 17.41 | 17.47 |
| 1686 | ITPKC | Non-matrisome | Non-matrisome | GO:CC not extracellular matrix | sp|Q96DU7|IP3KC_HUMAN | Inositol-trisphosphate 3-kinase C | 16.8 | 15.81 | 15.82 |
| 1687 | ITPR1 | Non-matrisome | Non-matrisome | GO:CC not extracellular matrix | sp|Q14643|ITPR1_HUMAN | Inositol 1,4,5-trisphosphate receptor type 1 | 14.86 | 17.66 | 16.6 |
| 1688 | ITPR2 | Non-matrisome | Non-matrisome | GO:CC not extracellular matrix | sp|Q14571|ITPR2_HUMAN | Inositol 1,4,5-trisphosphate receptor type 2 | 16 | 16.7 | 16.66 |
| 1689 | ITPR3 | Non-matrisome | Non-matrisome | GO:CC not extracellular matrix | sp|Q14573|ITPR3_HUMAN | Inositol 1,4,5-trisphosphate receptor type 3 | 17.43 | 18.25 | 18.12 |
| 1690 | ITPRID2 | Non-matrisome | Non-matrisome | GO:CC not extracellular matrix | sp|P28290|ITPI2_HUMAN | Protein ITPRID2 | 17.17 | 17.11 | 17.2 |
| 1691 | ITPRIP | Non-matrisome | Non-matrisome | GO:CC not extracellular matrix | sp|Q8IWB1|IPRI_HUMAN | Inositol 1,4,5-trisphosphate receptor-interacting protein | 17.76 | 18.08 | 18.27 |
| 1692 | IVD | Non-matrisome | Non-matrisome | GO:CC not extracellular matrix | sp|P26440|IVD_HUMAN | Isovaleryl-CoA dehydrogenase, mitochondrial | 17.81 | 18.38 | 18.29 |
| 1693 | IWS1 | Non-matrisome | Non-matrisome | GO:CC not extracellular matrix | sp|Q96ST2|IWS1_HUMAN | Protein IWS1 homolog | 16.99 | 16.69 | 16.83 |
| 1694 | JAK1 | Non-matrisome | Non-matrisome | GO:CC not extracellular matrix | sp|P23458|JAK1_HUMAN | Tyrosine-protein kinase JAK1 | 17.87 | 17.47 | 17.29 |
| 1695 | JAM3 | Non-matrisome | Non-matrisome | GO:CC not extracellular matrix | sp|Q9BX67|JAM3_HUMAN | Junctional adhesion molecule C | 17.33 | 17.22 | 17.35 |
| 1696 | JMJD6 | Non-matrisome | Non-matrisome | GO:CC extracellular matrix (GO:0005576, GO:0005578 & GO:0031012) & GO:CC not extracellular matrix | sp|Q6NYC1|JMJD6_HUMAN | Bifunctional arginine demethylase and lysyl-hydroxylase JMJD6 | 17.31 | 16.81 | 16.89 |
| 1697 | JMJD7 | Non-matrisome | Non-matrisome | GO:CC extracellular matrix (GO:0005576, GO:0005578 & GO:0031012) & GO:CC not extracellular matrix | sp|P0C870|JMJD7_HUMAN | Bifunctional peptidase and (3S)-lysyl hydroxylase JMJD7 | 14.89 | 15.08 | 15.2 |
| 1698 | JPT1 | Non-matrisome | Non-matrisome | GO:CC not extracellular matrix | sp|Q9UK76|JUPI1_HUMAN | Jupiter microtubule associated homolog 1 | 20.36 | 18.48 | 18.68 |
| 1699 | JPT2 | Non-matrisome | Non-matrisome | GO:CC not extracellular matrix | sp|Q9H910|JUPI2_HUMAN | Jupiter microtubule associated homolog 2 | 19.48 | 18.06 | 18.15 |
| 1700 | JUP | Non-matrisome | Non-matrisome | GO:CC extracellular matrix (GO:0005576, GO:0005578 & GO:0031012) & GO:CC not extracellular matrix | sp|P14923|PLAK_HUMAN | Junction plakoglobin | 14.25 | 15.72 | 16.17 |
| 1701 | KANK2 | Non-matrisome | Non-matrisome | GO:CC not extracellular matrix | sp|Q63ZY3|KANK2_HUMAN | KN motif and ankyrin repeat domain-containing protein 2 | 16.94 | 17.53 | 17.64 |
| 1702 | KARS1 | Non-matrisome | Non-matrisome | GO:CC not extracellular matrix | sp|Q15046|SYK_HUMAN | Lysine--tRNA ligase | 19.58 | 19.12 | 18.82 |
| 1703 | KAT5 | Non-matrisome | Non-matrisome | GO:CC not extracellular matrix | sp|Q92993|KAT5_HUMAN | Histone acetyltransferase KAT5 | 17.23 | 17.36 | 17.4 |
| 1704 | KBTBD11 | Non-matrisome | Non-matrisome | gene not present in GO IDs | sp|O94819|KBTBB_HUMAN | Kelch repeat and BTB domain-containing protein 11 | 15.7 | 16.66 | 16.81 |
| 1705 | KCNAB2 | Non-matrisome | Non-matrisome | GO:CC not extracellular matrix | sp|Q13303|KCAB2_HUMAN | Voltage-gated potassium channel subunit beta-2 | 16.51 | 17.36 | 17.54 |
| 1706 | KCTD12 | Non-matrisome | Non-matrisome | GO:CC not extracellular matrix | sp|Q96CX2|KCD12_HUMAN | BTB/POZ domain-containing protein KCTD12 | 16.36 | 16.34 | 17.07 |
| 1707 | KCTD5 | Non-matrisome | Non-matrisome | GO:CC not extracellular matrix | sp|Q9NXV2|KCTD5_HUMAN | BTB/POZ domain-containing protein KCTD5 | 17.46 | 16.34 | 16.43 |
| 1708 | KDELR1 | Non-matrisome | Non-matrisome | GO:CC not extracellular matrix | sp|P24390|ERD21_HUMAN | ER lumen protein-retaining receptor 1 | 15.82 | 15.71 | 15.44 |
| 1709 | KDM3B | Non-matrisome | Non-matrisome | GO:CC not extracellular matrix | sp|Q7LBC6|KDM3B_HUMAN | Lysine-specific demethylase 3B | 16.76 | 16.89 | 16.94 |
| 1710 | KDSR | Non-matrisome | Non-matrisome | GO:CC not extracellular matrix | sp|Q06136|KDSR_HUMAN | 3-ketodihydrosphingosine reductase | 18.41 | 18.78 | 18.66 |
| 1711 | KEAP1 | Non-matrisome | Non-matrisome | GO:CC not extracellular matrix | sp|Q14145|KEAP1_HUMAN | Kelch-like ECH-associated protein 1 | 16.65 | 16.42 | 16.53 |
| 1712 | KHDRBS1 | Non-matrisome | Non-matrisome | GO:CC not extracellular matrix | sp|Q07666|KHDR1_HUMAN | KH domain-containing, RNA-binding, signal transduction-associated protein 1 | 17.87 | 17.94 | 17.99 |
| 1713 | KHSRP | Non-matrisome | Non-matrisome | GO:CC not extracellular matrix | sp|Q92945|FUBP2_HUMAN | Far upstream element-binding protein 2 | 18.09 | 18.11 | 18.11 |
| 1714 | KIAA1217 | Non-matrisome | Non-matrisome | GO:CC not extracellular matrix | sp|Q5T5P2|SKT_HUMAN | Sickle tail protein homolog | 15.56 | 16.69 | 16.46 |
| 1715 | KIAA1522 | Non-matrisome | Non-matrisome | GO:CC not extracellular matrix | sp|Q9P206|K1522_HUMAN | Uncharacterized protein KIAA1522 | 16.14 | 16.07 | 15.94 |
| 1716 | KIF13B | Non-matrisome | Non-matrisome | GO:CC not extracellular matrix | sp|Q9NQT8|KI13B_HUMAN | Kinesin-like protein KIF13B | 16.56 | 16.97 | 17.24 |
| 1717 | KIF20B | Non-matrisome | Non-matrisome | GO:CC not extracellular matrix | sp|Q96Q89|KI20B_HUMAN | Kinesin-like protein KIF20B | 16.1 | 14.84 | 14.98 |
| 1718 | KIF2A | Non-matrisome | Non-matrisome | GO:CC not extracellular matrix | sp|O00139|KIF2A_HUMAN | Kinesin-like protein KIF2A | 18.79 | 18.7 | 18.72 |
| 1719 | KIF5B | Non-matrisome | Non-matrisome | GO:CC not extracellular matrix | sp|P33176|KINH_HUMAN | Kinesin-1 heavy chain | 19.05 | 19.03 | 19.03 |
| 1720 | KIF7 | Non-matrisome | Non-matrisome | GO:CC not extracellular matrix | sp|Q2M1P5|KIF7_HUMAN | Kinesin-like protein KIF7 | 16.81 | 16.31 | 16.63 |
| 1721 | KLC1 | Non-matrisome | Non-matrisome | GO:CC not extracellular matrix | sp|Q07866|KLC1_HUMAN | Kinesin light chain 1 | 18.34 | 18.16 | 18.2 |
| 1722 | KLC2 | Non-matrisome | Non-matrisome | GO:CC not extracellular matrix | sp|Q9H0B6|KLC2_HUMAN | Kinesin light chain 2 | 17.26 | 16.95 | 17 |
| 1723 | KLC4 | Non-matrisome | Non-matrisome | GO:CC not extracellular matrix | sp|Q9NSK0|KLC4_HUMAN | Kinesin light chain 4 | 16.58 | 17.28 | 17.16 |
| 1724 | KLF4 | Non-matrisome | Non-matrisome | GO:CC not extracellular matrix | sp|O43474|KLF4_HUMAN | Krueppel-like factor 4 | 15.27 | 14.52 | 14.07 |
| 1725 | KMT2D | Non-matrisome | Non-matrisome | GO:CC not extracellular matrix | sp|O14686|KMT2D_HUMAN | Histone-lysine N-methyltransferase 2D | 16.26 | 16.09 | 16.14 |
| 1726 | KNG1 | Matrisome-associated | ECM Regulators | GO:CC extracellular matrix (GO:0005576, GO:0005578 & GO:0031012) & GO:CC not extracellular matrix | sp|P01042|KNG1_HUMAN | Kininogen-1 | 19.01 | 18.1 | 18.01 |
| 1727 | KPNA1 | Non-matrisome | Non-matrisome | GO:CC not extracellular matrix | sp|P52294|IMA5_HUMAN | Importin subunit alpha-5 | 18.33 | 17.94 | 17.69 |
| 1728 | KPNA2 | Non-matrisome | Non-matrisome | GO:CC not extracellular matrix | sp|P52292|IMA1_HUMAN | Importin subunit alpha-1 | 18.9 | 15.89 | 16.62 |
| 1729 | KPNA3 | Non-matrisome | Non-matrisome | GO:CC not extracellular matrix | sp|O00505|IMA4_HUMAN | Importin subunit alpha-4 | 18.86 | 18.15 | 18.17 |
| 1730 | KPNA4 | Non-matrisome | Non-matrisome | GO:CC not extracellular matrix | sp|O00629|IMA3_HUMAN | Importin subunit alpha-3 | 18.83 | 18.05 | 18.01 |
| 1731 | KPNA6 | Non-matrisome | Non-matrisome | GO:CC not extracellular matrix | sp|O60684|IMA7_HUMAN | Importin subunit alpha-7 | 17.88 | 17.61 | 17.52 |
| 1732 | KPNB1 | Non-matrisome | Non-matrisome | GO:CC extracellular matrix (GO:0005576, GO:0005578 & GO:0031012) & GO:CC not extracellular matrix | sp|Q14974|IMB1_HUMAN | Importin subunit beta-1 | 20.17 | 19.93 | 19.61 |
| 1733 | KRR1 | Non-matrisome | Non-matrisome | GO:CC not extracellular matrix | sp|Q13601|KRR1_HUMAN | KRR1 small subunit processome component homolog | 18.78 | 18.67 | 18.6 |
| 1734 | KRT1 | Non-matrisome | Non-matrisome | GO:CC extracellular matrix (GO:0005576, GO:0005578 & GO:0031012) & GO:CC not extracellular matrix | sp|P04264|K2C1_HUMAN | Keratin, type II cytoskeletal 1 | 18.32 | 16.87 | 18.92 |
| 1735 | KRT10 | Non-matrisome | Non-matrisome | GO:CC not extracellular matrix | sp|P13645|K1C10_HUMAN | Keratin, type I cytoskeletal 10 | 16.41 | 15.08 | 17.08 |
| 1736 | KRT14 | Non-matrisome | Non-matrisome | GO:CC not extracellular matrix | sp|P02533|K1C14_HUMAN | Keratin, type I cytoskeletal 14 | 12.86 | 12.63 | 13 |
| 1737 | KRT17 | Non-matrisome | Non-matrisome | GO:CC not extracellular matrix | sp|Q04695|K1C17_HUMAN | Keratin, type I cytoskeletal 17 | 15.06 | 14.95 | 14.83 |
| 1738 | KRT18 | Non-matrisome | Non-matrisome | GO:CC not extracellular matrix | sp|P05783|K1C18_HUMAN | Keratin, type I cytoskeletal 18 | 15.82 | 16.51 | 16.26 |
| 1739 | KRT2 | Non-matrisome | Non-matrisome | GO:CC not extracellular matrix | sp|P35908|K22E_HUMAN | Keratin, type II cytoskeletal 2 epidermal | 16.33 | 15.06 | 17.13 |
| 1740 | KRT5 | Non-matrisome | Non-matrisome | GO:CC not extracellular matrix | sp|P13647|K2C5_HUMAN | Keratin, type II cytoskeletal 5 | 15.78 | 14.73 | 16.12 |
| 1741 | KRT7 | Non-matrisome | Non-matrisome | GO:CC not extracellular matrix | sp|P08729|K2C7_HUMAN | Keratin, type II cytoskeletal 7 | 17.82 | 16.73 | 16.28 |
| 1742 | KRT72 | Non-matrisome | Non-matrisome | GO:CC not extracellular matrix | sp|Q14CN4|K2C72_HUMAN | Keratin, type II cytoskeletal 72 | 20.36 | 20.26 | 20.3 |
| 1743 | KRT78 | Non-matrisome | Non-matrisome | GO:CC not extracellular matrix | sp|Q8N1N4|K2C78_HUMAN | Keratin, type II cytoskeletal 78 | 15.48 | 14.97 | 15.62 |
| 1744 | KRT8 | Non-matrisome | Non-matrisome | GO:CC not extracellular matrix | sp|P05787|K2C8_HUMAN | Keratin, type II cytoskeletal 8 | 13.25 | 13.84 | 13.18 |
| 1745 | KRT84 | Non-matrisome | Non-matrisome | GO:CC not extracellular matrix | sp|Q9NSB2|KRT84_HUMAN | Keratin, type II cuticular Hb4 | 18.57 | 19.26 | 18.83 |
| 1746 | KRT9 | Non-matrisome | Non-matrisome | GO:CC not extracellular matrix | sp|P35527|K1C9_HUMAN | Keratin, type I cytoskeletal 9 | 16.49 | 15.13 | 17.07 |
| 1747 | KRTCAP2 | Non-matrisome | Non-matrisome | GO:CC not extracellular matrix | sp|Q8N6L1|KTAP2_HUMAN | Keratinocyte-associated protein 2 | 19.97 | 20.2 | 20.26 |
| 1748 | KSR1 | Non-matrisome | Non-matrisome | GO:CC not extracellular matrix | sp|Q8IVT5|KSR1_HUMAN | Kinase suppressor of Ras 1 | 16.86 | 17.4 | 17.63 |
| 1749 | KTN1 | Non-matrisome | Non-matrisome | GO:CC not extracellular matrix | sp|Q86UP2|KTN1_HUMAN | Kinectin | 18.02 | 18.27 | 18.43 |
| 1750 | KYAT3 | Non-matrisome | Non-matrisome | GO:CC not extracellular matrix | sp|Q6YP21|KAT3_HUMAN | Kynurenine--oxoglutarate transaminase 3 | 17.33 | 18.19 | 18.03 |
| 1751 | KYNU | Non-matrisome | Non-matrisome | GO:CC not extracellular matrix | sp|Q16719|KYNU_HUMAN | Kynureninase | 15.4 | 17.92 | 18.84 |
| 1752 | L1CAM | Non-matrisome | Non-matrisome | GO:CC not extracellular matrix | sp|P32004|L1CAM_HUMAN | Neural cell adhesion molecule L1 | 17.18 | 15.15 | 14.87 |
| 1753 | L3HYPDH | Non-matrisome | Non-matrisome | GO:CC not extracellular matrix | sp|Q96EM0|T3HPD_HUMAN | Trans-3-hydroxy-L-proline dehydratase | 18.36 | 18.37 | 18.52 |
| 1754 | L3MBTL3 | Non-matrisome | Non-matrisome | GO:CC not extracellular matrix | sp|Q96JM7|LMBL3_HUMAN | Lethal(3)malignant brain tumor-like protein 3 | 14.83 | 15.25 | 15.08 |
| 1755 | LACTB | Non-matrisome | Non-matrisome | GO:CC not extracellular matrix | sp|P83111|LACTB_HUMAN | Serine beta-lactamase-like protein LACTB, mitochondrial | 16.38 | 17.88 | 17.53 |
| 1756 | LACTB2 | Non-matrisome | Non-matrisome | GO:CC not extracellular matrix | sp|Q53H82|LACB2_HUMAN | Endoribonuclease LACTB2 | 17.8 | 17.62 | 17.77 |
| 1757 | LAMA2 | Core matrisome | ECM Glycoproteins | GO:CC extracellular matrix (GO:0005576, GO:0005578 & GO:0031012) & GO:CC not extracellular matrix | sp|P24043|LAMA2_HUMAN | Laminin subunit alpha-2 | 15.53 | 15.81 | 15.97 |
| 1758 | LAMA3 | Core matrisome | ECM Glycoproteins | GO:CC extracellular matrix (GO:0005576, GO:0005578 & GO:0031012) & GO:CC not extracellular matrix | sp|Q16787|LAMA3_HUMAN | Laminin subunit alpha-3 | 14.26 | 14.94 | 14.64 |
| 1759 | LAMA4 | Core matrisome | ECM Glycoproteins | GO:CC extracellular matrix (GO:0005576, GO:0005578 & GO:0031012) & GO:CC not extracellular matrix | sp|Q16363|LAMA4_HUMAN | Laminin subunit alpha-4 | 15.57 | 18.14 | 17.56 |
| 1760 | LAMA5 | Core matrisome | ECM Glycoproteins | GO:CC extracellular matrix (GO:0005576, GO:0005578 & GO:0031012) & GO:CC not extracellular matrix | sp|O15230|LAMA5_HUMAN | Laminin subunit alpha-5 | 14.18 | 14.37 | 14.73 |
| 1761 | LAMB1 | Core matrisome | ECM Glycoproteins | GO:CC extracellular matrix (GO:0005576, GO:0005578 & GO:0031012) & GO:CC not extracellular matrix | sp|P07942|LAMB1_HUMAN | Laminin subunit beta-1 | 18.46 | 18.7 | 18.84 |
| 1762 | LAMB2 | Core matrisome | ECM Glycoproteins | GO:CC extracellular matrix (GO:0005576, GO:0005578 & GO:0031012) & GO:CC not extracellular matrix | sp|P55268|LAMB2_HUMAN | Laminin subunit beta-2 | 16.87 | 17.82 | 17.39 |
| 1763 | LAMB3 | Core matrisome | ECM Glycoproteins | GO:CC extracellular matrix (GO:0005576, GO:0005578 & GO:0031012) & GO:CC not extracellular matrix | sp|Q13751|LAMB3_HUMAN | Laminin subunit beta-3 | 13.82 | 14.28 | 14.11 |
| 1764 | LAMC1 | Core matrisome | ECM Glycoproteins | GO:CC extracellular matrix (GO:0005576, GO:0005578 & GO:0031012) & GO:CC not extracellular matrix | sp|P11047|LAMC1_HUMAN | Laminin subunit gamma-1 | 17.53 | 17.49 | 17.8 |
| 1765 | LAMC2 | Core matrisome | ECM Glycoproteins | GO:CC extracellular matrix (GO:0005576, GO:0005578 & GO:0031012) & GO:CC not extracellular matrix | sp|Q13753|LAMC2_HUMAN | Laminin subunit gamma-2 | 14.44 | 13.13 | 13.21 |
| 1766 | LAMC3 | Core matrisome | ECM Glycoproteins | GO:CC extracellular matrix (GO:0005576, GO:0005578 & GO:0031012) & GO:CC not extracellular matrix | sp|Q9Y6N6|LAMC3_HUMAN | Laminin subunit gamma-3 | 14.12 | 15.26 | 16.17 |
| 1767 | LAMP1 | Non-matrisome | Non-matrisome | GO:CC not extracellular matrix | sp|P11279|LAMP1_HUMAN | Lysosome-associated membrane glycoprotein 1 | 19.76 | 21.11 | 21.4 |
| 1768 | LAMP2 | Non-matrisome | Non-matrisome | GO:CC not extracellular matrix | sp|P13473|LAMP2_HUMAN | Lysosome-associated membrane glycoprotein 2 | 17.34 | 18.84 | 19.15 |
| 1769 | LAMTOR1 | Non-matrisome | Non-matrisome | GO:CC not extracellular matrix | sp|Q6IAA8|LTOR1_HUMAN | Ragulator complex protein LAMTOR1 | 18.91 | 19.66 | 19.93 |
| 1770 | LAMTOR2 | Non-matrisome | Non-matrisome | GO:CC not extracellular matrix | sp|Q9Y2Q5|LTOR2_HUMAN | Ragulator complex protein LAMTOR2 | 17.13 | 18.25 | 18.37 |
| 1771 | LAMTOR3 | Non-matrisome | Non-matrisome | GO:CC not extracellular matrix | sp|Q9UHA4|LTOR3_HUMAN | Ragulator complex protein LAMTOR3 | 17.31 | 18.44 | 18.79 |
| 1772 | LAMTOR4 | Non-matrisome | Non-matrisome | GO:CC not extracellular matrix | sp|Q0VGL1|LTOR4_HUMAN | Ragulator complex protein LAMTOR4 | 19.61 | 20.28 | 20.58 |
| 1773 | LAMTOR5 | Non-matrisome | Non-matrisome | GO:CC not extracellular matrix | sp|O43504|LTOR5_HUMAN | Ragulator complex protein LAMTOR5 | 18.11 | 18.69 | 19.13 |
| 1774 | LANCL1 | Non-matrisome | Non-matrisome | GO:CC not extracellular matrix | sp|O43813|LANC1_HUMAN | Glutathione S-transferase LANCL1 | 18.28 | 18.73 | 18.76 |
| 1775 | LANCL2 | Non-matrisome | Non-matrisome | GO:CC not extracellular matrix | sp|Q9NS86|LANC2_HUMAN | LanC-like protein 2 | 17.14 | 17.43 | 17.32 |
| 1776 | LAP3 | Non-matrisome | Non-matrisome | GO:CC not extracellular matrix | sp|P28838|AMPL_HUMAN | Cytosol aminopeptidase | 18.71 | 18.76 | 18.69 |
| 1777 | LARP1 | Non-matrisome | Non-matrisome | GO:CC not extracellular matrix | sp|Q6PKG0|LARP1_HUMAN | La-related protein 1 | 18.26 | 17.46 | 17.46 |
| 1778 | LARP7 | Non-matrisome | Non-matrisome | GO:CC not extracellular matrix | sp|Q4G0J3|LARP7_HUMAN | La-related protein 7 | 17.64 | 17.84 | 17.83 |
| 1779 | LARS1 | Non-matrisome | Non-matrisome | GO:CC not extracellular matrix | sp|Q9P2J5|SYLC_HUMAN | Leucine--tRNA ligase, cytoplasmic | 18.4 | 18.35 | 18.21 |
| 1780 | LAS1L | Non-matrisome | Non-matrisome | GO:CC not extracellular matrix | sp|Q9Y4W2|LAS1L_HUMAN | Ribosomal biogenesis protein LAS1L | 17.52 | 17.73 | 17.66 |
| 1781 | LASP1 | Non-matrisome | Non-matrisome | GO:CC not extracellular matrix | sp|Q14847|LASP1_HUMAN | LIM and SH3 domain protein 1 | 18.96 | 18.56 | 18.61 |
| 1782 | LBR | Non-matrisome | Non-matrisome | GO:CC extracellular matrix (GO:0005576, GO:0005578 & GO:0031012) & GO:CC not extracellular matrix | sp|Q14739|LBR_HUMAN | Delta(14)-sterol reductase LBR | 19.4 | 18.97 | 18.66 |
| 1783 | LCLAT1 | Non-matrisome | Non-matrisome | GO:CC not extracellular matrix | sp|Q6UWP7|LCLT1_HUMAN | Lysocardiolipin acyltransferase 1 | 18.64 | 18.74 | 18.43 |
| 1784 | LCMT1 | Non-matrisome | Non-matrisome | GO:CC not extracellular matrix | sp|Q9UIC8|LCMT1_HUMAN | Leucine carboxyl methyltransferase 1 | 19.09 | 19.33 | 19.33 |
| 1785 | LCP1 | Non-matrisome | Non-matrisome | GO:CC not extracellular matrix | sp|P13796|PLSL_HUMAN | Plastin-2 | 16.85 | 15.2 | 15.51 |
| 1786 | LDHA | Non-matrisome | Non-matrisome | GO:CC not extracellular matrix | sp|P00338|LDHA_HUMAN | L-lactate dehydrogenase A chain | 18.46 | 19.05 | 18.9 |
| 1787 | LDHB | Non-matrisome | Non-matrisome | GO:CC not extracellular matrix | sp|P07195|LDHB_HUMAN | L-lactate dehydrogenase B chain | 19.03 | 18.93 | 18.98 |
| 1788 | LEMD2 | Non-matrisome | Non-matrisome | GO:CC not extracellular matrix | sp|Q8NC56|LEMD2_HUMAN | LEM domain-containing protein 2 | 17.07 | 18.27 | 18.33 |
| 1789 | LEMD3 | Non-matrisome | Non-matrisome | GO:CC not extracellular matrix | sp|Q9Y2U8|MAN1_HUMAN | Inner nuclear membrane protein Man1 | 17.79 | 17.91 | 18.09 |
| 1790 | LETM1 | Non-matrisome | Non-matrisome | GO:CC not extracellular matrix | sp|O95202|LETM1_HUMAN | Mitochondrial proton/calcium exchanger protein | 18.48 | 18.81 | 18.68 |
| 1791 | LGALS1 | Matrisome-associated | ECM-affiliated Proteins | GO:CC extracellular matrix (GO:0005576, GO:0005578 & GO:0031012) & GO:CC not extracellular matrix | sp|P09382|LEG1_HUMAN | Galectin-1 | 19.36 | 19.79 | 19.81 |
| 1792 | LGALS3 | Matrisome-associated | ECM-affiliated Proteins | GO:CC extracellular matrix (GO:0005576, GO:0005578 & GO:0031012) & GO:CC not extracellular matrix | sp|P17931|LEG3_HUMAN | Galectin-3 | 19.07 | 20.52 | 20.25 |
| 1793 | LGALS3BP | Non-matrisome | Non-matrisome | GO:CC extracellular matrix (GO:0005576, GO:0005578 & GO:0031012) & GO:CC not extracellular matrix | sp|Q08380|LG3BP_HUMAN | Galectin-3-binding protein | 16.13 | 18.31 | 18 |
| 1794 | LGALS9 | Matrisome-associated | ECM-affiliated Proteins | GO:CC not extracellular matrix | sp|O00182|LEG9_HUMAN | Galectin-9 | 15.07 | 16.74 | 17.25 |
| 1795 | LGALSL | Non-matrisome | Non-matrisome | GO:CC not extracellular matrix | sp|Q3ZCW2|LEGL_HUMAN | Galectin-related protein | 14.14 | 14.53 | 15.08 |
| 1796 | LGMN | Non-matrisome | Non-matrisome | GO:CC extracellular matrix (GO:0005576, GO:0005578 & GO:0031012) & GO:CC not extracellular matrix | sp|Q99538|LGMN_HUMAN | Legumain | 16.65 | 16.78 | 17.06 |
| 1797 | LHPP | Non-matrisome | Non-matrisome | GO:CC not extracellular matrix | sp|Q9H008|LHPP_HUMAN | Phospholysine phosphohistidine inorganic pyrophosphate phosphatase | 16.44 | 17.6 | 17.6 |
| 1798 | LIG3 | Non-matrisome | Non-matrisome | GO:CC not extracellular matrix | sp|P49916|DNLI3_HUMAN | DNA ligase 3 | 17.46 | 17.21 | 17.25 |
| 1799 | LIMA1 | Non-matrisome | Non-matrisome | GO:CC not extracellular matrix | sp|Q9UHB6|LIMA1_HUMAN | LIM domain and actin-binding protein 1 | 18.54 | 17.39 | 17.8 |
| 1800 | LIMS1 | Non-matrisome | Non-matrisome | GO:CC not extracellular matrix | sp|P48059|LIMS1_HUMAN | LIM and senescent cell antigen-like-containing domain protein 1 | 18.14 | 18.23 | 18.02 |
| 1801 | LIN7C | Non-matrisome | Non-matrisome | GO:CC not extracellular matrix | sp|Q9NUP9|LIN7C_HUMAN | Protein lin-7 homolog C | 17.01 | 17.27 | 17.29 |
| 1802 | LLGL1 | Non-matrisome | Non-matrisome | GO:CC not extracellular matrix | sp|Q15334|L2GL1_HUMAN | Lethal(2) giant larvae protein homolog 1 | 17.22 | 17.38 | 17.47 |
[truncated: 376,687 more chars]
